# DCM alternatives for use in Steglich esterifications, for green and sustainable liquid crystal syntheses

**DOI:** 10.1039/d6ra00795c

**Published:** 2026-07-07

**Authors:** William C. Ogle, Calum J. Gibb, Stuart R. Berrow, Daniel L. Baker, Michael E. Ries, Richard J. Mandle

**Affiliations:** a School of Physics and Astronomy, University of Leeds Leeds LS2 9JT UK r.mandle@leeds.ac.uk; b School of Chemistry, University of Leeds Leeds LS2 9JT UK; c School of Mathematics, University of Leeds Leeds LS2 9JT UK

## Abstract

Dichloromethane (DCM) is a popular solvent for the synthesis of liquid crystalline materials owing to its solvating power, moderate polarity, and ease of removal. The environmental and toxicological issues surrounding DCM prompt us to explore alternatives. Here, we have screened an extensive selection of green and sustainable solvents to find the most efficacious DCM alternative for the synthesis of a conventional liquid crystal, CZP-5-N. Several factors are considered: yield, environmental impact, and health and safety concerns. The best performing solvent, dimethyl carbonate, was then used in the synthesis of common and novel liquid crystals, along with intermediates, giving excellent results, even for electron poor phenols. With esterifications prevalent in trending research areas like the ferroelectric nematic phase, this work allows a facile means for sustainability to be incorporated into synthetic pathways.

## Abstract 2

The use of dimethyl carbonate in Steglich esterifications has been shown to be highly efficacious in liquid crystal (LC) syntheses, as a drop-in replacement for DCM. A range of LC building blocks were then used to show its utility for LC synthesis.

## Graphical abstract

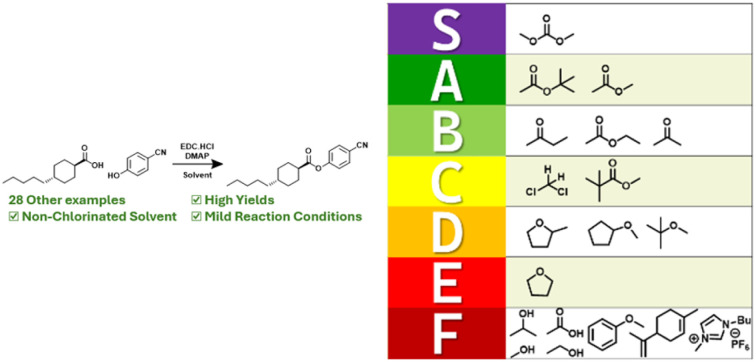

## 1 Introduction

**Fig. 1:**
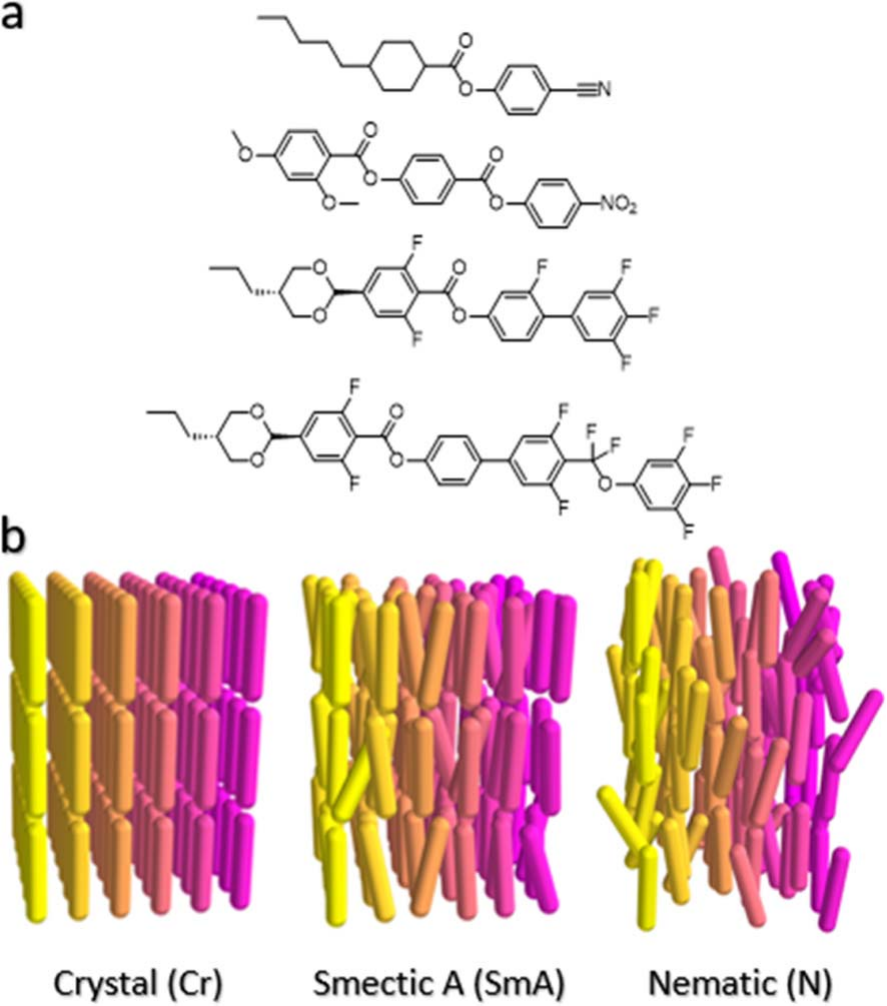
(a) Molecules that display liquid crystalline behaviour. From top to bottom CZP-5-N,^14^ RM734,^8^ DIO,^15^ RM2914.^16^ (b) The schematic representation of a crystal (Cr), smectic A (SmA), and nematic (N) mesophases.

Sustainable chemistry has become a leading area of study in recent years,^1–3^ however, one area in which it is still under studied is liquid crystals. At the international liquid crystal conference 2024, only one contribution was made to the sustainability category.^4^ Liquid crystals are materials that combine fluidity with some degree of molecular organisation intermediate between crystalline solids and isotropic liquids. In the nematic (N) phase, molecules retain a degree of orientational order without positional order, thus the N phase remains fluid (Fig. 1b).^5^ The combination of ordering, processability and stimuli response (*e.g.* to temperature, or electric/magnetic fields) underpins their widespread use in display technologies (*i.e.* LCDs).^6^ Differing degrees of positional and/or orientational ordering of the constituent molecules give rise to different phase types; for example, smectic phases possess highly diffuse layers (Fig. 1b) and the molecules can be orthogonal or tilted to the layers.^7^

The Steglich esterification is widely used in the preparation of carboxylate esters across synthetic chemistry, including the synthesis of liquid-crystalline compounds,^8–12^ due to their ability to connect different, readily available molecular fragments. These fragments are used to control the hydrophobicity, polarity, polarizability of the target liquid crystals. In polar liquid crystal phases, such as the ferroelectric nematic (N_F_) phase, esters can be crucial to the propagation of long-range polarity in the molecule.^13^

In this reaction, a carboxylic acid reacts with a carbodiimide to form an *O*-acylurea intermediate which then reacts with a suitable acyl transfer catalyst (*e.g.* DMAP) which then reacts rapidly with the alcohol/phenol to yield the desired ester. The use of modern coupling reagents such as 1-ethyl-3-(3-dimethylaminopropyl)carbodiimide (EDC HCl) ensures that the urea byproducts are trivially removed, overcoming issues with older reagents (*e.g. N*,*N*′-Dicyclohexylmethanediimine, DCC). The ambient reaction temperatures and high functional group tolerance of the Steglich Esterification make it far more attractive than Fischer-Speier Esterification, acid chlorides and so on.

The highly anisotropic shape of typical liquid crystalline materials, Fig. 1a, can result in poor solubility in organic solvents; however, DCM is widely used as a solvent for Steglich esterification in these materials owing to its unique solvation properties for the reagents, products and also various intermediates in this transformation.

A US EPA ban has greatly limited the usage of dichloromethane (DCM), with strict workplace controls to be implemented for the limited applications in which its use remains permitted.^17,18^ DCM is hepatotoxic, neurotoxic, carcinogenic, and the cause of at least 88 deaths since 1980 through acute exposure.^18–21^ DCM also contributes to stratospheric ozone depletion.^21,22^ However, it is difficult to replace DCM due to its solvating power, low flammability, minimal cost and high volatility. The US EPA ban aligns with the ACS Green Chemistry Institute Pharmaceutical Roundtable, who also expressed the importance of finding replacements for halogenated solvents.^21^ DCM is effectively the standard solvent for the Steglich esterification of liquid-crystalline materials,^12,23,24^ and identifying a greener yet equally effective alternative for this transformation is an important challenge. This is made non-trivial as liquid-crystalline materials display somewhat poor solubility in many organic solvents.

**Fig. 2:**
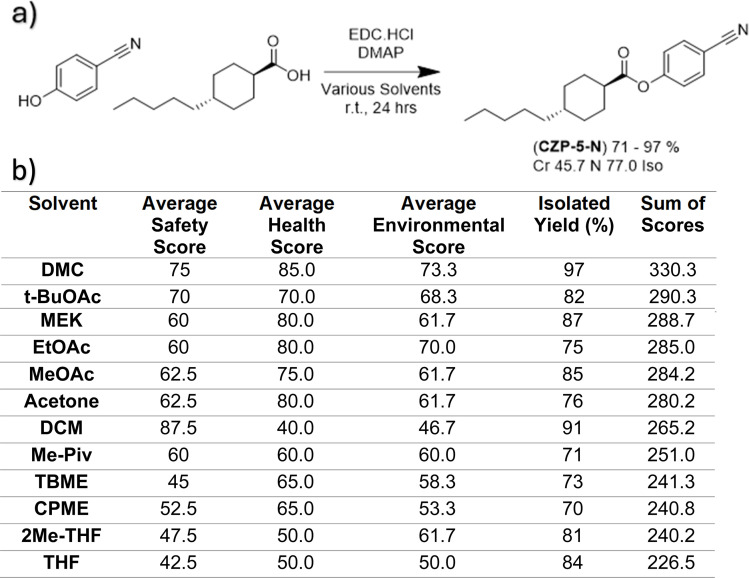
(a): the Steglich esterification for the liquid crystal CZP-5-N,^14^ (b): a table showing isolated yield each solvent studied in the synthesis of CZP-5-N *via* Steglich esterification of (*trans*)-4-pentylcyclohexane-1-carboxylic acid and 4-cyanophenol, also average health, safety, and environmental score, from the CHEM21 and GSK solvent selection guides. GSK safety score and is an average of flammability and explosion, and reactivity and stability scores. GSK health score is used directly. GSK environmental score is the average of the waste, environmental impact and life cycle scores. CHEM21 scores are reported already reported directly as safety, health, and environmental metrics. Me-Piv not assessed by GSK at time of writing. CHEM21 score is generated for Me-Piv using autoignition of ^*t*^BuOAc as it is an unreported metric for Me-Piv.^33,38^

Herein, we report a systematic approach for the replacement of DCM in the Steglich esterification to find the optimal solvent for a synthesis of the liquid crystal CZP-5-N,^14^Fig. 1a and 2a. The optimal solvent was then employed in the synthesis of a series of intermediates, other traditional and novel and polar liquid crystals.

## 2 Methods

### 2.1 Materials

Reagents and solvents were obtained from commercial suppliers and used as received. In-house materials were synthesised as per referenced methodology. In-house phenols used in the synthesis of esters: 5,^25^13 and 23,^26^19 and 20,^27^ in-house carboxylic acid used in the synthesis of esters: 9,^28^12,^29^11,^30^14.^31^

In-house carboxylic acids used in the synthesis of 15, 17, 18, 19, 20, 24 were all prepared *via* Suzuki coupling reactions using the methodology as described in Section 2.3, and S3.2.

### 2.2 General Steglich-esterification protocol

The relevant alcohol (1.0 equivalent), carboxylic acid (1.2 equivalents), EDC.HCl (1.5 equivalents) and DMAP (*ca.* 5 mol%) were added to a round bottomed flask or reaction vial. The relevant solvent (concentration *ca.* 0.3 M) was then added to the flask and left at room temperature with stirring for 24 hours. Dimers 1.0 equiv. carboxylic acid, 2.5 equiv. alcohol, 3.0 equiv. EDC.HCl, 5 mol% DMAP, r.t., 72 hours. The products were then isolated with flash chromatography. Solid compounds were then recrystalised using an appropriate solvent, typically ethanol or acetonitrile.

### 2.3 General Suzuki–Miyaura protocol

A biphasic mixture of THF (∼2 vol) and 2M aqueous K_2_CO_3_ (∼1 vol) was degassed by sparging with argon or N_2_ for 20 minutes. Aryl bromide (1.0 mol eqv.) and boronic acid/ester (1.2 mol eqv.) were added in sequence. The reaction was stirred and heated under reflux and under an atmosphere of dry nitgrogen gas. Pd-XPhos-G3 (cat.) was added in one portion. After 16 hours, the reaction was cooled to ambient temperature, then acidified to pH 2 with 2M aqueous HCl. Ethyl acetate was added; the organic phase was separated and retained. The aqueous phase was washed three times with ethyl acetate, then discarded. The combined organic phases were washed with brine three times. The organic phase was dried over MgSO_4_, which was subsequently removed by filtration. The volatiles were removed *in vacuo*, before recrystallisation of the crude material from isopropanol. The recrystalised material was collected by filtration, washed with cold ethanol (−18 °C), and dried under suction to yield the title compounds.

### 2.4 Purification

Chromatographic purification was performed using a Combiflash NextGen 300+ system (Teledyne Isco) using silica gel cartridges as the stationary phase and a hexane/ethyl acetate gradient as the mobile phase. Solid compounds were then recrystalised using an appropriate solvent, typically ethanol or acetonitrile.

### 2.5 Charactorisation

NMR was performed on a Bruker AV4 NEO 11.75 T (500 MHz 1H) NMR spectrometer (500-CP), or Bruker AV3HD 9.4 T (400 MHz 1H) NMR spectrometer (AV3HD-400). High resolution mass spectrometry data HRMS was collected using a Bruker MaXis Impact spectrometer with a positive ESI source (VIP-HES), the sample was introduced *via* direct infusion as solution in acetonitrile.

Phase sequences of compounds were performed with polarised optical microscopy (POM) and differential scanning calorimetry (DSC). Polarised light optical microscopy (POM) was performed using a Leica DM2700P polarised light microscope (Leica Microsystems (UK) Ltd, Milton Keynes, UK), equipped with 10× magnification, and a rotatable stage. A Linkam LTS 350 heating stage (Linkam Scientific Instruments Ltd, Redhill, UK) equipped with a TMS 93 temperature controller (Linkam Scientific Instruments Ltd, Redhill, UK) was used for temperature variation. Images were recorded using a XIMEA USB3 Camera (XIMEA GmbH, Münster, Germany) using IDS uEye Camera Software (IDS Imaging Development Systems Ltd, Farnham, UK). Samples were analysed sandwiched between untreated glass substrates, unless otherwise specified. Differential scanning calorimetry (DSC) was performed using a TA instruments Q20 or TA instruments Q2000 heat flux calorimeter, both with an RCS 90 cooling system for temperature control. Transitions reported on cooling except for the initial melt recorded on the second heating cycle.

Dielectric anisotropy was measured in cells with a planar alignment layer, 5 µm LC devices (AWAT, Poland), which contained 15 Ω per sq. ITO electrodes, SE130 planar alignment layer and were rubbed antiparallel. The quality of alignment was monitored with POM throughout. Temperatures were controlled with a THMS600 hot stage with Linkam 95 PE temperature controller. The Fréedericksz transitions were induced electrically with an out of plane electric field at 10 kHz as a function of voltage, across the nematic phase on cooling from 83 to 35 °C. An Agilent Precision LCR meter E4980A was used to measure capacitance.

Spontaneous polarisation measurements were undertaken using the current reversal technique, using an Agilent 33220A signal generator and a RIGOL DHO4204 high-resolution oscilloscope, with temperature control *via* an Instec HCS402 hot stage.

## 3 Results and discussion

### 3.1 Identifying candidate replacement solvents

The Hansen Solubility Parameters (HSP) were used as an initial starting point to find potential suitable replacements for DCM. Within HSP there are three parameters: dispersion (*δ*_D_), hydrogen bonding (*δ*_H_) and polar interactions (*δ*_P_), these give coordinates in ‘Hansen space’.^32^ Solvents with similar coordinates are more likely to dissolve similar solutes, making HSP a useful preliminary screening tool in assessing alternatives to DCM. With the use of other basic physical properties, and several solvent selection guides,^20,22,24,33,34^ a short-list was created of potential DCM replacements. The GSK solvent guide is particularly insightful as it breaks SHE score into seven factors: including a LCA of the solvent from cradle to grave, heath, flammability and stability factors. The ideal replacement must have minimal toxicity, be easy to recycle, inert, and easily removed.^35^ It is the solvation power of DCM that is most sought after, but this is also the aspect most difficult to replicate.

DCM occupies a rather unusual region of Hansen space (Table 1; *δ*_D_: 17, *δ*_P_: 7.3, *δ*_H_: 7.1). The high dispersion term stems from the polarisability and electronegativity of the chlorine atoms, while the moderate hydrogen bonding and polar interactions reflect a limited ability to engage in strong H-bond or polar interactions. Few greener solvents combine these features. Solvents with comparable dispersion parameters to DCM typically contain far more carbon (and/or oxygen) atoms but are widely different in there *δ*_P_ and *δ*_H_ for these reasons (*e.g.* limonene). Increasing molecular weight to reach the required *δ*_D_ predictably leads to increased boiling point and reduced vapour pressure, limiting ease of removal. Additionally, DCM is not flammable under typical lab conditions, distinguishing it from many classical organic solvents.

**Table 1:** Showing a range of solvents along with their HSP values,^32^ and physical characteristics, such as b.p., vapour pressure and flash point

Solvent	*δ*_D_	*δ*_P_	*δ*_H_	Boiling point (^o^C)	Vapour pressure (mbar at 20 °C)	Flash point (^o^C)
Dichloromethane (DCM)	17.0	7.3	7.1	40	350	N/A
Limonene	17.2	1.8	4.3	176	2.1	48
Methyl acetate (MeOAc)	15.5	7.2	7.6	57	228	−13
Ethyl acetate (EtOAc)	15.8	5.3	7.2	77	103	−4
^*s*^Butyl acetate (^*s*^BuOAc)	15	3.7	7.6	112	16	25
Isobutyl acetate (Iso-BuOAc)	15.1	3.7	6.3	117	20	18
^*t*^Butyl acetate (^*t*^BuOAc)	15	3.7	6.0	97	56	4
Methyl pivalate (Me-Piv)	15.1	4.0	5.1	101	23	6
Methyl laurate	15.4	3.2	3.2	261	0.0055 (at 25 °C)	139
Dimethyl carbonate (DMC)	15.5	8.6	9.7	90	53	16
Propylene carbonate	20.0	18	4.1	240	0.04	116
Acetone	15.5	10.4	7.0	56	245.3	−17
Methyl ethyl ketone (MEK)	16.0	9.0	5.1	80	95	−1
Cyrene	18.9	12.4	7.1	227	0.28	108
Tetrahydrofuran (THF)	16.8	5.7	8	65	170	−21.2
2-Methyl tetrahydrofuran (2Me-THF)	16.9	5	4.3	78	136	−10
^*t*^Butyl methyl ether (TBME)	14.8	4.3	5	55	268	−28
Cyclopentyl methyl ether (CPME)	16.7	4.3	4.3	107	42.7 (at 25 °C)	< 3
Anisole	17.8	4.4	6.9	154	13.3 (at 42.2 °C)	43

Solvent selection guides such as CHEM21, the GSK guide, and others typically recommend water, alcohols, esters, and some carboxylic acids as greener alternatives.^22,33,34^ However, protic solvents are incompatible with esterification reactions. There are many high boiling point solvents that are derived from biomass,^33^ such as, Cyrene, terpenoids, and esters of fatty acids, as seen in Table 1 below. Due to the energy demand on work up, these too were discounted from the study. It is likely that the environmental impact of a liquid–liquid extraction, or azeotropic removal of the solvent would negate positive effects of not using DCM to begin with, although a full life cycle assessment (beyond the scope of this work) would be required to confirm this. These solvents can potentially be removed *via* freeze-drying, but this is a far less flexible technique than removal under reduced pressure and is not widely used in the field. Ionic liquids were also considered but again, the product would likely be extracted into one of the solvents that is already being considered, making them a less favourable option. Solvent-less conditions are also worth considering, when possible,^36^ although few liquid crystal fragments have sufficiently low melting points/or are two costly to also facilitate the solvation role. Therefore, a selection of ‘green’ solvents and several classical solvents have been chosen, to assess their ability to perform the esterification.

For the initial stage of the work, we focused on the synthesis of an ester-containing liquid crystal, CZP-5-N (Fig. 2a),^37^ using various solvents. The reaction was performed with twelve solvents, including DCM as a control, with each solvent being tested on gram scale to obtain an isolated yield, seen below Fig. 2b. An isolated yield for gram scale reactions is a superior metric to HPLC conversion in this case. An in-depth characterisation and analysis of liquid crystalline materials, requires having access to >100 mg quantities of material in high purity (>99%). A HPLC/NMR yield in this case would not be sufficient, as isolated material is required for these analyses to fully study the applications, *e.g.* for display devices (LCDs).

Out of the several solvent selections guides, the numerical CHEM21 and GSK guides were the most extensive encompassing many solvents that that the others did not include.^33,38^ These served to provide the safety, health, and environmental (SHE) scores for the solvents, with the CHEM21 SHE scores being normalised against the GSK SHE scores. The averaged SHE score was then combined with the yield to provide a final score to indicate the suitability of a solvent for the reaction. The final scores can be seen in Fig. 2b with the score decreasing down the figure.

Dimethyl carbonate (DMC) was the only solvent that gave consistently higher yields than DCM (Fig. 2b) and is not only the most efficacious (97% isolated yield) but also scores best across the health (85) and environmental metrics (73.3). DCM scores highly for safety (87.5) mainly due to being non-flammable, but scores lowest across the health (40) and environmental metrics (46.7). Therefore, DCM achieved a total score of 265.2, whilst DMC scored a total of 330.3, with 400 being the maximum score.

One factor not considered here, owing to its regional and temporal variation, is pricing. At time of writing DCM is vastly cheaper than DMC. For the small-scale preparations typically undertaken in academic groups, this is not a huge concern ordinarily. Sustainability can be viewed through multiple lenses and is often said to have three pillars: environmental, economic and social.^35^ Perhaps then, it should be noted that the economies of scale need to shift to greener solvents for a truly sustainable solvent to emerge.

### 3.2 Scope of dimethyl carbonate utility in liquid crystal synthesis

**Fig. 3:**
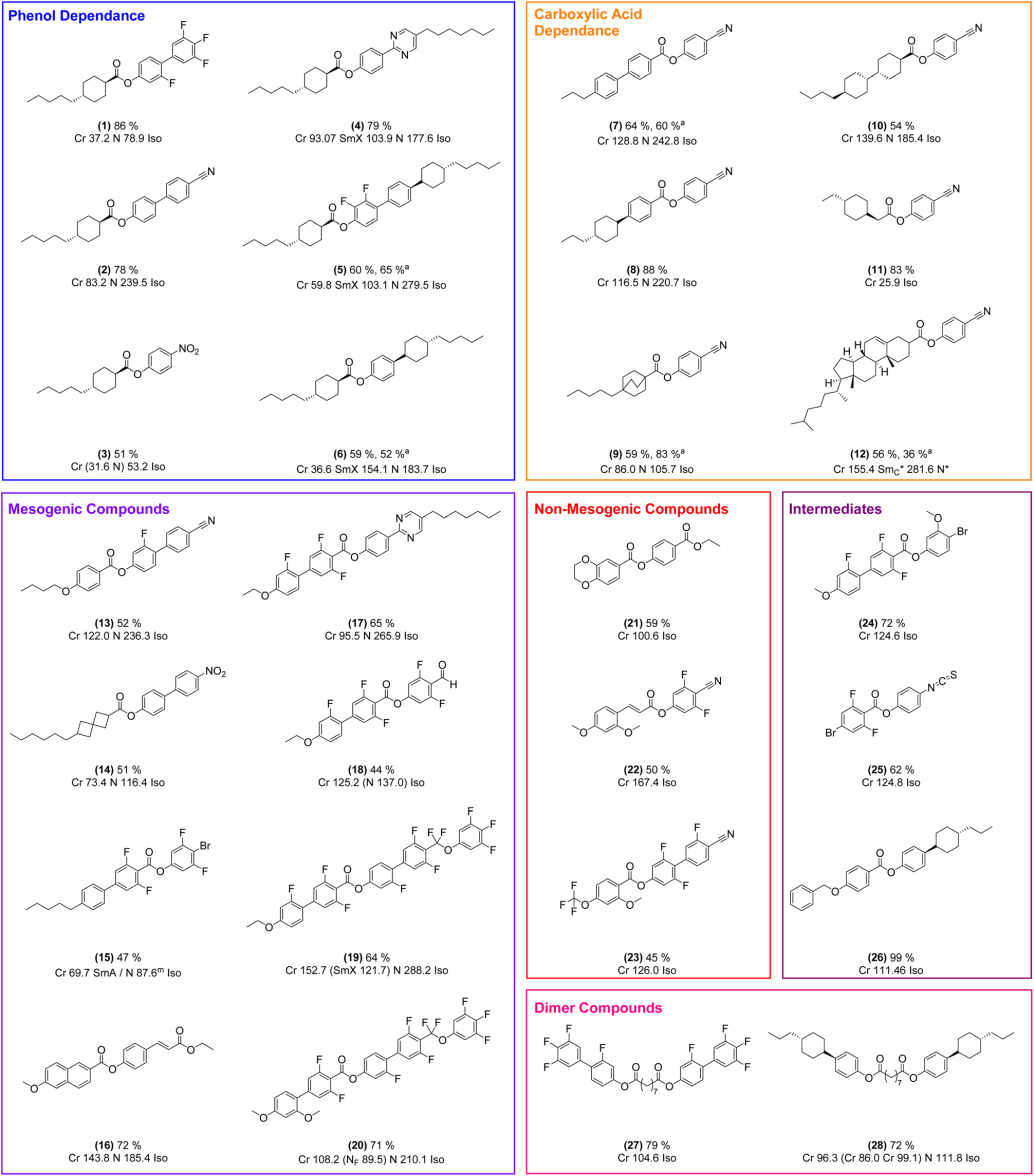
A series of liquid crystals and intermediates that have been prepared using DMC as the solvent. Yields refer to isolated yields of chromatographically and spectroscopically homogenous material. Transition temperatures, given in °C, at the onset. Typically, 1.0 mmol alcohol, 1.2 mmol carboxylic acid, 1.5 mmol EDC.HCl, *ca.* 5% DMAP, 30 mL DMC, r.t., 24 hours. Dimers 1.0 equiv. carboxylic acid, 2.5 equiv. alcohol, 3.0 equiv. EDC.HCl, 5 mol% DMAP, r.t., 72 hours. a compounds made using a 1 : 1 DMC: ^*t*^BuOAc solvent system. m SmA and N phases were seen to occur simultaneously by POM. N = nematic, N* = chiral nematic, N_F_ = ferroelectric nematic, SmA = smectic A, SmC* = chiral smectic C, SmX = unknown smectic phase, Cr = melting point, iso = isotropic liquid.

To assess the generality of DMC as a solvent for Steglich Esterification, we examined a representative series of phenols and carboxylic acids commonly used in liquid-crystal synthesis (Fig. 3). These substrates cover a broad range of electronic and steric environments and include motifs used in widely studied nematic and ferroelectric nematic materials.

Esterification with electron poor 4-hydroxybiphenyls afforded 1, and 2 (Fig. 3) in excellent yield (86% and 78%, respectively). Compound 2 exhibits a nematic phase, with transition temperatures in excellent agreement with literature values.^14^ Compound 1 is analogous to DIO (Fig. 1a),^15^ and CZGU-3-F, reported by Hobbs *et al.*^39^, and displays a non-polar nematic phase close to ambient temperature. Single ringed electron poor phenols such as 4-nitrophenol used in RM734 (Fig. 1a),^8^ gave a lower yield as expected (3, Fig. 3), whereas electron rich phenols (*e.g.*4) gave a high yield. It is however, more lipophilic starting materials which lead to a slightly lower yield than might be expected, this trend was also seen in 5 and 6.

To explore the influence of the carboxylic acid component, 4-cyanophenol (the phenol from CZP-5-N) was coupled with a variety of acids (7–12), including aromatic, primary, secondary, and tertiary aliphatic examples. As anticipated, electronic effects in the acid had only a minor influence on the reaction outcome. Instead, increased hydrophobicity again correlated with somewhat lower yields, suggesting that solubility in DMC is one of the main factors determining isolated yield.

To investigate the lipophilic/hydrophilic interactions a blend of 50 : 50 DMC (HSP: *δ*_D_ 15.5, *δ*_P_ 8.60, *δ*_H_ 9.7) and ^*t*^BuOAc which is far more lipophilic (HSP: *δ*_D_ 15.0, *δ*_P_ 3.7, *δ*_H_ 6.0) was used. As seen in Fig. 3 superscript a, the yields of 5, 6, 7, are near identical, with a slight decrease in the yield of 12, but a large increase in the yield of 9. This would suggest that the solvation is not the issue and there is another more nuanced bottleneck to higher yields.

The liquid crystalline behaviour of compounds 2,^14^3,^40^4,^41^7,^42^9,^37^ is in excellent agreement with earlier reports, with all materials displaying a non-polar nematic phase at elevated temperature.

As with the initial compounds investigated, better nucleophiles provided better yields, and as these compounds contain more polar components, lipophilic/hydrophobic interactions playing a lesser role. Many of the compounds displayed nematic phases (13, 14, 16), slightly unusually 15 showed two phases which were indistinguishable by DSC but could be seen going from N to SmA by POM on cooling.

Compounds 19 and 20 are structurally related to the DUZPUQU-*n*-F materials which display polar nematic phases and spontaneously break mirror symmetry to yield heliconical polar phases.^16,27^ Compound 20 exhibits a ferroelectric nematic phase, whilst compound 19 is non-polar. 17 and 18 using the same carboxylic acid as 19, did not show any polar phases.

Compounds 21, 22, and 23 are non-mesogenic and melt directly into isotropic liquids. Several intermediates for future synthesis were also produced (24, 25, 26). Interestingly, the esterification of benzyl protected hydroxybenzoic acid (26) gave an isolated yield of 99%; high yielding reactions such as this are especially attractive for telescoped multi-step flow processes (*e.g.* esterification-hydrogenolysis-esterification).^43^ Dimers were also produced 27, 28 in high yields, although requiring longer reaction times (72 hours), possibly reflecting the reduced solubility of the mono-esterified species, but there is likely to be a significant kinetic component too. The dimer 28 displays a short-range nematic phase, whereas 27 is non-mesogenic.

Overall, our results confirm that DMC is compatible with a wide range of functional groups and structural motifs relevant to liquid-crystal chemistry, including electron-poor phenols, aliphatic acids, and sterically hindered dimers. Importantly no transesterification was observed in the synthesis of any molecules presented in this study.

**Fig. 4:**
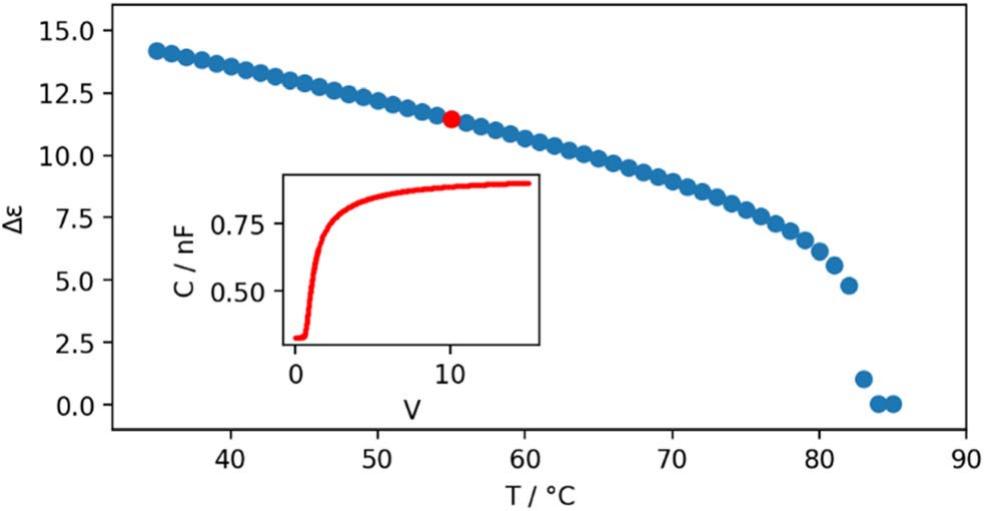
Dielectric anisotropy of compound 1 as a function of temperature. The inset shows the capacitance measured as a function of voltage at a temperature of 55 °C, shown with a red data point on the plot.

As compound 1 is a moderately close to room temperature single component nematic material; for completeness we measured the dielectric anisotropy (Δ*ε*) of this material as a function of temperature, reaching an unsaturated value of ∼14. The magnitude is consistent with strongly polar mesogens and confirms the substitution pattern here generates the expected dielectric properties (Fig. 4).

## 4 Conclusions

We have identified DMC as an effective and sustainable replacement for DCM in Steglich esterification, a transformation widely used in the synthesis of liquid-crystalline materials. Using a combination of isolated yields and composite sustainability metrics derived from the CHEM21 and GSK solvent-selection guides, DMC emerged as the top-performing solvent among more than twenty candidates.

DMC delivered excellent results across a diverse range of phenols, carboxylic acids, and more complex liquid-crystal building blocks, including sterically demanding dimers and useful synthetic intermediates. Electron-poor phenols behaved as expected, while lipophilic substrates showed slightly reduced yields due to solubility limitations; nevertheless, synthetically useful quantities of product were obtained in all cases. The method also tolerated representative mesogenic motifs without affecting their characteristic phase behaviour or dielectric properties.

DMC offers a practical, lower-toxicity alternative to DCM for the synthesis of ester-linked liquid-crystal materials. Its combination of good solvating ability, favourable environmental profile, and ease of removal makes it an attractive solvent for laboratories seeking to implement greener and more sustainable synthetic protocols.

## Author contributions

Chemical synthesis performed by WCO. Characterisation of liquid crystals performed by WCO, CJG, SRB. First draft was written by WCO and RJM, with contributions from all others.

## Conflicts of interest

There are no conflicts to declare.

## Supplementary Material

RA-016-D6RA00795C-s001

## Data Availability

DCM Alternatives for use in Steglich Esterifications, for Green and Sustainable Liquid Crystal Syntheses. The data associated with this paper are openly available from the University of Leeds Data Repository at DOI: https://doi.org/10.5518/1793. Supplementary information (SI) is available. See DOI: https://doi.org/10.1039/d6ra00795c.
