# Supplementary material for: DCM alternatives for use in Steglich esterifications, for green and sustainable liquid crystal syntheses
Source: RSC Adv. 2026 Jul 7;16(36):38442–8. doi: 10.1039/d6ra00795c (PMC13340035; doi:10.1039/d6ra00795c)
Supplement: Supplementary file 1 [file RA-016-D6RA00795C-s001.pdf]

This journal is © The Royal Society of Chemistry 2026

DCM Alternatives for use in Steglich Esterifications, for Green and Sustainable Liquid Crystal Syntheses ESI

William C. Ogle[1,2,3], Calum J. Gibb[2], Stuart R. Berrow,[1] Daniel L. Baker[1], Michael E. Ries[1], Richard J. Mandle [1,2*]

[1]School of Physics and Astronomy, University of Leeds, Leeds, LS2 9JT, UK
[2]School of Chemistry, University of Leeds, Leeds, LS2 9JT, UK
[3]School of Mathematics, University of Leeds, Leeds, LS2 9JT, UK
[*]r.mandle@leeds.ac.uk

Contents

DCM Alternatives for use in Steglich Esterifications, for Green and Sustainable Liquid Crystal Syntheses ESI 1

1. Methods 4

1.1 Chemical Synthesis 4

1.2. Chemical Characterisation 4

1.3. Phase characterisation 4

1.4. Dielectric Anisotropy Measurements 5

1.5 Spontaneous Polarisation 5

2. Supplemental results 5

2.1 Liquid Crystal Characterisation 5

2.2 Solvent Rankings 8

3 Organic Synthesis 9

3.1 Synthetic method for esterification 9

3.2 General Suzuki-Miyaura Protocol 9

3.3 Structural characterisation data for final compounds CZP-5-N and compounds 1-28 10

CZP-5-N | 4-cyanophenyl (1s,4r)-4-pentylcyclohexane-1-carboxylate 10

1 | trans-[3-fluoro-4-(3,4,5-trifluorophenyl)phenyl] 4-pentylcyclohexane-1-carboxylate 11

2 | [4-(4-cyanophenyl)phenyl] 4-pentylcyclohexane-1-carboxylate 12

3 | (4-nitrophenyl) 4-pentylcyclohexane-1-carboxylate 13

4 | 4-(5-heptylpyrimidin-2-yl)phenyl (1r,4s)-4-pentylcyclohexane-1-carboxylate 13

5 | 2,3-difluoro-4'-((1s,4r)-4-pentylcyclohexyl)-[1,1'-biphenyl]-4-yl (1s,4r)-4-entylcyclohexane-1-carboxylate 14

6 | 4-((1s,4R)-4-pentylcyclohexyl)phenyl (1s,4R)-4-pentylcyclohexane-1-carboxylate 15

7 | 4-cyanophenyl 4'-propyl-[1,1'-biphenyl]-4-carboxylate 15

8 | 4-cyanophenyl 4-((1s,4r)-4-propylcyclohexyl)benzoate 16

9 | 4-cyanophenyl 4-pentylbicyclo[2.2.2]octane-1-carboxylate 16

10 | 4-cyanophenyl (1s,1'r,4S,4'S)-4'-butyl-[1,1'-bi(cyclohexane)]-4-carboxylate 17

11 | 4-cyanobenzyl (1r,4r)-4-ethylcyclohexane-1-carboxylate..18

12 | 4’-cyanophenyl cholesteroate 18

13 | 4'-cyano-2-fluoro-[1,1'-biphenyl]-4-yl 4-butoxybenzoate 19

14 | 4'-nitro-[1,1'-biphenyl]-4-yl 6-hexylspiro[3.3]heptane-2-carboxylate 20

15 | 4-bromo-3,5-difluorophenyl 3,5-difluoro-4'-pentyl-[1,1'-biphenyl]-4-carboxylate 20

16 | (E)-4-(3-ethoxy-3-oxoprop-1-en-1-yl)phenyl 6-methoxy-2-naphthoate 21

17 | 4-(5-heptylpyrimidin-2-yl)phenyl 4'-ethoxy-2',3,5-trifluoro-[1,1'-biphenyl]-4-carboxylate .22

18 | 3,5-difluoro-4-formylphenyl 4'-ethoxy-2',3,5-trifluoro-[1,1'-biphenyl]-4-carboxylate 22

19 | 4'-(difluoro(3,4,5-trifluorophenoxy)methyl)-2,3',5'-trifluoro-[1,1'-biphenyl]-4-yl 4'-ethoxy-2',3,5-trifluoro-[1,1'-biphenyl]-4-carboxylate..23

20 | 4'-(difluoro(3,4,5-trifluorophenoxy)methyl)-2,3',5'-trifluoro-[1,1'-biphenyl]-4-yl 3,5-difluoro-2',4'-dimethoxy-[1,1'-biphenyl]-4-carboxylate 24

21 | 4-(ethoxycarbonyl)phenyl 2,3-dihydrobenzo[b][1,4]dioxine-6-carboxylate........................25

22 | 4-cyanophenyl (E)-3-(2,4-dimethoxyphenyl)acrylate 26

23 | 4'-cyano-2,3',6-trifluoro-[1,1'-biphenyl]-4-yl 2-methoxy-4-(trifluoromethoxy)benzoate.....26

24 | 4-bromo-3-methoxyphenyl 2',3,5-trifluoro-4'-methoxy-[1,1'-biphenyl]-4-carboxylate 27

25 | 4-isothiocyanatophenyl 4-bromo-2,6-difluorobenzoate 28

26 | 4-((1r,4s)-4-propylcyclohexyl)phenyl 4-(benzyloxy)benzoate..28

27 | bis(2,3',4',5'-tetrafluoro-[1,1'-biphenyl]-4-yl) nonanedioate..29

28 | bis(4-((1s,4r)-4-propylcyclohexyl)phenyl) nonanedioate...30

3.4 Novel biphenyl carboxylic acid intermediates 30

CA1 | 2,6-difluoro-4-(4-pentylphenyl)benzoic acid...30

CA2 | 4-(4-Ethoxy-2-fluorophenyl)-2,6-difluorobenzoic acid 31

CA3 | 4-(2,4-Dimethoxyphenyl)-2,6-difluorobenzoic acid...32

CA4 | 4-(4-Methoxy-2-fluorophenyl)-2,6-difluorobenzoic acid 32

4 Supplemental References 33

5 Compound Spectra, POM Images and DSC Thermograms 33

CZP-5-N | 4-cyanophenyl (1s,4r)-4-pentylcyclohexane-1-carboxylate.......................................33

1 | trans-[3-fluoro-4-(3,4,5-trifluorophenyl)phenyl] 4-pentylcyclohexane-1-carboxylate 36

2 | [4-(4-cyanophenyl)phenyl] 4-pentylcyclohexane-1-carboxylate 39

3 | (4-nitrophenyl) 4-pentylcyclohexane-1-carboxylate...41

4 | 4-(5-heptylpyrimidin-2-yl)phenyl (1r,4s)-4-pentylcyclohexane-1-carboxylate 43

5 | 2,3-difluoro-4'-((1s,4r)-4-pentylcyclohexyl)-[1,1'-biphenyl]-4-yl (1s,4r)-4-entylcyclohexane-1-carboxylate 46

6 | 4-((1s,4R)-4-pentylcyclohexyl)phenyl (1s,4R)-4-pentylcyclohexane-1-carboxylate................49

7 | 4-cyanophenyl 4'-propyl-[1,1'-biphenyl]-4-carboxylate 52

8 | 4-cyanophenyl 4-((1s,4r)-4-propylcyclohexyl)benzoate 55

9 | 4-cyanophenyl 4-pentylbicyclo[2.2.2]octane-1-carboxylate...58

10 | 4-cyanophenyl (1s,1'r,4S,4'S)-4'-butyl-[1,1'-bi(cyclohexane)]-4-carboxylate 61

11 | 4-cyanobenzyl (1r,4r)-4-ethylcyclohexane-1-carboxylate...64

12 | 4'-cyanophenyl cholesteroate 66

13 | 4'-cyano-2-fluoro-[1,1'-biphenyl]-4-yl 4-butoxybenzoate 69

14 | 4'-nitro-[1,1'-biphenyl]-4-yl 6-hexylspiro[3.3]heptane-2-carboxylate 73

15 | 4-bromo-3,5-difluorophenyl 3,5-difluoro-4'-pentyl-[1,1'-biphenyl]-4-carboxylate 75

16 | (E)-4-(3-ethoxy-3-oxoprop-1-en-1-yl)phenyl 6-methoxy-2-naphthoate 79

17 | 4-(5-heptylpyrimidin-2-yl)phenyl 4'-ethoxy-2',3,5-trifluoro-[1,1'-biphenyl]-4-carboxylate .82

18 | 3,5-difluoro-4-formylphenyl 4'-ethoxy-2',3,5-trifluoro-[1,1'-biphenyl]-4-carboxylate 86

19 | 4'-(difluoro(3,4,5-trifluorophenoxy)methyl)-2,3',5'-trifluoro-[1,1'-biphenyl]-4-yl 4'-ethoxy-2',3,5-trifluoro-[1,1'-biphenyl]-4-carboxylate...88

20 | 4'-(difluoro(3,4,5-trifluorophenoxy)methyl)-2,3',5'-trifluoro-[1,1'-biphenyl]-4-yl 3,5-difluoro-2',4'-dimethoxy-[1,1'-biphenyl]-4-carboxylate 92

21 | 4-(ethoxycarbonyl)phenyl 2,3-dihydrobenzo[b][1,4]dioxine-6-carboxylate........................96

22 | 4-cyanophenyl (E)-3-(2,4-dimethoxyphenyl)acrylate 99

23 | 4'-cyano-2,3',6-trifluoro-[1,1'-biphenyl]-4-yl 2-methoxy-4-(trifluoromethoxy)benzoate...102

24 | 4-bromo-3-methoxyphenyl 2',3,5-trifluoro-4'-methoxy-[1,1'-biphenyl]-4-carboxylate 105

25 | 4-isothiocyanatophenyl 4-bromo-2,6-difluorobenzoate 109

26 | 4-((1r,4s)-4-propylcyclohexyl)phenyl 4-(benzyloxy)benzoate...112

27 | bis(2,3',4',5'-tetrafluoro-[1,1'-biphenyl]-4-yl) nonanedioate..114

28 | bis(4-((1s,4r)-4-propylcyclohexyl)phenyl) nonanedioate..118

CA1 | 2,6-difluoro-4-(4-pentylphenyl)benzoic acid..121

CA2 | 4-(4-Ethoxy-2-fluorophenyl)-2,6-difluorobenzoic acid 123

CA3 | 4-(2,4-Dimethoxyphenyl)-2,6-difluorobenzoic acid...126

CA4 | 4-(4-Methoxy-2-fluorophenyl)-2,6-difluorobenzoic acid 129

1. Methods

1.1 Chemical Synthesis

Chemicals and solvents were purchased from commercial suppliers (Fluorochem, Merck, ChemScene, Ambeed) and used as received, without further purification. Reactions were performed in standard laboratory glassware at ambient temperature and atmosphere and were monitored by TLC with an appropriate eluent and visualised with 254 nm light. Chromatographic purification was performed using a Combiflash NextGen 300+ System (Teledyne Isco) with a silica gel stationary phase and a hexane/ethyl acetate gradient as the mobile phase, with detection made in the 200-800 nm range. Chromatographed materials were filtered through 200 nm PTFE frits and then subjected to re-crystallisation from an appropriate solvent system, such as ethanol or acetonitrile. Yields refer to chromatographically and spectroscopically homogenous material.

1.2. Chemical Characterisation

Chemical materials were characterised by NMR spectroscopy using either a Bruker Avance III HDNMR spectrometer operating at 400 MHz, 100.5 MHz or 376.4 MHz (^{1}H, $^{13}C\{^{1}H\}$ and ^{19}F, respectively) or a Bruker AV4 NEO 11.75T spectrometer operating at 500 MHz, 125.5 MHz or 470.5 MHz (^{1}H, $^{13}C\{^{1}H\}$). NMR were recorded in deuterated chloroform ($CDCl_3$) unless otherwise stated. High resolution mass spectrometry data HRMS was collected using a Bruker MaXis Impact spectrometer with a positive ESI source (VIP-HES), the sample was introduced via direct infusion as solution in acetonitrile.

1.3. Phase characterisation

Phase transition temperatures and associated enthalpies of transition were determined by differential scanning calorimetry (DSC) using a TA instruments Q2000 heat flux calorimeter with a liquid nitrogen cooling system for temperature control. Samples were measured with 10 ℃ min^{-1} heating and cooling rates. The transition temperatures and enthalpy values reported are averages obtained for duplicate runs. Phase transition temperatures were measured on cooling cycles for consistency between monotropic and enantiotropic phase transitions, while crystal melts were obtained on heating. Phase identification by polarised optical microscopy (POM) was performed using a Leica DM 2700 P polarised optical microscope equipped with a Linkam TMS 92 heating stage. Samples were studied sandwiched between two untreated glass coverslips.

1.4. Dielectric Anisotropy Measurements

Dielectric anisotropy was measured in cells with a planar alignment layer, 5 μm LC devices (AWAT, Poland), which contained 15 Ω/ sq. ITO electrodes, SE130 planar alignment layer and were rubbed antiparallel. The quality of alignment was monitored with POM throughout. Temperatures were controlled with a THMS600 hot stage with Linkam 95-PE temperature controller. The Fréedericksz transitions were induced electrically with an out of plane electric field at 10 kHz as a function of voltage, across the nematic phase on cooling from 83 to 35 °C. An Agilent Precision LCR meter E4980A was used to measure capacitance.

1.5 Spontaneous Polarisation

Spontaneous polarisation (Ps) measurements were undertaken using the current reversal technique, using an Agilent 33220A signal generator and a RIGOL DHO4204 high-resolution oscilloscope, with temperature control via an Instec HCS402 hot stage.

2. Supplemental results

2.1 Liquid Crystal Characterisation

The phase behaviour of each of the 29 materials described in the manuscript was determined by polarised optical microscopy (POM) and differential scanning calorimetry (DSC). Phase types were assigned based on characteristic POM textures, whereas transition temperatures and associated enthalpy were determined by DSC.

Nematic phases were assigned by the observation of 2 and 4-point schlieren brush singularities or marbled textures between untreated glass slides. The schlieren textures flashes under mechanical stress. The associated entropy changes are wholly consistent with this assignment. Due to the high nematic-isotropic transition temperatures ($T_{N\text{-}I}$), some materials decompose on repeated heating and cooling cycles to the isotropic (Iso) phase, as evidenced by the lack of reproducibility within the DSC thermograms.

The SmA phase was assigned by the observation of either a focal conic fan texture or the samples align homeotropically, and a uniformly dark texture is seen. A distinct first-order transition is observed at the SmA-N transition in the DSC thermograms with associated entropy changes consistent with the N-SmA transition.

The textures of the SmX phase are paramorphic with the preceding phase and hence differ depending on the phase sequence, however the transition is reversable on repeated heating and cooling cycles using DSC.

In the case of **12** the N* phase was characterised by the observation a typical oily streak texture. The optical flickering associated with director fluctuations in the N phase freezes upon entering the SmC* phase where a typical focal conic texture is observed.

For **20** the N_F phase was identified by its characteristic optical textures, The magnitude of the enthalpy associated with the phase transition, and by measurement of P_S *via* current reversal techniques, as seen in figure S1. Examples of textures from POM can be seen in figure S2. Examples of DSC thermograms can be seen in figure S3. See section 5 for POM and DSC thermograms for individual compounds.

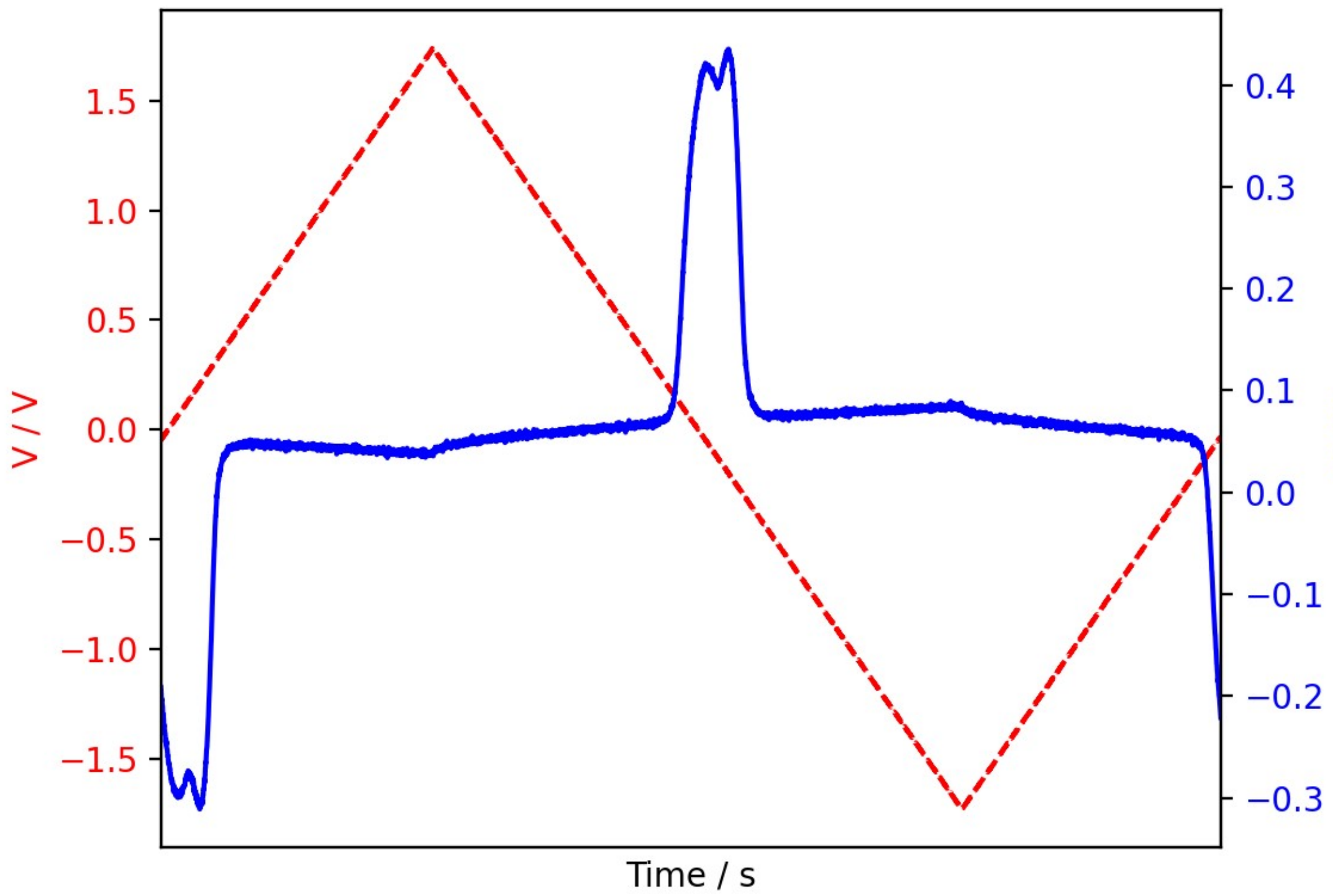

Figure S1. Ps measurement of **20** at 1 V, 20 Hz at 60 °C

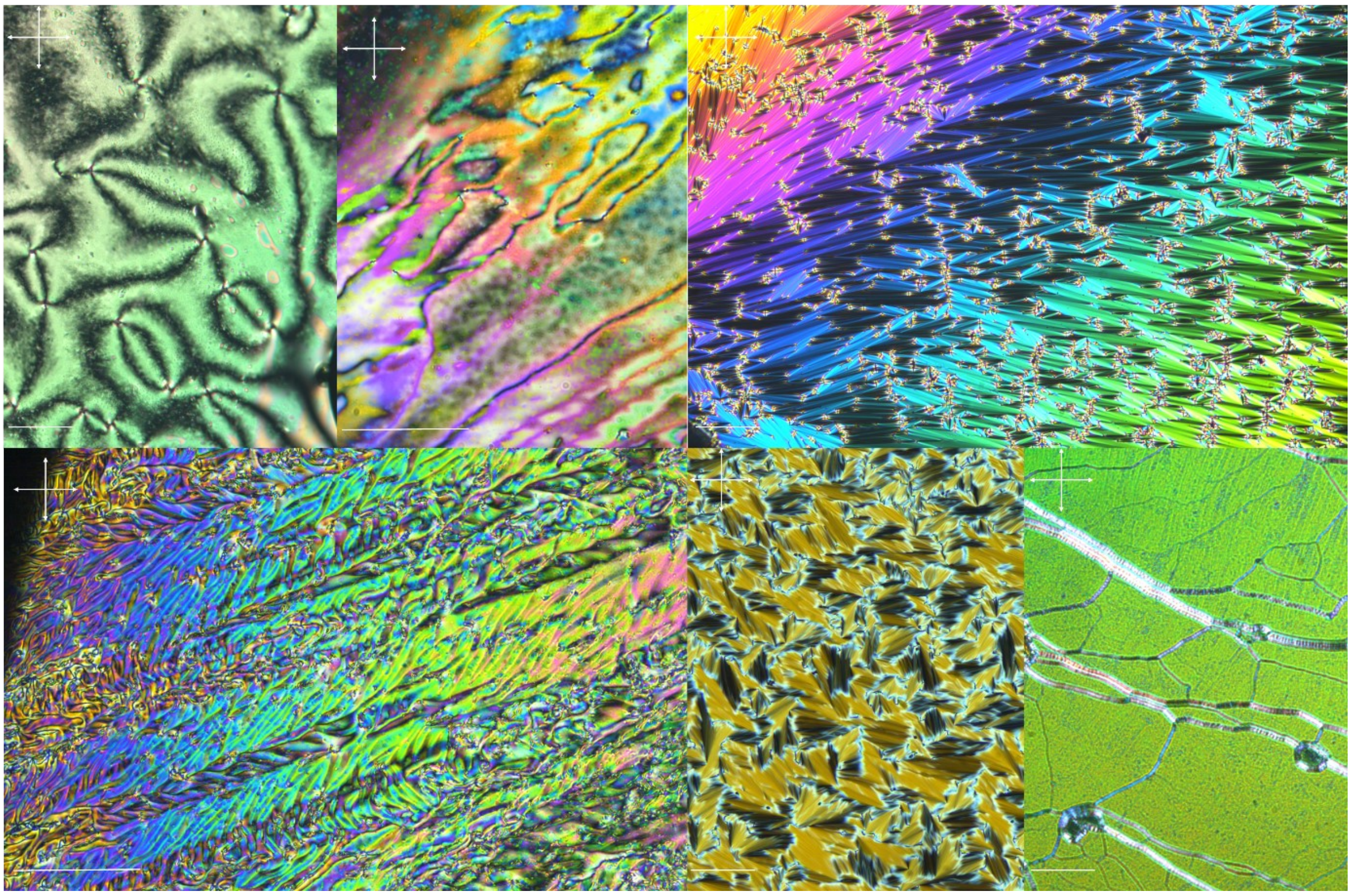

Figure S2, showing example textures: a nematic schlieren texture (**19** at 252 °C - top-left), nematic marble texture (**6** at 169 °C – top-centre-left) smectic A focal conic fan texture (**15** at 87 °C - top-right), ferroelectric nematic texture (**20** at 92 °C bottom-left), smectic C* focal conic texture (**12** at 150 °C - bottom-centre-right). Oil streak texture (**12** at 277 °C) – bottom-right). Scale bars (bottom left of each image) in white having a scale of 1 μm. Arrows (top left of each texture) show polariser direction). See section 5 for POM of individual compounds.

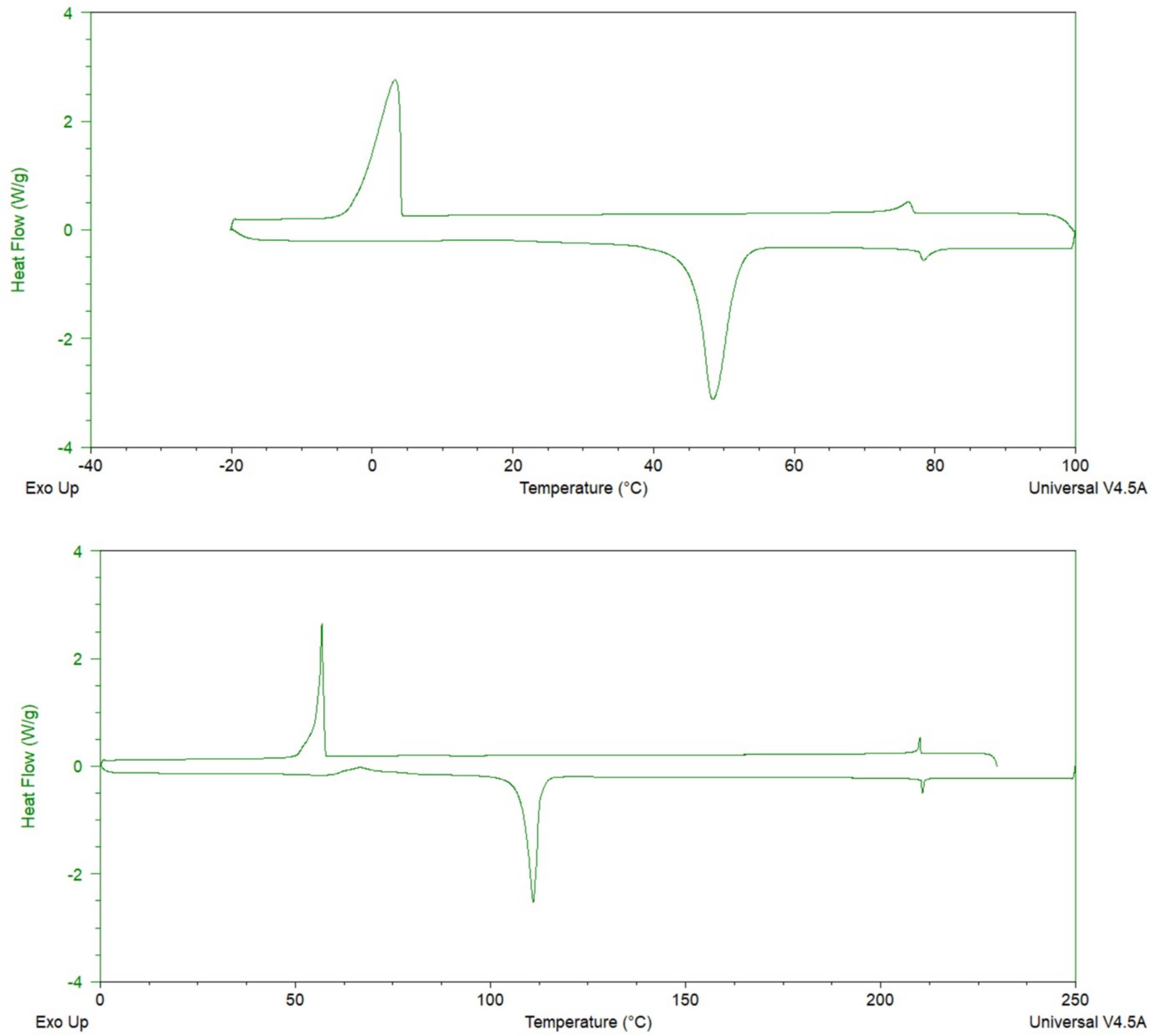

Figure S3, example DSC thermograms Q20, **CZP-5-N** (top), Q2000 **20** (bottom). See section 5 for DSC thermograms of individual compounds.

2.2 Solvent Rankings

Table S1 shows the ranking of the organic solvents used. GSK Safety score and is an average of Flammability and explosion, and reactivity and stability scores. GSK Health score is used directly. GSK environmental score is the average of the waste, environmental impact and life cycle scores. CHEM21 scores are reported already reported directly as safety, health, and

environmental metrics. Me-Piv not assessed by GSK at time of writing. CHEM21 score is generated for Me-Piv using autoignition of ^{t}BuOAc as it is an unreported metric for Me-Piv.

Average safety, health and environmental scores taken from CHEM21,[1] and GSK selection guides.[2] CHEM21 scores 1-10 with 1 being best. GSK from 10-1 with 10 being best. CHEM21 scores subtracted from 11 to rank them from 10-1 with 10 being best.

Average scores then multiplied by 10 to give them equal weighting to the yield scores. Yield and SHE score then summed together resulting in a final score.

Table S1. The SHE scores, yields and total score out of 400 for the solvents used in the initial screening reaction.

Solvent	Average Safety Score	Average Health Score	Average Environmental Score	Isolated Yield (%)	Sum of Scores
DMC	75	85.0	73.3	97	330.3
t-BuOAc	70	70.0	68.3	82	290.3
MEK	60	80.0	61.7	87	288.7
EtOAc	60	80.0	70.0	75	285.0
MeOAc	62.5	75.0	61.7	85	284.2
Acetone	62.5	80.0	61.7	76	280.2
DCM	87.5	40.0	46.7	91	265.2
Me-Piv	60	60.0	60.0	71	251.0
TBME	45	65.0	58.3	73	241.3
CPME	52.5	65.0	53.3	70	240.8
2Me-THF	47.5	50.0	61.7	81	240.2
THF	42.5	50.0	50.0	84	226.5

3 Organic Synthesis

3.1 Synthetic method for esterification

The relevant alcohol (1.0 equivalent), carboxylic acid (1.2 equivalents), EDC.HCl (1.5 equivalents) and DMAP (ca. 5 mol %) were added to a round bottomed flask or reaction vial. The relevant solvent (concentration ca. 0.3 M) was then added to the flask and left at room temperature with stirring for 24 hours (dimers 72 hours). The products were then isolated with flash chromatography using a Combiflash NextGen 300+ system (Teledyne Isco) using silica gel cartridges as the stationary phase and a hexane/ethyl acetate gradient as the mobile phase. Solid compounds were then recrystalised using an appropriate solvent, typically ethanol or acetonitrile.

3.2 General Suzuki-Miyaura Protocol

A biphasic mixture of THF (~ 2 vol) and 2M aqueous K_2CO_3 (~ 1 vol) was degassed by sparging with argon for 20 minutes. Aryl bromide (1.0 mol eqv) and boronic acid/ester (1.2 mol eqv) were added in sequence. The reaction was stirred and heated under reflux and under an atmosphere of dry nitrogen gas. PdXPhos G3 (cat.) was added in one portion. After 16h, the reaction was cooled to ambient temperature, then acidified to pH 2 with 2M aqueous HCl. Ethyl acetate was added; the organic phase was separated and retained. The aqueous phase was washed three times with ethyl acetate, then discarded. The combined organic phases were washed with brine three times. The organic phase was dried over $MgSO_4$, which was subsequently removed by filtration. The volatiles were removed *in vacuo*, before recrystallisation of the crude material from isopropanol. The recrystalised material was collected by filtration, washed with cold ethanol (-18 °C), and dried under suction to yield the title compounds.

3.3 Structural characterisation data for final compounds CZP-5-N and compounds 1-28

CZP-5-N | *4-cyanophenyl (1s,4r)-4-pentylcyclohexane-1-carboxylate*

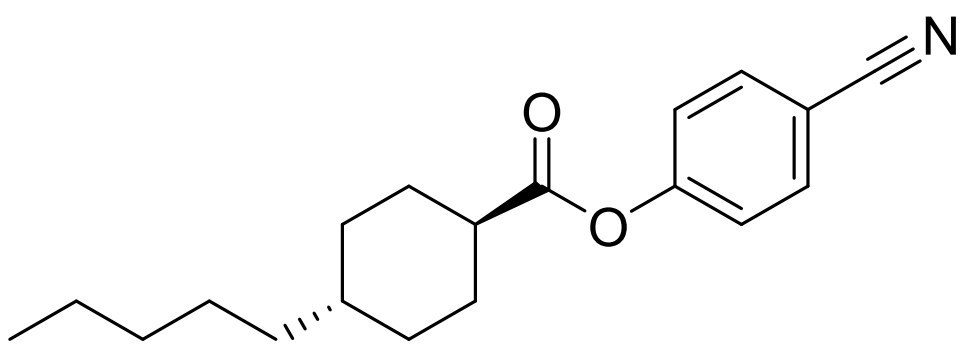

Standard Steglich esterification protocol was used. See table S2 for quantities of each reactant, reagent and yields.

Table S2, values of solvent, 4-cyanophenol, trans 4-pentylcyclohexanecarboxylic acid, EDC.HCl, DMAP used, along with the isolated yield.

Solvent (12ml)	4-4-Cyanophenol (mg, mmol)	trans 4-pentylcyclohexanecarboxylic acid (mg, mmol)	EDC.HCl (mg, mmol)	DMAP	Isolated Yield (%, mg)
DCM	470, 3.946	944, 4.760	1134, 5.916	Ca. 5 mol %	91, 1070
DMC	457, 3.834	942, 4.750	1117, 5.823	Ca. 5 mol %	97, 1119
Acetone	483, 4.055	955, 4.816	1144, 5.968	Ca. 5 mol %	76, 926
MeOAc	447, 3.753	976, 4.922	1183, 6.171	Ca. 5 mol %	85, 956

EtOAc	465, 3.904	942, 4.750	1062, 5.603	Ca. 5 mol %	75, 878
MEK	465, 3.904	931, 4.695	1115, 5.816	Ca. 5 mol %	87, 1021
THF	458, 3.845	930, 4.690	1095, 5.712	Ca. 5 mol %	84, 968
2-MeTHF	455, 3.820	962, 4.851	1130, 5.895	Ca. 5 mol %	81, 924
TBME	463, 3.887	961, 4.850	1088, 5.676	Ca. 5 mol %	73, 848
CPME	472, 3.962	923, 4.654	1099, 5.733	Ca. 5 mol %	70, 0.835
^{t}BuOAc	452, 3.794	942, 4.750	1156, 6.030	Ca. 5 mol %	82, 935
Methyl Pivalate	472, 3.962	933, 4.705	1113, 5.806	Ca. 5 mol %	71, 838

Transition Temperatures (°C): Cr 45.7 N 77.0 Iso

Transition Enthalpies (kJ/ mol): Cr 23.1 N 0.70 Iso

^{1}H NMR (501 MHz): 7.68 (2 H, dd, *J* = 6.6, 2.1 Hz), 7.20 (2 H, dd, *J* = 6.8, 2.1 Hz), 2.49 (1 H, tt, *J* = 12.3, 3.5 Hz), 2.17 – 2.08 (2 H, m), 1.88 (2 H, dd, *J* = 13.9, 3.2 Hz), 1.53 (2 H, qd, *J* = 13.3, 3.6 Hz), 1.37 – 1.15 (9 H, m), 0.98 (2 H, qd, *J* = 13.5, 3.5 Hz), 0.89 (3 H, t, *J* = 7.1 Hz).

^{13}C{^{1}H} NMR (126 MHz): 173.97, 154.37, 133.75, 122.87, 118.45, 109.66, 43.73, 37.21, 36.99, 32.29, 32.26, 29.04, 26.64, 22.81, 14.23.

HRMS: 301.1996 (- 1.1 ppm err) (M + H), 322.1782 (- 1.5 ppm err) (M + Na), expected 300.1959, 322.1778

1 | *trans-[3-fluoro-4-(3,4,5-trifluorophenyl)phenyl] 4-pentylcyclohexane-1-carboxylate*

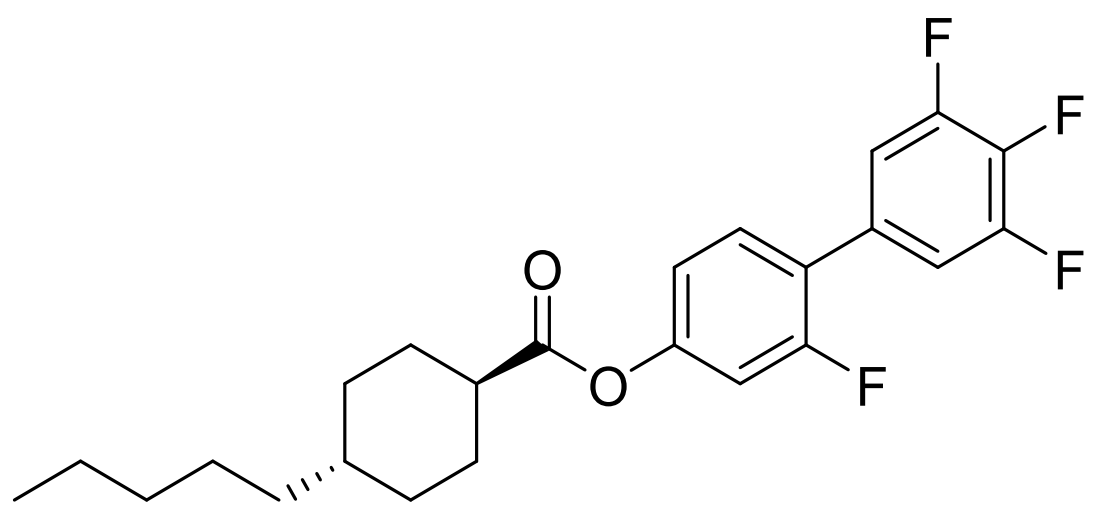

2,3',4',5'-tetrafluoro-[1,1'-biphenyl]-4-ol (230 mg, 0.950 mmol), (1r,4s)-4-pentylcyclohexane-1-carboxylic acid (236 mg, 1.190 mmol), EDC.HCl (291 mg, 1.518 mmol), DMAP (cat. amount). Standard Steglich esterification protocol was used. Yield: 86 %, 345 mg, 0.861 mmol

Transition Temperatures (°C):	Cr 37.2 N 78.7 Iso
Transition Enthalpies (kJ/ mol):	Cr 21.1 N 0.4 Iso
^{1}H NMR (400 MHz):	7.36 (1 H, t, *J* = 8.7 Hz), 7.15 (2 H, td, *J* = 7.5, 1.1 Hz), 6.98 (1 H, t, *J* = 2.6 Hz), 6.97 – 6.94 (1 H, m), 2.49 (1 H, tt, *J* = 12.2, 3.6 Hz), 2.14 (2 H, dd, *J* = 13.7, 3.3 Hz), 1.89 (2 H, dd, *J* = 13.7, 3.2 Hz), 1.56 (2 H, qd, *J* = 13.0, 3.5 Hz), 1.37 – 1.17 (9 H, m), 0.99 (2 H, qd, *J* = 13.3, 3.4 Hz), 0.90 (3 H, t, *J* = 7.2 Hz).
^{13}C{^{1}H} NMR (101 MHz):	174.34, 159.47 (d, *J* = 250.8 Hz), 151.88 (d, *J* = 11.0 Hz), 150.90 (ddd, *J* = 249.9, 10.1, 4.7 Hz), 139.11 (dd, *J* = 252.3, 15.1 Hz), 130.58 (d, *J* = 4.0 Hz), 123.71 (d, *J* = 14.0 Hz), 118.24 (d, *J* = 3.6 Hz), 113.29 (dd, *J* = 19.1, 3.2 Hz), 110.67 (t, *J* = 25.6 Hz), 43.74, 37.25, 37.03, 32.31 (d, *J* = 5.9 Hz), 29.10, 26.66, 22.83, 14.24.
^{19}F NMR (376 MHz):	-114.88 (1 F, t, *J* = 9.7 Hz), -134.30 (1 F, dd, *J* = 20.5, 8.7 Hz), -161.40 (1 F, tt, *J* = 20.6, 6.7 Hz).

2 | *[4-(4-cyanophenyl)phenyl] 4-pentylcyclohexane-1-carboxylate*

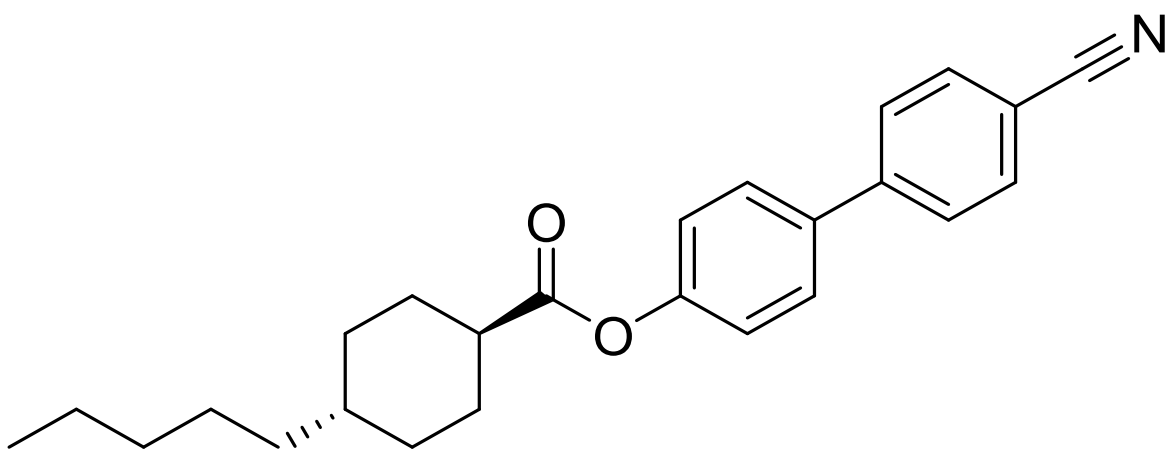

4'-hydroxy-[1,1'-biphenyl]-4-carbonitrile (191 mg, 0.978 mmol) (1r,4s)-4-pentylcyclohexane-1-carboxylic acid (236 mg, 1.190 mmol), EDC.HCl (301 mg, 1.570 mmol), DMAP (cat. amount). Standard Steglich esterification protocol was used. Yield: 78 %, 287 mg, 0.765 mmol

Transition Temperatures (°C):	Cr 83.2 N 239.5 Iso
Transition Enthalpies (kJ/ mol):	Cr 21.2 N 0.7 Iso
^{1}H NMR (400 MHz):	7.72 (2 H, d, *J* = 8.5 Hz), 7.65 (2 H, d, *J* = 8.4 Hz), 7.58 (2 H, dd, *J* = 6.4, 2.0 Hz), 7.18 (2 H, dd, *J* = 6.6, 2.0 Hz), 2.50 (1 H, tt, *J* = 12.2, 3.6 Hz), 2.15 (2 H, dd, *J* = 13.8, 3.6 Hz), 1.89 (2 H, dd, *J* = 13.6, 3.4 Hz), 1.57 (2 H, qd, *J* = 12.8, 3.5 Hz), 1.40 – 1.15 (9 H, m), 0.99 (2 H, qd, *J* = 13.5, 3.4 Hz), 0.89 (3 H, t, *J* = 7.0 Hz).

$^{13}C\{^1H\}$ NMR (101 MHz):	174.78, 151.52, 144.99, 136.80, 132.78, 128.42, 127.82, 122.46, 119.01, 111.14, 43.81, 37.27, 37.06, 32.39, 32.29, 29.16, 26.66, 22.82, 14.24.

3 | *(4-nitrophenyl) 4-pentylcyclohexane-1-carboxylate*

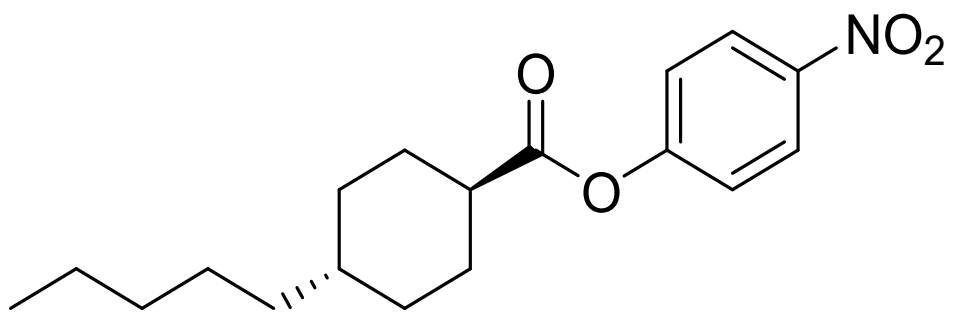

Nitrophenol (135 mg, 0.978 mmol), (1r,4s)-4-pentylcyclohexane-1-carboxylic acid (230 mg, 1.160 mmol), EDC.HCl (283 mg, 1.476 mmol), DMAP (cat. amount). Standard Steglich esterification protocol was used. Yield: 51 %, 159 mg, 0.498 mmol

Transition Temperatures (°C):	Cr (35.12 N) 52.2 Iso
Transition Enthalpies (kJ/ mol):	Cr (28.5 N) 0.2 Iso
^1H NMR (501 MHz):	8.26 (2 H, dd, *J* = 7.1, 2.1 Hz), 7.25 (2 H, dd, *J* = 7.3, 1.9 Hz),* 2.51 (1 H, tt, *J* = 12.2, 3.6 Hz), 2.14 (2 H, dd, *J* = 14.2, 3.7 Hz), 1.89 (2 H, dd, *J* = 13.8, 3.4 Hz), 1.55 (2 H, qd, *J* = 13.0, 3.5 Hz), 1.37 – 1.17 (9 H, m), 0.99 (2 H, qd, *J* = 13.3, 3.5 Hz), 0.89 (3 H, t, *J* = 7.1 Hz).<br><br>*Solvent Overlap
$^{13}C\{^1H\}$ NMR (126 MHz):	174.14, 156.14, 145.61, 125.57, 122.82, 44.02, 37.47, 37.26, 32.55, 32.53, 29.31, 26.91, 23.08, 14.50.
HRMS:	290.2115 (± 0.0 ppm) (M + H)*, expected 290.2115<br><br>*Nitro group reduced to amine group in flight

4 | *4-(5-heptylpyrimidin-2-yl)phenyl (1r,4s)-4-pentylcyclohexane-1-carboxylate*

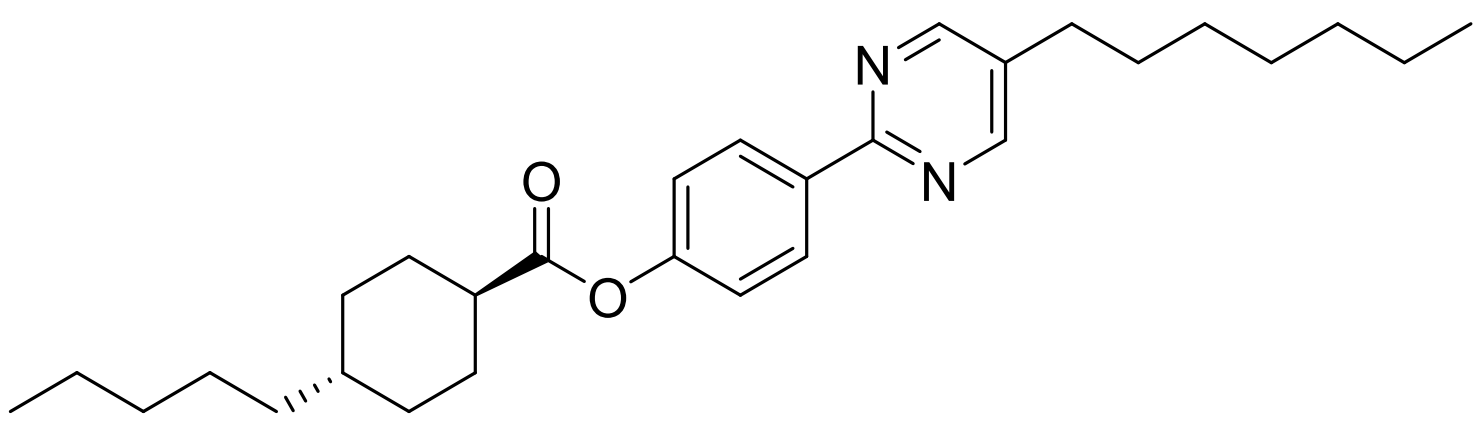

4-(5-heptylpyrimidin-2-yl)phenol (276 mg, 1.021 mmol), (1r,4s)-4-pentylcyclohexane-1-carboxylic acid (243 mg, 1.225 mmol), EDC.HCl (273 mg, 1.424 mmol), DMAP (cat. amount). Standard Steglich esterification protocol was used. Yield: 79 %, 363 mg, 0.805 mmol

Transition Temperatures (°C):	Cr 93.1 SmX 103.7 N 177.6 Iso

Transition Enthalpies (kJ/ mol): Cr 7.2 SmX 22.0 N 1.7 Iso

^{1}H NMR (400 MHz): 8.60 (2 H, s), 8.43 (2 H, dd, *J* = 6.9, 2.0 Hz), 7.18 (2 H, dd, *J* = 6.9, 2.0 Hz), 2.62 (2 H, d, *J* = 7.8 Hz), 2.49 (1 H, tt, *J* = 12.2, 3.6 Hz), 2.15 (2 H, dd, *J* = 13.9, 3.5 Hz), 1.88 (2 H, dd, *J* = 13.2, 2.7 Hz), 1.71 – 1.49 (4 H, m), 1.39 – 1.16 (17 H, m), 0.99 (2 H, qd, *J* = 13.1, 3.4 Hz), 0.92 – 0.83 (6 H, m).

^{13}C{^{1}H} NMR (101 MHz): 174.63, 162.04, 157.18, 152.92, 135.26, 133.10, 129.23, 121.78, 43.87, 37.30, 37.08, 32.43, 32.31, 31.87, 30.92, 30.33, 29.16, 26.68, 22.83, 22.76, 14.25, 14.21.

HRMS: 451.3317 (+ 0.5 ppm err) (M + H), expected 451.3320

5 | *2,3-difluoro-4'-((1s,4r)-4-pentylcyclohexyl)-[1,1'-biphenyl]-4-yl (1s,4r)-4-entylcyclohexane-1-carboxylate*

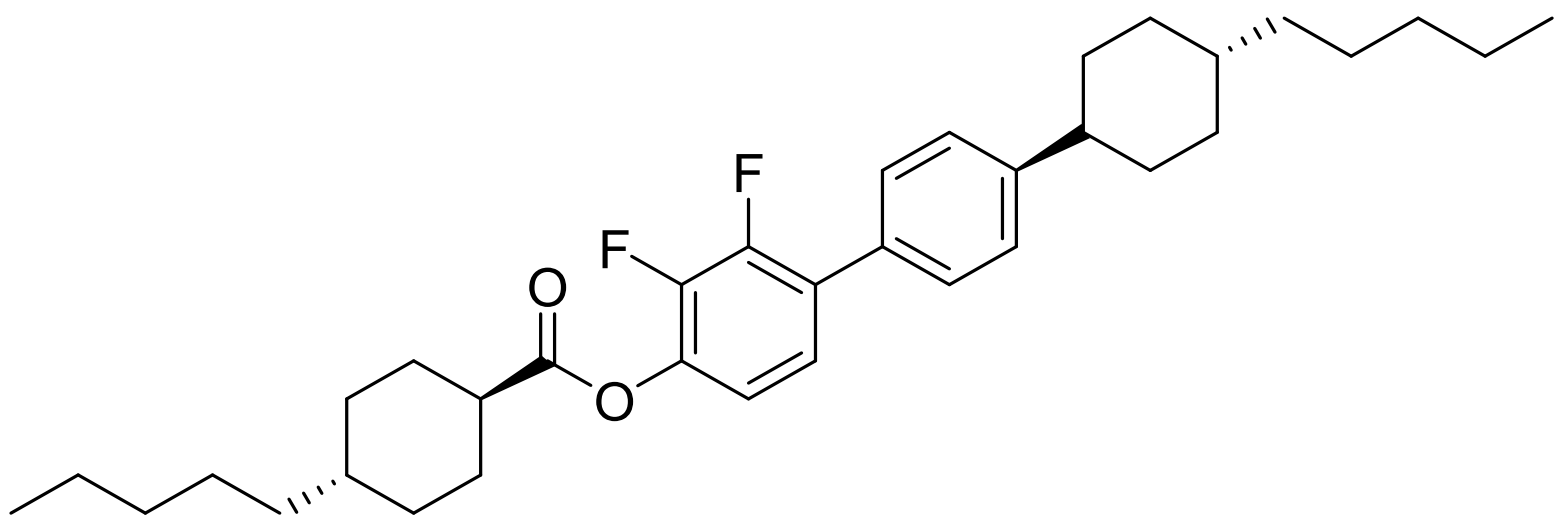

2,3-difluoro-4'-((1r,4s)-4-pentylcyclohexyl)-[1,1'-biphenyl]-4-ol (349 mg, 0.974 mmol), (1r,4s)-4-pentylcyclohexane-1-carboxylic acid (231 mg, 1.165 mmol), EDC.HCl (273 mg, 1.424 mmol), DMAP (cat. amount). Standard Steglich esterification protocol was used. Yield: 60 %, 315 mg, 0.585 mmol

Transition Temperatures (°C): Cr 59.8 SmX 103.1 N 279.5 Iso

Transition Enthalpies (kJ/ mol): Cr 11.6 SmX 0.2 N 1.6 Iso

^{1}H NMR (501 MHz): 7.43 (2 H, d, *J* = 6.5 Hz), 7.29 (2 H, d, *J* = 8.0 Hz), 7.16 (1 H, td, *J* = 8.5, 2.0 Hz), 6.94 (1 H, t, *J* = 7.7 Hz), 2.61 – 2.46 (2 H, m), 2.17 (2 H, d, *J* = 10.3 Hz), 1.96 – 1.85 (6 H, m), 1.60 (2 H, qd, *J* = 12.9, 3.1 Hz), 1.49 (2 H, qd, *J* = 12.5, 2.5 Hz), 1.37 – 1.18 (18 H, m), 1.08 (2 H, d, *J* = 10.7 Hz), 0.99 (2 H, t, *J* = 13.1 Hz), 0.93 – 0.86 (6 H, m).

^{13}C{^{1}H} NMR (126 MHz): 173.68, 148.60 (dd, *J* = 250.80 Hz), 148.23, 143.76 (dd, *J* = 250.74, 15.42 Hz), 138.54 (dd, *J* = 10.69, 1.60 Hz Hz), 131.93, 128.92 (d *J* = 2.88 Hz), 128.72 (d, *J* = 10.64 Hz), 127.28, 124.02 (t, *J* = 3.55 Hz), 118.44 (d, *J* = 4.03 Hz), 44.53, 43.49, 37.53, 37.45, 37.26, 37.00, 34.41, 33.72, 32.37, 32.35, 32.29, 29.14, 26.81, 26.66, 22.88, 22.83, 14.28, 14.25

^{19}F NMR (376 MHz):	-140.56 (1 F, dd, J = 20.2, 5.9 Hz), -151.02 (1 F, dd, J = 20.1, 5.2 Hz).

6 | *4-((1s,4R)-4-pentylcyclohexyl)phenyl (1s,4R)-4-pentylcyclohexane-1-carboxylate*

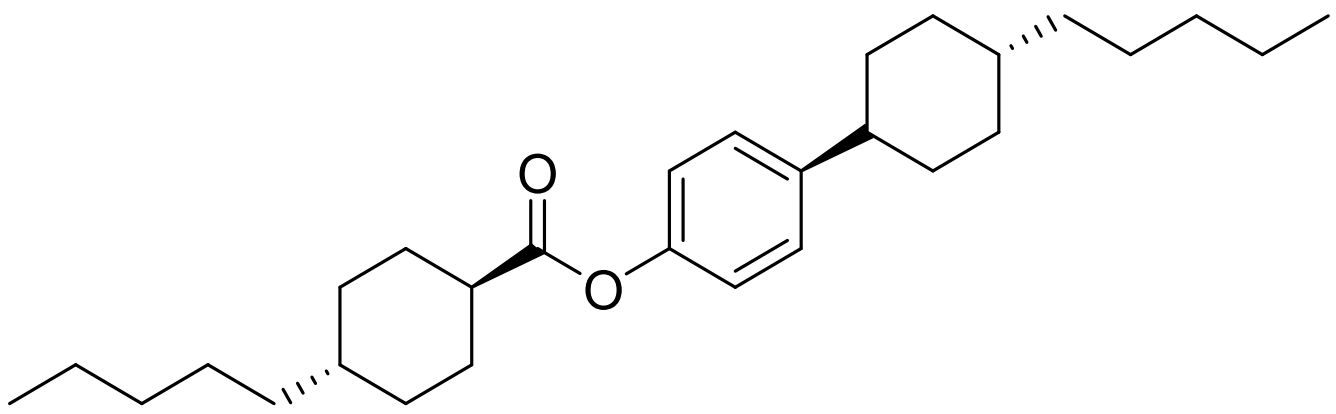

4-((1r,4s)-4-pentylcyclohexyl)phenol (298 mg, 1.209 mmol), (1r,4s)-4-pentylcyclohexane-1-carboxylic acid (227 mg, 1.145 mmol), EDC.HCl (276 mg, 1.440 mmol), DMAP (cat. amount). Standard Steglich esterification protocol was used. Yield: 59 %, 306 mg, 0.717 mmol

Transition Temperatures (°C):	Cr 36.6 SmX 154.1 N 183.7 Iso
Transition Enthalpies (kJ/ mol):	Cr 11.0 SmX 6.5 N 1.0 Iso
^{1}H NMR (501 MHz):	7.19 (2 H, dd, *J* = 13.1, 4.4 Hz), 6.96 (2 H, dd, *J* = 6.6, 2.1 Hz), 2.45 (2 H, tt, *J* = 12.2, 3.6 Hz), 2.12 (2 H, dd, *J* = 13.9, 3.7 Hz), 1.91 – 1.81 (6 H, m), 1.54 (2 H, qd, *J* = 13.5, 3.5 Hz), 1.41 (2 H, qd, *J* = 12.9, 3.1 Hz), 1.35 – 1.14 (18 H, m), 1.03 (2 H, qd, *J* = 12.8, 3.9), 0.96 (2 H, qd, *J* = 11.4, 3.2 Hz), 0.92 – 0.86 (6 H, m).
^{13}C{^{1}H} NMR (126 MHz):	175.09, 148.92, 145.33, 127.79, 121.27, 44.21, 43.80, 37.51, 37.42, 37.30, 37.07, 34.55, 33.72, 32.44, 32.35, 32.30, 29.18, 26.79, 26.67, 22.86, 22.83, 14.27, 14.25.
HRMS:	449.3386 (+ 0.9 ppm) (M + Na), expected 449.3390

7 | *4-cyanophenyl 4'-propyl-[1,1'-biphenyl]-4-carboxylate*

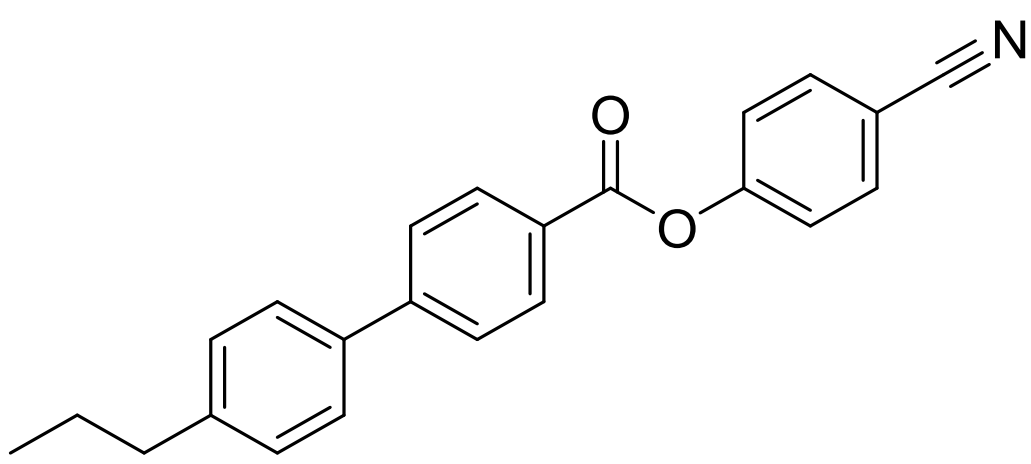

4-Cyanophenol (0.111 g, 0.932 mmol), 4'-propyl-[1,1'-biphenyl]-4-carboxylic acid (295 mg, 1.228 mmol), EDC.HCl (259 mg, 1.351 mmol), DMAP (cat. amount). Standard Steglich esterification protocol was used. Yield: 64 %, 205 mg, 0.600 mmol

Transition Temperatures (°C):	Cr 128.8 N 242.8 Iso

Transition Enthalpies (kJ/ mol):	Cr 24.7 N 1.1 Iso
^{1}H NMR (501 MHz):	8.24 (2 H, d, *J* = 8.5 Hz), 7.78 – 7.72 (4 H, m), 7.59 (2 H, d, *J* = 8.2 Hz), 7.39 (2 H, d, *J* = 8.7 Hz), 7.31 (2 H, d, *J* = 8.0 Hz), 2.66 (2 H, t, *J* = 7.7 Hz), 1.70 (2 H, h, *J* = 7.3 Hz), 0.99 (3 H, t, *J* = 7.3 Hz).
^{13}C{^{1}H} NMR (126 MHz):	164.43, 154.48, 147.06, 143.55, 137.06, 133.89, 130.97, 129.34, 127.30, 127.29, 127.08, 123.11, 118.46, 109.93, 37.87, 24.66, 13.99.
HRMS:	342.1488 (M + H), expected 342.1489

8 | *4-cyanophenyl 4-((1s,4r)-4-propylcyclohexyl)benzoate*

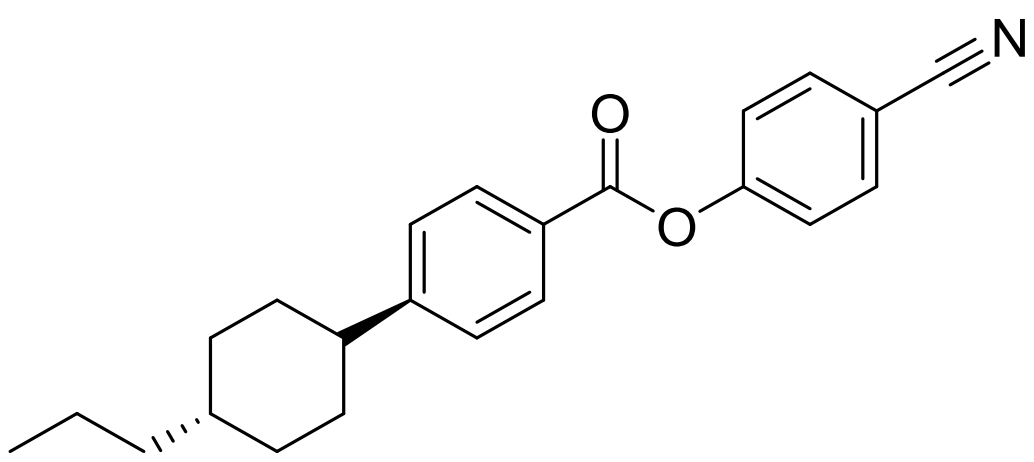

4-Cyanophenol (110 mg, 0.923 mmol), 4-((1s,4r)-4-pentylcyclohexyl)benzoic acid (335 mg, 1.221 mmol), EDC.HCl (294 mg, 1.534 mmol), DMAP (cat. amount). Standard Steglich esterification protocol was used. Yield: 88 %, 282, 0.811 mmol

Transition Temperatures (°C):	Cr 116.5 N 229.7 Iso
Transition Enthalpies (kJ/ mol):	Cr 7.2 N 0.8 Iso
^{1}H NMR (400 MHz):	8.10 (2 H, dd, *J* = 6.8, 1.9 Hz), 7.74 (2 H, dd, *J* = 6.8, 2.2 Hz), 7.36 (2 H, d, *J* = 3.2 Hz), 7.35 (2 H, d, *J* = 4.0 Hz), 2.58 (1 H, tt, *J* = 12.2, 3.3 Hz), 1.91 (4 H, t, *J* = 10.8), 1.50 (2 H, qd, *J* = 12.8, 3.1 Hz), 1.40 – 1.29 (3 H, m), 1.27 – 1.18 (2 H, m), 1.08 (2 H, qd, *J* = 13.7, 4.1 Hz), 0.91 (3 H, t, *J* = 7.2 Hz).
^{13}C{^{1}H} NMR (101 MHz):	164.20, 154.76, 154.22, 133.53, 130.29, 127.14, 125.98, 122.80, 118.16, 109.52, 44.72, 39.44, 36.77, 33.84, 33.18, 19.83, 14.21.

9 | *4-cyanophenyl 4-pentylbicyclo[2.2.2]octane-1-carboxylate*

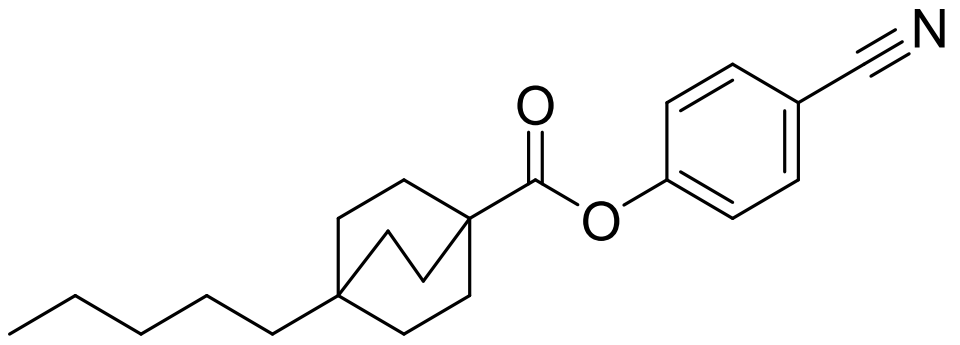

4-Cyanophenol (114 mg, 0.957 mmol), 4-pentylbicyclo[2.2.2]octane-1-carboxylic acid (268 mg, 1.195 mmol), EDC.HCl (283 mg, 1.523 mmol), DMAP (cat. amount). Standard Steglich esterification protocol was used. Yield: 59 %, 185 mg, 0.568 mmol

Transition Temperatures (°C):	Cr 86.0 N 105.7 Iso
Transition Enthalpies (kJ/ mol):	Cr 21.3 N 0.7 Iso
^{1}H NMR (400 MHz):	7.67 (2 H, dd, *J* = 6.7, 1.9 Hz), 7.17 (2 H, dd, *J* = 6.8, 2.2 Hz), 1.95 – 1.87 (6 H, m), 1.50 – 1.41 (6 H, m), 1.36 – 1.28 (2 H, m), 1.27 – 1.17 (4 H, m), 1.15 – 1.08 (2 H, m), 0.89 (3 H, t, *J* = 7.2 Hz).
^{13}C{^{1}H} NMR (101 MHz):	176.08, 154.62, 133.72, 122.89, 118.48, 109.57, 41.37, 39.70, 32.91, 30.62, 30.39, 28.70, 23.49, 22.82, 14.22.
HRMS:	326.2116 (- 0.6 ppm err) (M + H), expected 326.2115

10 | *4-cyanophenyl (1s,1'r,4S,4'S)-4'-butyl-[1,1'-bi(cyclohexane)]-4-carboxylate*

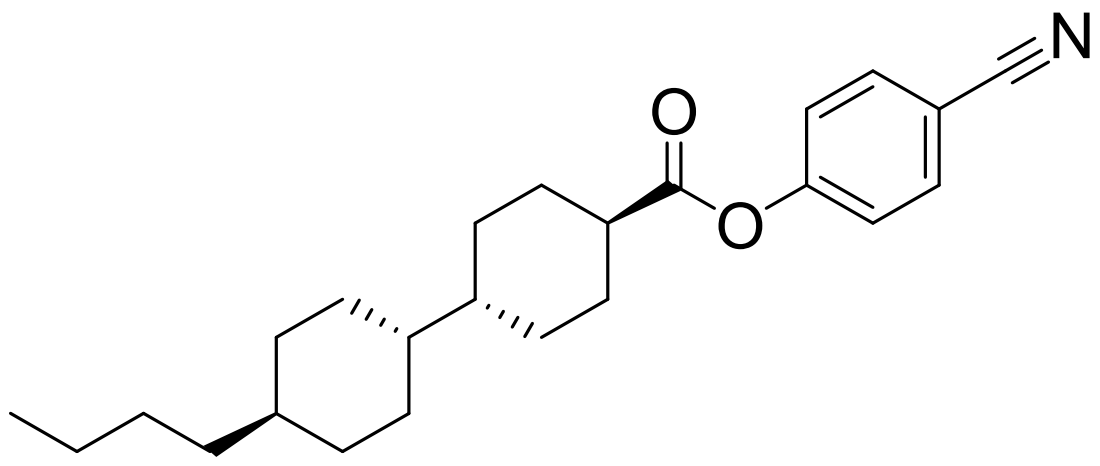

4-Cyanophenol (111 mg, 0.931 mmol), (1r,1's,4R,4'R)-4'-butyl-[1,1'-bi(cyclohexane)]-4-carboxylic acid (274, 1.028 mmol), EDC.HCl (309 mg, 1.612 mmol), DMAP (cat. amount). Standard Steglich esterification protocol was used. Yield: 184 mg, 0.5006 mmol, 53.77 %

Transition Temperatures (°C):	Cr 79.6 N 239.4 Iso
Transition Enthalpies (kJ/ mol):	Cr 23.5 N 1.4 Iso
^{1}H NMR (501 MHz):	7.67 (2 H, dd, *J* = 6.8, 2.0 Hz), 7.21 (2 H, dd, *J* = 6.6, 2.1 Hz), 2.47 (1 H, tt, *J* = 12.2, 3.5 Hz), 2.15 (2 H, d, *J* = 13.6 Hz), 1.86 (2 H, d, *J* = 11.5 Hz), 1.77 (2 H, d, *J* = 13.7), 1.71 (2 H, d, *J* = 12.8), 1.52 (2 H, q, *J* = 12.5 Hz), 1.33 – 1.21 (4 H, m), 1.18 – 1.02 (7 H, m), 0.98 (2 H, qd, *J* = 11.7, 2.9 Hz), 0.93 – 0.79 (5 H, m).

$^{13}C\{^{1}H\}$ NMR (126 MHz):	173.98, 154.38, 133.76, 122.87, 118.46, 109.66, 43.79, 43.32, 42.59, 38.00, 37.29, 33.68, 30.14, 29.39, 29.32, 29.18, 23.17, 14.30.
HRMS:	368.2585 (- 0.1 ppm err) (M + H), 390.2403 (+ 0.1 ppm err) (M + Na), expected 368.2585, 390.2404

11 | *4-cyanobenzyl (1r,4r)-4-ethylcyclohexane-1-carboxylate*

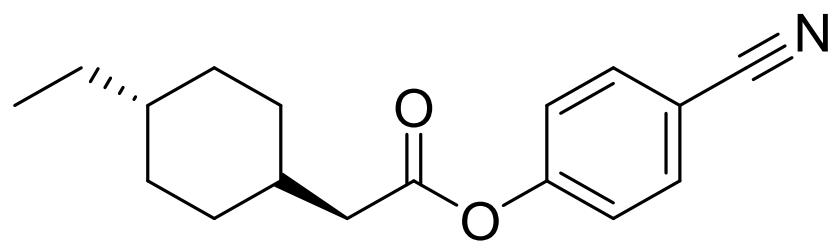

4-Cyanophenol (113 mg, 0.949 mmol), 2-((1r,4r)-4-ethylcyclohexyl)acetic acid (200 mg, 1.175 mmol), EDC.HCl (280 mg, 1.461 mmol), DMAP (cat. amount). Standard Steglich esterification protocol was used. Yield: 83 %, 213 mg, 0.785 mmol

Transition Temperatures (°C):	Cr 25.9 Iso
Transition Enthalpies (kJ/ mol):	Cr 22.1 Iso
^{1}H NMR (400 MHz):	7.67 (2 H, dd, *J* = 7.9, 2.1 Hz), 7.21 (2 H, dd, *J* = 6.8, 2.1 Hz), 2.45 (2 H, d, *J* = 6.6 Hz), 1.92 – 1.74 (5 H, m), 1.22 (2 H, p, *J* = 7.3 Hz), 1.14 – 1.01 (3 H, m), 0.96 (2 H, t, *J* = 11.0 Hz), 0.87 (3 H, t, *J* = 7.3 Hz).
$^{13}C\{^{1}H\}$ NMR (101 MHz):	170.82, 154.16, 133.76, 122.91, 118.41, 109.73, 42.11, 39.14, 35.30, 33.05, 32.53, 29.94, 11.60.

12 | *4'-cyanophenyl cholesteroate*

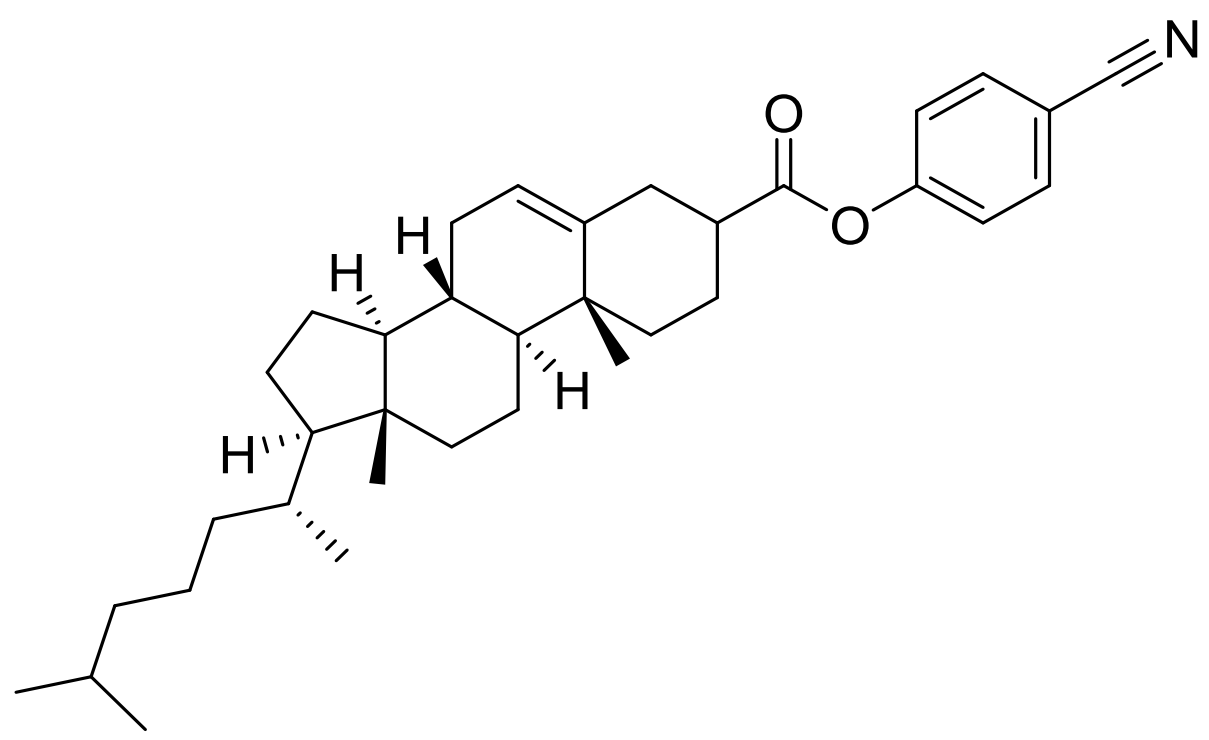

4-Cyanophenol (118 mg, 0.957 mmol), cholesterolic acid (495 mg, 1.193 mmol),EDC.HCl (308 mg, 1.607 mmol), DMAP (cat. amount). Standard Steglich esterification protocol was used. Yield: 56 %, 294 mg, 0.570 mmol

Transition Temperatures (°C):	Cr 155.4 SmC* 281.6 N* – compound degrades before becoming isotropic therefore all transitions given on heating
Transition Enthalpies (kJ/ mol):	Cr 24.9 SmC* 1.8 N*
^{1}H NMR (400 MHz):	7.68 (2 H, dd, *J* = 6.6, 1.9), 7.22 (2 H, dd, *J* = 6.9, 2.2), 5.42 (1 H, d, *J* = 5.1), 2.54 (2 H, d, *J* = 8.5), 2.38 (1 H, d, *J* = 8.6), 2.07 – 1.93 (5 H, m), 1.89 – 1.75 (2 H, m), 1.64 – 0.94 (24 H, m), 0.92 (3 H, d, *J* = 6.6), 0.87 (6 H, dd, *J* = 6.6, 1.7), 0.69 (3 H, s).
^{13}C{^{1}H} NMR (101 MHz):	173.50, 154.33, 140.40, 133.79, 122.88, 121.96, 118.44, 109.75, 56.92, 56.32, 50.39, 44.84, 42.46, 39.90, 39.67, 38.71, 37.09, 36.34, 35.94, 34.88, 32.05, 31.93, 28.38, 28.17, 25.32, 24.42, 23.99, 22.97, 22.72, 21.04, 19.49, 18.88, 12.02.

13 | *4'-cyano-2-fluoro-[1,1'-biphenyl]-4-yl 4-butoxybenzoate*

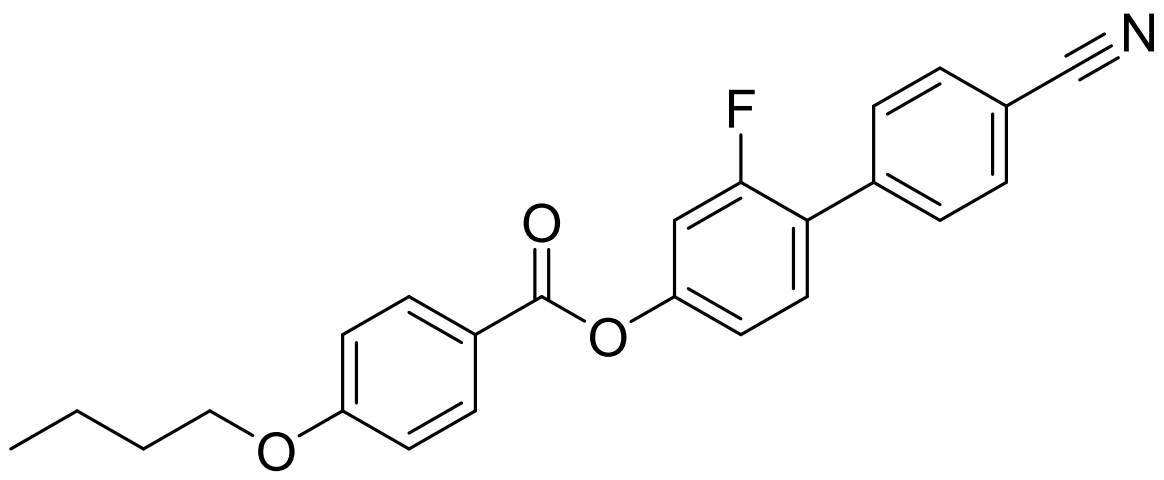

2'-fluoro-4'-hydroxy-[1,1'-biphenyl]-4-carbonitrile (144 mg, 0.675 mmol), 4-butoxybenzoic acid (238 mg, 1.225 mmol), EDC.HCl (377 mg, 1.967 mmol), DMAP (cat. amount). Standard Steglich esterification protocol was used. Yield: 52 %, 137 mg, 0.3518 mmol

Transition Temperatures (°C):	Cr 119.0 N 236.3 Iso
Transition Enthalpies (kJ/ mol):	Cr 25.3 N 1.0 Iso
^{1}H NMR (501 MHz):	8.14 (2 H, dd, *J* = 9.0, 1.9 Hz), 7.75 (2 H, d, *J* = 8.4 Hz), 7.66 (2 H, dd, *J* = 8.1, 1.2 Hz), 7.47 (1 H, t, *J* = 8.7 Hz), 7.18 – 7.10 (2 H, m), 6.98 (2 H, dd, *J* = 9.0, 1.9 Hz), 4.07 (2 H, t, *J* = 6.5 Hz), 1.82 (2 H, p, *J* = 7.2 Hz), 1.53 (2 H, h, *J* = 7.2 Hz), 1.00 (3 H, t, *J* = 7.4 Hz).
^{13}C{^{1}H} NMR (126 MHz):	164.58, 164.03, 159.70 (d, *J* = 251.1 Hz), 152.28 (d, *J* = 11.1 Hz), 139.99 (d, *J* = 1.4 Hz), 132.51 (d, *J* = 15.3 Hz), 130.90 (d, *J* = 4.1 Hz), 129.77 (d, *J* = 3.2 Hz), 124.80 (d, *J* = 13.1 Hz), 120.89, 118.88, 118.56 (d, *J* = 3.6 Hz), 114.57, 111.62, 111.05, 110.85, 68.22, 31.24, 19.33, 13.96.

^{19}F NMR (376 MHz): -114.61 (1 F, t, *J* = 9.9 Hz).

HRMS: 390.1501 (- 0.3 ppm err) (M + H), expected 390.1500

14 | *4'-nitro-[1,1'-biphenyl]-4-yl 6-hexylspiro[3.3]heptane-2-carboxylate*

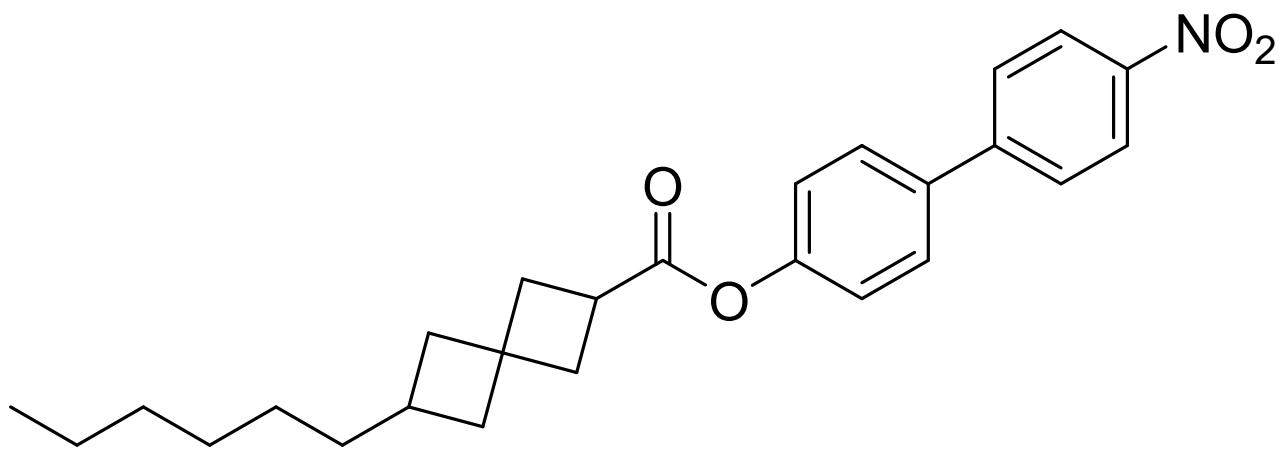

4'-nitro-[1,1'-biphenyl]-4-ol (122 mg, 0.567 mmol), 6-hexylspiro[3.3]heptane-2-carboxylic acid (70 mg, 0.314 mmol), EDC.HCl (120 mg, 0.626 mmol, DMAP (cat. amount). Yield 51 %, 67 mg, 0.159 mmol

Transition Temperatures (°C): Cr 73.4 N 116.4 Iso

Transition Enthalpies (kJ/ mol): Cr 17.6 N 1.5 Iso

^{1}H NMR (501 MHz): 8.29 (2 H, d, *J* = 8.8 Hz), 7.71 (2 H, d, *J* = 8.8 Hz), 7.62 (2 H, d, *J* = 8.6 Hz), 7.20 (2 H, d, *J* = 8.6 Hz), 3.26 (1 H, p, *J* = 8.5 Hz), 2.48 – 2.39 (2 H, m), 2.36 (1 H, dd, *J* = 11.5, 8.6 Hz), 2.29 – 2.18 (2 H, m), 2.15 – 2.03 (2 H, m), 1.67 (1 H, dd, *J* = 10.9, 7.2 Hz), 1.63 – 1.57 (1 H, m), 1.37 – 1.15 (10 H, m), 0.88 (3 H, t, *J* = 7.0 Hz).

$^{13}C\{^{1}H\}$ NMR (126 MHz): 174.15, 151.71, 147.22, 146.89, 136.35, 128.58, 127.86, 124.28, 122.47, 41.36, 40.82, 38.80, 38.06, 37.18, 36.85, 33.56, 32.04, 30.07, 29.42, 27.42, 22.79, 14.25.

15 | *4-bromo-3,5-difluorophenyl 3,5-difluoro-4'-pentyl-[1,1'-biphenyl]-4-carboxylate*

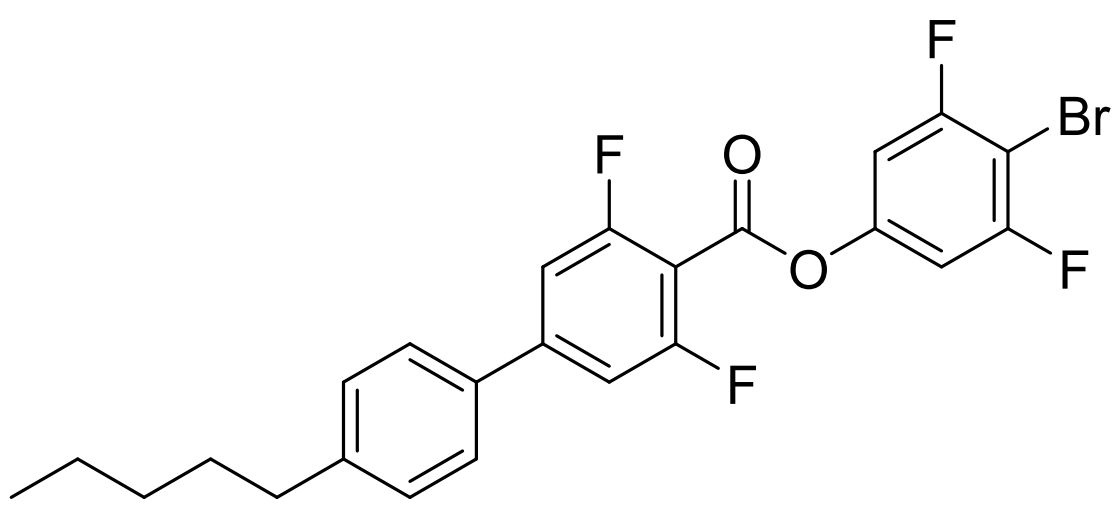

4-bromo-3,5-difluorophenol (312 mg, 1.025 mmol), 2,6-difluoro-4-(4-pentylphenyl)benzoic acid (227 mg, 1.086 mmol), EDC.HCl (299 mg, 1.560 mmol), DMAP (cat. amount). Standard Steglich esterification protocol was used. Yield: 240 mg, 0.4845 mmol, 47.26 %

Transition Temperatures (°C): Cr 71.5 SmC/ N 87.6 Iso – Nematic and smectic C phase present simultaneously

Transition Enthalpies (kJ/ mol): Cr 17.9 SmC/ N 3.1 Iso

^{1}H NMR (501 MHz, DMSO): 7.79 (2 H, d, *J* = 8.3 Hz), 7.72 (2 H, d, *J* = 10.6 Hz), 7.48 (2 H, d, *J* = 7.3 Hz), 7.34 (2 H, d, *J* = 8.5 Hz), 2.63 (2 H, t, *J* = 7.6 Hz), 1.60 (2 H, p, *J* = 7.6 Hz), 1.36 – 1.23 (4 H, m), 0.87 (3 H, t, *J* = 7.0 Hz).

^{13}C{^{1}H} NMR (126 MHz): 161.76 (dd *J* = 260.1, 7.2 Hz), 160.05 (dd, *J* = 248.6 Hz), 159.0, 149.32 (dt, *J* = 235.5, 13.0 Hz), 145.07, 134.83 (t, *J* = 2.3 Hz), 129.50, 127.03, 110.63 (dd, *J* = 22.5, 4.0 Hz), 107.20 – 106.47 (m), 95.76 (t, *J* = 24.6 Hz), 35.78, 31.62, 31.17, 22.68, 14.17.

^{19}F NMR (376 MHz): -103.20 (2 F, d, *J* = 7.9 Hz), -107.96 (2 F, d, *J* = 10.7 Hz).

16 | *(E)-4-(3-ethoxy-3-oxoprop-1-en-1-yl)phenyl 6-methoxy-2-naphthoate*

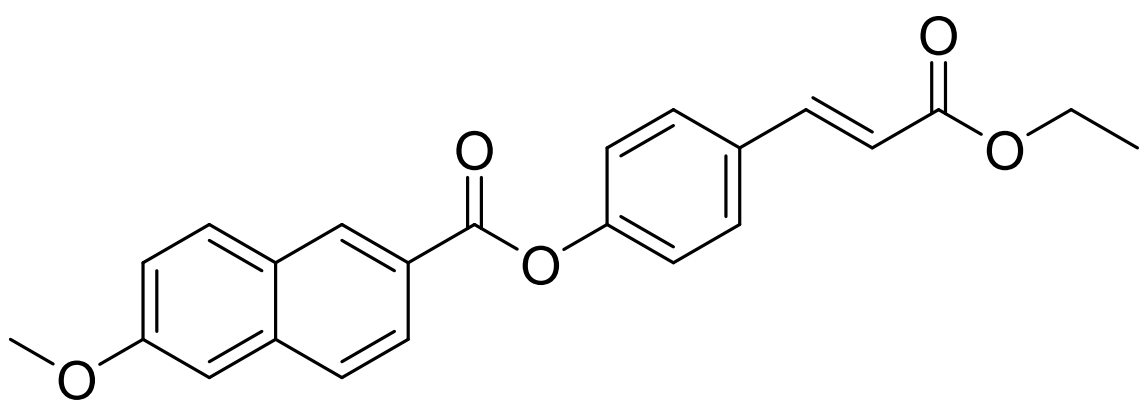

Ethyl (E)-3-(4-hydroxyphenyl)acrylate (198 mg, 1.030 mmol), 6-methoxy-2-naphthoic acid (268 mg, 1.325 mmol), EDC.HCl (407 mg, 2.123 mmol), DMAP (cat. amount). Standard Steglich esterification protocol was used. Yield 72 %, 280 mg, 0.744 mmol

Transition Temperatures (°C): Cr 139.6 N 185.4 Iso

Transition Enthalpies (kJ/ mol): Cr 33.2 N 0.4 Iso

^{1}H NMR (400 MHz): 8.70 (1 H, s), 8.15 (1 H, dd, *J* = 8.6, 1.8 Hz), 7.89 (1 H, d, *J* = 9.0 Hz), 7.83 (1 H, d, *J* = 8.7 Hz), 7.71 (1 H, d, *J* = 16.0 Hz), 7.61 (2 H, d, *J* = 8.6 Hz), 7.30 (2 H, d, *J* = 8.6 Hz), 7.24 (1 H, dd, *J* = 8.9, 2.5 Hz), 7.20 (1 H, d, *J* = 2.5 Hz), 6.43 (1 H, d, *J* = 16.0 Hz), 4.28 (2 H, q, *J* = 7.1 Hz), 3.97 (3 H, s), 1.35 (3 H, t, *J* = 7.1 Hz).

^{13}C{^{1}H} NMR (101 MHz): 167.07, 165.32, 160.12, 152.69, 143.71, 137.80, 132.33, 131.99, 131.22, 129.40, 128.04, 127.28, 126.29, 124.27, 122.52, 120.09, 118.56, 105.91, 60.71, 55.60, 14.48.

HRMS: 377.1384 (- 0.1 ppm err) (M + H), 399.1203 (- 0.1 ppm err) (M + Na), expected 377.1384, 399.1203

17 | *4-(5-heptylpyrimidin-2-yl)phenyl 4'-ethoxy-2',3,5-trifluoro-[1,1'-biphenyl]-4-carboxylate*

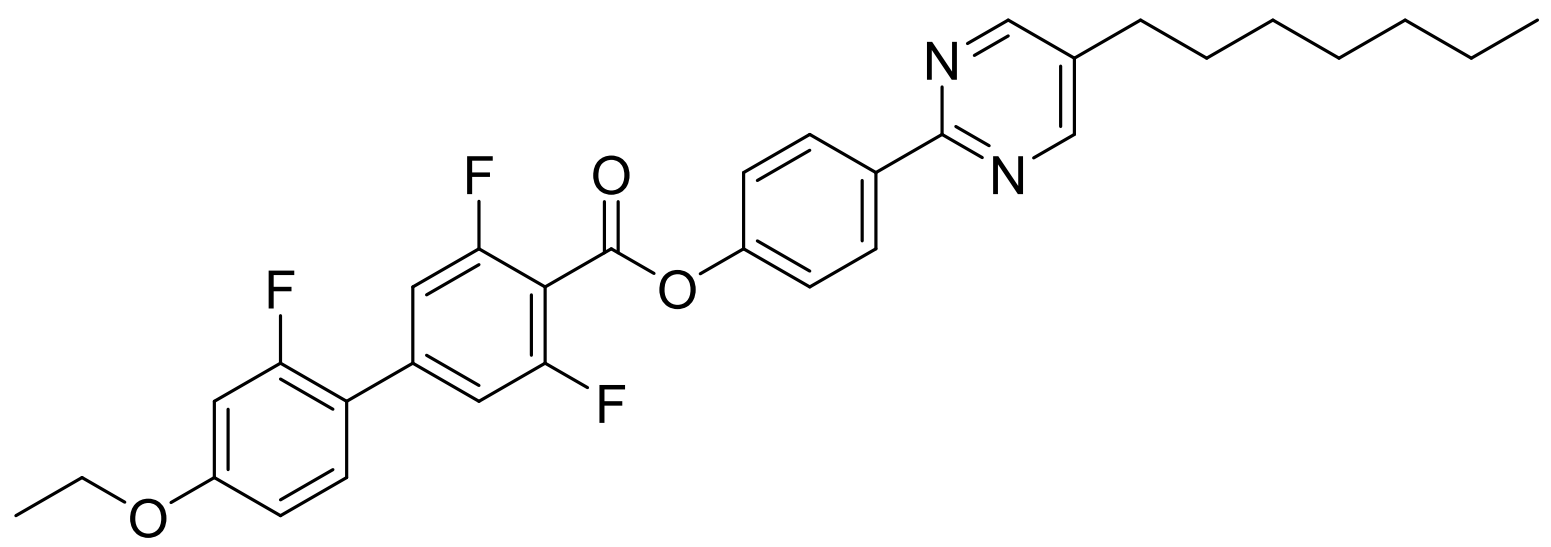

4-(5-heptylpyrimidin-2-yl)phenol (137 mg, 0.507 mmol), 4'-ethoxy-2',3,5-trifluoro-[1,1'-biphenyl]-4-carboxylic acid (277 mg, 0.935 mmol), EDC.HCl (289 mg, 1.508 mmol), DMAP (cat. amount). Standard Steglich esterification protocol was used. Yield: 180 mg, 0.3281 mmol, 64.75 %

Transition Temperatures (°C):	Cr 95.5 N 265.9 Iso
Transition Enthalpies (kJ/ mol):	Cr 30.5 N 2.9 Iso
^{1}H NMR (501 MHz):	8.63 (2 H, s), 8.51 (2 H, dd, *J* = 6.9, 1.7 Hz), 7.44 – 7.33 (3 H, m), 7.22 (2 H, d, *J* = 10.2 Hz), 6.79 (1 H, dd, *J* = 8.6, 2.5 Hz), 6.73 (1 H, dd, *J* = 12.8, 2.5 Hz), 4.08 (2 H, q, *J* = 7.0 Hz), 2.63 (2 H, t, *J* = 7.7 Hz), 1.66 (2 H, p, *J* = 7.5 Hz), 1.45 (3 H, t, *J* = 7.0 Hz), 1.41 – 1.23 (8 H, m), 0.89 (3 H, t, *J* = 6.9 Hz).
^{13}C{^{1}H} NMR (126 MHz):	161.89, 161.29 (d, *J* = 11.2 Hz), 159.78 (t, *J* = 2.0 Hz), 157.20, 152.36, 142.17 (t, *J* = 10.6 Hz), 135.88, 133.24, 130.70 (d, *J* = 4.5 Hz), 129.39, 121.81, 117.88 (d, *J* = 12.4 Hz), 112.38 (dd, *J* = 26.3, 4.1 Hz), 111.51 (d, *J* = 3.0 Hz), 108.36 (t, *J* = 17.0 Hz), 103.03, 102.82, 64.26, 31.87, 30.92, 30.34, 29.16, 22.76, 14.76, 14.21.
^{19}F NMR (376 MHz):	-109.14 (2 F, d, *J* = 10.3 Hz), -114.24 (1 F, t, *J* = 11.6 Hz).
HRMS:	549.2362 (- 0.3 ppm err) (M + H), expected 549.2360

18 | *3,5-difluoro-4-formylphenyl 4'-ethoxy-2',3,5-trifluoro-[1,1'-biphenyl]-4-carboxylate*

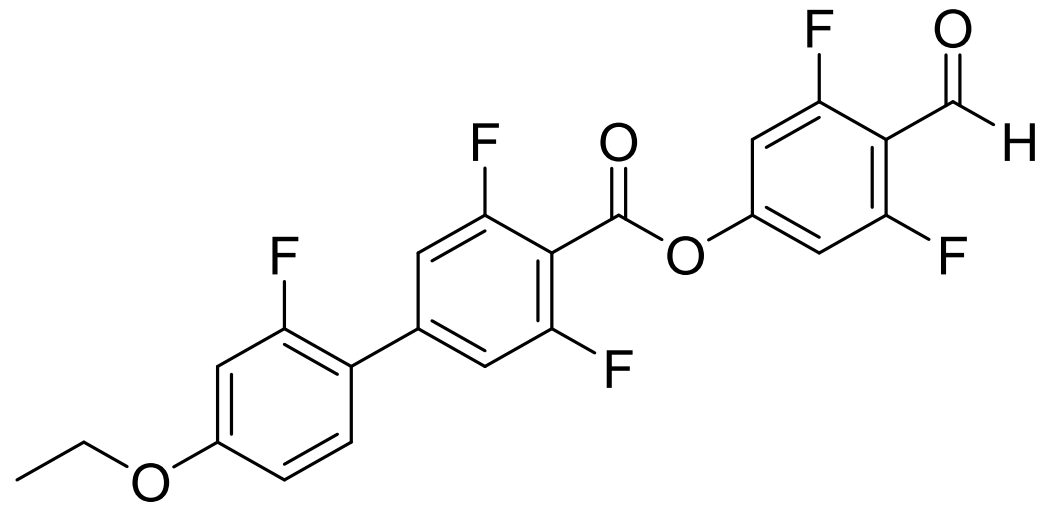

2,6-difluoro-4-hydroxybenzaldehyde (110 mg, 0.696 mmol), 4'-ethoxy-2',3,5-trifluoro-[1,1'-biphenyl]-4-carboxylic acid (273, 0.922 mmol), EDC.HCl (303 mg, 1.581 mmol), DMAP (cat.

amount). Standard Steglich esterification protocol was used. Yield: 134 mg, 0.3071 mmol, 44.14 %

Transition Temperatures (°C): Cr 125.2 (N 137.0) Iso

Transition Enthalpies (kJ/ mol): Cr 30.2 N 0.2 Iso

^{1}H NMR (501 MHz): 10.32 (1 H, s), 7.37 (1 H, t, *J* = 8.8 Hz), 7.24 (3 H, d, *J* = 10.7 Hz)*, 7.04 (2 H, d, *J* = 9.1 Hz), 6.81 (1 H, dd, *J* = 8.6, 2.5 Hz), 6.73 (1 H, dd, *J* = 13.0, 2.5 Hz), 4.08 (2 H, q, *J* = 7.0 Hz), 1.46 (3 H, t, *J* = 7.0 Hz).

*Solvent peak overlap

^{13}C{^{1}H} NMR (126 MHz): 183.49 (t, *J* = 4.1 Hz), 163.61 (dd, *J* = 263.6, 8.0 Hz), 161.50 (d, *J* = 5.9 Hz), 161.32 (dd, *J* = 259.1, 6.06 Hz), 160.37 (d, *J* = 233.21 Hz), 158.19 (t, *J* = 2.3 Hz), 155.57 (t, *J* = 14.9 Hz), 143.25 (t, *J* = 11.3 Hz), 130.54 (d, *J* = 4.5 Hz), 117.40 (d, *J* = 12.3 Hz), 112.66 – 112.14 (m)*, 111.51 (d, *J* = 2.9 Hz), 107.13 – 106.77 (m), 106.61 (t, *J* = 16.0 Hz), 102.85 (d, *J* = 26.1 Hz), 64.19, 14.62.

*Overlapping signals

^{19}F NMR (376 MHz): -108.15 (2 F, d, *J* = 10.8 Hz), -112.29 (2 F, d, *J* = 10.1 Hz), -114.07 (1 F, t, *J* = 10.9 Hz).

19 | *4'-(difluoro(3,4,5-trifluorophenoxy)methyl)-2,3',5'-trifluoro-[1,1'-biphenyl]-4-yl 4'-ethoxy-2',3,5-trifluoro-[1,1'-biphenyl]-4-carboxylate*

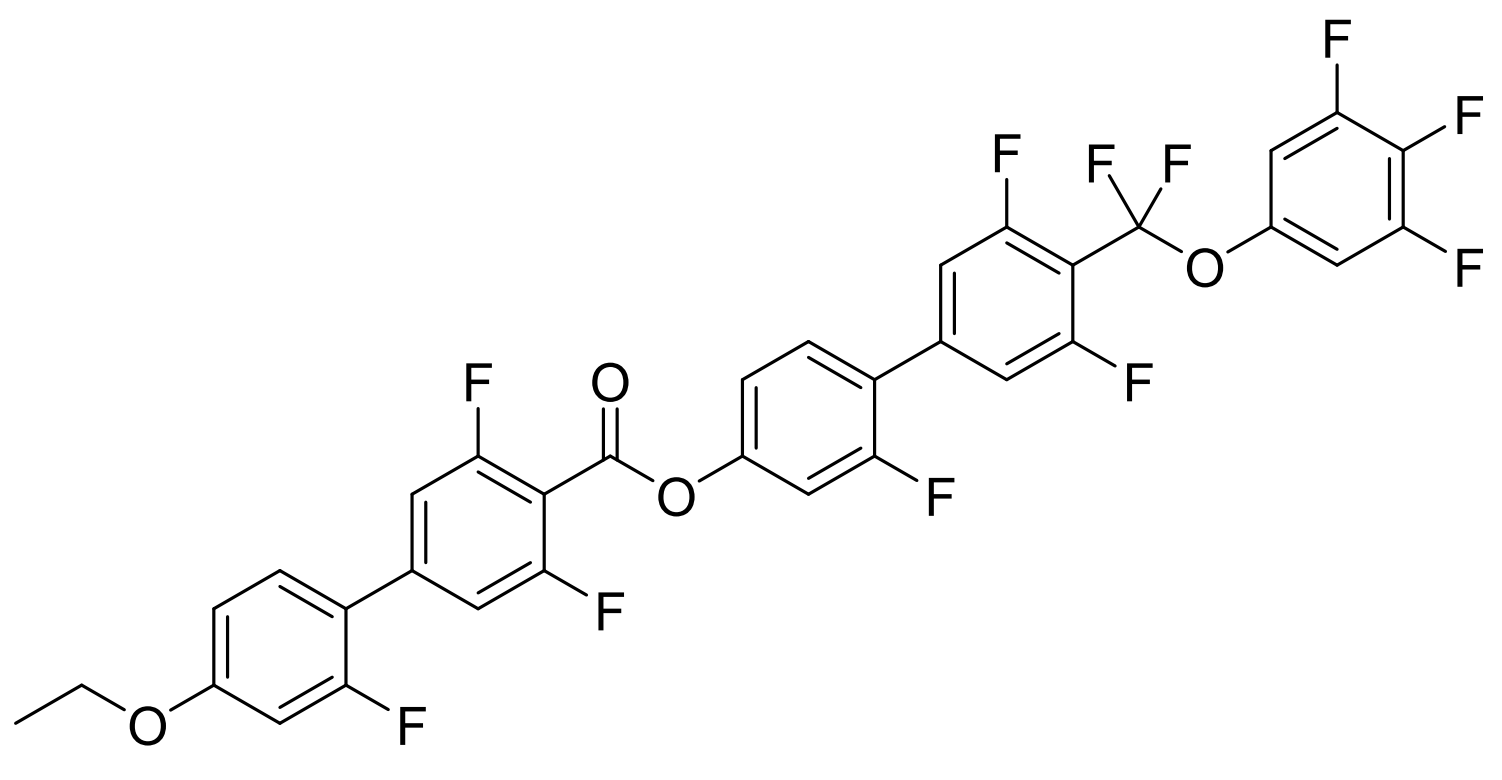

4'-(difluoro(3,4,5-trifluorophenoxy)methyl)-2,3',5'-trifluoro-[1,1'-biphenyl]-4-ol (189 mg, 0.450 mmol), 4'-ethoxy-2',3,5-trifluoro-[1,1'-biphenyl]-4-carboxylic acid (205 mg, 0.692 mmol), EDC.HCl (274 mg, 1.429 mmol), DMAP (cat. amount). Standard Steglich esterification protocol was used. Yield 64 %, 201 mg, 0.288 mmol.

Transition Temperatures (°C): Cr 152.7 (SmX 121.7) N 288.2 Iso

Transition Enthalpies (kJ/ mol): Cr 60.7 (SmX 6.7) N 2.1 Iso

^{1}H NMR (501 MHz):	7.51 (1 H, t, *J* = 8.5 Hz), 7.38 (1 H, t, *J* = 8.8 Hz), 7.27 – 7.19 (6 H, m), 7.05 – 6.95 (2 H, m), 6.80 (1 H, dd, *J* = 8.7, 2.6 Hz), 6.73 (1 H, dd, *J* = 12.8, 2.5 Hz), 4.09 (2 H, q, *J* = 7.0 Hz), 1.46 (3 H, t, *J* = 7.0 Hz).
^{13}C{^{1}H} NMR (126 MHz):	161.50, 161.33 (dd, *J* = 257.5, 6.52 Hz), 160.64 (d, 249.8 Hz), 160.4 (d, *J* = 250.5 Hz), 160.10 (dd, *J* = 257.5, 6.5 Hz), 159.67 (d, *J* = 252.3 Hz), 151.84 (d, *J* = 11.1 Hz), 151.16 (dq, *J* = 251.1, 5.6 Hz), 144.74 (t, *J* = 11.7 Hz), 141.95 (dt, *J* = 246.5, 10.9 Hz), 138.62 (dt, *J* = 250.6, 15.2 Hz), 130.72 (dd, *J* = 10.2, 4.1 Hz), 123.74 (d, *J* = 12.6 Hz), 122.39, 120.27, 118.52 (d, *J* = 3.7 Hz), 118.16, 117.70 (d, *J* = 12.6 Hz), 113.28 (dt, *J* = 23.7, 3.5 Hz), 112.50 (dt, *J* = 22.8, 4.0 Hz), 111.60 (d, *J* = 2.9 Hz), 111.00 (d, *J* = 25.9 Hz), 109.22 (tt, *J* = 32.5, 14.3 Hz), 107.84 – 107.44 (m), 107.42, 102.96 (d, *J* = 26.0 Hz), 64.31, 14.76.
^{19}F NMR (376 MHz):	-61.78 (2 F, t, *J* = 26.3 Hz), -108.73 (2 F, d, *J* = 10.5 Hz), -110.33 (2 F, td, *J* = 26.3, 10.9 Hz), -113.61 (1 F, t, *J* = 9.7 Hz), -114.18 (1 F, t, *J* = 10.6 Hz), -132.43 (2 F, dd, *J* = 20.8, 8.5 Hz), -163.10 (1 F, tt, *J* = 20.7, 6.0 Hz).

20 | *4'-(difluoro(3,4,5-trifluorophenoxy)methyl)-2,3',5'-trifluoro-[1,1'-biphenyl]-4-yl 3,5-difluoro-2',4'-dimethoxy-[1,1'-biphenyl]-4-carboxylate*

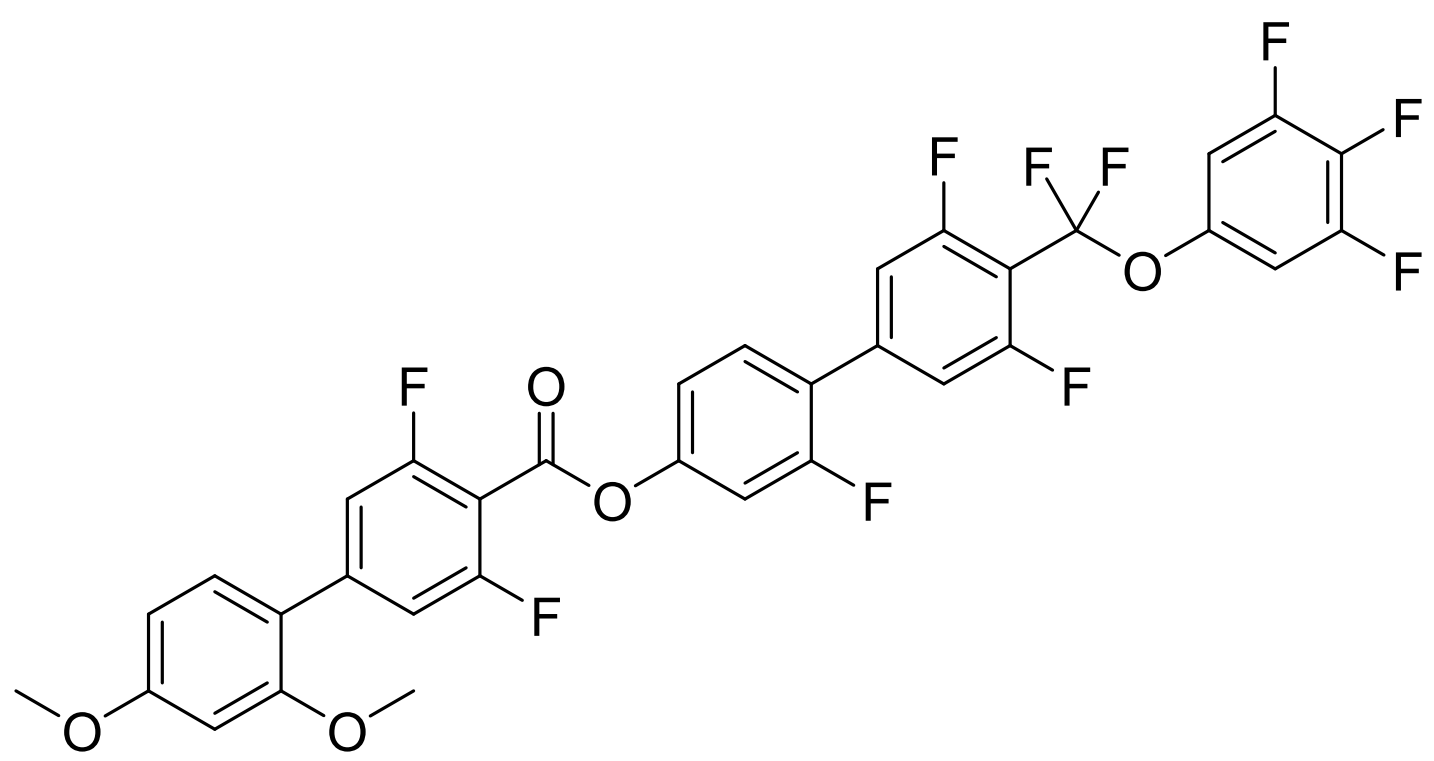

4'-(difluoro(3,4,5-trifluorophenoxy)methyl)-2,3',5'-trifluoro-[1,1'-biphenyl]-4-ol (313 mg, 0.745 mmol), 3,5-difluoro-2',4'-dimethoxy-[1,1'-biphenyl]-4-carboxylic acid (511 mg, 1.737 mmol), EDC.HCl (527 mg, 2.749 mmol), DMAP (cat. amount). Standard Steglich esterification protocol was used. Yield 71 %, 367 mg, 0.527 mmol

Transition Temperatures (°C):	Cr 108.2 (N_F 89.5) N 210.1 Iso
Transition Enthalpies (kJ/ mol):	Cr 52.5 (N_F 0.7) N 1.4 Iso
^{1}H NMR (501 MHz):	7.50 (1H, t, *J* = 8.6 Hz), 7.29 (1H, d, *J* = 8.4 Hz), 7.25 – 7.19 (6H, m),* 7.05 – 6.95 (2H, m), 6.60 (1H, dd, *J* = 8.5, 2.4 Hz), 6.57 (1H, d, *J* = 2.3 Hz), 3.88 (3H, s), 3.86 (3H, s)

*Solvent Overlap, overlapping signals

$^{13}C\{^{1}H\}$ NMR (126 MHz):	162.01, 159.67, 161.1 (dd, *J* = 255.6, 6.2 Hz), 160.1 (dd, *J* = 255.9, 6.33 Hz), 159.7 (d, *J* = 252.5 Hz), 157.79, 151.94 (d, *J* = 11.1 Hz), 151.2 (ddd, *J* = 251.5, 10.7, 5.2 Hz), 145.69 (t, *J* = 11.1 Hz), 144.75 (t, *J* = 13.5 Hz), 140.88 (t, *J* = 11.1 Hz), 138.62 (dt, *J* = 250.9, 15.1 Hz), 131.25, 130.73 (d, *J* = 3.8 Hz), 123.64 (d, *J* = 12.7 Hz), 122.40, 120.28, 119.92, 118.56 (d, *J* = 3.8 Hz), 113.73 – 113.00 (m),* 111.03 (d, *J* = 25.8 Hz), 107.62 (dd, *J* = 18.1, 6.8 Hz), 106.74 (t, *J* = 16.4 Hz), 105.31, 99.26, 55.76, 55.68

* Overlapping signals

^{19}F NMR (376 MHz):	-61.78 (2 F, t, *J* = 26.3 Hz), -109.85 (2 F, d, *J* = 10.9 Hz), -110.36 (2 F, td, *J* = 26.4, 11.0 Hz), -113.71 (1 F, t, *J* = 9.7 Hz), -132.43 (2 F, dd, *J* = 21.0, 8.6 Hz), -163.11 (1 F, t, *J* = 19.9 Hz)

21 | *4-(ethoxycarbonyl)phenyl 2,3-dihydrobenzo[b][1,4]dioxine-6-carboxylate*

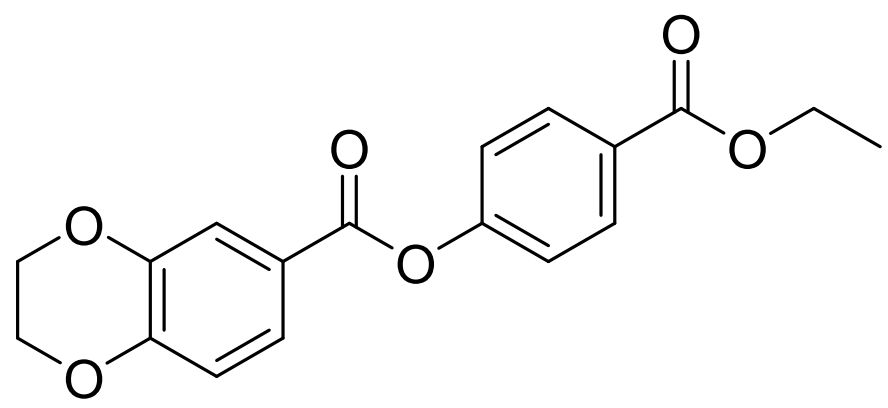

ethyl 4-hydroxybenzoate (179 mg, 1.077 mmol), 2,3-dihydrobenzo[b][1,4]dioxine-6-carboxylic acid (246 mg, 1.365 mmol), EDC.HCl (306 mg, 1.596 mmol), DMAP (cat. amount). Standard Steglich esterification protocol was used. Yield: 59 %, 208 mg, 0.634 mmol

Transition Temperatures (°C):	Cr 99.8 Iso
Transition Enthalpies (kJ/ mol):	Cr 27.1 Iso
^{1}H NMR (400 MHz):	8.11 (2 H, dd, *J* = 6.8, 2.1 Hz), 7.75 – 7.68 (2 H, m), 7.28 (2 H, dd, *J* = 6.6, 2.0 Hz), 6.96 (1 H, d, *J* = 9.0 Hz), 4.39 (2 H, q, *J* = 7.2 Hz), 4.32 (4 H, m), 1.40 (3 H, t, *J* = 7.1 Hz).
$^{13}C\{^{1}H\}$ NMR (101 MHz):	166.05, 164.25, 154.79, 148.76, 143.51, 131.26, 128.10, 124.36, 122.33, 121.85, 119.81, 117.57, 64.86, 64.22, 61.20, 14.47.

22 | *4-cyanophenyl (E)-3-(2,4-dimethoxyphenyl)acrylate*

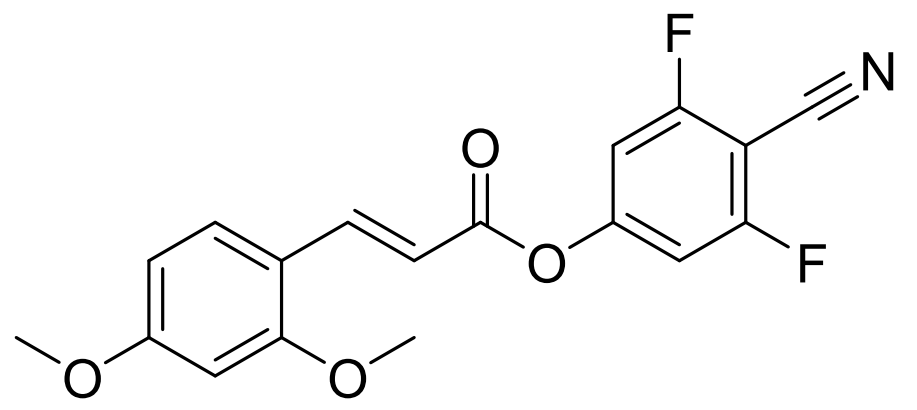

2,6-difluoro-4-hydroxybenzonitrile (95 mg, 0.613 mmol), (E)-3-(2,4-dimethoxyphenyl)acrylic acid (216 mg, 1.037 mmol), EDC.HCl (321 mg, 1.674 mmol), DMAP (cat. amount). Standard Steglich esterification protocol was used. Yield: 106 mg, 0.3070 mmol, 50.12 %

Transition Temperatures (°C):	Cr 164.8 Iso
Transition Enthalpies (kJ/ mol):	Cr 38.6 Iso
^{1}H NMR (501 MHz):	8.09 (1 H, d, *J* = 16.0 Hz), 7.49 (1 H, d, *J* = 8.7 Hz), 7.00 (2 H, dd, *J* = 10.5, 2.8 Hz), 6.57 (1 H, d, *J* = 16.0 Hz)*, 6.55 (1 H, dd, *J* = 8.6, 2.4)*, 6.48 (1 H, d, *J* = 2.3 Hz), 3.91 (3 H, s), 3.87 (3 H, s).<br><br>*Overlapping signals
^{13}C{^{1}H} NMR (126 MHz):	164.61, 163.95, 163.74 (dd, *J* = 260.3, 6.3 Hz) 160.71, 156.58 (t, *J* = 13.7 Hz), 144.68, 131.70, 115.99, 112.94, 109.11, 106.89 (dd, *J* = 22.9, 3.8 Hz), 105.79, 98.62, 89.54 (t, *J* = 19.5 Hz), 55.73.
^{19}F NMR (376 MHz):	-102.57 (2 F, d, *J* = 8.9 Hz).
HRMS:	346.0890 (- 1.2 ppm err) (M + H), expected 246.0886

23 | *4'-cyano-2,3',6-trifluoro-[1,1'-biphenyl]-4-yl 2-methoxy-4-(trifluoromethoxy)benzoate*

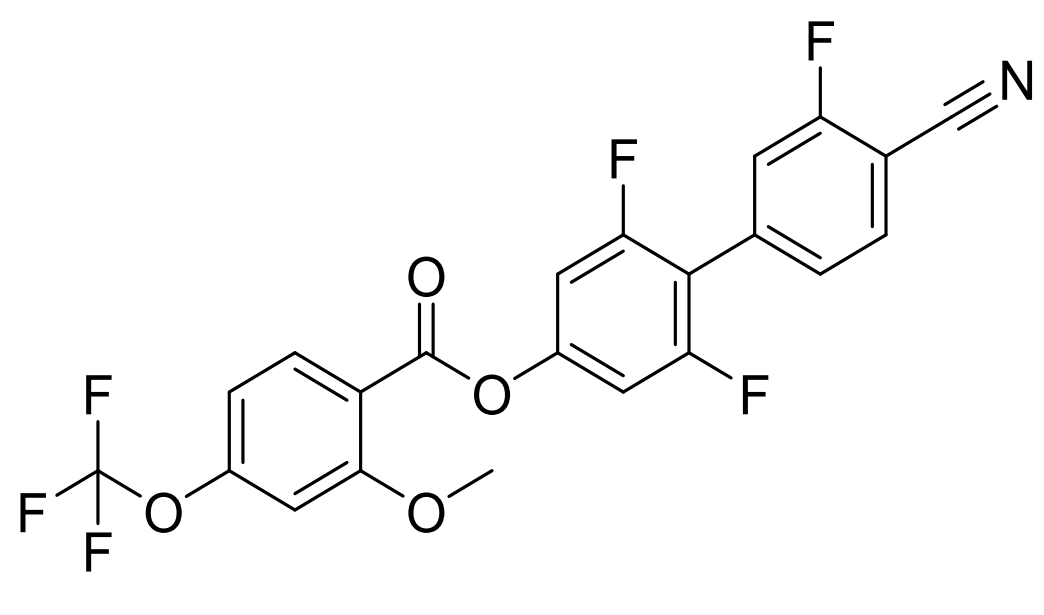

2',3,6'-trifluoro-4'-hydroxy-[1,1'-biphenyl]-4-carbonitrile (199 mg, 0.799 mmol), 2-methoxy-4-(trifluoromethoxy)benzoic acid (266 mg, 1.126 mmol), EDC.HCl (319 mg, 1.664 mmol), DMAP (cat. amount). Standard Steglich esterification protocol was used. Yield: 169 mg, 0.3616 mmol, 45.28 %

Transition Temperatures (°C):	Cr 126.0 Iso

Transition Enthalpies (kJ/ mol):	Cr 32.6 Iso
^{1}H NMR (501 MHz):	8.07 (1 H, d, *J* = 8.7 Hz), 7.72 (1 H, dd, *J* = 8.0, 6.7 Hz), 7.39 (2 H, td, *J* = 9.8, 1.2 Hz), 7.04 – 6.96 (2 H, m), 6.93 (1 H, ddt, *J* = 8.7, 2.5, 1.2 Hz), 6.87 (1 H, d, *J* = 2.2 Hz), 3.97 (3 H, s).
^{13}C{^{1}H} NMR (126 MHz):	162.75 (d, *J* = 259.6 Hz), 162.29, 161.80, 161.23 (dd, *J* = 257.5, 7.4 Hz), 160 (d, *J* = 251.0 Hz) 159.81 (dd, *J* = 251.2, 8.6 Hz), 154.32 (d, *J* = 1.8 Hz), 152.11 (t, *J* = 14.4 Hz), 136.15 (d, *J* = 8.8 Hz), 134.28, 133.37, 127.01 (d, *J* = 2.5 Hz), 120.90 (q, *J* = 257.8 Hz) 118.63 (dt, *J* = 21.0, 1.9 Hz), 116.11, 113.86, 113.53 (td, *J* = 17.9, 1.5 Hz), 111.74 (d, *J* = 1.1 Hz), 107.40 – 106.58 (m), 105.01, 101.38 (d, *J* = 15.4 Hz), 56.57.
^{19}F (376 MHz):	-57.45 (3 F, s), -106.15 (1 F, t, *J* = 8.2 Hz), -112.31 (2 F, d, *J* = 9.2 Hz).
HRMS :	468.0671 – 1.30 ppm (M + H), expected 468.0666

24 | *4-bromo-3-methoxyphenyl 2',3,5-trifluoro-4'-methoxy-[1,1'-biphenyl]-4-carboxylate*

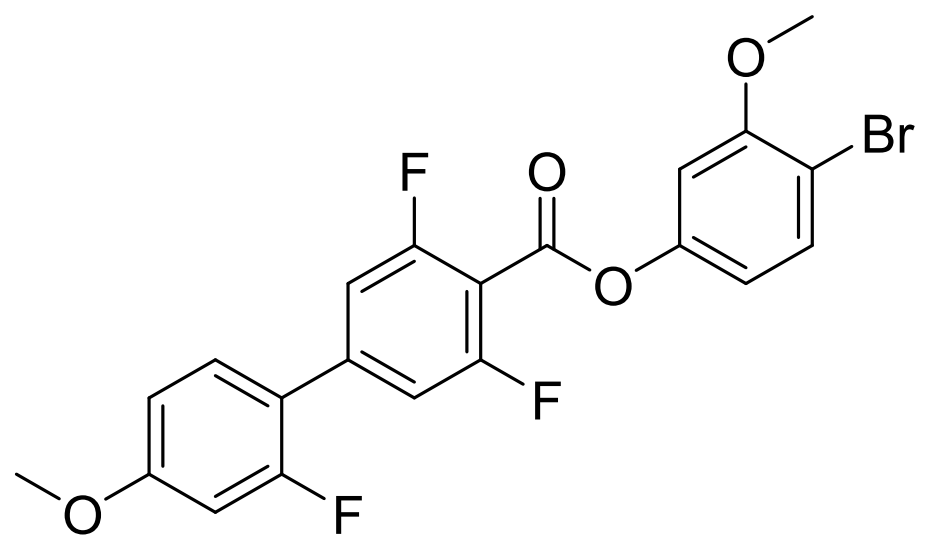

4-bromo-3-methoxyphenol (194 mg, 0.955 mmol), 2',3,5-trifluoro-4'-methoxy-[1,1'-biphenyl]-4-carboxylic acid (360 mg, 1.215 mmol), EDC.HCl (491 mg, 2.561 mmol), DMAP (cat. amount). Standard Steglich esterification protocol was used. Yield 72 %, 322 mg, 0.689 mmol

Transition Temperatures (°C):	Cr 124.6 Iso
Transition Enthalpies (kJ/ mol):	Cr 71.8 Iso
^{1}H NMR (501 MHz):	7.58 (1 H, d, *J* = 8.5 Hz), 7.38 (1 H, t, *J* = 8.8 Hz), 7.22 (2 H, dd, *J* = 10.9, 1.5 Hz), 6.85 (1 H, d, *J* = 2.5 Hz), 6.81 (2 H, ddd, *J* = 8.5, 6.0, 2.4 Hz), 6.74 (1 H, dd, *J* = 12.8, 2.5 Hz), 3.92 (3 H, s), 3.86 (3 H, s).
^{13}C{^{1}H} NMR (126 MHz):	161.98 (d, *J* 11.3), 161.22 (dd, *J* = 258.2 Hz), 160.61 (d, 250.5 Hz), 159.67 (t, *J* = 2.1), 156.70, 150.64, 142.32 (td, *J* 11.0, 1.5), 133.54, 130.74 (d, *J* 4.5), 117.99 (dt, *J* 12.4, 2.3), 114.91, 113.05 – 112.09 (m), 111.09 (d, *J*

3.0), 108.93, 108.08 (t, *J* 16.8), 106.30, 102.54 (d, *J* 26.2), 56.58, 55.92.

^{19}F NMR (376 MHz): -109.00 (2 F, d, *J* = 10.5 Hz), -114.14 (1 F, t, *J* = 10.1 Hz).

25 | *4-isothiocyanatophenyl 4-bromo-2,6-difluorobenzoate*

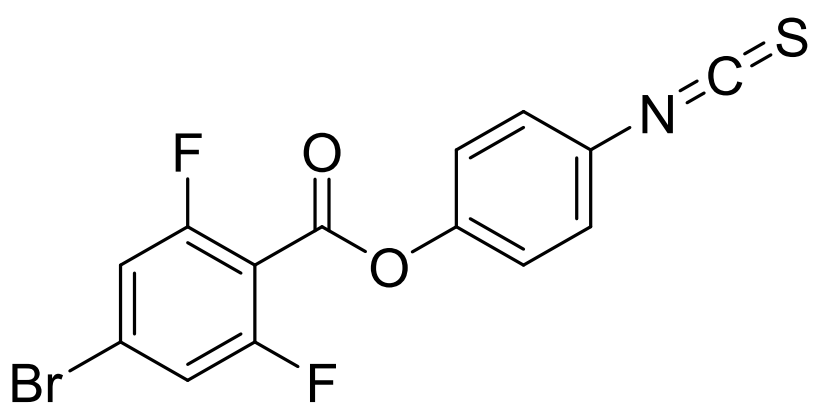

4-isothiocyanatophenol (106 mg, 0.701 mmol), 4-bromo-2,6-difluorobenzoic acid (234 mg, 0.987 mmol), EDC.HCl (399 mg, 2.081 mmol), DMAP (cat. amount). Standard Steglich esterification protocol was used. Yield: 161 mg, 0.4349 mmol, 62.03 %

Transition Temperatures (°C): Cr 123.6 Iso

Transition Enthalpies (kJ/ mol): Cr 33.3 Iso

^{1}H NMR (501 MHz, DMSO): 7.77 (2 H, d, *J* = 8.1 Hz), 7.56 (2 H, dd, *J* = 6.7, 2.2 Hz), 7.39 (2 H, dd, *J* = 6.7, 2.2 Hz).

^{13}C{^{1}H} NMR (126 MHz): 161.05 (dd, *J* = 262.6, 6.6 Hz), 158.97 (t, *J* = 2.0 Hz), 148.81, 136.56, 129.66, 127.07 (t, J = 12.3 Hz)*, 12.02*, 122.96, 116.63 (dd, *J* = 25.5, 3.3 Hz), 109.24 (t, *J* = 16.8 Hz).

*Overlapping signals

^{19}F NMR (376 MHz): -107.06 (2 F, d, *J* = 8.5 Hz).

26 | *4-((1r,4s)-4-propylcyclohexyl)phenyl 4-(benzyloxy)benzoate*

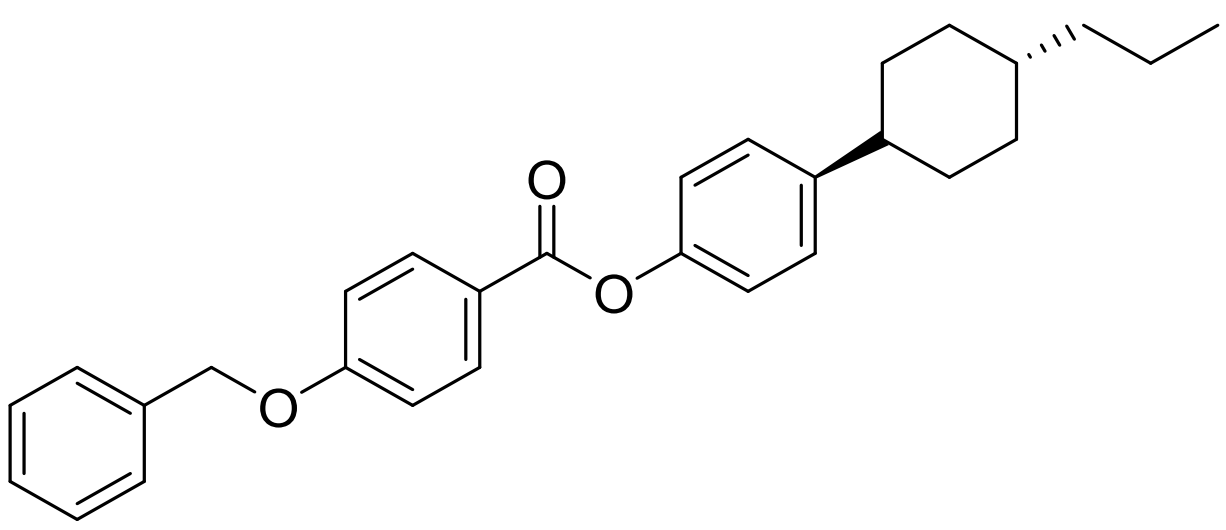

4-((1s,4r)-4-propylcyclohexyl)phenol (115 mg, 0.527 mmol), 4-(benzyloxy)benzoic acid (255 mg, 1.117 mmol), EDC.HCl (399 mg, 2.081 mmol), DMAP (cat. amount). Standard Steglich esterification protocol was used. Yield: 223 mg, 0.5203 mmol, 98.79 %

Transition Temperatures (°C): Cr 106.4 Iso

Transition Enthalpies (kJ/ mol):	Cr 30.7 Iso
^{1}H NMR (501 MHz, DMSO):	7.71 (1 H, dt, *J* = 7.7, 1.2 Hz), 7.69 – 7.67 (1 H, m), 7.52 (1 H, t, *J* = 8.0 Hz), 7.48 (2 H, d, *J* = 7.0 Hz), 7.40 (3 H, t, *J* = 7.9 Hz), 7.34 (1 H, tt, *J* = 7.3, 1.8 Hz), 7.30 (2 H, d, *J* = 8.6 Hz), 7.16 (2 H, dd, *J* = 6.6, 2.0 Hz), 5.21 (2 H, s), 2.54 – 2.46 (1 H, m)*, 1.82 (4 H, d, *J* = 11.6 Hz), 1.45 (2 H, qd, *J* = 12.6, 2.8 Hz), 1.38 – 1.27 (3 H, m), 1.24 – 1.15 (2 H, m), 1.03 (2 H, qd, *J* = 12.4, 2.5 Hz), 0.88 (3 H, t, *J* = 7.3 Hz).<br><br>*Solvent Overlap
^{13}C{^{1}H} NMR (101 MHz):	165.30, 158.96, 148.99, 145.65, 136.62, 131.18, 129.76, 128.80, 128.29, 127.93, 127.73, 122.96, 121.40, 120.89, 115.78, 70.39, 44.25, 39.85, 37.15, 34.56, 33.69, 20.18, 14.56.
HRMS:	429.2420 (+ 1.0 ppm err) (M + H), 451.2241 (+ 0.7 ppm err) (M + Na), expected 429.2425, 451.224

27 | *bis(2,3',4',5'-tetrafluoro-[1,1'-biphenyl]-4-yl) nonanedioate*

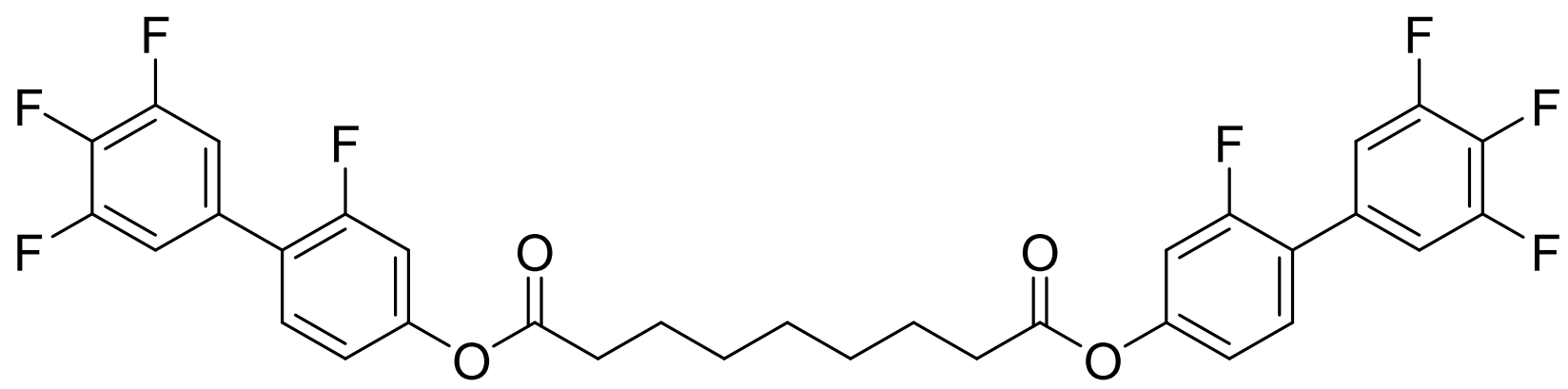

2,3',4',5'-tetrafluoro-[1,1'-biphenyl]-4-ol (885 mg, 3.654 mmol), heptanedioic acid (199 mg, 1.057 mmol), EDC.HCl (730 mg, 3.801 mmol), DMAP (cat. amount). Standard Steglich esterification protocol was used. Yield: 532 mg, 0.8358 mmol %, 79.05 %

Transition Temperatures (°C):	Cr 104.6 Iso
Transition Enthalpies (kJ/ mol):	Cr 49.1 Iso
^{1}H NMR (501 MHz, acetone):	7.65 (2 H, dd, *J* = 11.5, 2.2 Hz), 7.61 – 7.54 (6 H, m), 7.36 (2 H, t, *J* = 8.2 Hz), 2.69 (4 H, t, *J* = 7.4 Hz), 1.79 (4 H, p, *J* = 7.4 Hz), 1.56 – 1.42 (6 H, m).
^{13}C{^{1}H} NMR (126 MHz):	171.22, 154.42 (d, *J* = 250.4 Hz), 151.62 (ddd, *J* = 250.4, 10.1, 4.3 Hz), 139.76 (dt, *J* = 253.1, 15.4 Hz), 138.43 (d, *J* = 13.1 Hz), 137.61 (dd, *J* = 7.1, 1.9 Hz), 135.54 (dtd, *J* = 7.9, 5.2, 2.5 Hz), 124.57 (d, *J* = 1.6 Hz), 122.99 (d, *J* = 3.4 Hz), 115.42 (d, *J* = 20.0 Hz), 111.28 (dd, *J* = 16.5, 5.3 Hz), 33.96, 28.94, 28.89, 24.90.

^{19}F NMR (376 MHz):	-126.73 (1 F, t, *J* = 9.0 Hz), -133.40 (2 F, dd, *J* = 20.5, 8.6 Hz), -161.37 (1 F, tt, *J* = 20.7, 6.5 Hz).
HRMS:	659.1435 (+ 0.1 pm err) (M + Na), expected 659.1440

28 | *bis(4-((1s,4r)-4-propylcyclohexyl)phenyl) nonanedioate*

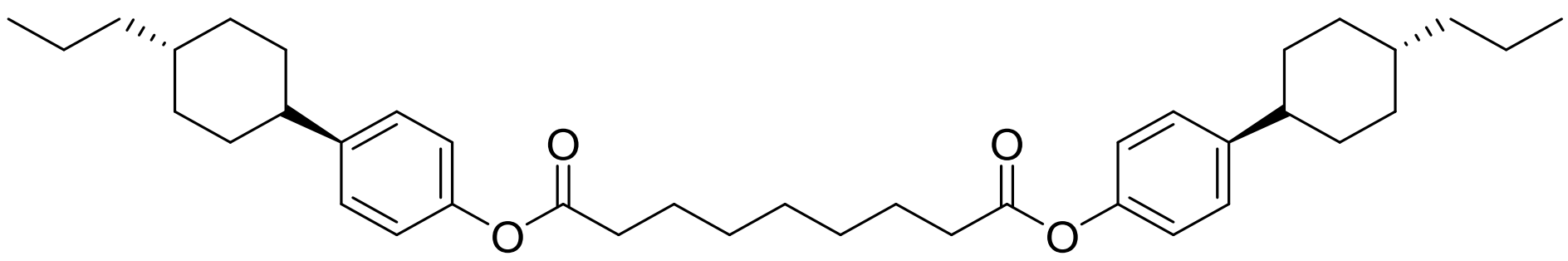

4-((1s,4r)-4-propylcyclohexyl)phenol (660 mg, 3.023 mmol), heptanedioic acid (200 mg, 1.062 mmol), EDC.HCl (625 mg, 3.260 mmol), DMAP (cat. amount). Standard Steglich esterification protocol was used. Yield: 452 mg, 0.7676 mmol, 72.24 %

Transition Temperatures (°C):	Cr (Cr 86.0 Cr 99.1) N 111.8 Iso
Transition Enthalpies (kJ/ mol):	Cr 36.0 (12.3 Cr 23.1) N 2.2 Iso
^{1}H NMR (501 MHz):	7.19 (4 H, dd, *J* = 6.5, 2.1 Hz), 6.98 (4 H, dd, *J* = 6.6, 2.0 Hz), 2.55 (4 H, t, *J* = 7.5 Hz), 2.46 (2 H, tt, *J* = 12.2, 3.3 Hz), 1.87 (8 H, t, *J* = 12.4 Hz), 1.76 (4 H, p, *J* = 7.5 Hz), 1.48 – 1.24 (16 H, m), 1.24 – 1.17 (4 H, m), 1.04 (4 H, qd, *J* = 13.8, 4.4 Hz), 0.90 (6 H, t, *J* = 7.3 Hz).
^{13}C{^{1}H} NMR (126 MHz):	172.58, 148.78, 145.43, 127.83, 121.29, 44.20, 39.84, 37.13, 34.51 (d, *J* = 4.5 Hz), 33.67, 29.02, 25.03, 20.17, 14.55
HRMS:	589.4250 (+ 0.2 ppm err) (M + H), 611.4068 (+ 0.4 ppm err) (M + Na), expected 589.4252, 611.4071

3.4 Novel biphenyl carboxylic acid intermediates

CA1 | *2,6-difluoro-4-(4-pentylphenyl)benzoic acid*

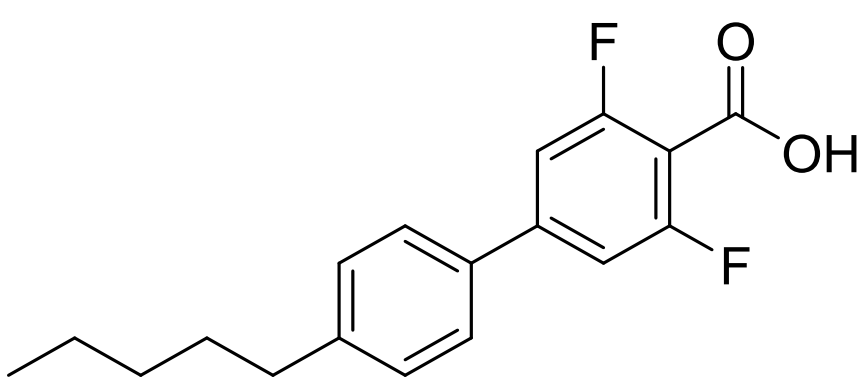

THF (100 ml), potassium carbonate 2 M aqueous (50 ml), *4-bromo-2,6-difluorobenzoic acid* (3.550 g, 14.98 mmol), *(4-pentylphenyl)boronic* acid (2.660 g, 13.85 mmol) and PdXPhos G3

(cat. amount). The general Suzuki-Miyaura protocol afforded the title compound with a yield of 61.47 %, 2.591 g, 8.514 mmol.

^{1}H NMR (501 MHz, DMSO): 13.84 (1 H, s), 7.70 (2 H, d, *J* = 8.2 Hz), 7.54 (2 H, d, *J* = 10.1 Hz), 7.31 (2 H, d, *J* = 8.0 Hz), 2.61 (2 H, t, *J* = 7.7 Hz), 1.59 (2 H, p, *J* = 7.4 Hz), 1.37 – 1.22 (4 H, m), 0.86 (3 H, t, *J* = 6.9 Hz).

^{13}C{^{1}H} NMR (126 MHz, DMSO): 162.14, 160.91 (d, *J* = 7.9 Hz), 158.91 (d, *J* = 7.9 Hz), 144.93 (t, *J* = 10.3 Hz), 143.81, 134.10 (t, *J* = 2.3 Hz), 129.05, 126.89, 110.07 – 109.71 (m), 34.72, 30.86, 30.47, 21.95, 13.91.

^{19}F NMR (376 MHz, DMSO): -111.34 (2 F, d, *J* = 10.3 Hz).

CA2 | *4-(4-Ethoxy-2-fluorophenyl)-2,6-difluorobenzoic acid*

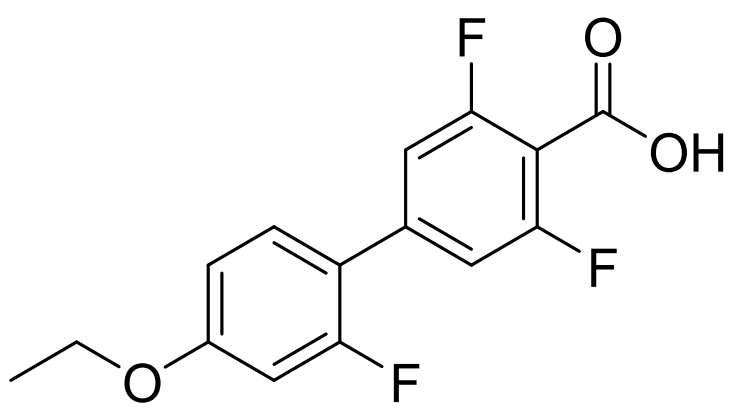

THF (ca. 100 ml), potassium carbonate 2 M aqueous (ca. 50 ml), *4-bromo-2,6-difluorobenzoic acid* (7.787 g, 32.85 mmol), *(4-ethoxy-2,fluorophenyl)boronic acid* (5.045 g, 27.42 mmol) and PdXPhos G3 (cat. amount). The general Suzuki-Miyaura protocol afforded the title compound with a yield of (77.90 %, 6.362 g, 21.36 mmol).

^{1}H NMR (501 MHz, DMSO): 13.92 (1 H, s), 7.58 (1 H, t, *J* = 9.0 Hz), 7.36 (2 H, d, *J* = 9.1 Hz), 6.97 (1 H, dd, *J* = 13.3, 2.5 Hz), 6.89 (1 H, dd, *J* = 8.6, 2.5 Hz), 4.10 (2 H, q, *J* = 7.0 Hz), 1.34 (3 H, t, *J* = 7.0 Hz).

^{13}C{^{1}H} NMR (126 MHz, DMSO): 162.01, 160.63 (d, *J* = 11.5 Hz), 159.83 (d, *J* = 247.8 Hz), 159.4 (dd, *J* = 251.9, 7.81 Hz) 139.78 (t, *J* = 10.2 Hz), 131.25 (d, *J* = 4.4 Hz), 117.09 (d, *J* = 12.6 Hz), 112.08 (dt, *J* = 21.3, 4.2 Hz), 111.62 (d, *J* = 2.8 Hz), 102.59 (d, *J* = 26.1 Hz), 63.94, 14.44.

^{19}F NMR (376 MHz, DMSO): -111.83 (2 F, d, *J* = 10.1 Hz), -114.90 (1 F, t, *J* = 11.5 Hz).

CA3 | *4-(2,4-Dimethoxyphenyl)-2,6-difluorobenzoic acid*

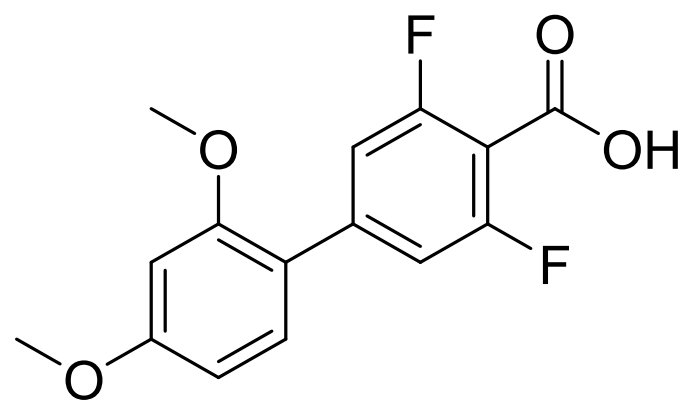

THF (ca. 100 ml), potassium carbonate 2 M aqueous (50 ml), *4-bromo-2,6-difluorobenzoic acid* (3.644 g, 15.38 mmol), *(2,4-methoxyphenyl)boronic acid* (2.420 g, 13.30 mmol) and PdXPhos G3 (cat. amount). The general Suzuki-Miyaura protocol afforded the title compound with a yield of (62.44 %, 2.442 g, 8.302 mmol).

^{1}H NMR (501 MHz, DMSO): 13.78 (1 H, s), 7.37 (1 H, d, *J* = 8.5 Hz), 7.29 (2 H, d, *J* = 9.7 Hz), 6.69 (1 H, d, *J* = 2.4 Hz), 6.64 (1 H, dd, *J* = 8.5, 2.4 Hz), 3.81 (6 H, d, *J* = 3.2 Hz).

^{13}C{^{1}H} NMR (126 MHz, DMSO): 162.20, 161.33, 159.07 (dd, *J* = 250.6, 8.1 Hz), 157.34, 142.96 (t, *J* = 10.8 Hz), 131.16, 118.92 (t, *J* = 2.2 Hz), 112.44 (dd, *J* = 20.7, 4.6 Hz), 109.40 (t, *J* = 19.9 Hz), 105.73, 98.99, 55.74, 55.43.

^{19}F NMR (376 MHz, DMSO): -112.78 (2 F, d, *J* = 10.5 Hz).

CA4 | *4-(4-Methoxy-2-fluorophenyl)-2,6-difluorobenzoic acid*

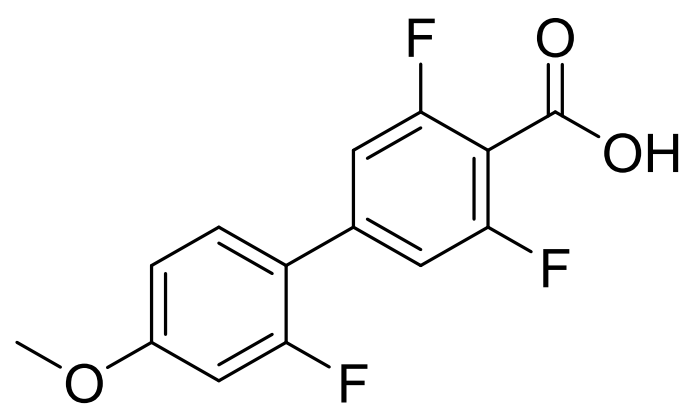

THF (120 ml), potassium carbonate 2 M aqueous (40 ml), *4-bromo-2,6-difluorobenzoic acid* (3.508 g, 14.80 mmol), *(2-fluoro-4-methoxyphenyl)boronic acid* (2.289 g, 13.47 mmol), and PdXPhos G3 (cat. amount). The general Suzuki-Miyaura protocol afforded the title compound with a yield of (71.10 %, 2.703 g, 9.577 mmol).

^{1}H NMR (501 MHz, DMSO): 13.92 (1 H, s), 7.59 (1 H, t, *J* = 9.0 Hz), 7.38 (2 H, dd, *J* = 9.5, 1.2 Hz), 7.00 (1 H, dd, *J* = 13.3, 2.5 Hz), 6.92 (1 H, dd, *J* = 8.7, 2.6 Hz), 3.83 (3 H, s).

^{13}C{^{1}H} NMR (126 MHz, DMSO): 162.01, 161.39 (d, *J* = 11.5 Hz), 159.89 (d, *J* = 247.7 Hz), 159.85 (dd, *J* = 251.6, 7.7 Hz), 139.79 (t, *J* = 11.0 Hz), 131.27 (d, *J* = 4.5 Hz), 117.23 (d, *J* = 12.3 Hz), 112.47 – 111.87 (m), 111.26 (d, *J* = 2.8 Hz), 110.39 (t, *J* = 19.8 Hz), 102.23 (d, *J* = 26.3 Hz), 55.91.

^{19}F NMR (376 MHz, DMSO): -111.82 (2 F, d, *J* = 10.1 Hz), -114.88 (1 F, t, *J* = 11.1 Hz).

4 Supplemental References

1 D. Prat, A. Wells, J. Hayler, H. Sneddon, C. R. McElroy, S. Abou-Shehada and P. J. Dunn, *Green Chemistry*, 2015, **18**, 288–296.

2 R. K. Henderson, C. Jiménez-González, D. J. C. Constable, S. R. Alston, G. G. A. Inglis, G. Fisher, J. Sherwood, S. P. Binks and A. D. Curzons, *Green Chemistry*, 2011, **13**, 854–862.

5 Compound Spectra, POM Images and DSC Thermograms

CZP-5-N | *4-cyanophenyl (1s,4r)-4-pentylcyclohexane-1-carboxylate*

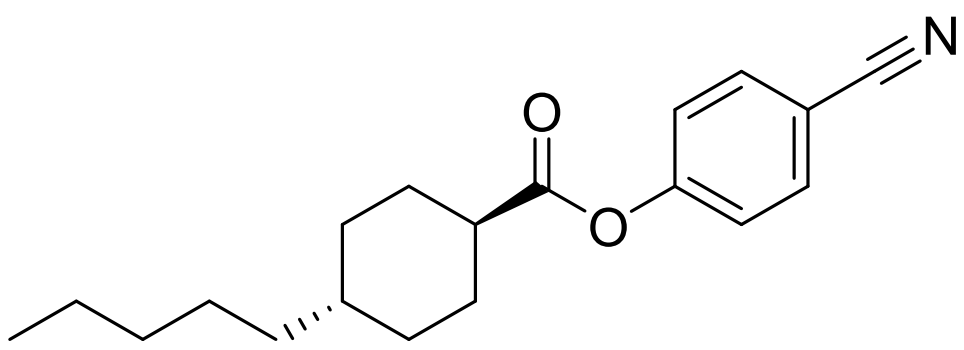

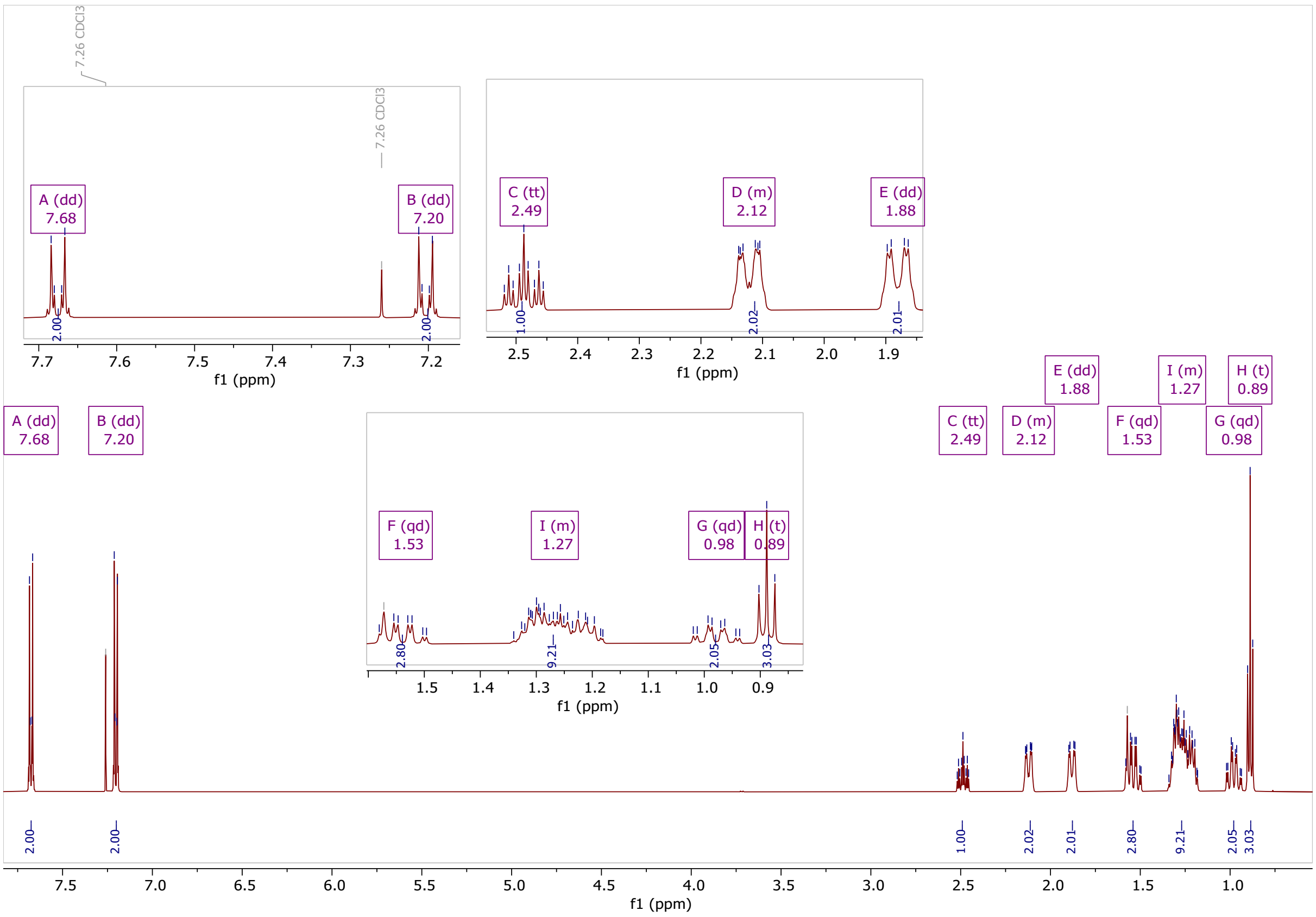

Figure S4 proton NMR spectrum of **CZP-5-N**

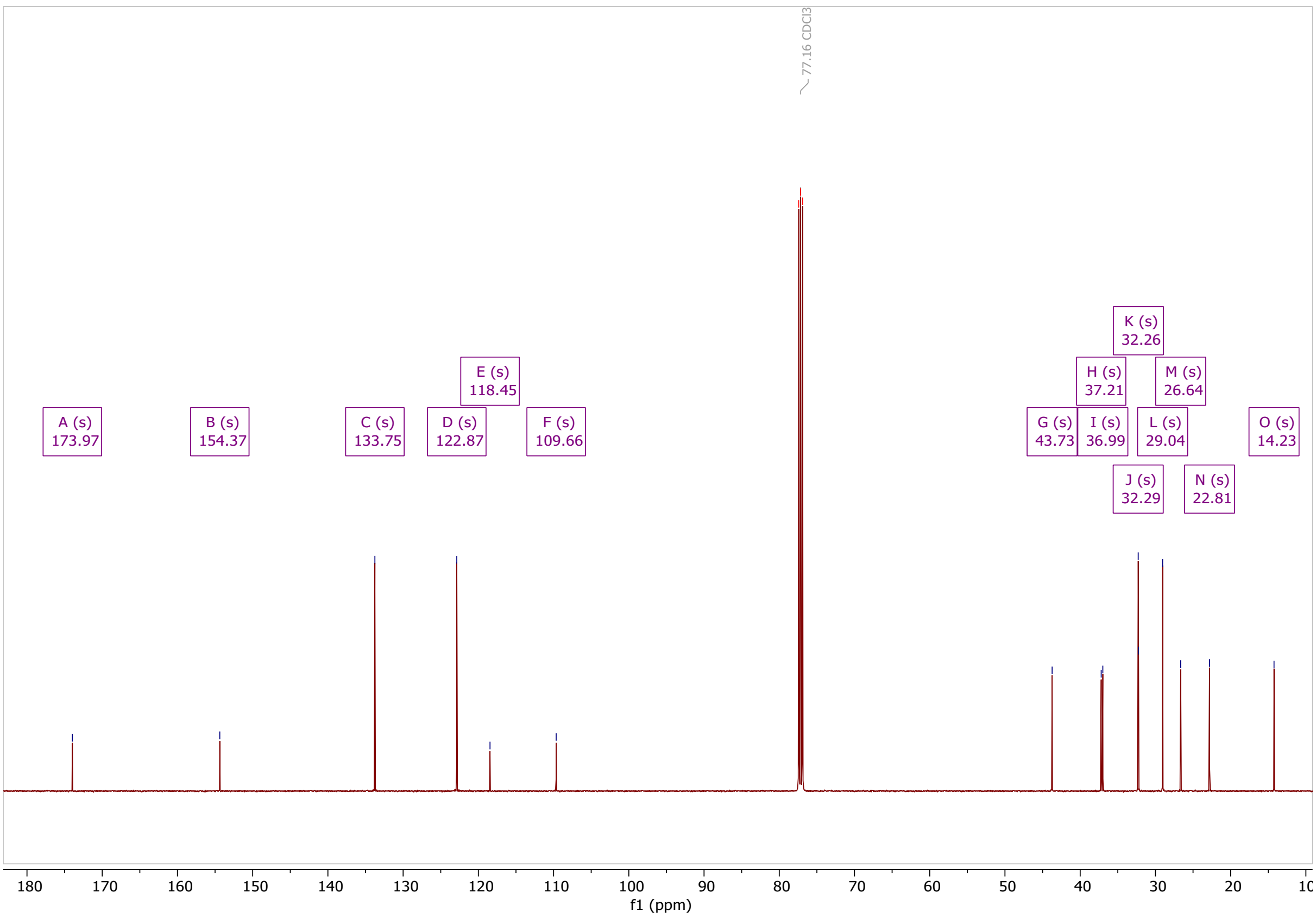

Figure S5 carbon NMR Spectrum of **CZP-5-N**

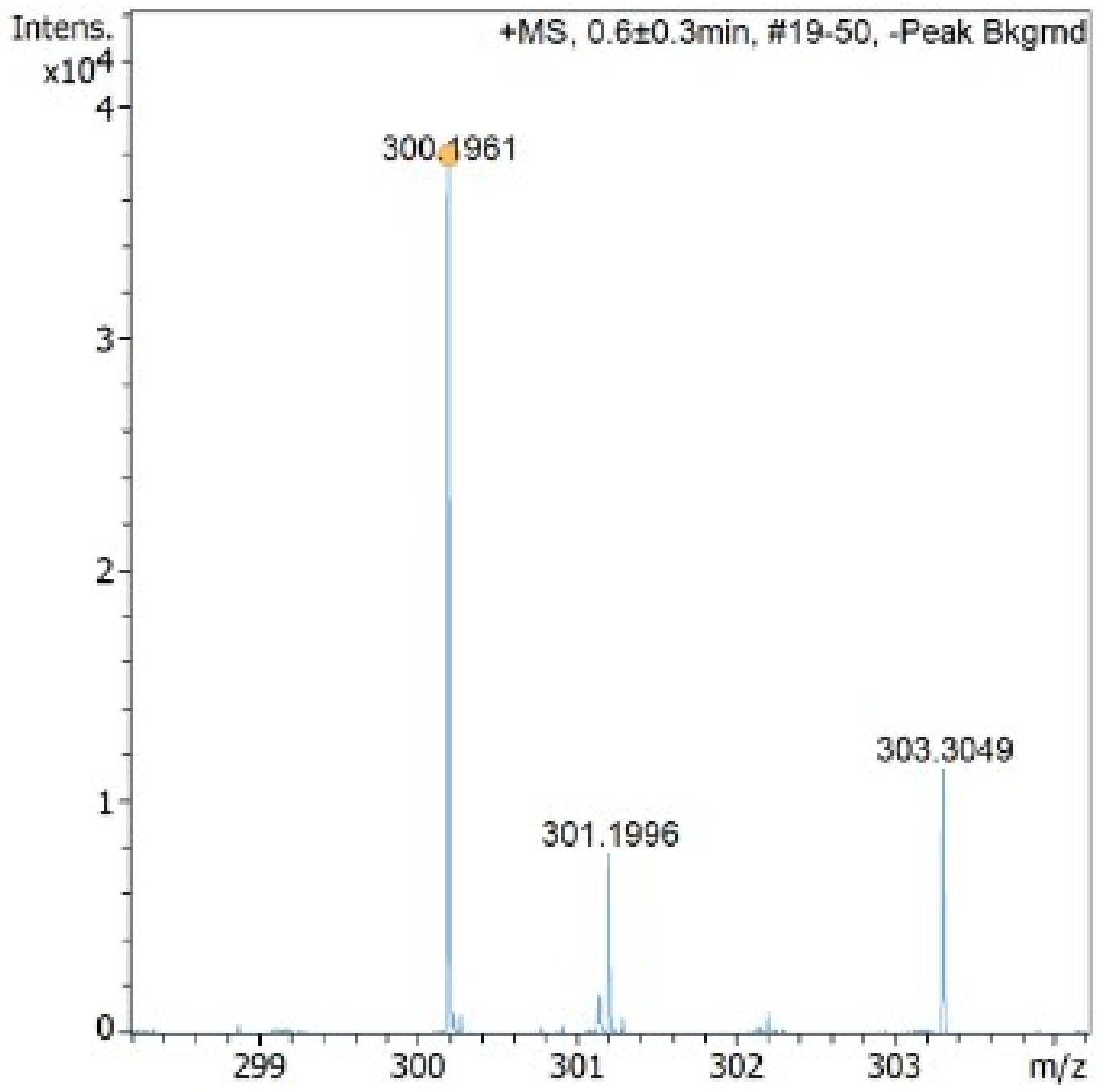

Figure S6 HRMS of **CZP-5-N**

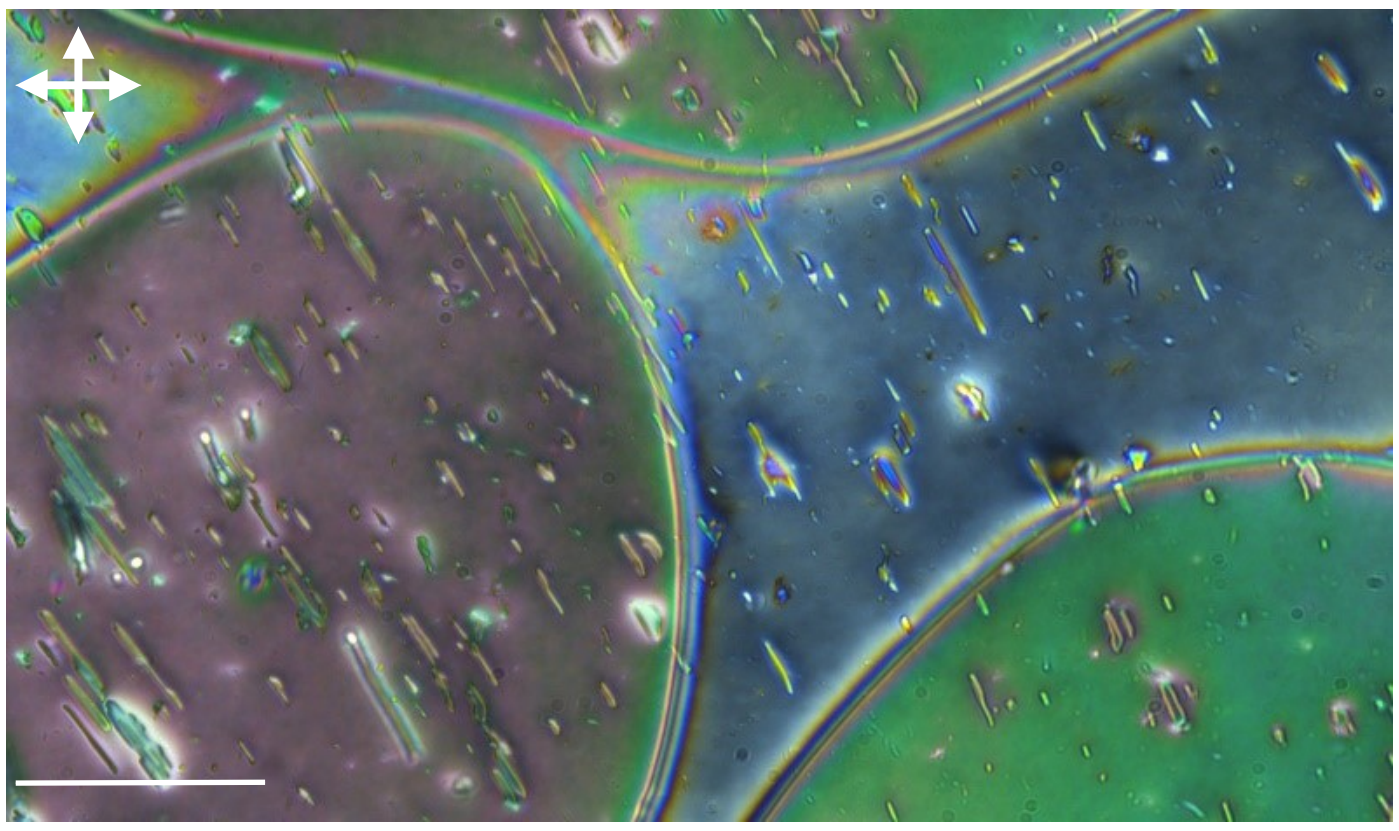

Figure S7, POM image of **CZP-5-N** at 65 °C, scale bar (bottom-left) shows 1 μm, arrows show polariser direction.

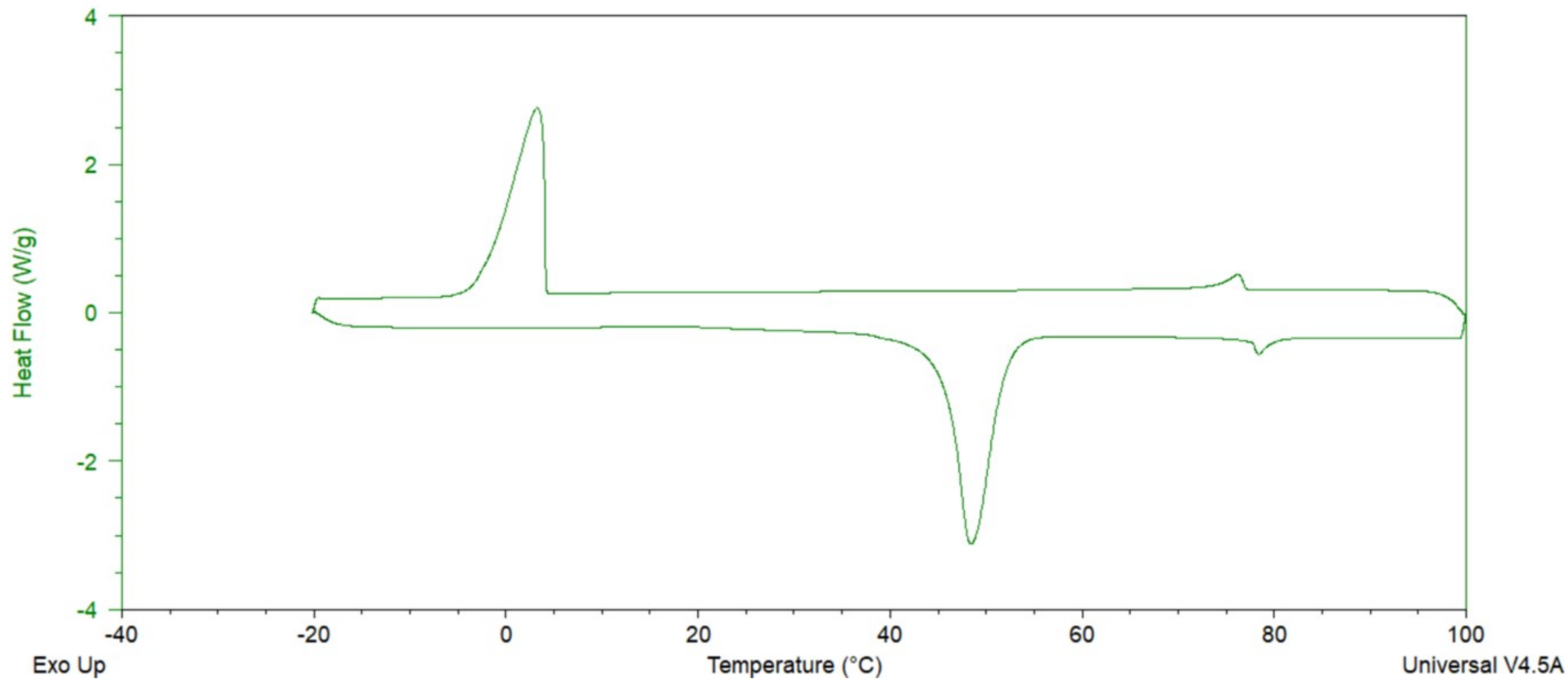

Figure S8 DSC thermogram of **CZP-5-N**

1 | *trans-[3-fluoro-4-(3,4,5-trifluorophenyl)phenyl] 4-pentylcyclohexane-1-carboxylate*

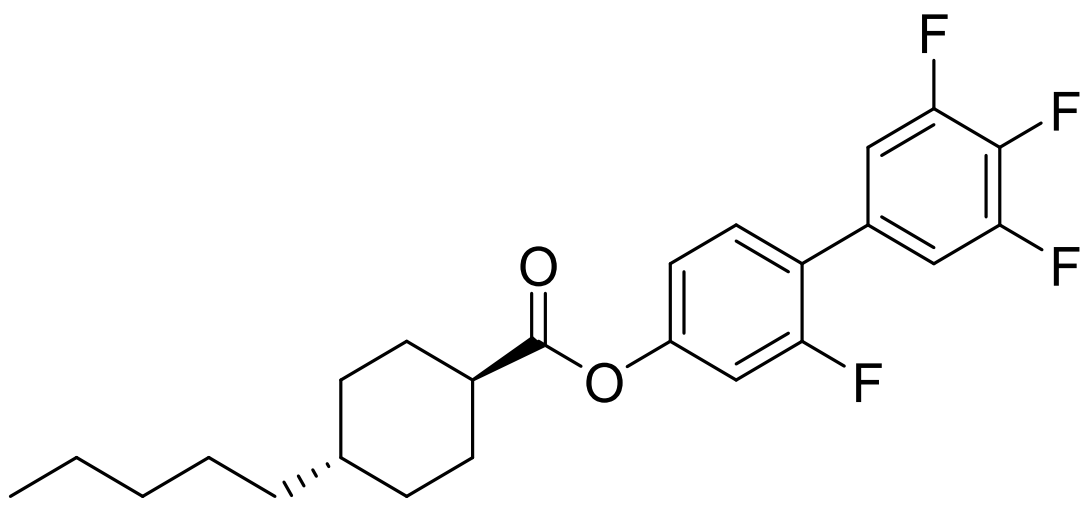

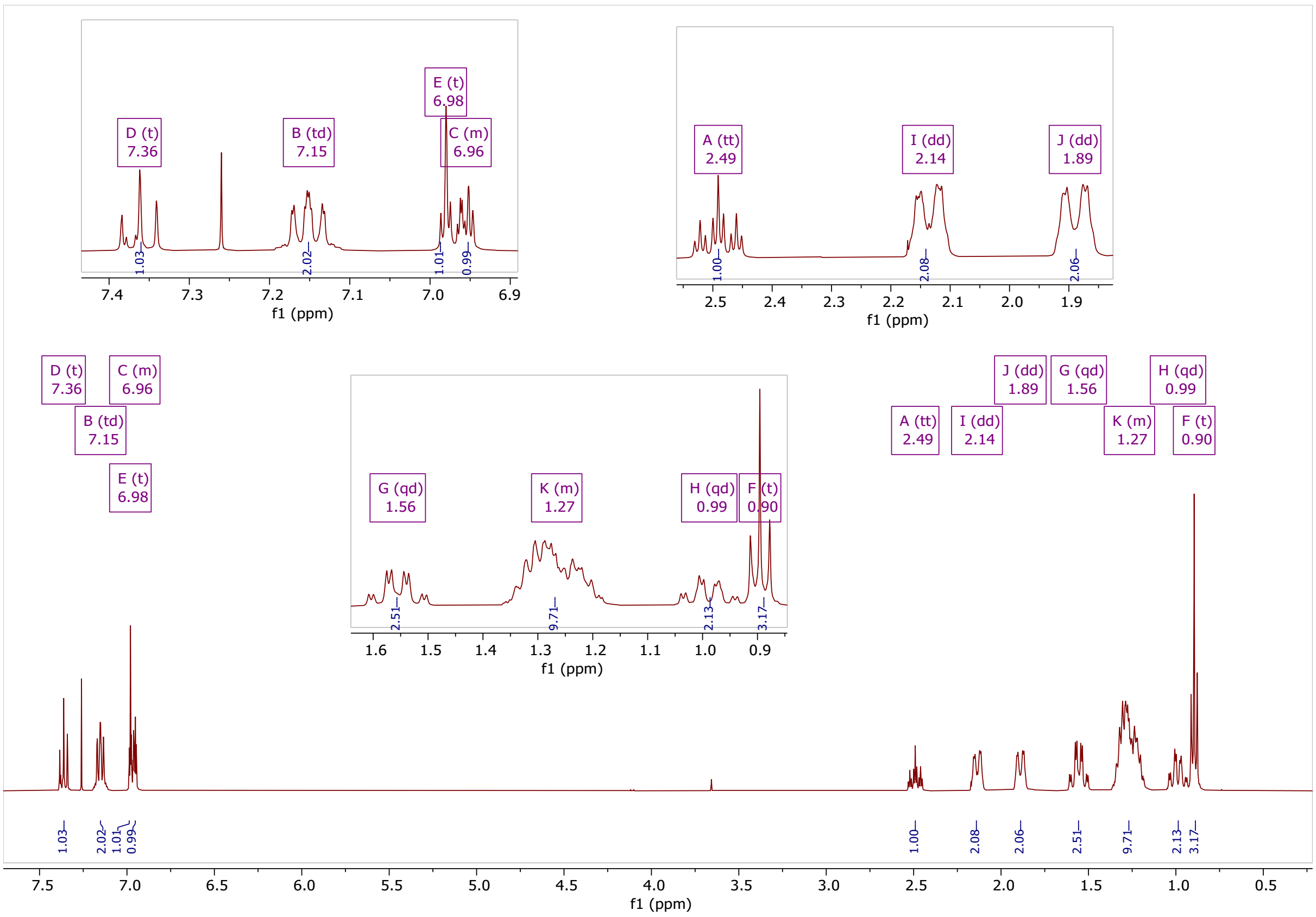

Figure S9 proton NMR spectrum of **1**

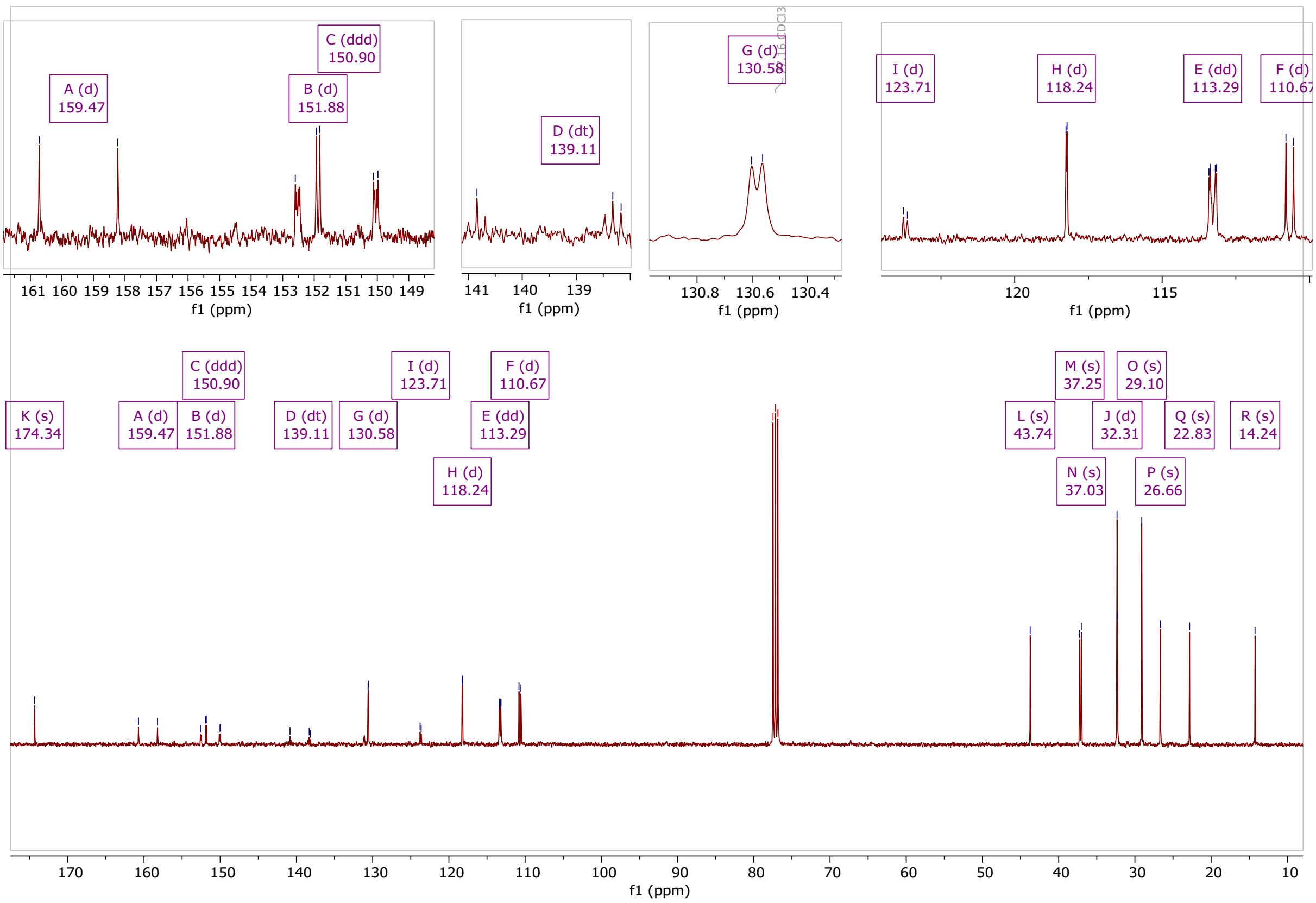

Figure S10 carbon NMR spectrum of **1**

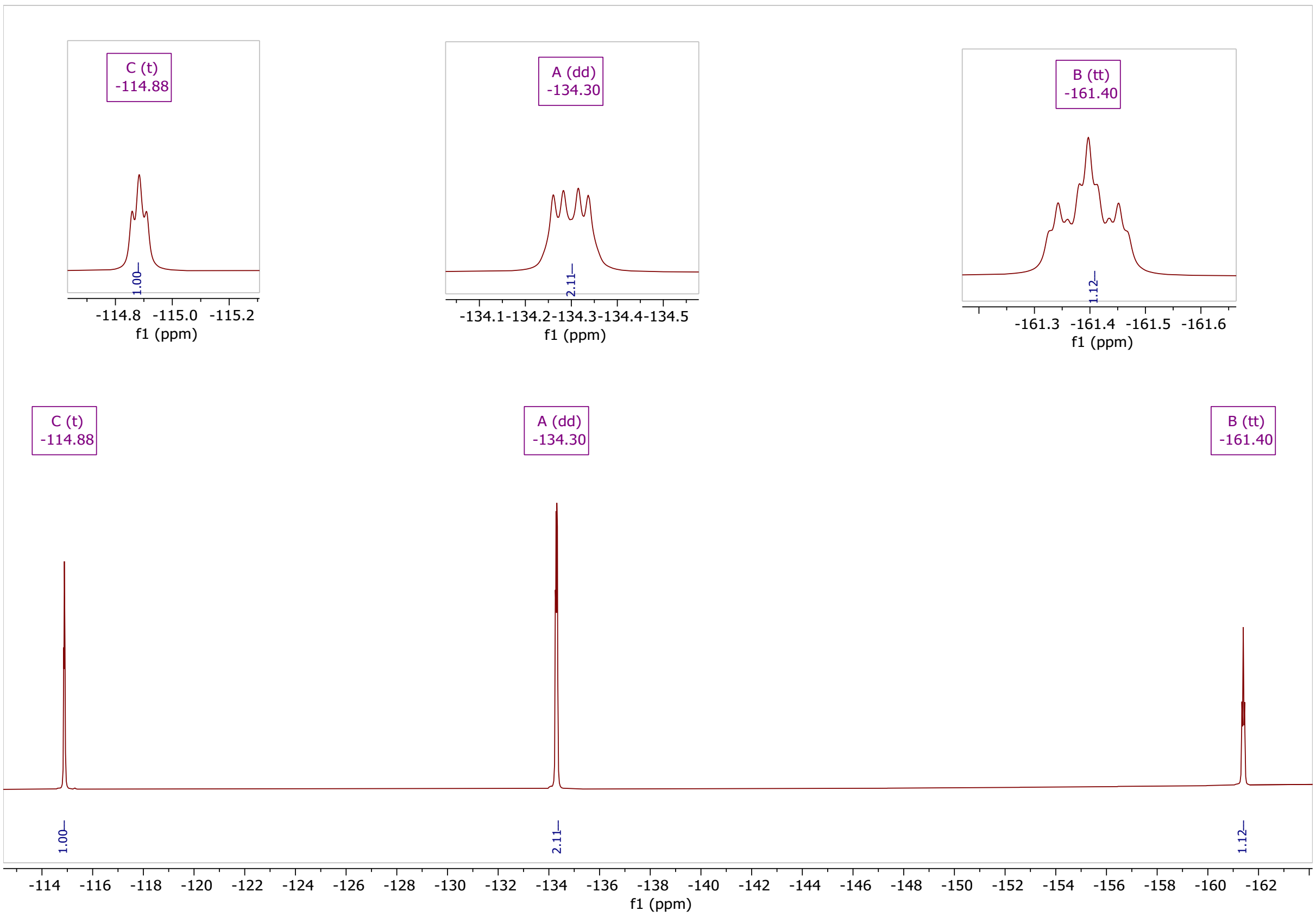

Figure S11 fluorine NMR spectrum of **1**

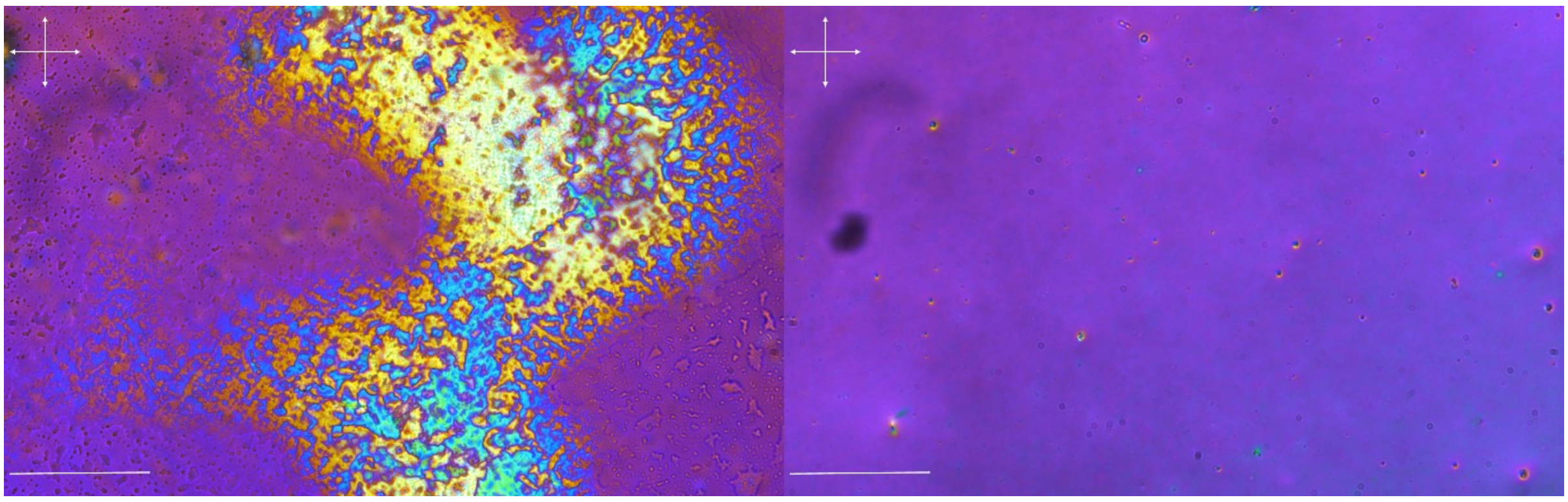

Figure S12 POM images of **1**. Left image at 57 °C, right image at 63 °C, both showing a nematic phase. Scale bar (bottom-left) shows 1 μm, arrows show polariser direction.

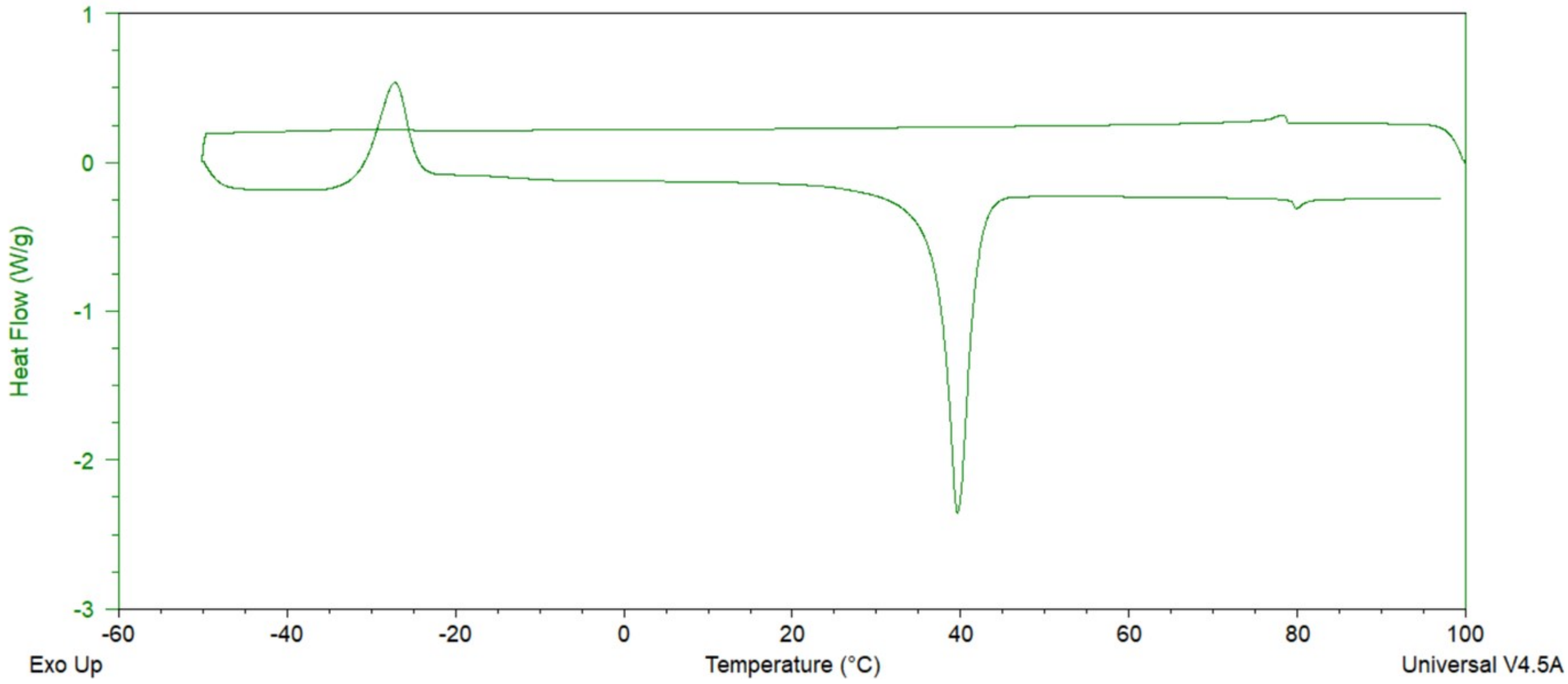

Figure S13 DSC thermogram of **1**.

2 | *[4-(4-cyanophenyl)phenyl] 4-pentylcyclohexane-1-carboxylate*

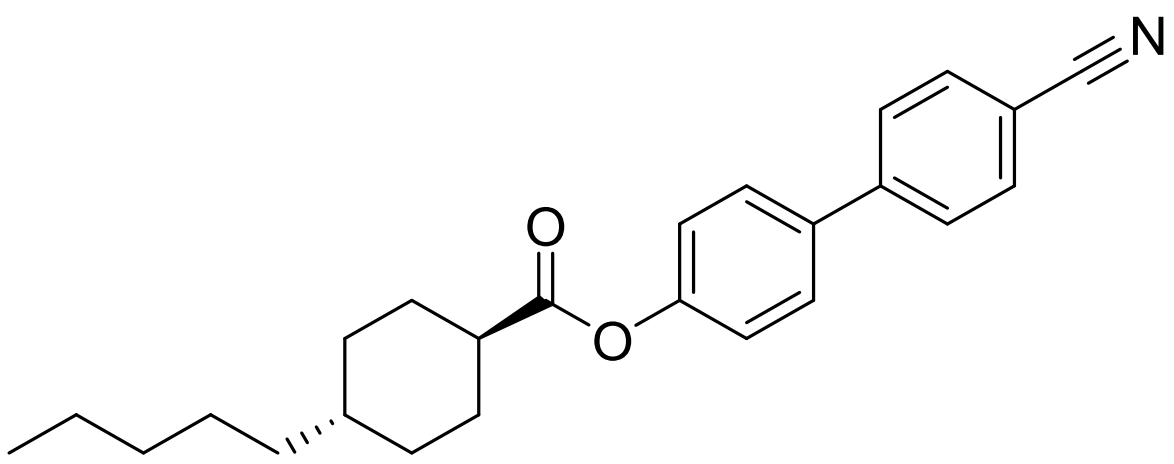

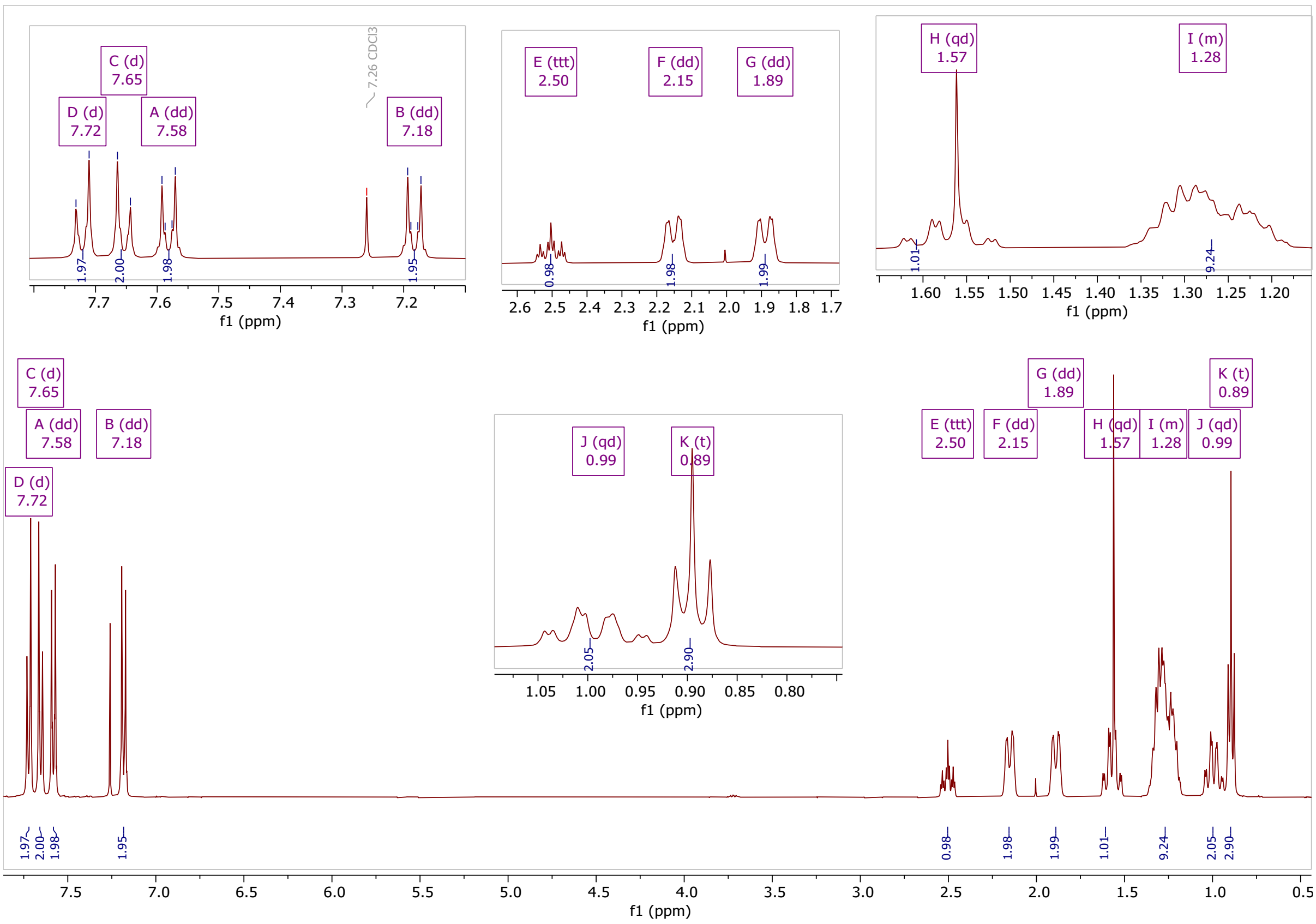

Figure S14 proton NMR spectrum of **2**

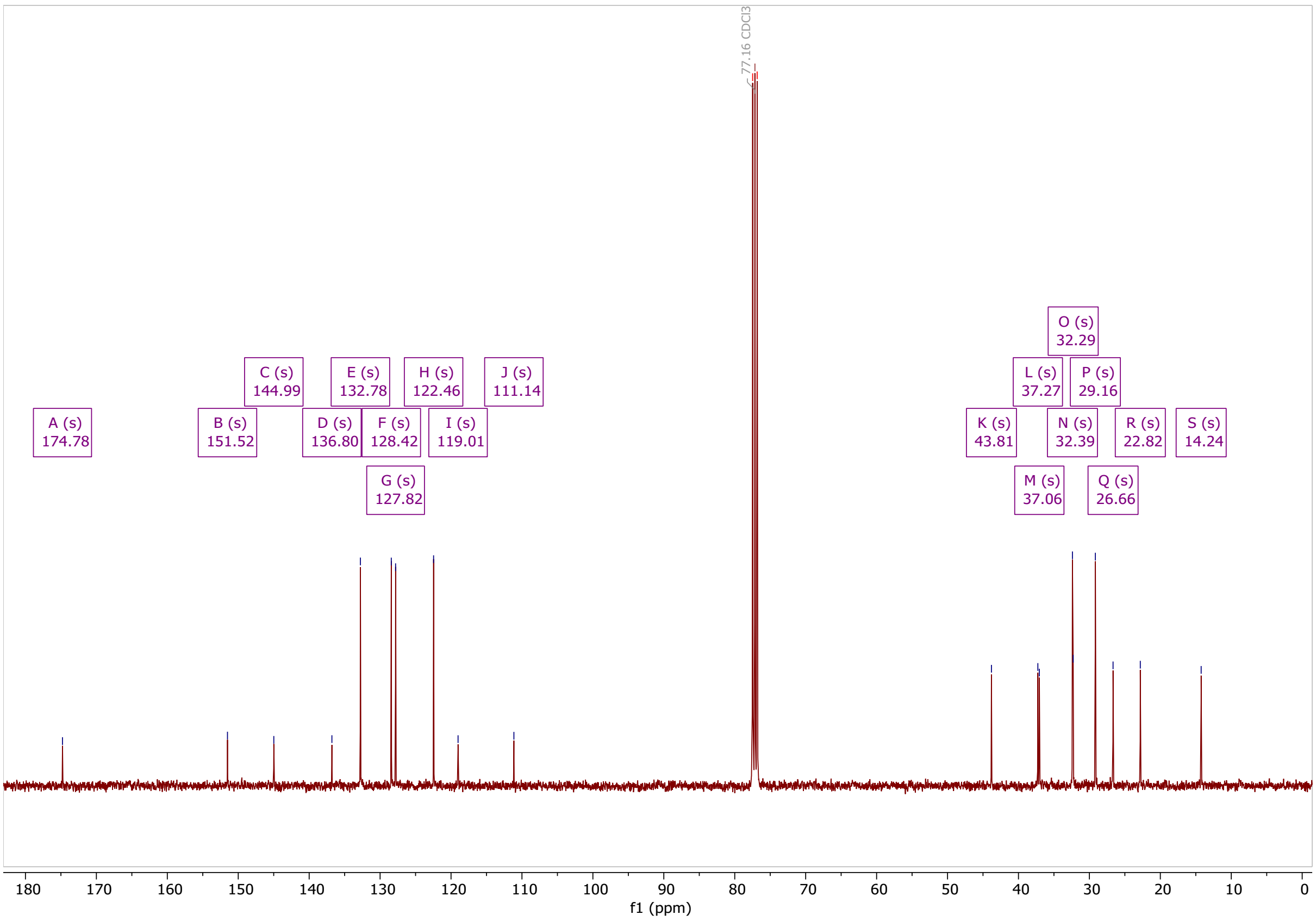

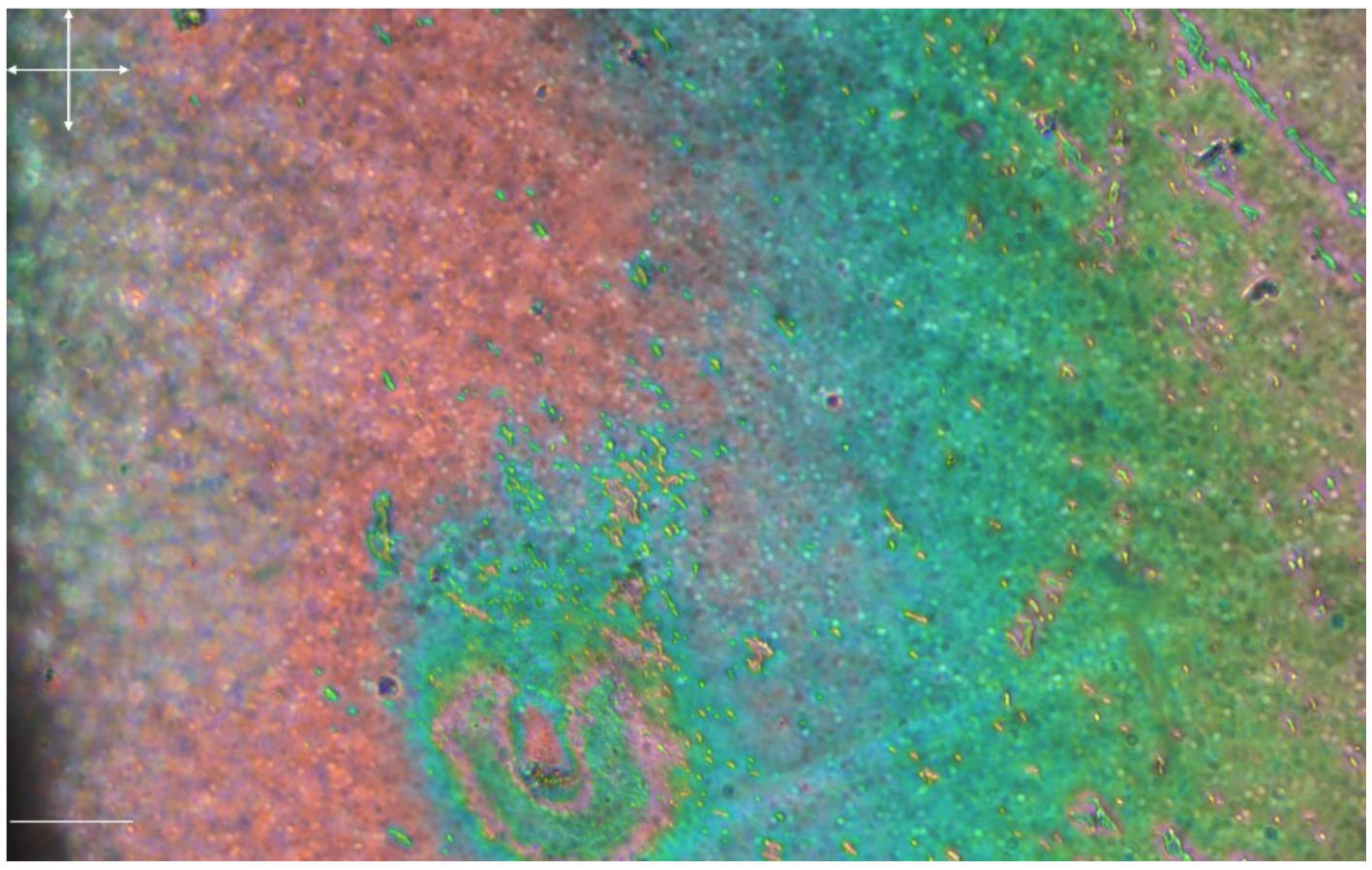

Figure S15 carbon NMR spectrum of **2**

Figure S16 POM image of **2**, nematic phase at 160 °C. Scale bar (bottom-left) shows 1 µm, arrows show polariser direction.

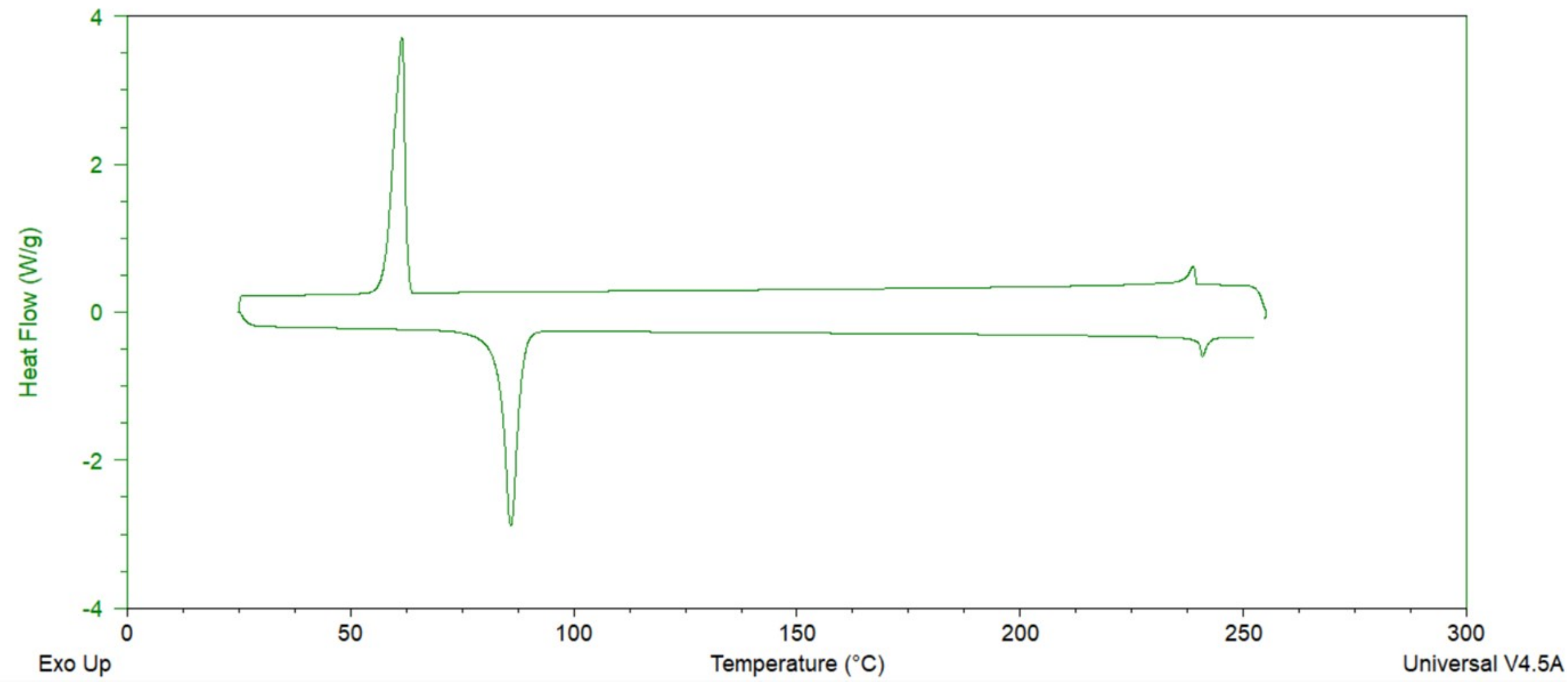

Figure S17 DSC thermogram of **2**.

3 | *(4-nitrophenyl) 4-pentylcyclohexane-1-carboxylate*

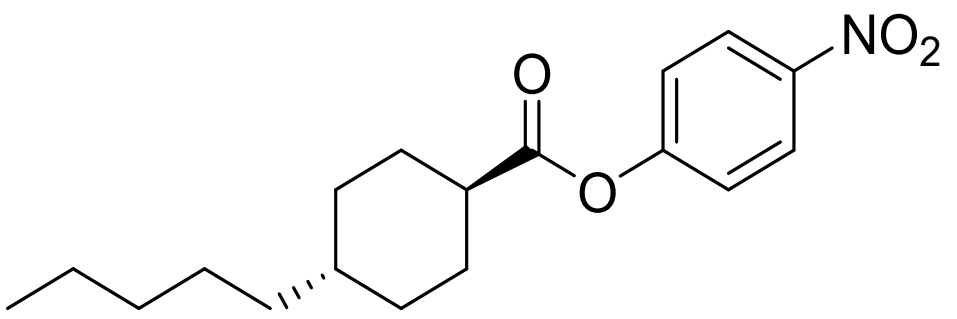

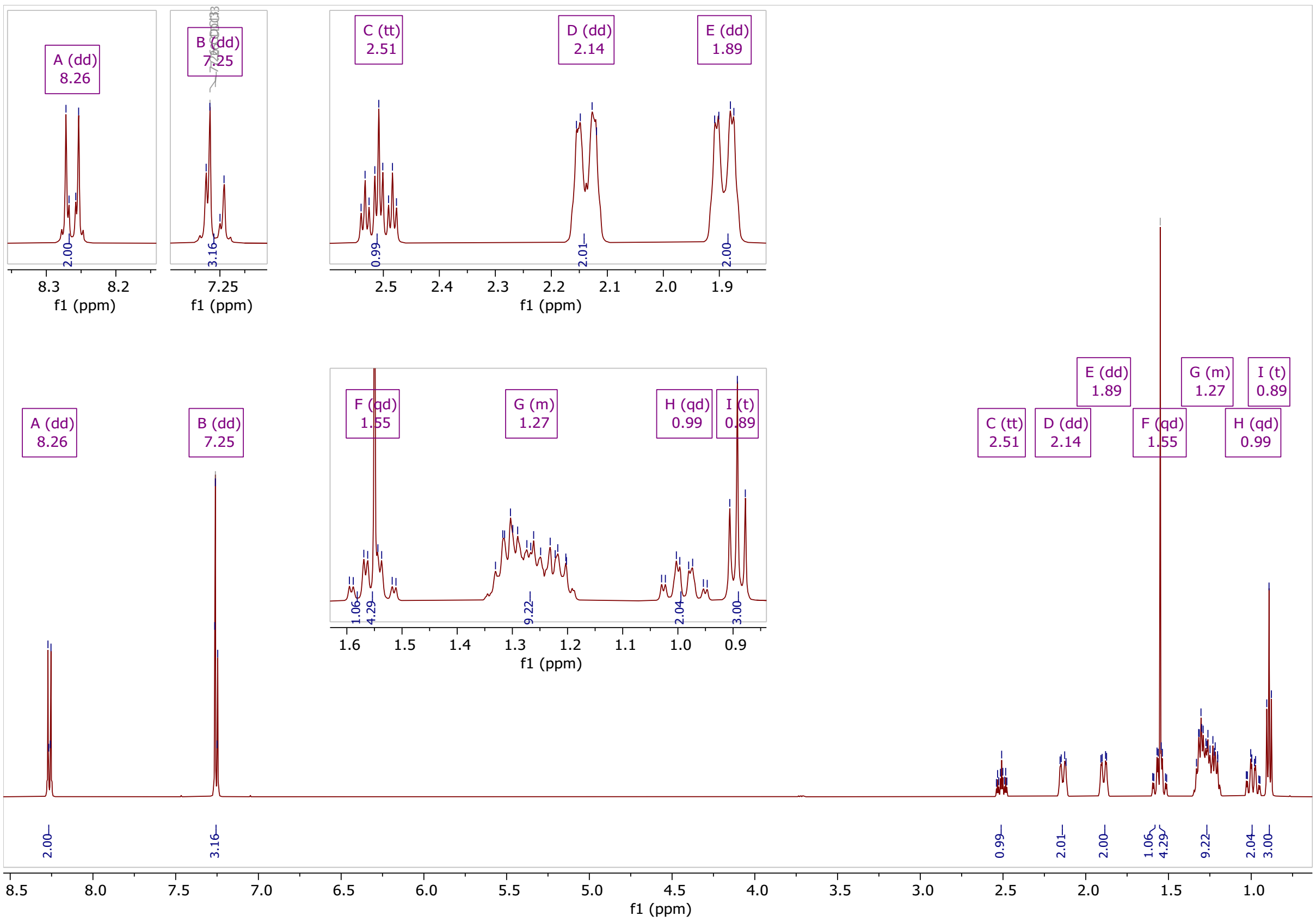

Figure S18 proton NMR spectrum of **3.**

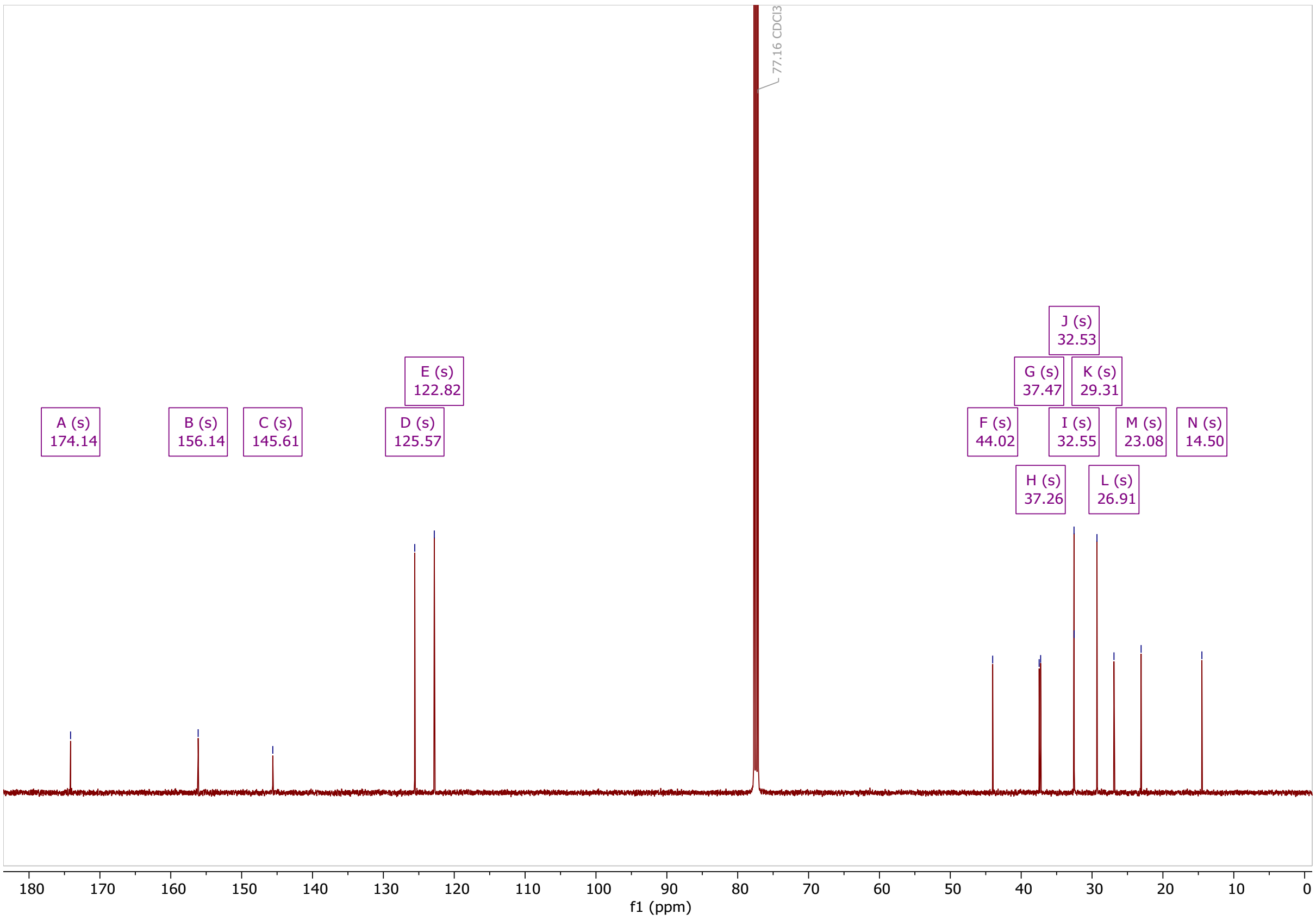

Figure S19 carbon NMR spectrum of **3**

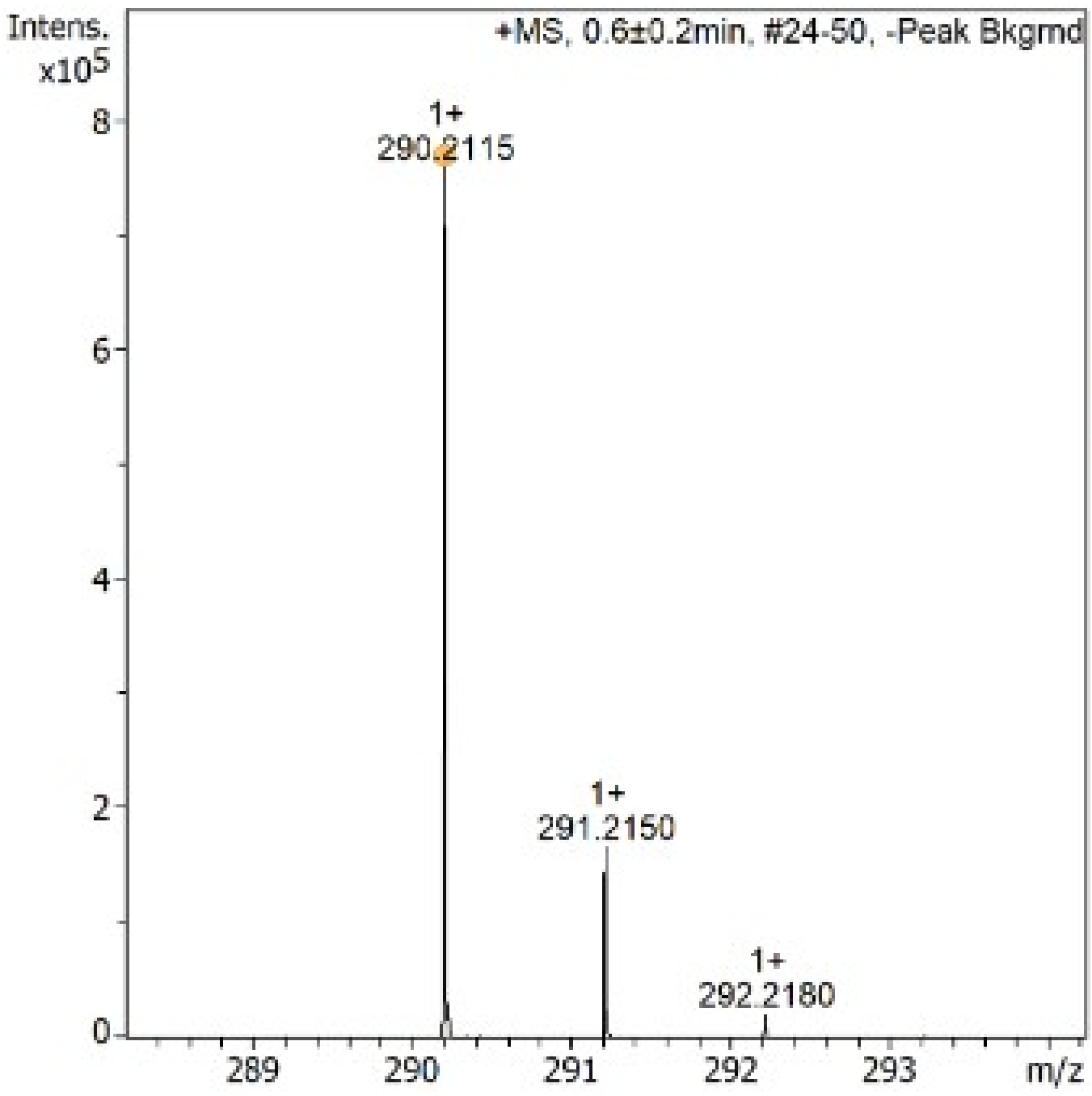

Figure S20 HRMS of **3.** Nitro group reduced to amine group in flight.

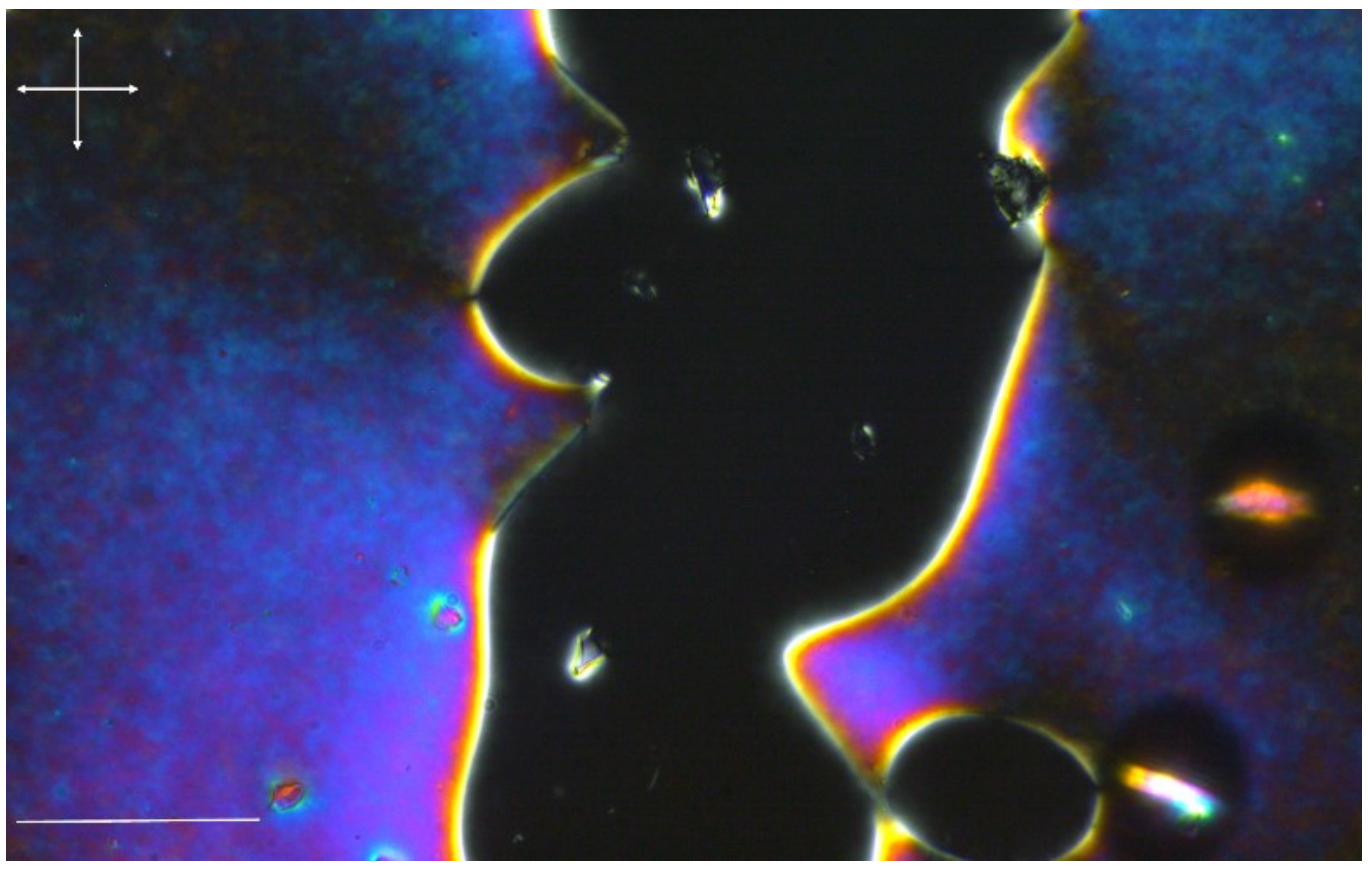

Figure S21 POM image of **3**. Nematic phase at 37 C. Scale bar (bottom-left) shows 1 µm, arrows show polariser direction.

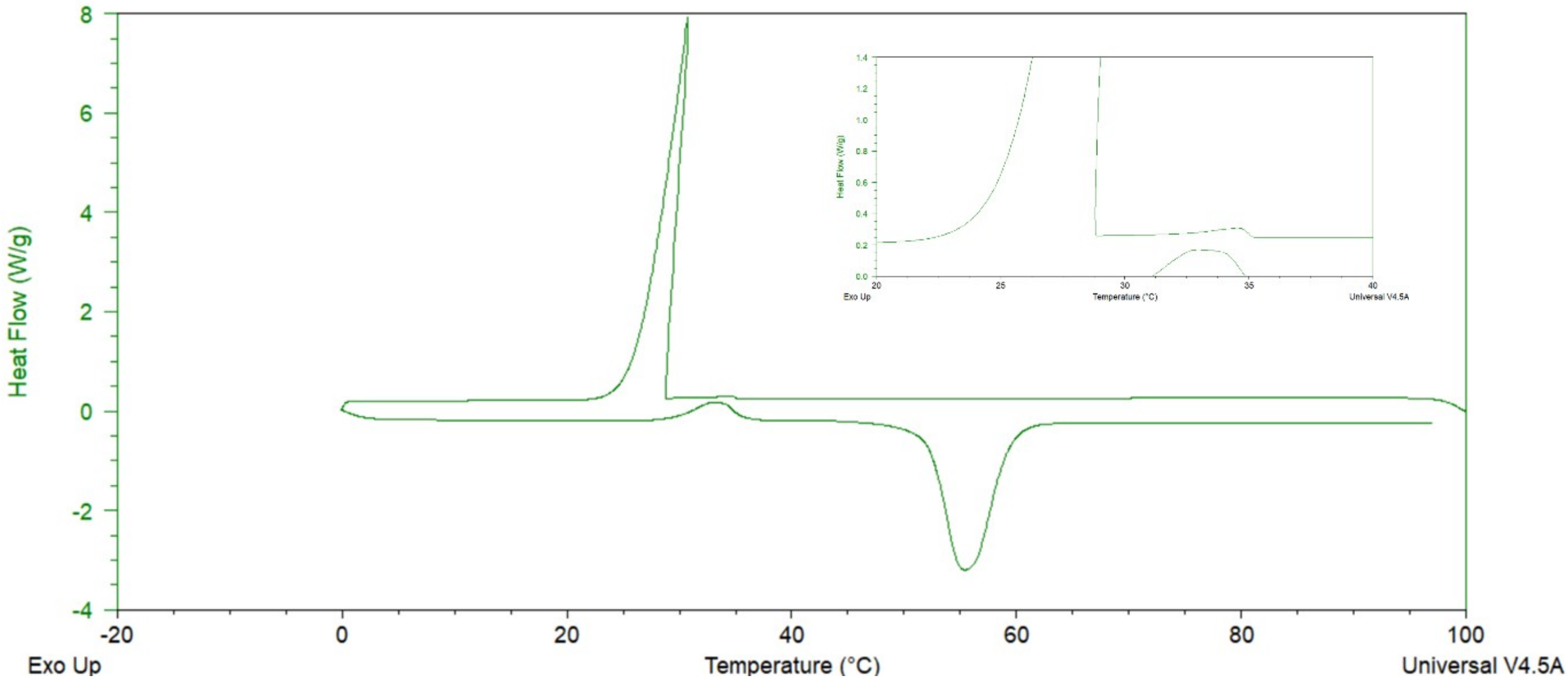

Figure S22 DSC thermogram of **3**.

4 | *4-(5-heptylpyrimidin-2-yl)phenyl (1r,4s)-4-pentylcyclohexane-1-carboxylate*

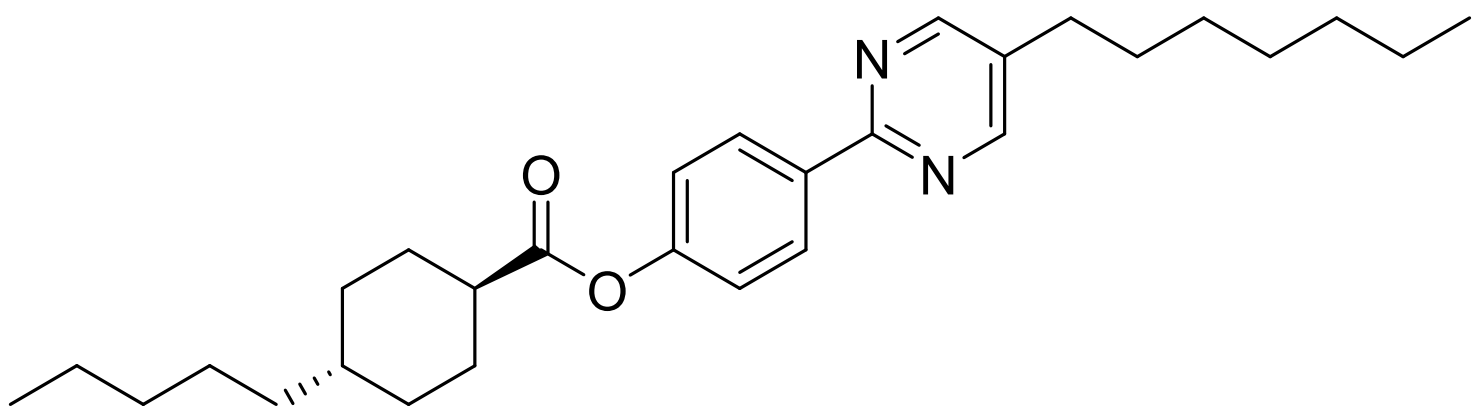

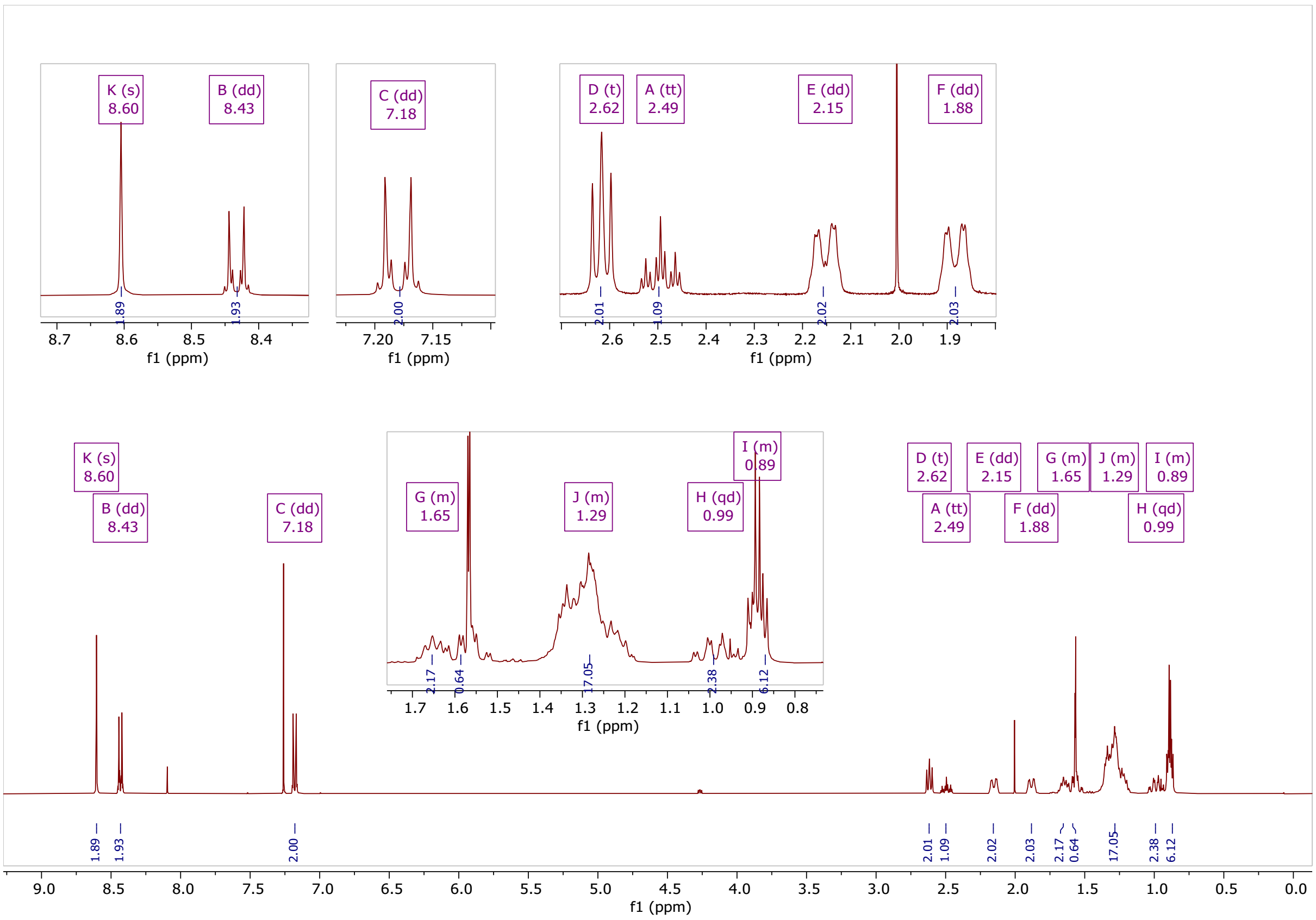

Figure S23 Proton NMR spectrum of **4**.

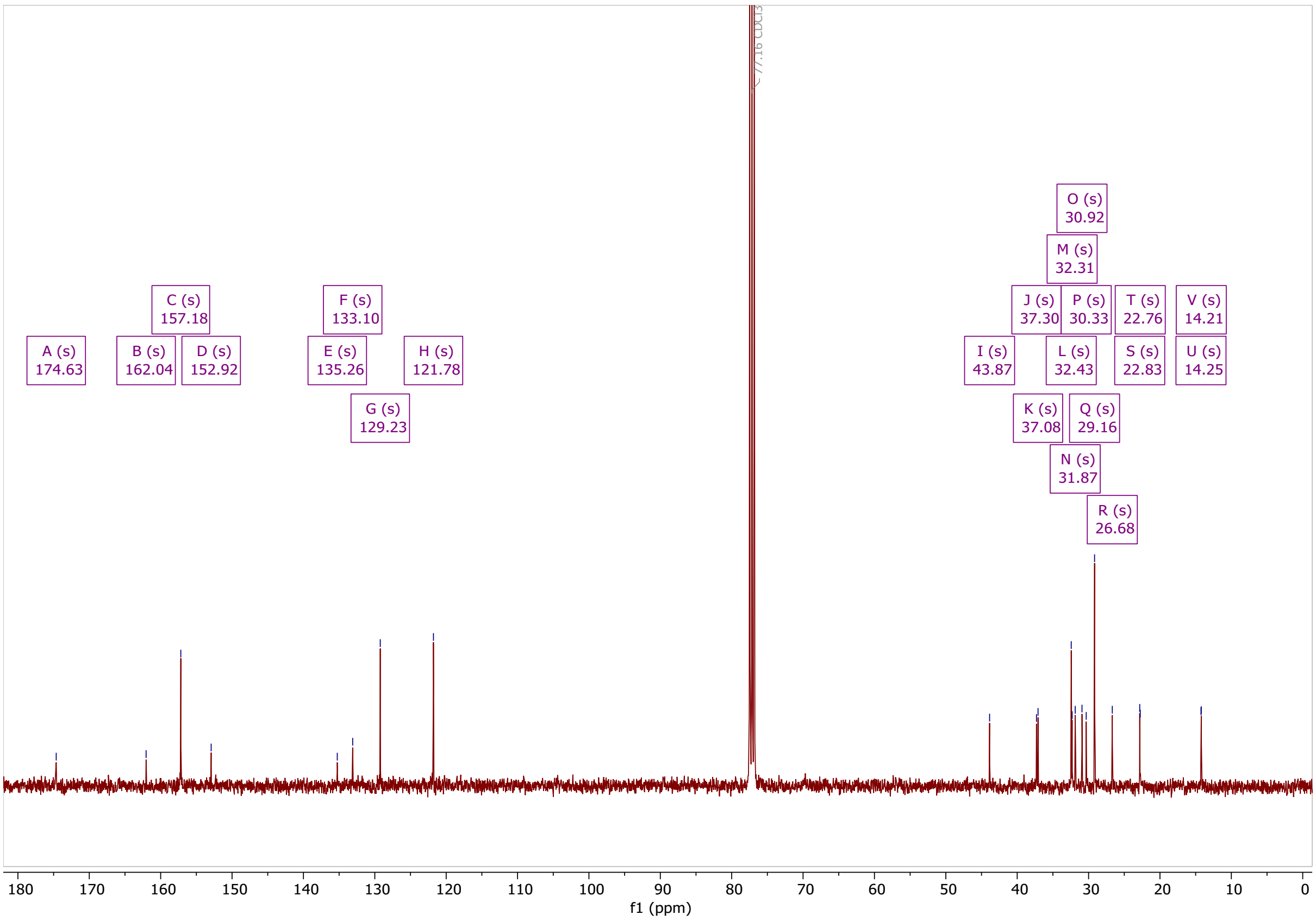

Figure S24 Carbon NMR spectrum of **4**.

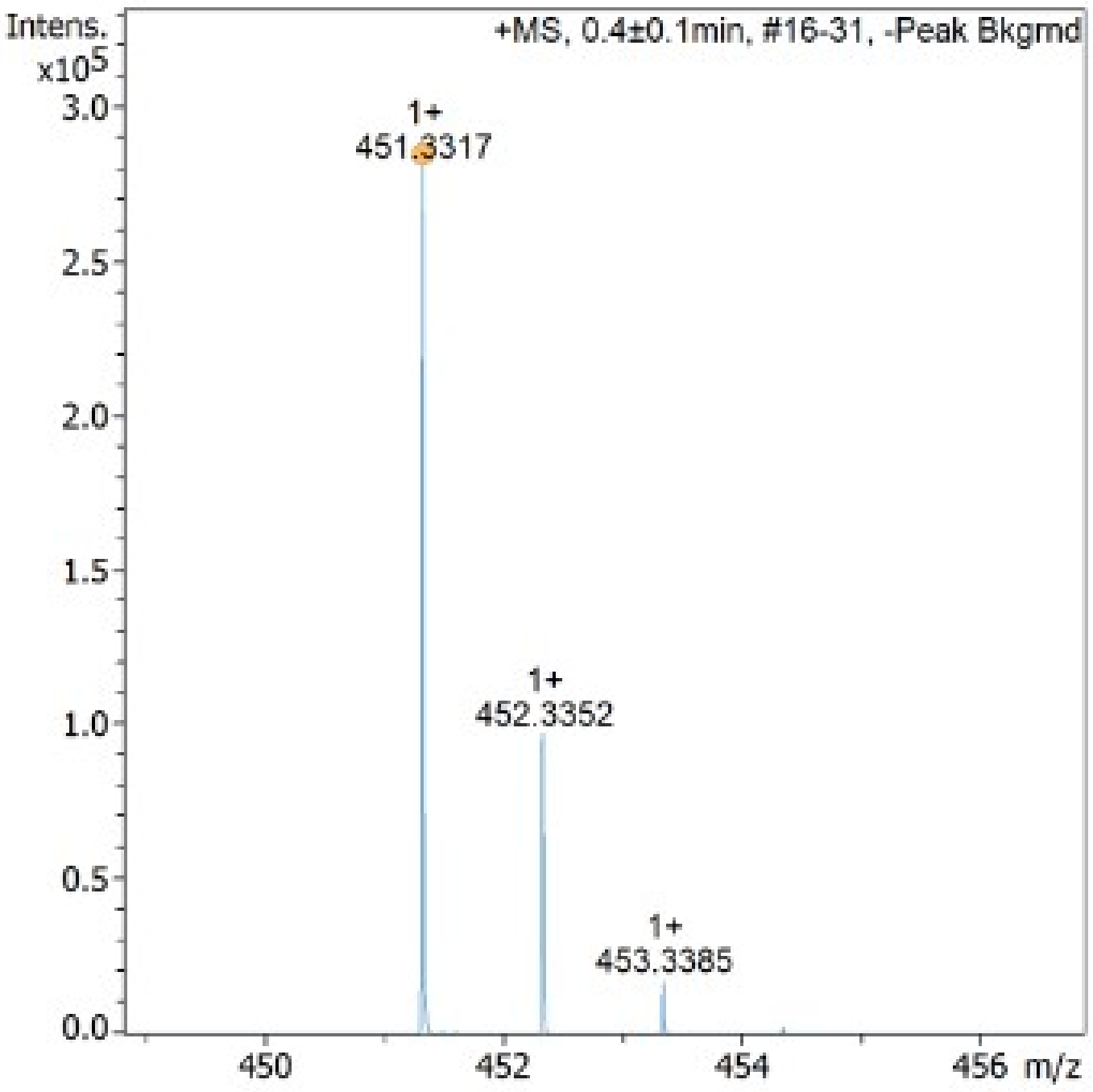

Figure S25 HRMS of **9**

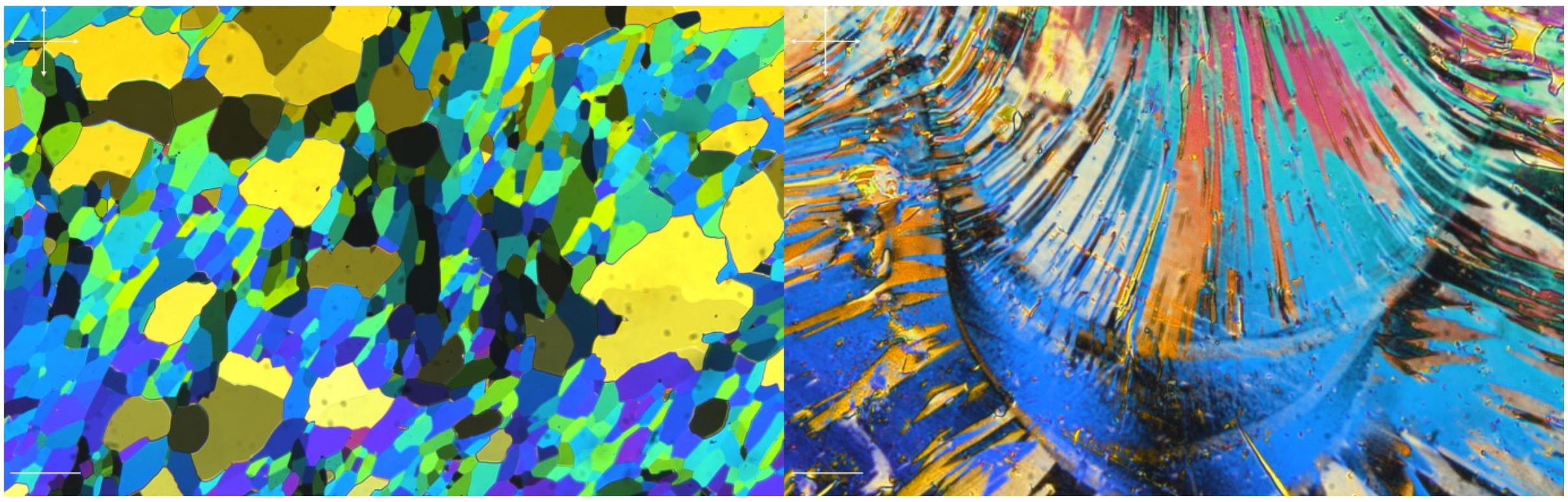

Figure S26 POM images of **4**. Left image HexI at 90 °C. Right image N at 110 °C. Scale bar (bottom-left) shows 1 μm, arrows show polariser direction.

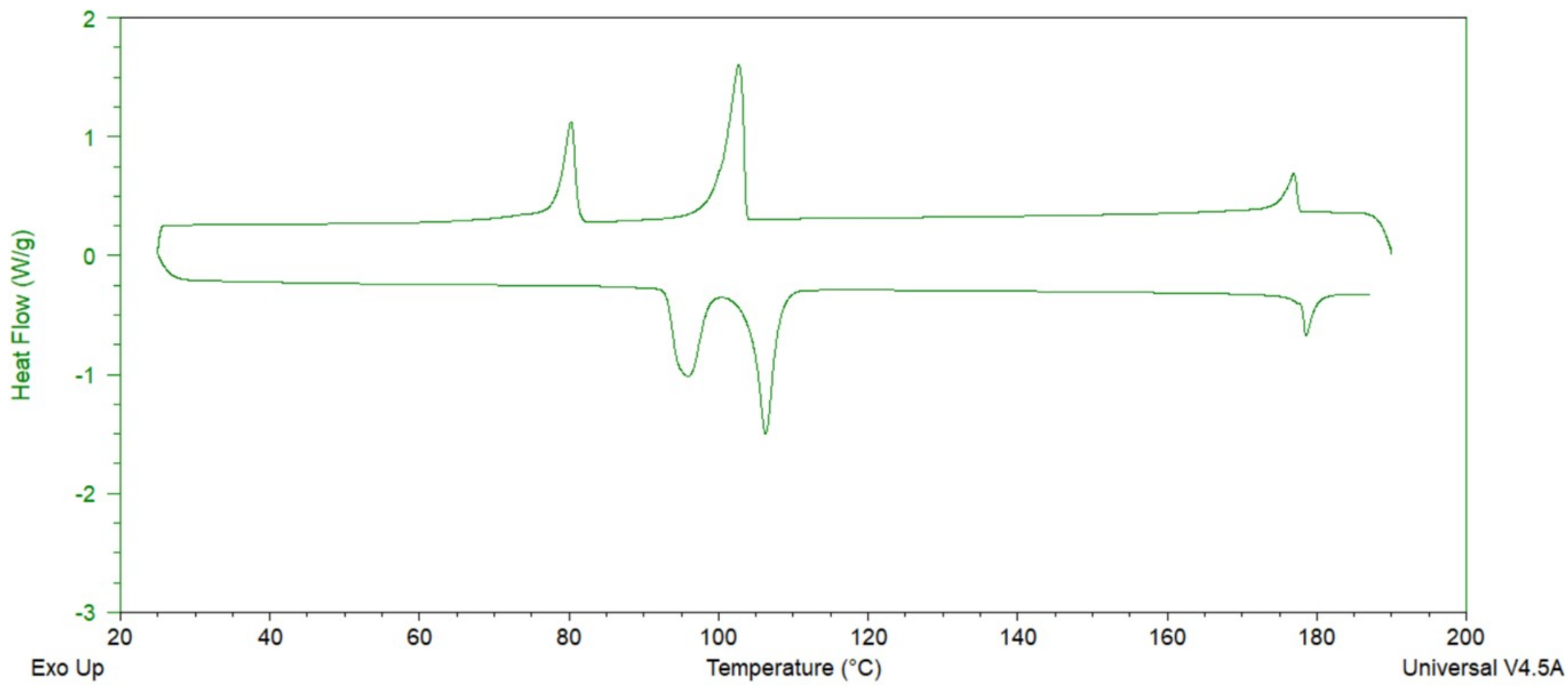

Figure S27 DSC thermogram of **4**.

5 | *2,3-difluoro-4'-((1s,4r)-4-pentylcyclohexyl)-[1,1'-biphenyl]-4-yl (1s,4r)-4-entylcyclohexane-1-carboxylate*

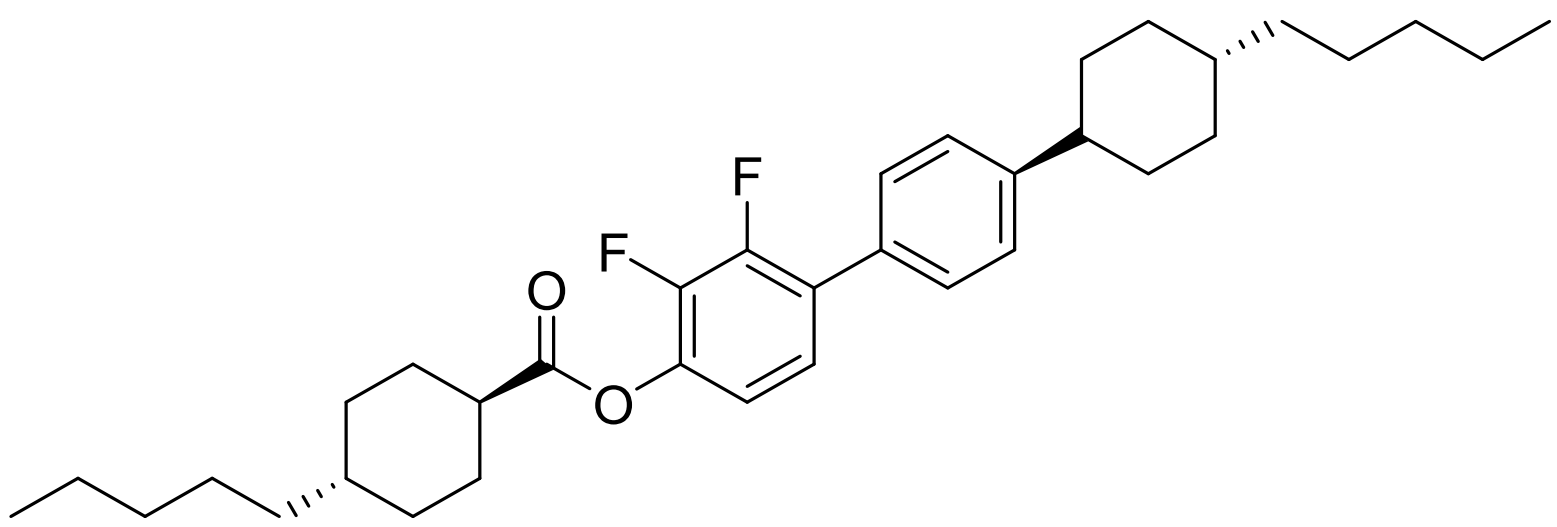

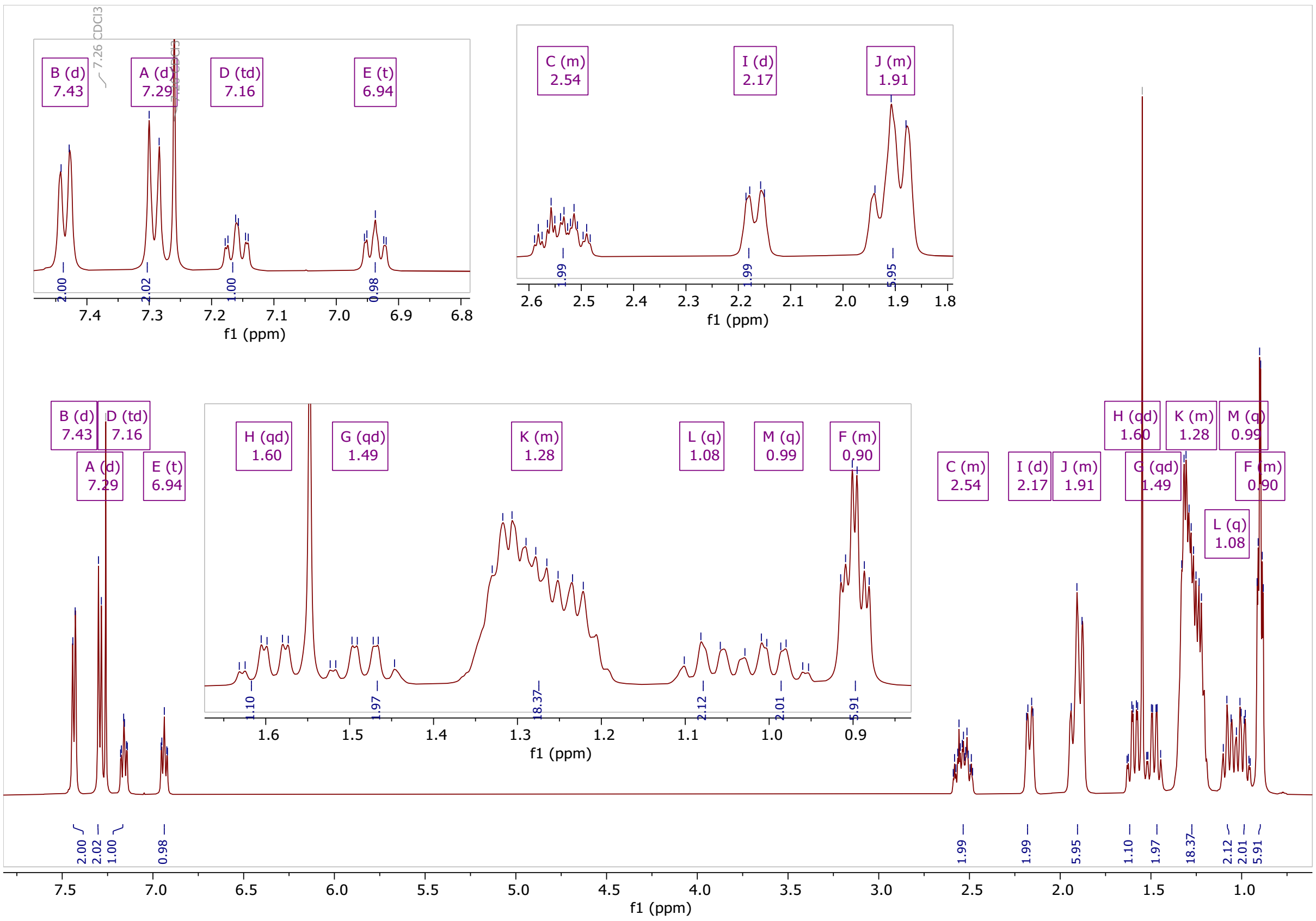

Figure S28 proton NMR spectrum of **5**

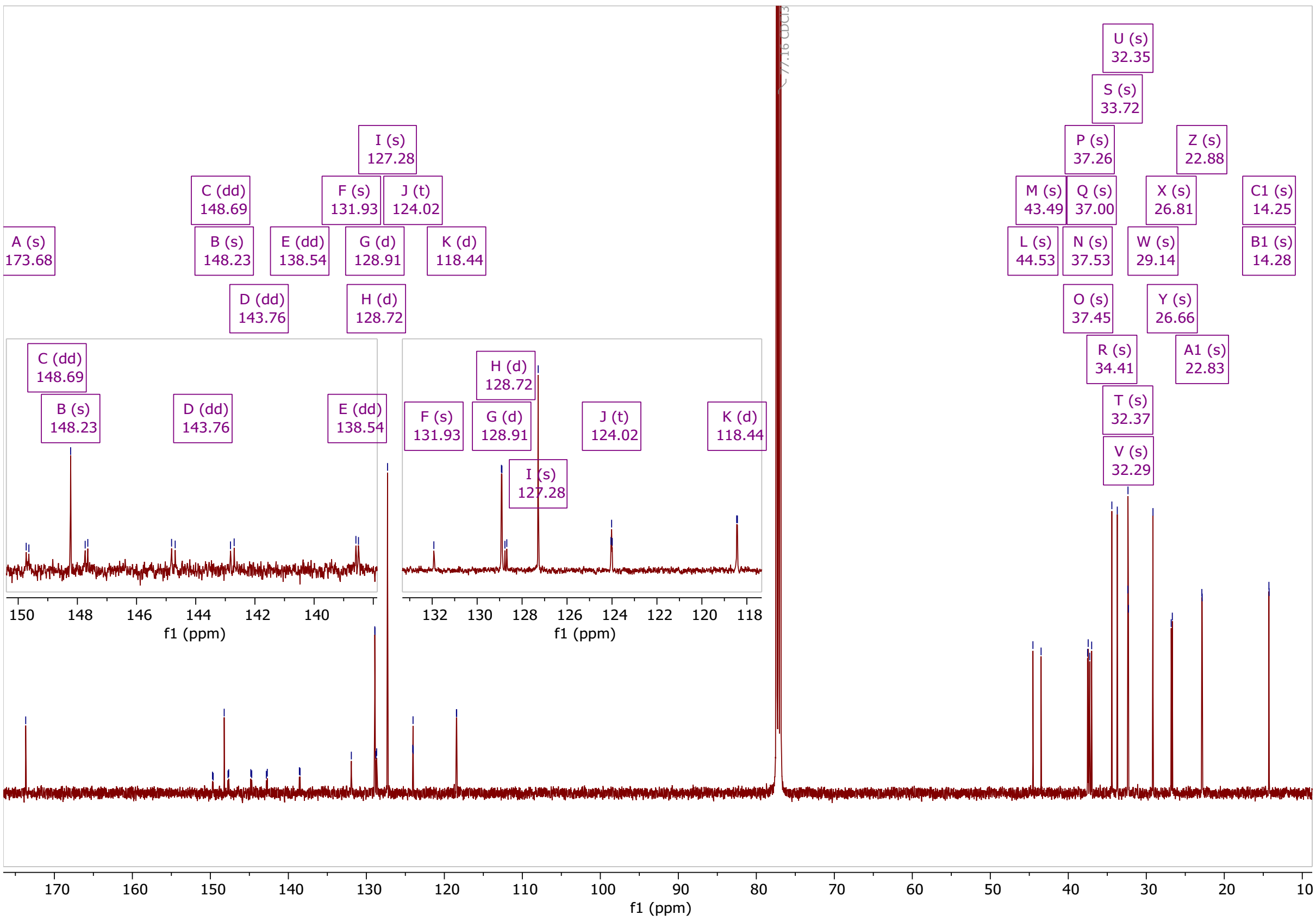

Figure S29 carbon NMR spectrum of **5**

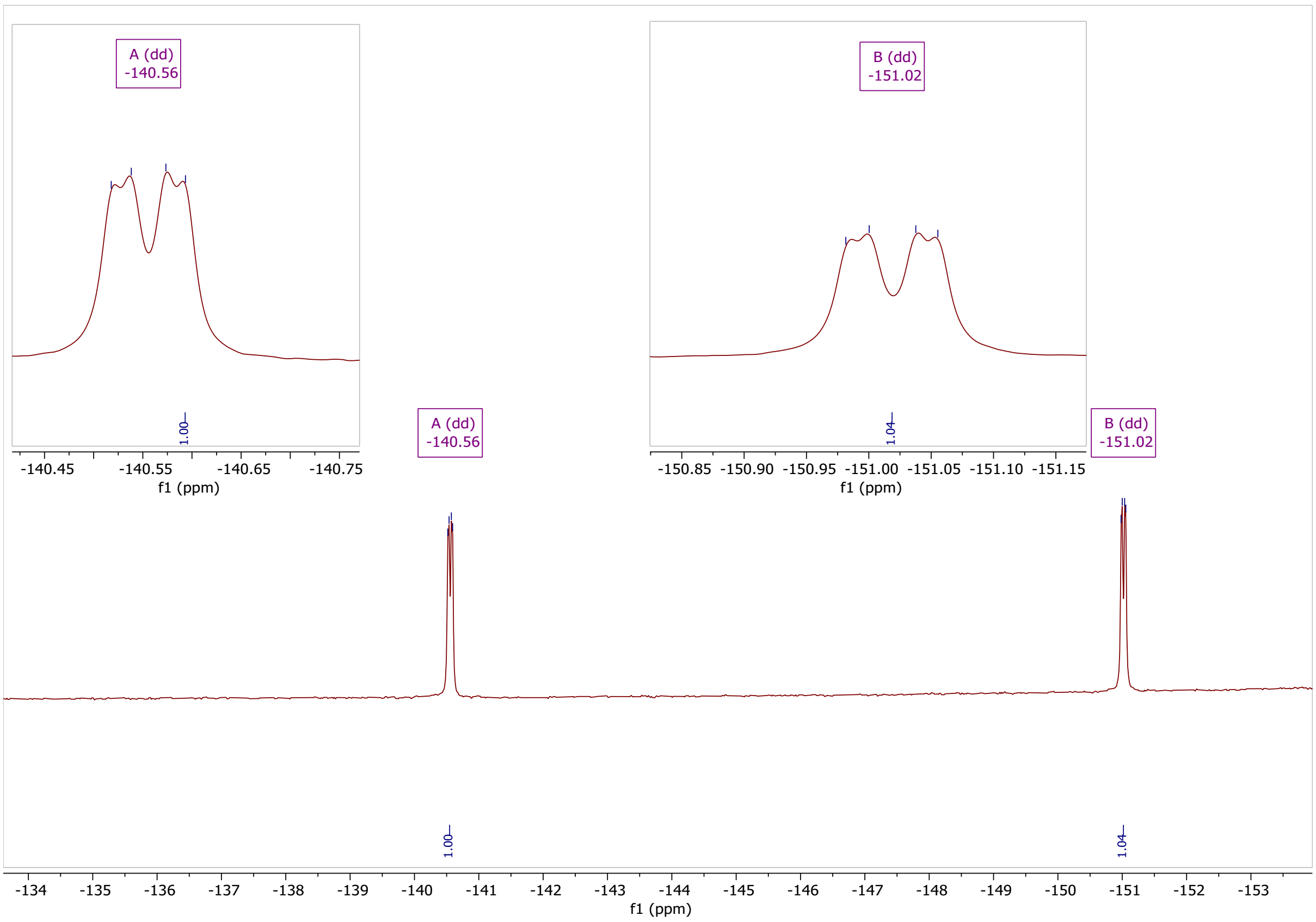

Figure S30 fluorine NMR spectrum of **5**

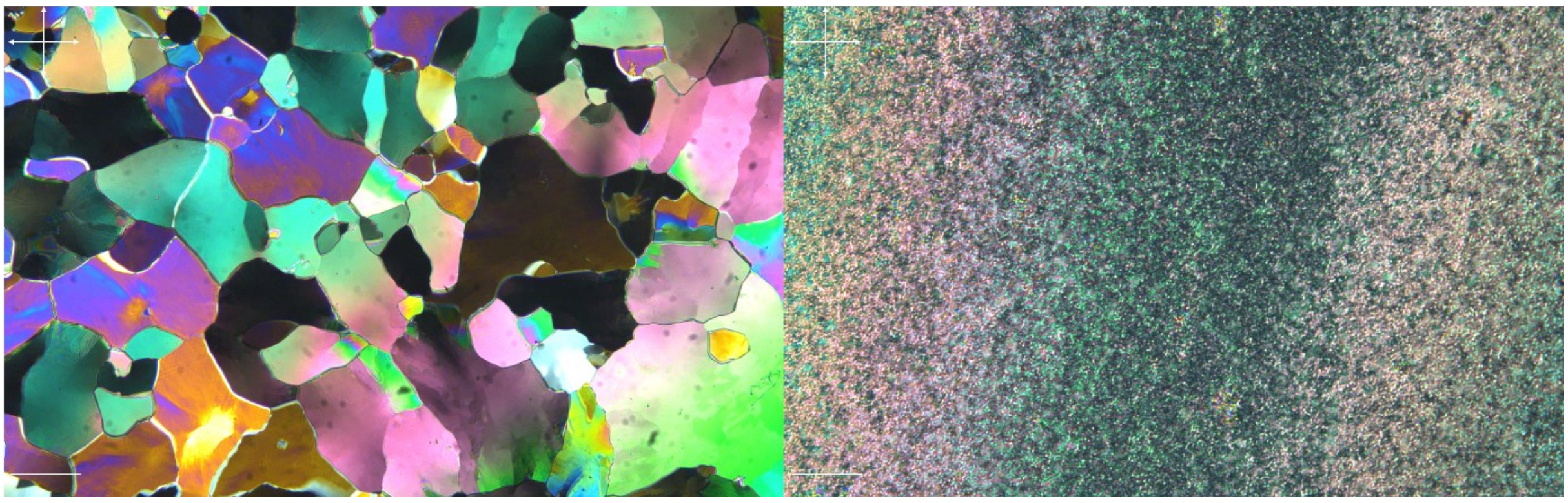

Figure S31 POM images of **5**. Left image HexI at 89 °C. Right image N at 147 °C. Scale bar (bottom-left) shows 1 μm, arrows show polariser direction.

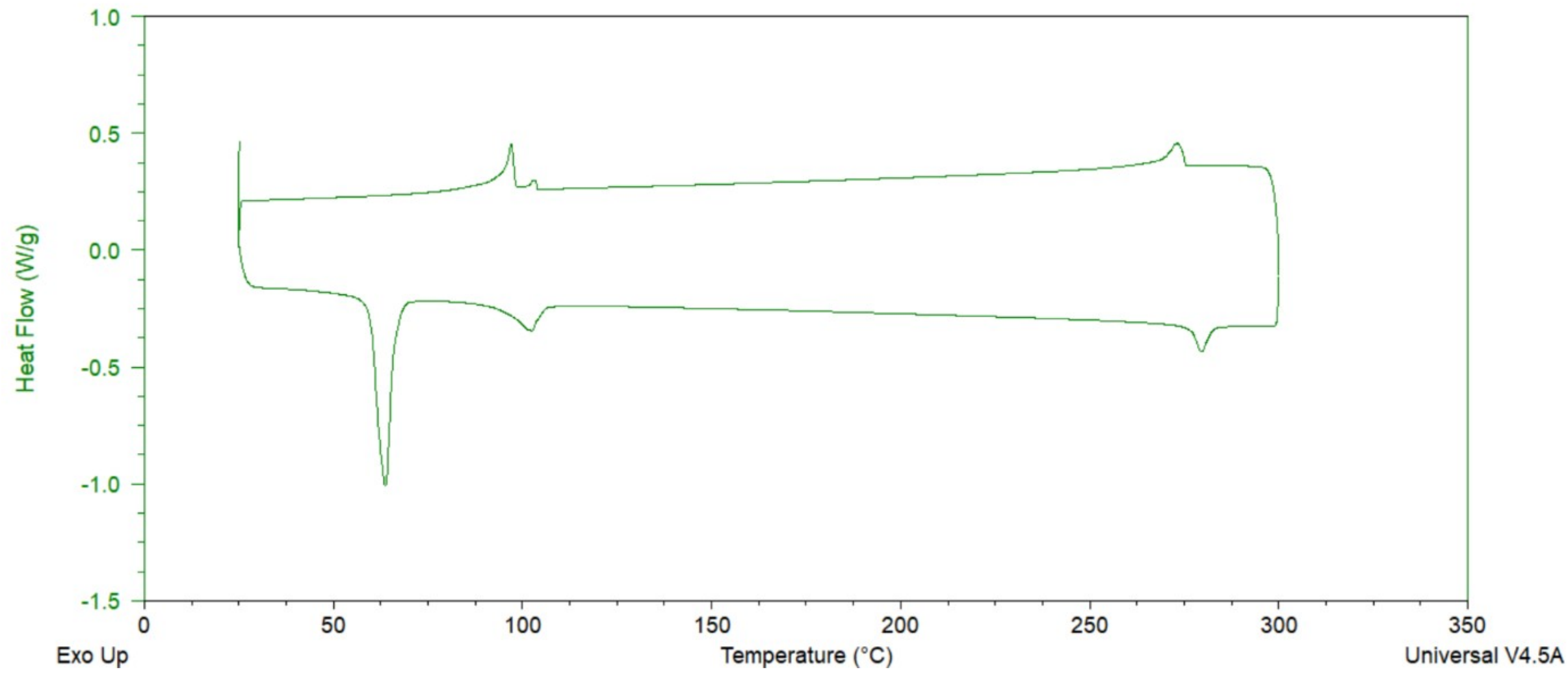

Figure S32 DSC thermogram of **5**.

6 | *4-((1s,4R)-4-pentylcyclohexyl)phenyl (1s,4R)-4-pentylcyclohexane-1-carboxylate*

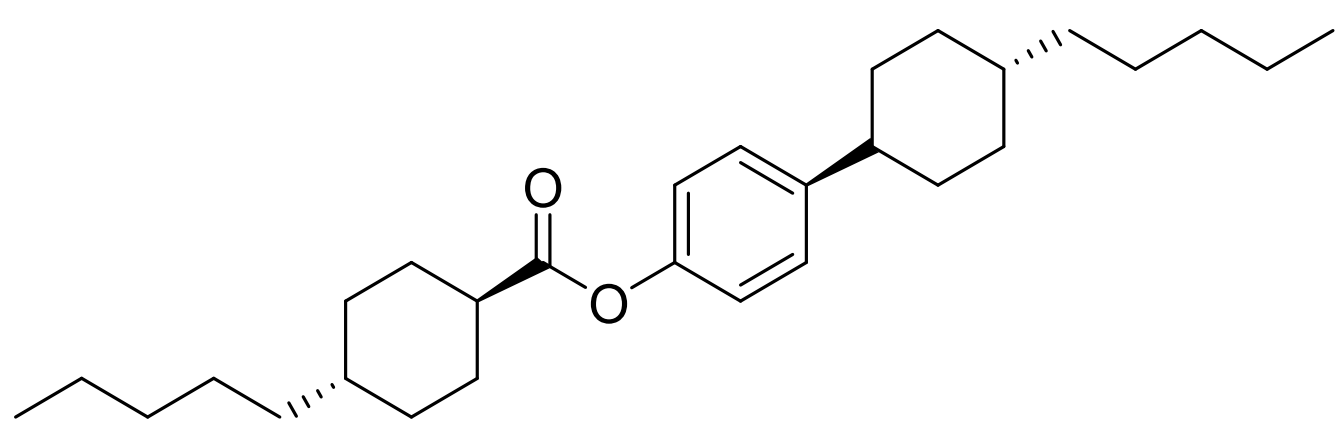

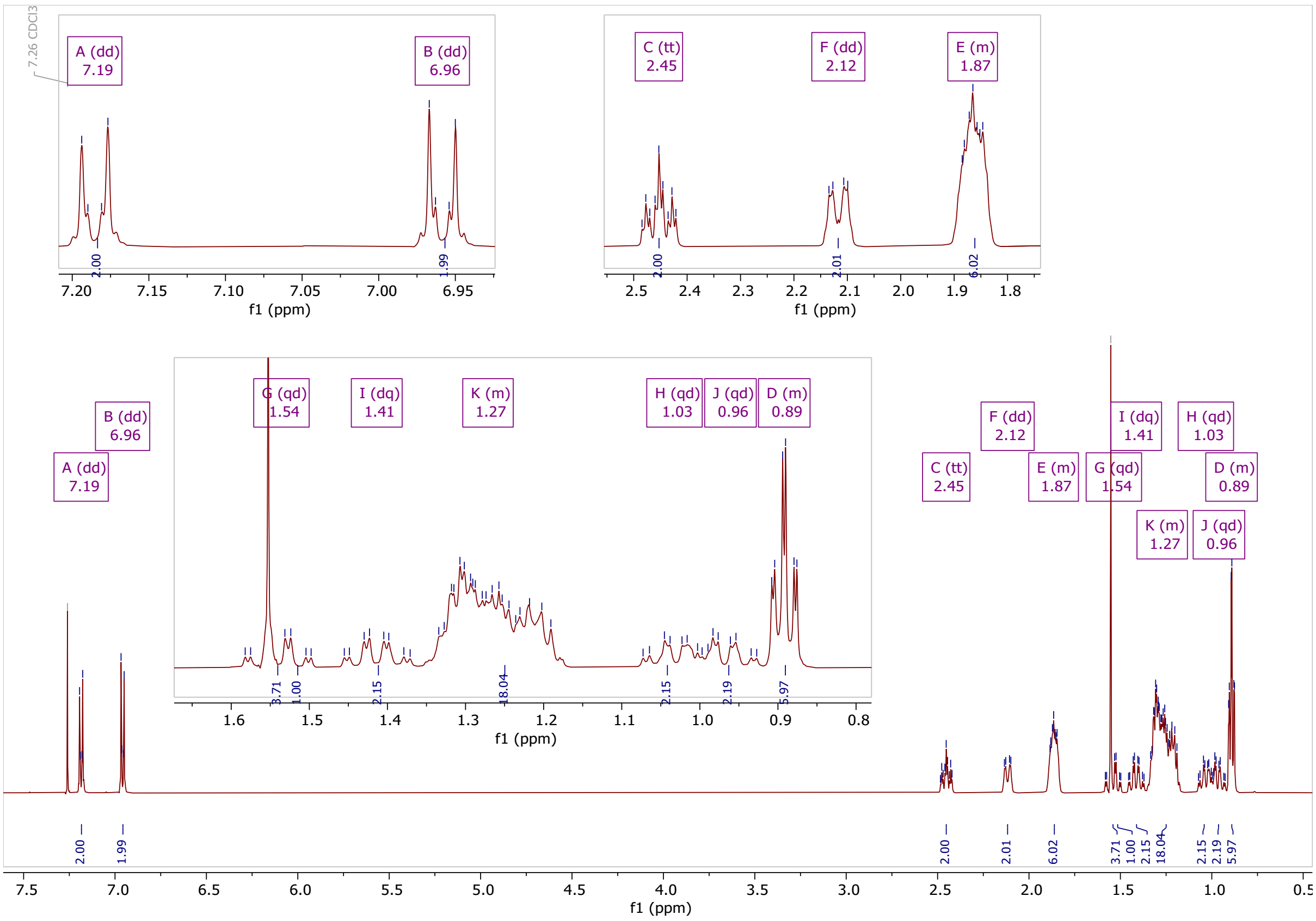

Figure S33 proton NMR spectrum of **6**

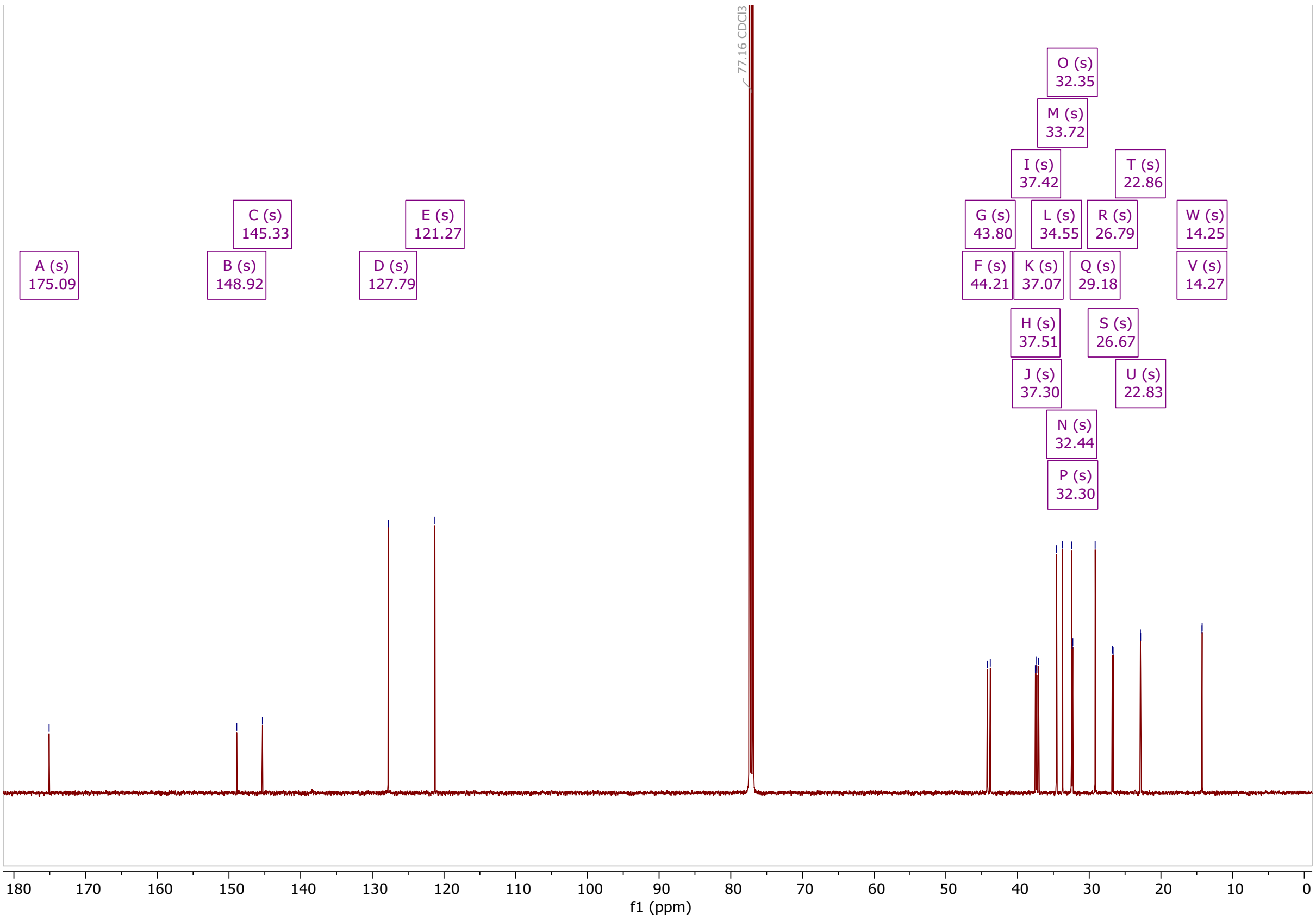

Figure S34 carbon NMR spectrum of **6**

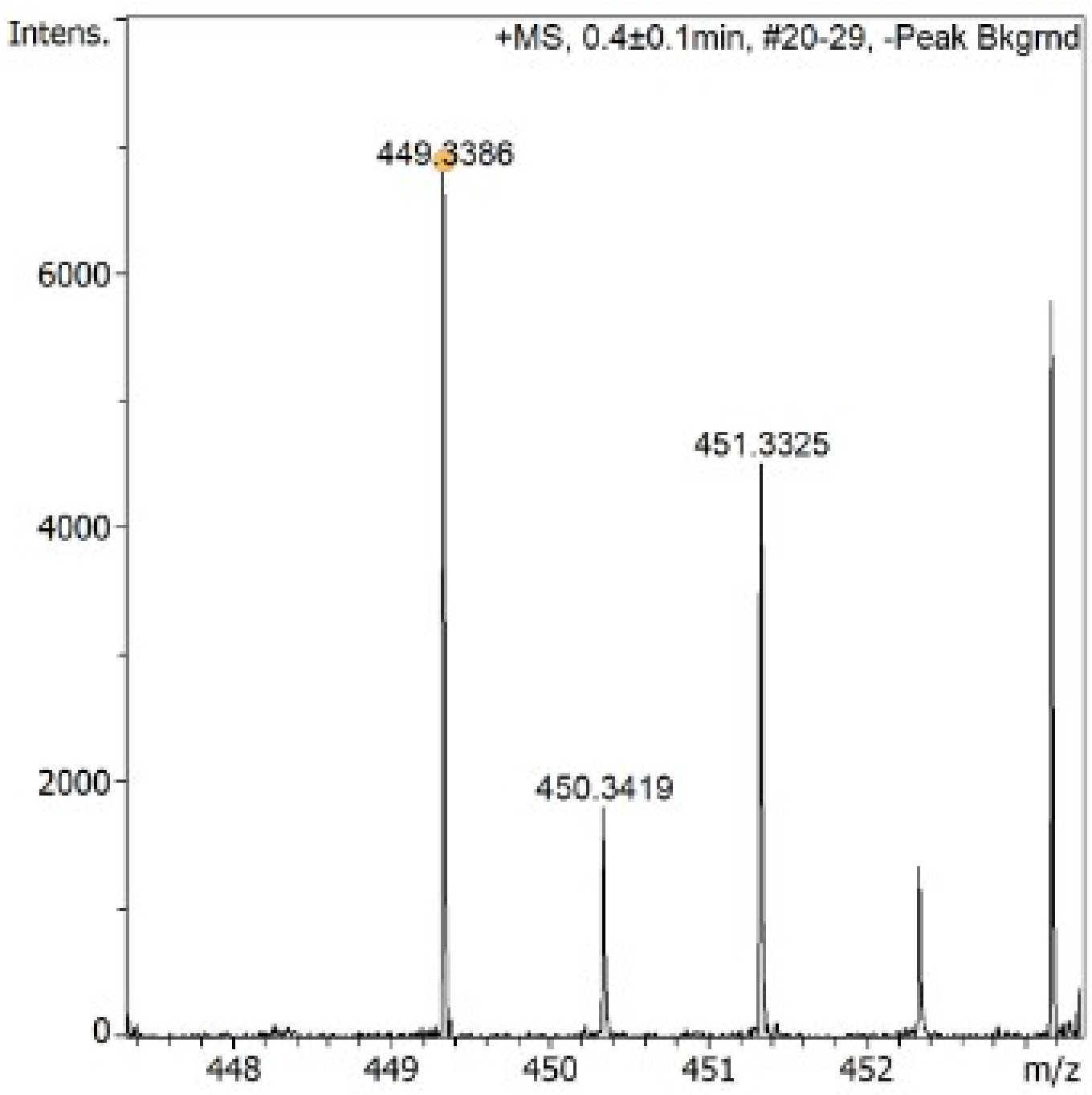

Figure S35 HRMS of **6**

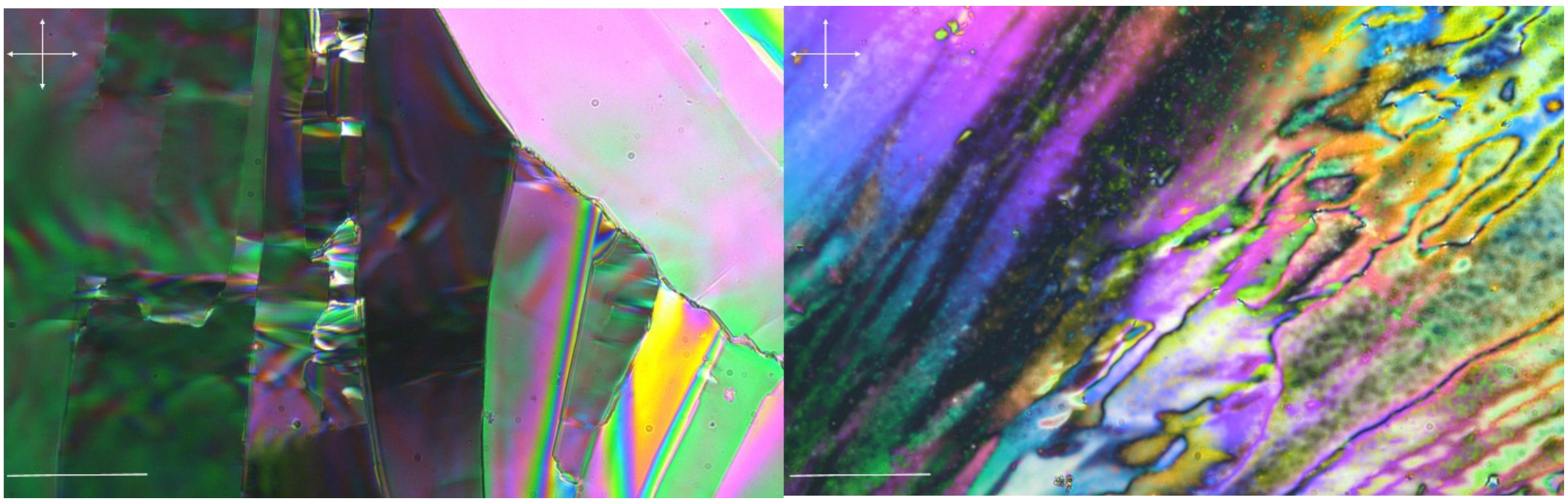

Figure S36 POM images of **7**. Left images SmX at 94 °C. Right imagine N 169 °C. Scale bar (bottom-left) shows 1 µm, arrows show polariser direction.

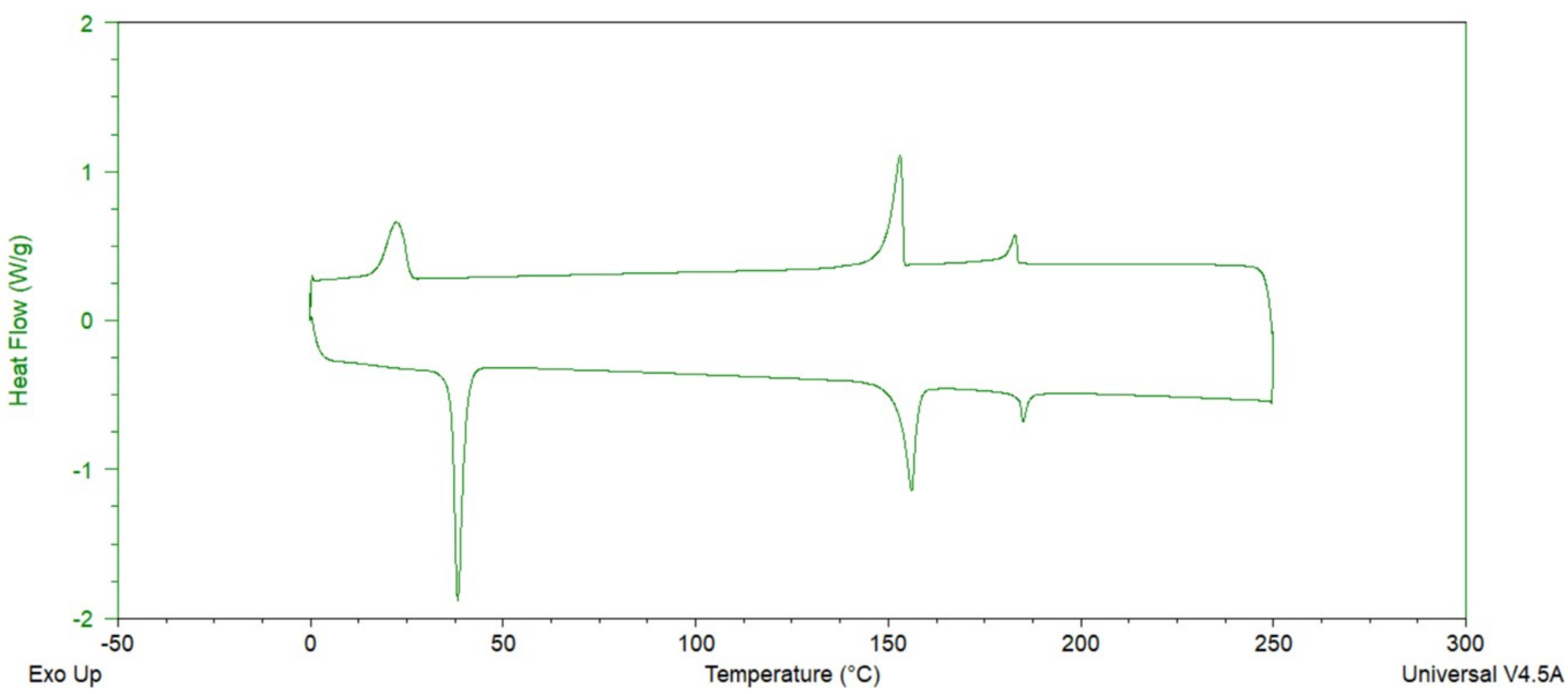

Figure S37 DSC thermogram of **6**.

7 | *4-cyanophenyl 4'-propyl-[1,1'-biphenyl]-4-carboxylate*

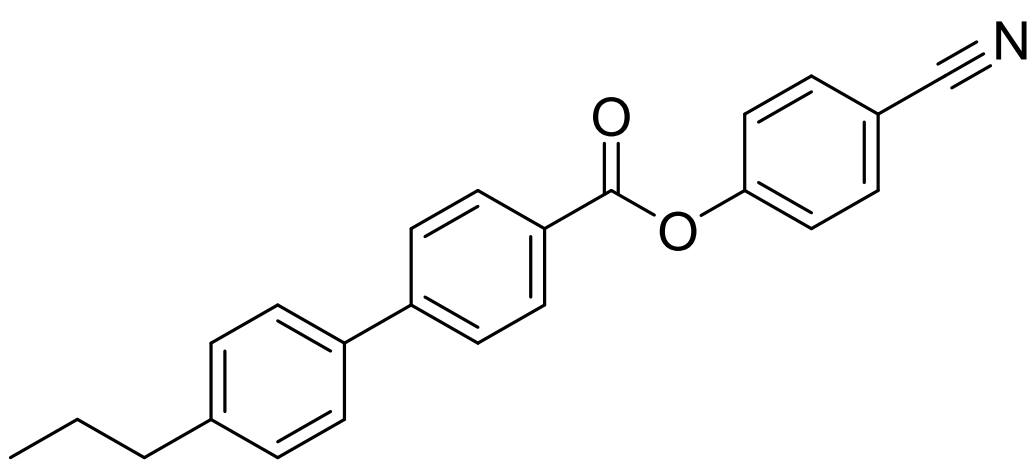

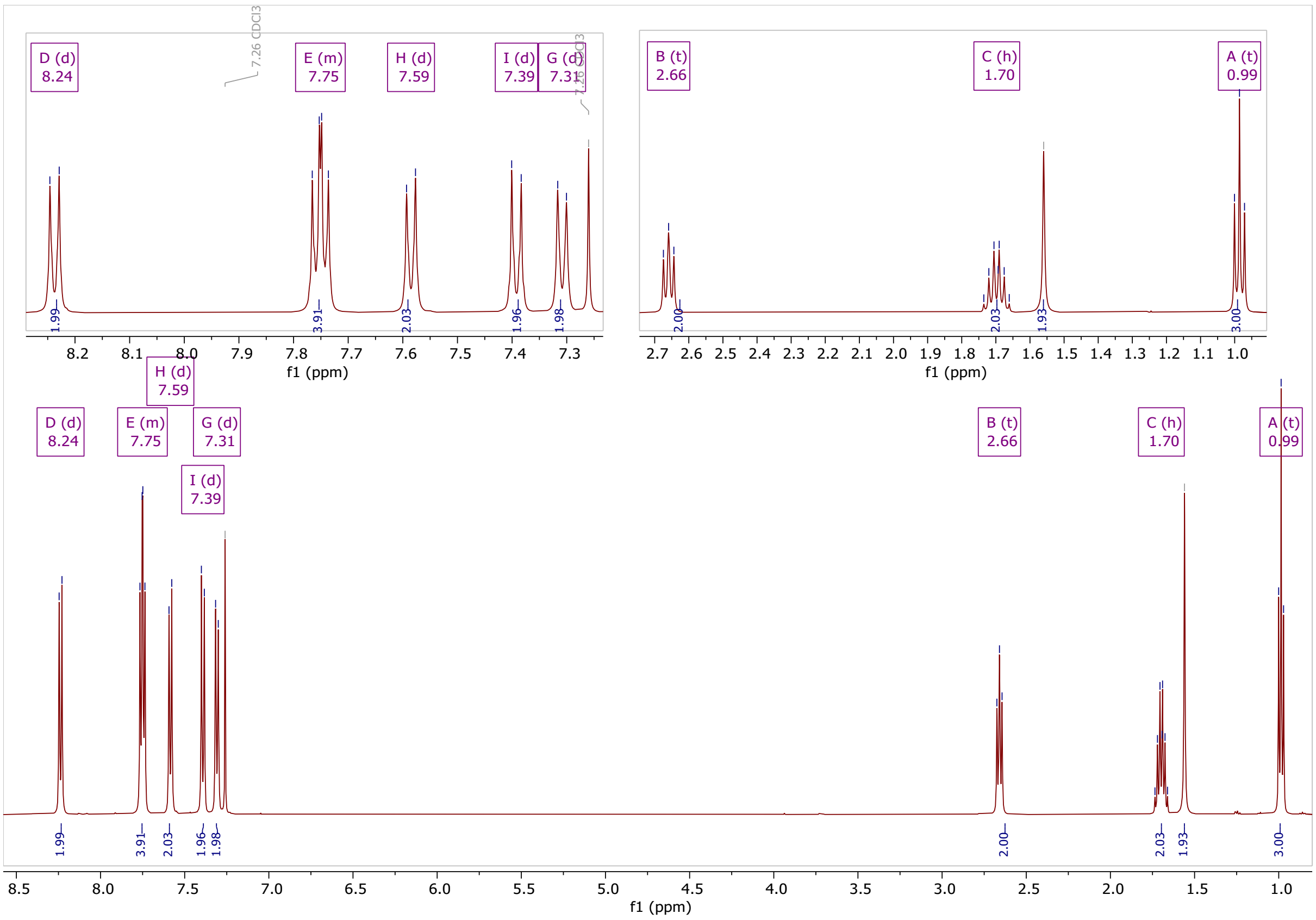

Figure S38 proton NMR spectrum of **7**

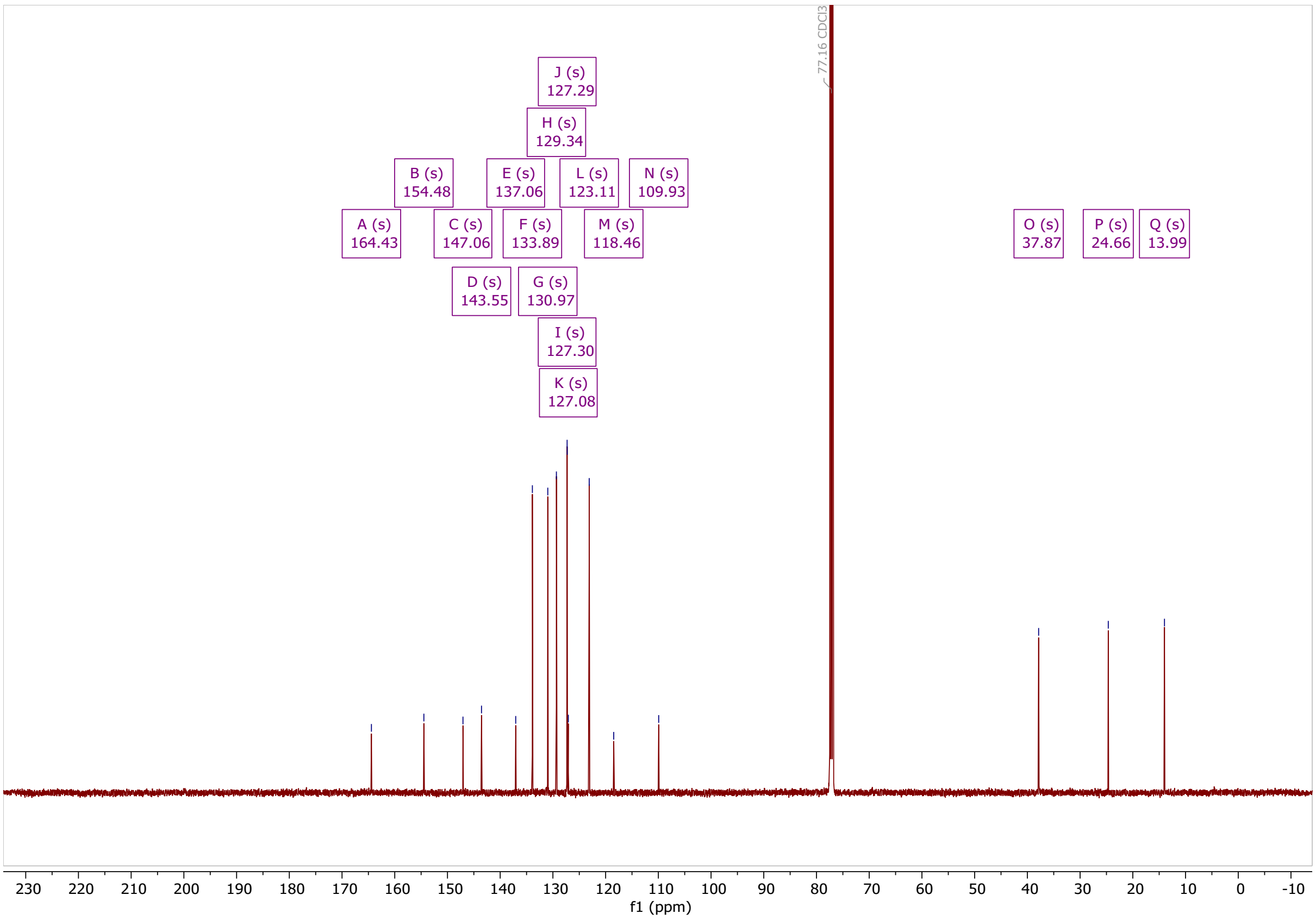

Figure S39 carbon NMR spectrum of **7**

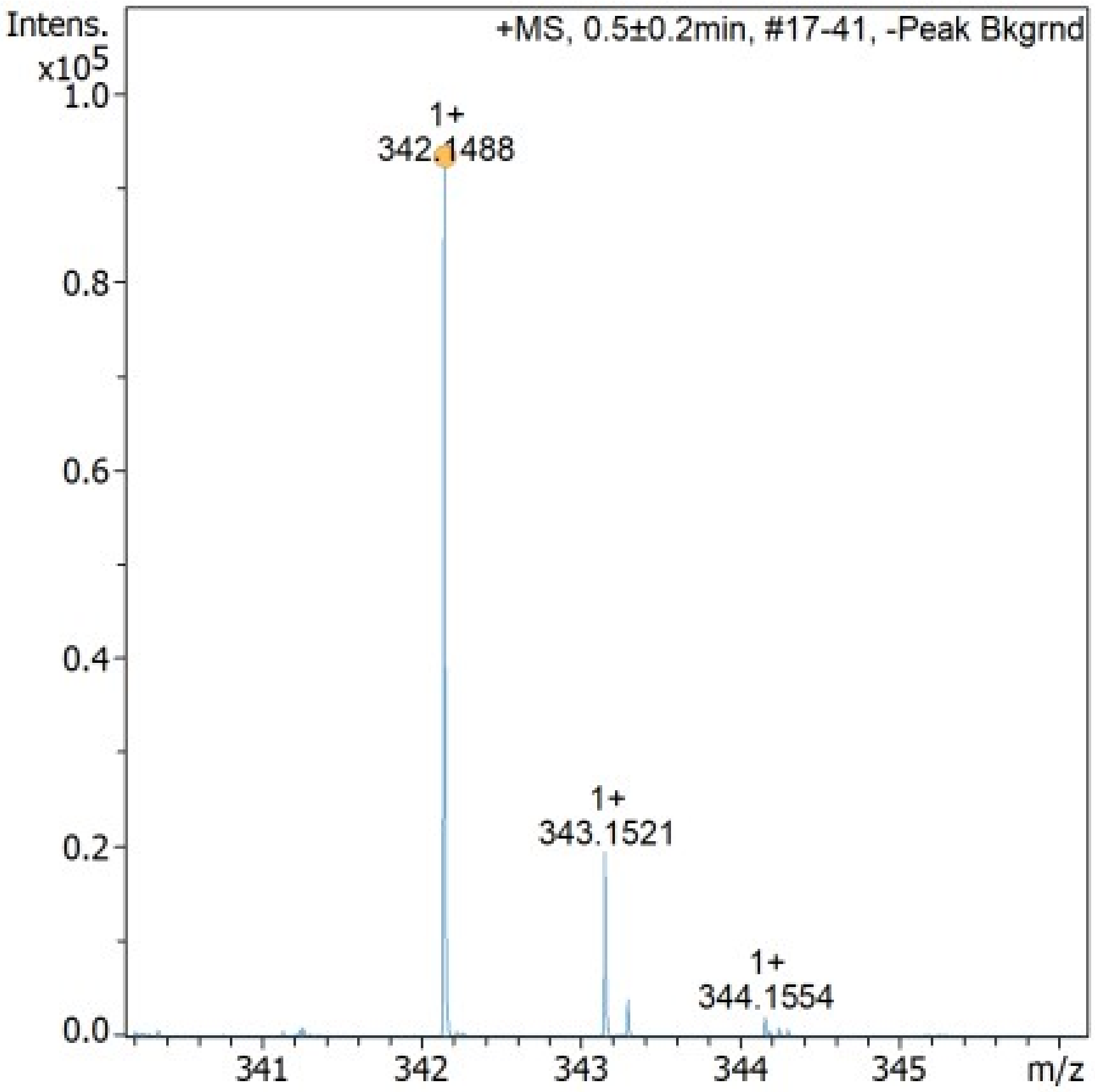

Figure S40 HRMS of **7**

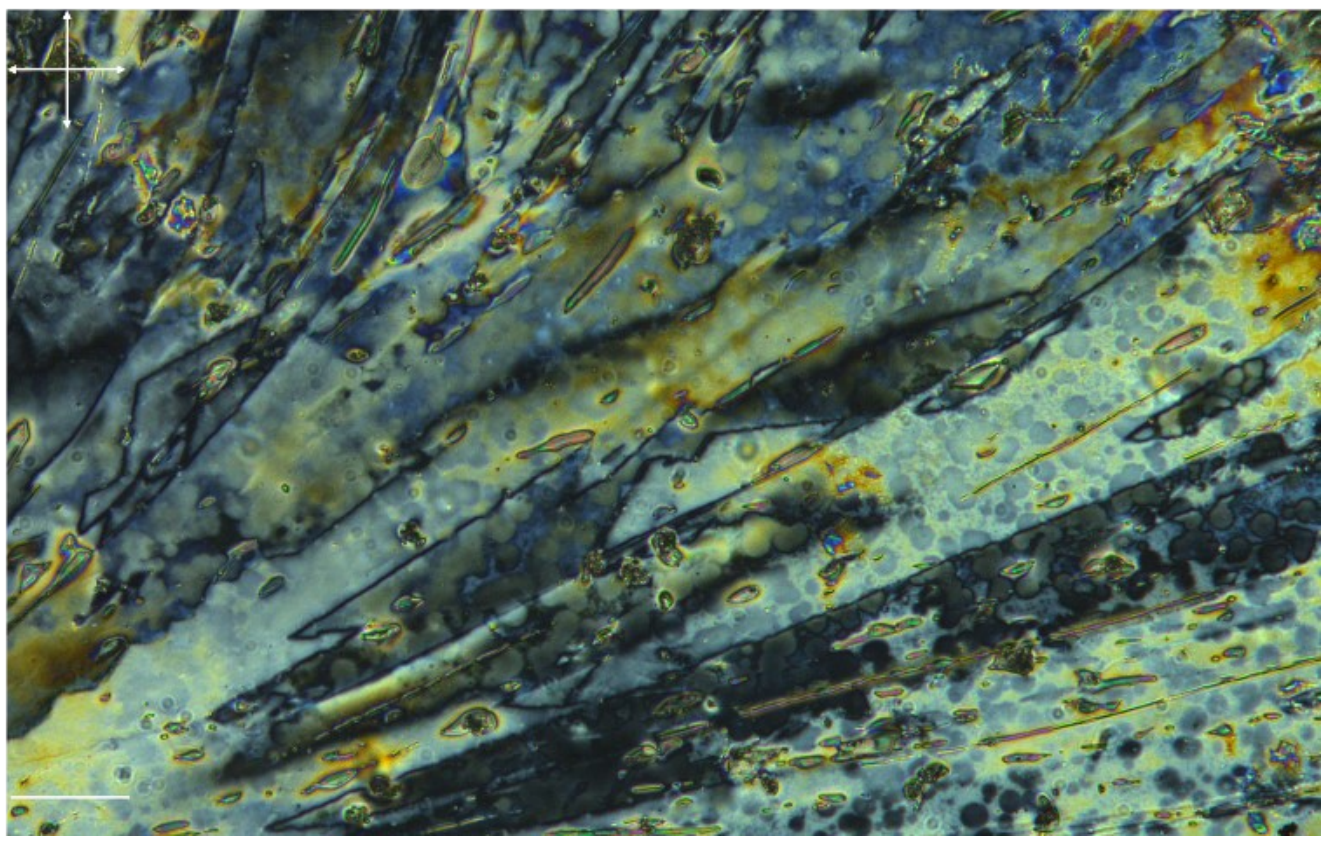

Figure S41 POM image of **7**, N at 140 °C. Scale bar (bottom-left) shows 1 μm, arrows show polariser direction.

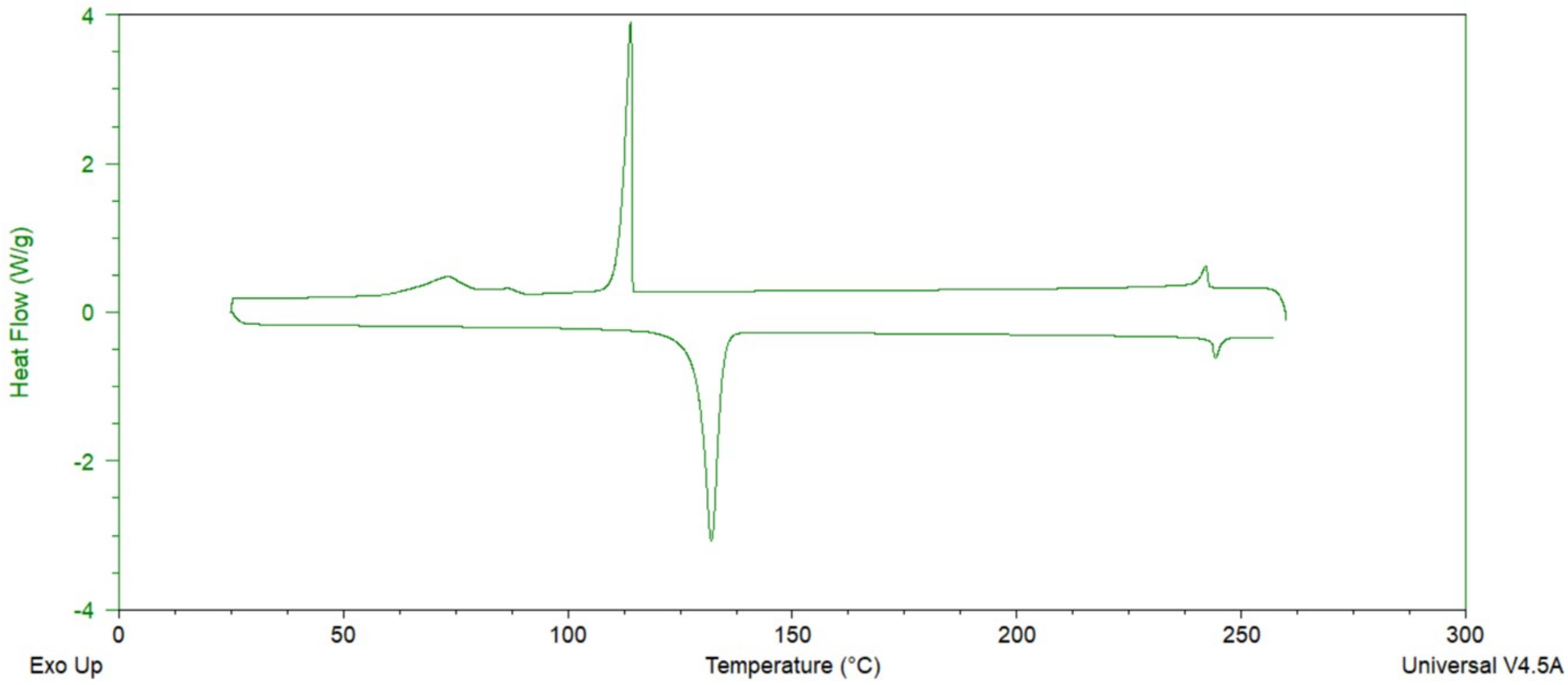

Figure S42 DSC thermogram of **7**.

8 | *4-cyanophenyl 4-((1s,4r)-4-propylcyclohexyl)benzoate*

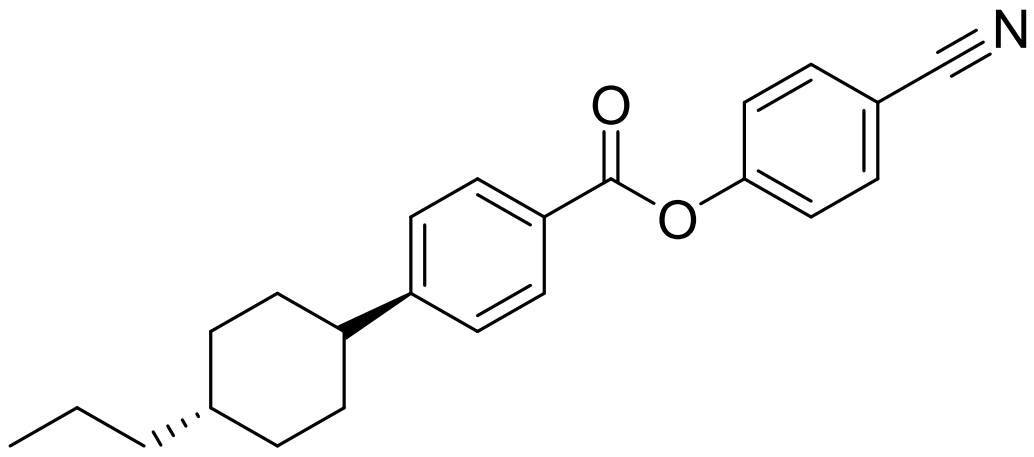

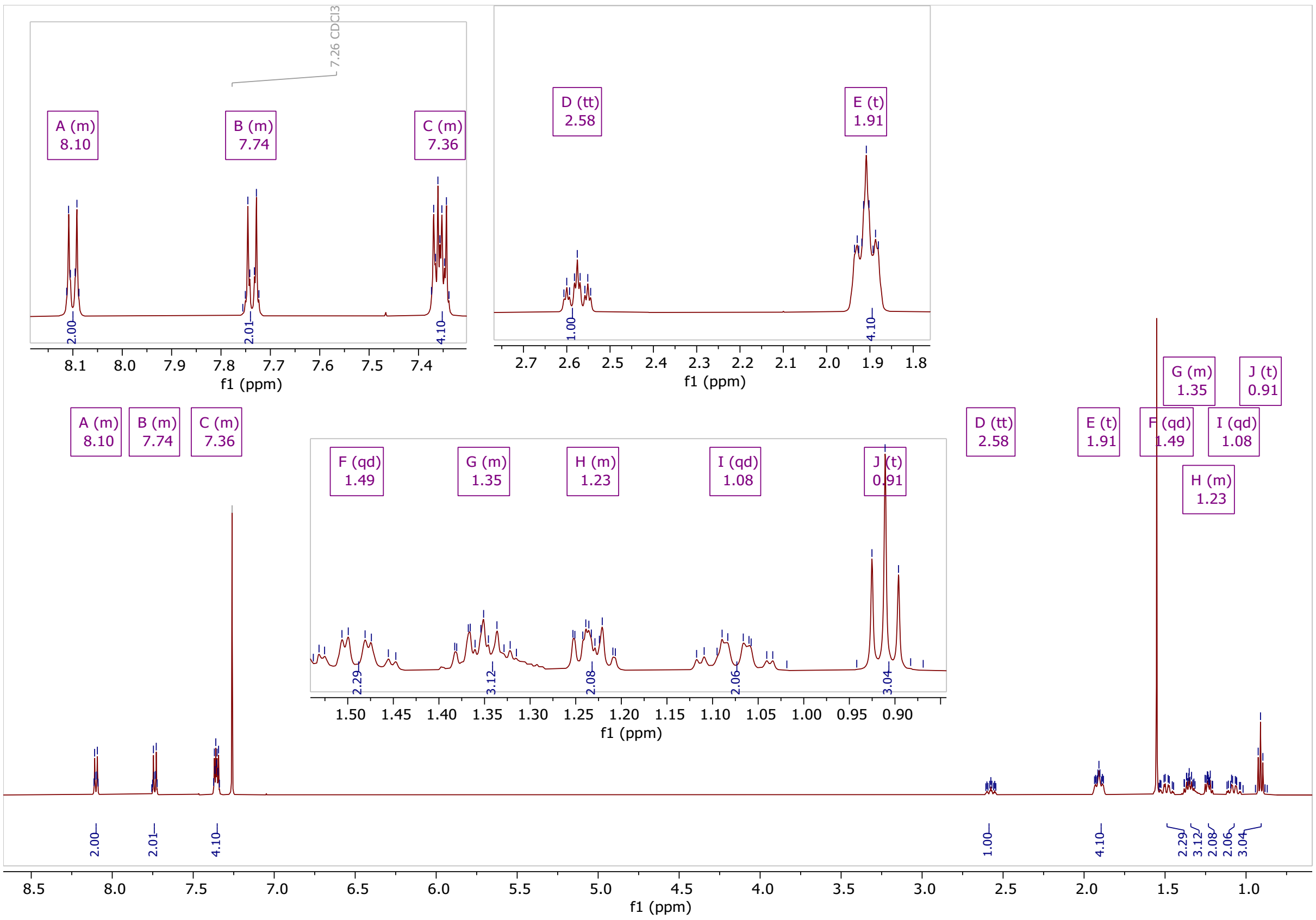

Figure S43 proton NMR spectrum of **8**

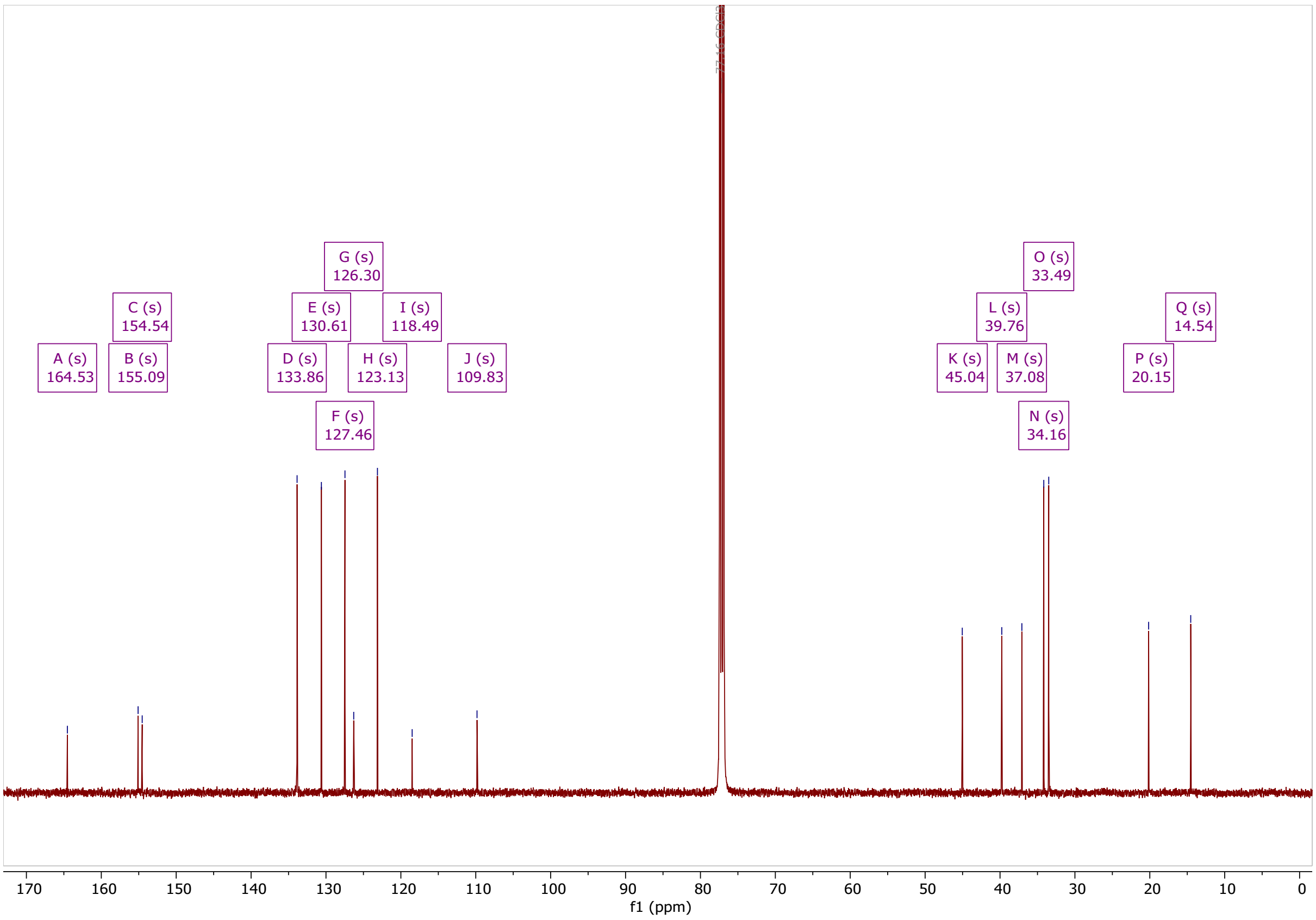

Figure S44 carbon NMR spectrum of **8**

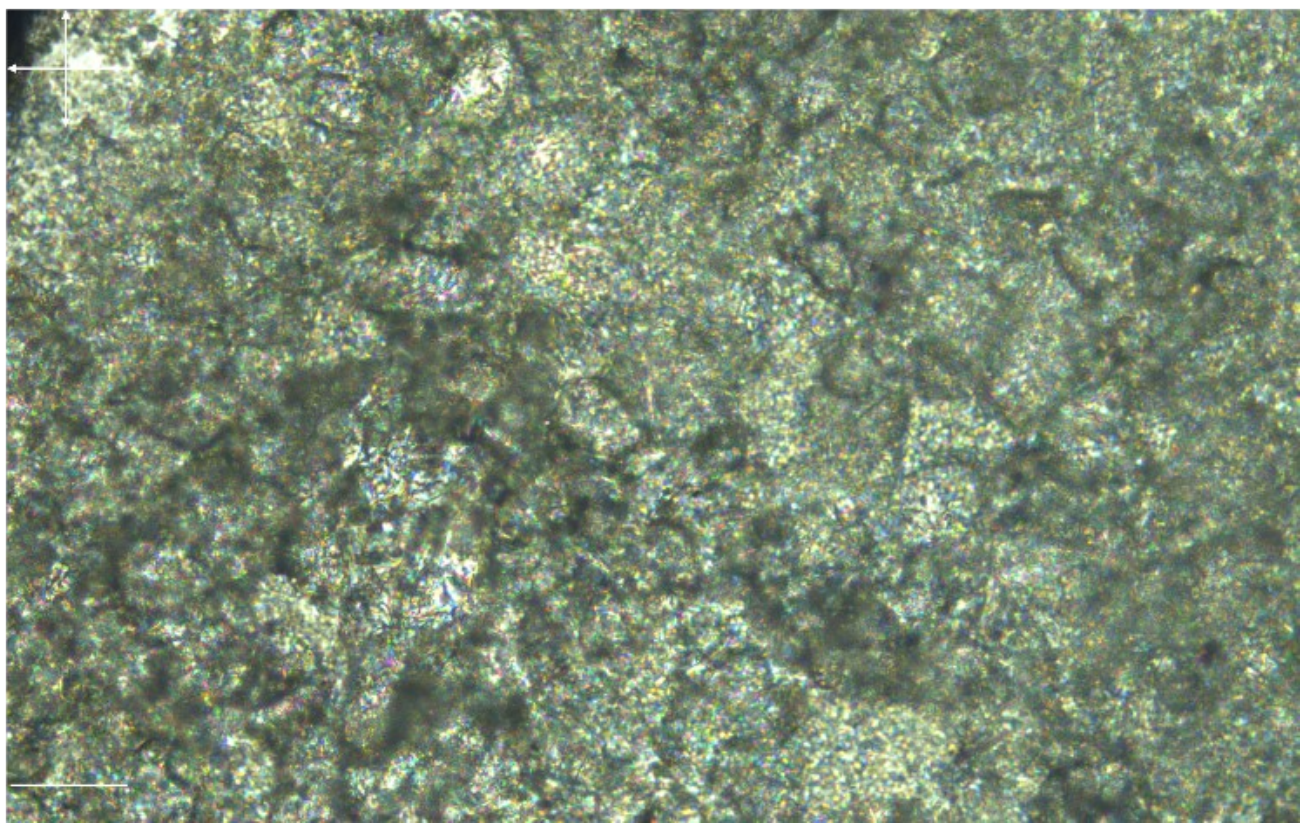

Figure S45 POM image of **8** at 220 °C showing a nematic texture. Scale bar (bottom-left) shows 1 μm, arrows show polariser direction.

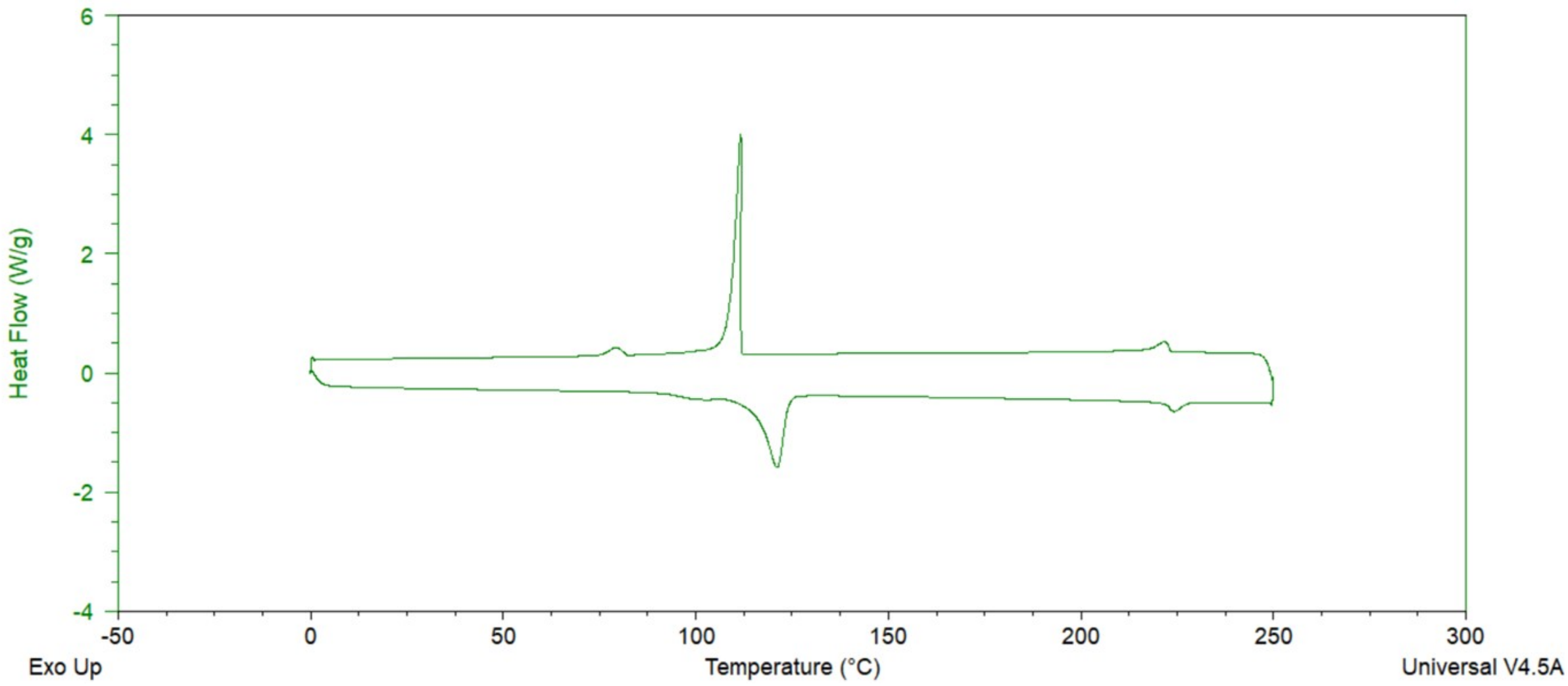

Figure S46 DSC thermogram of **8**.

9 | *4-cyanophenyl 4-pentylbicyclo[2.2.2]octane-1-carboxylate*

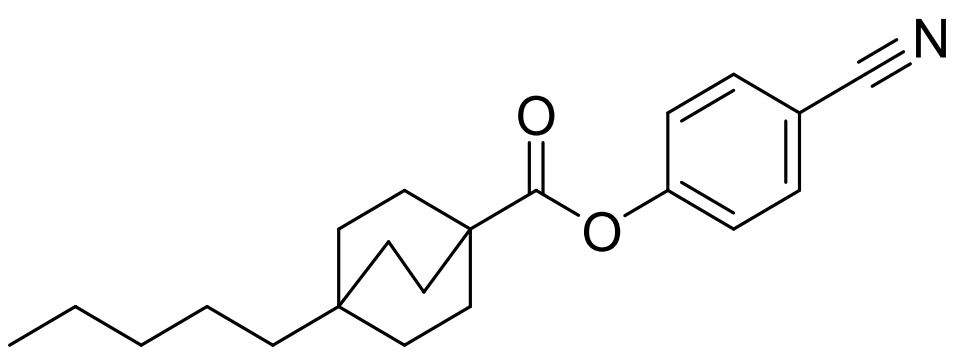

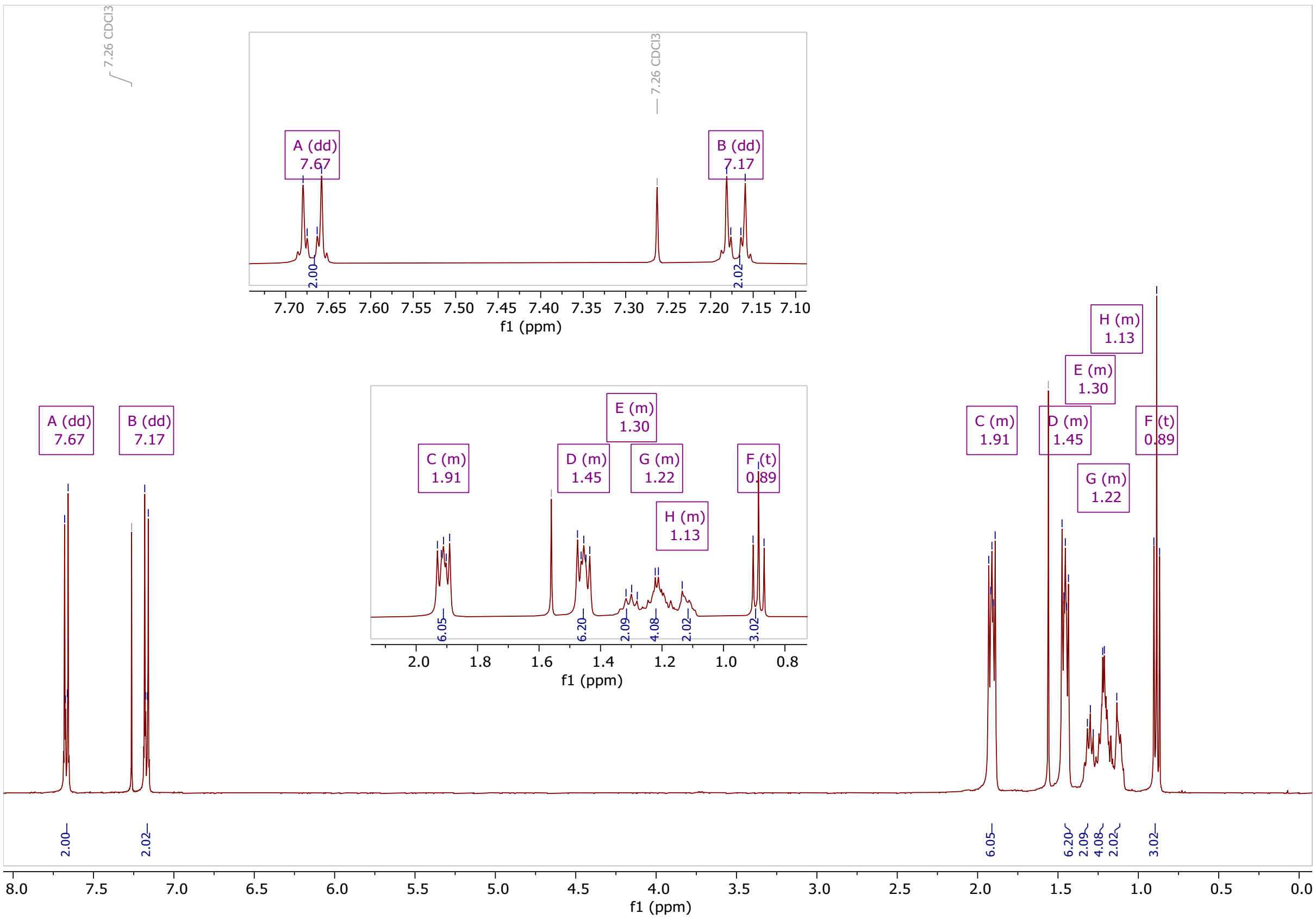

Figure S47 proton NMR spectrum of **9**

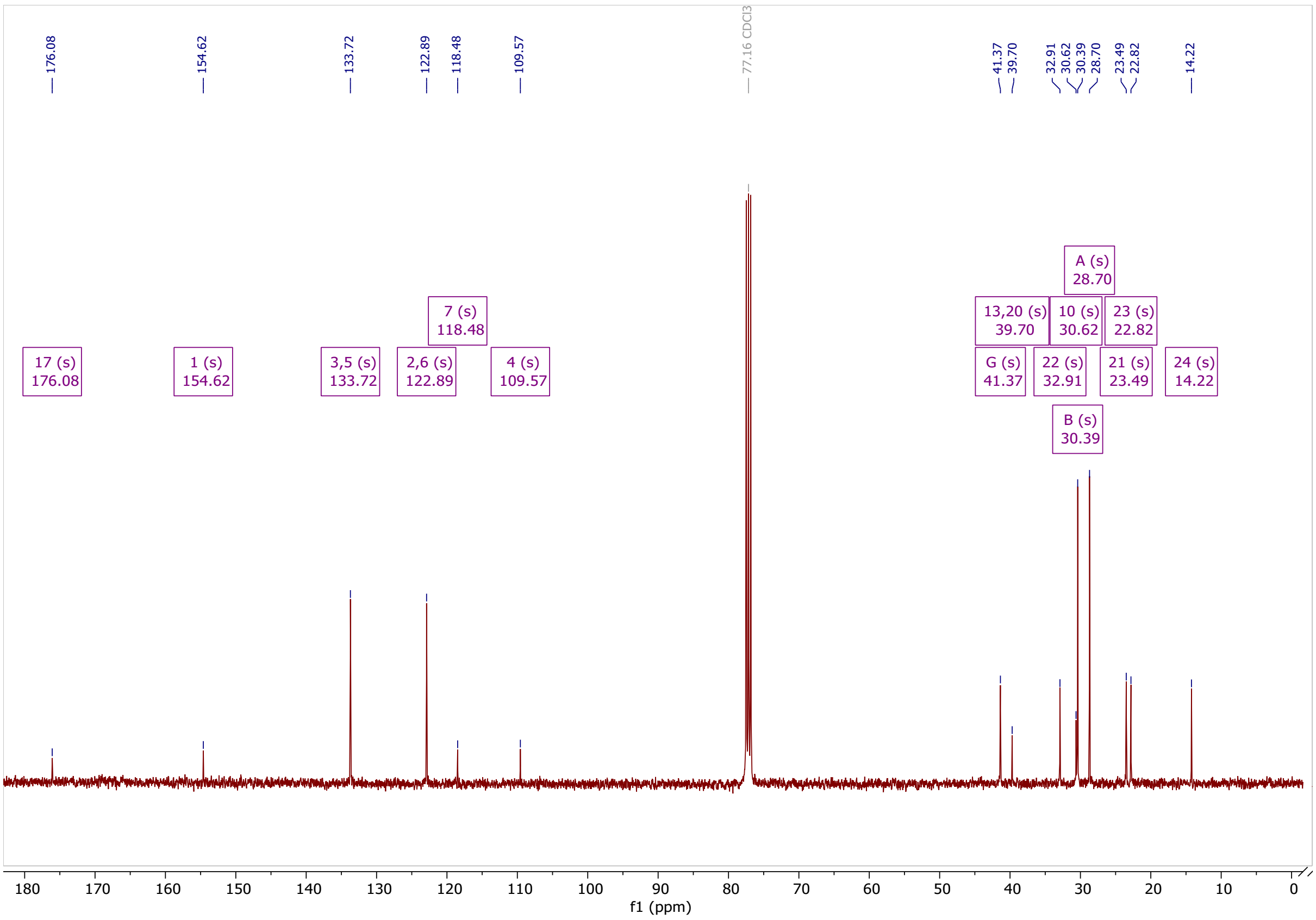

Figure S48 carbon NMR spectrum of **9**

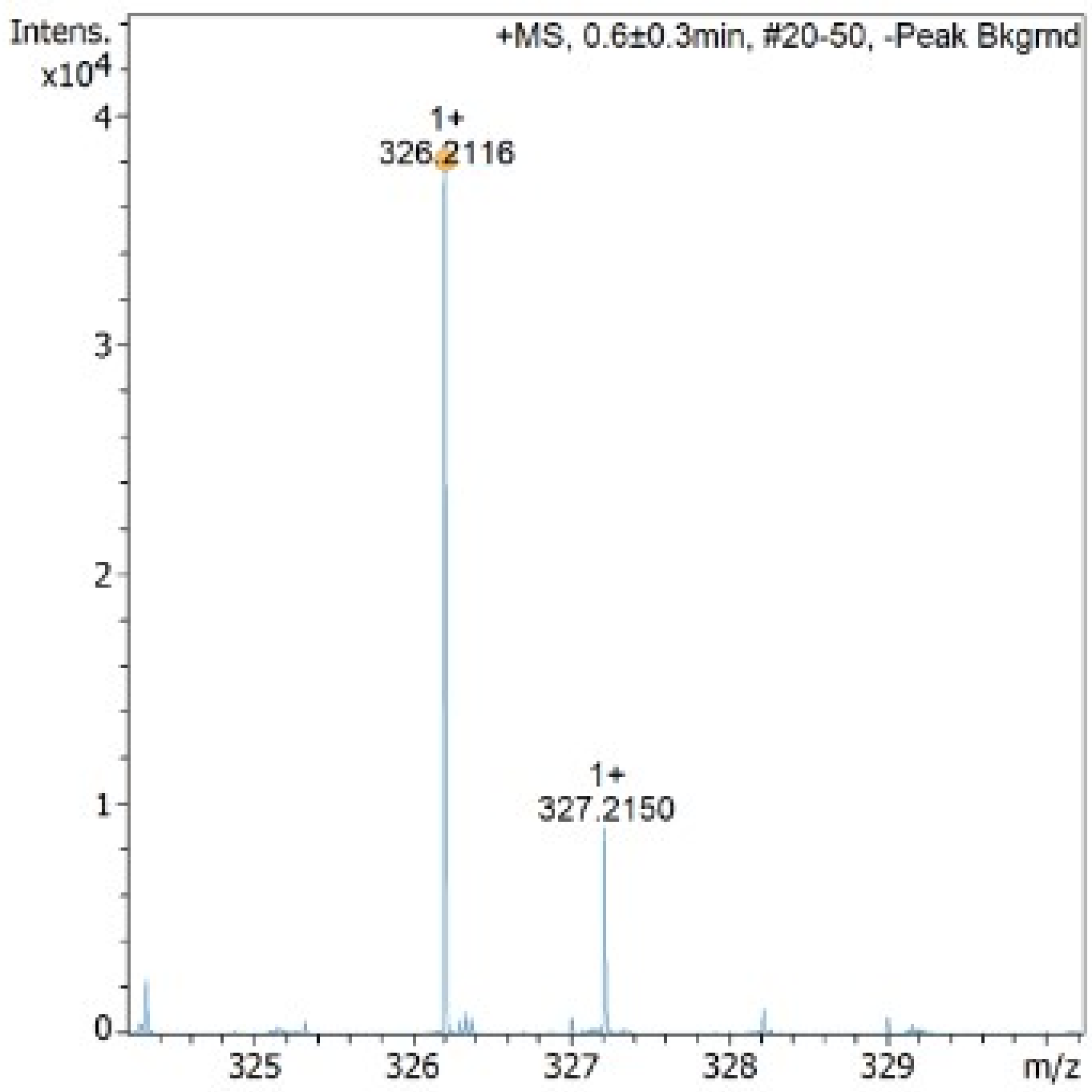

Figure S49 HRMS of **9**

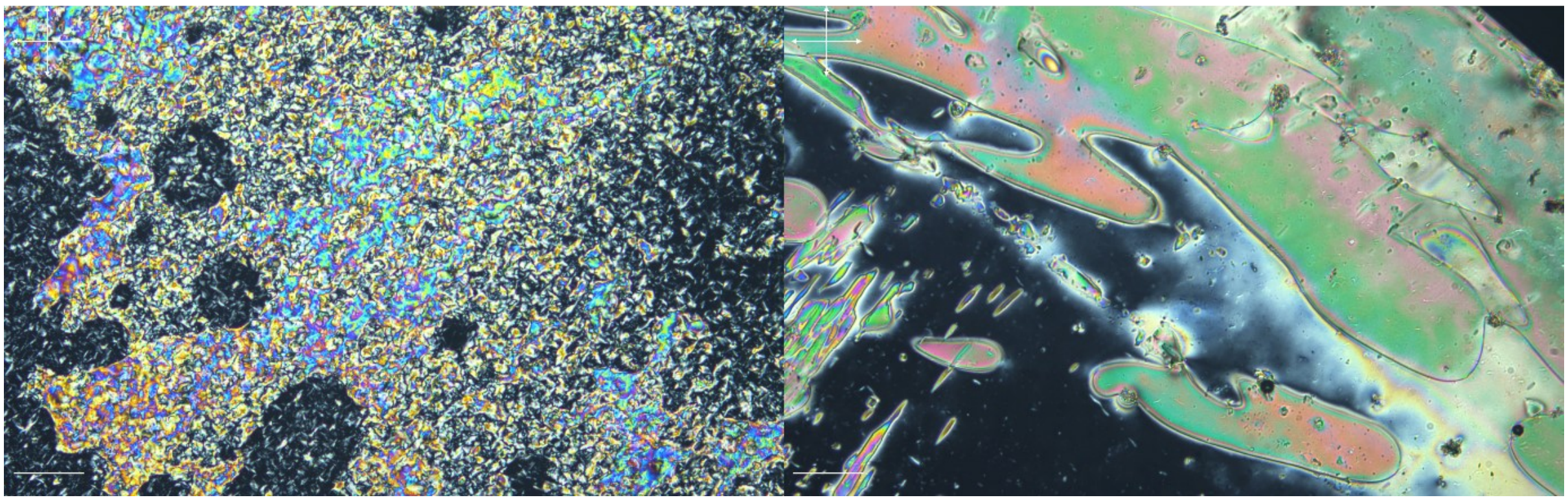

Figure S50 POM image of **9**, both showing nematic textures left at 86 °C and right at 93 °C. Scale bar (bottom-left) shows 1 µm, arrows show polariser direction.

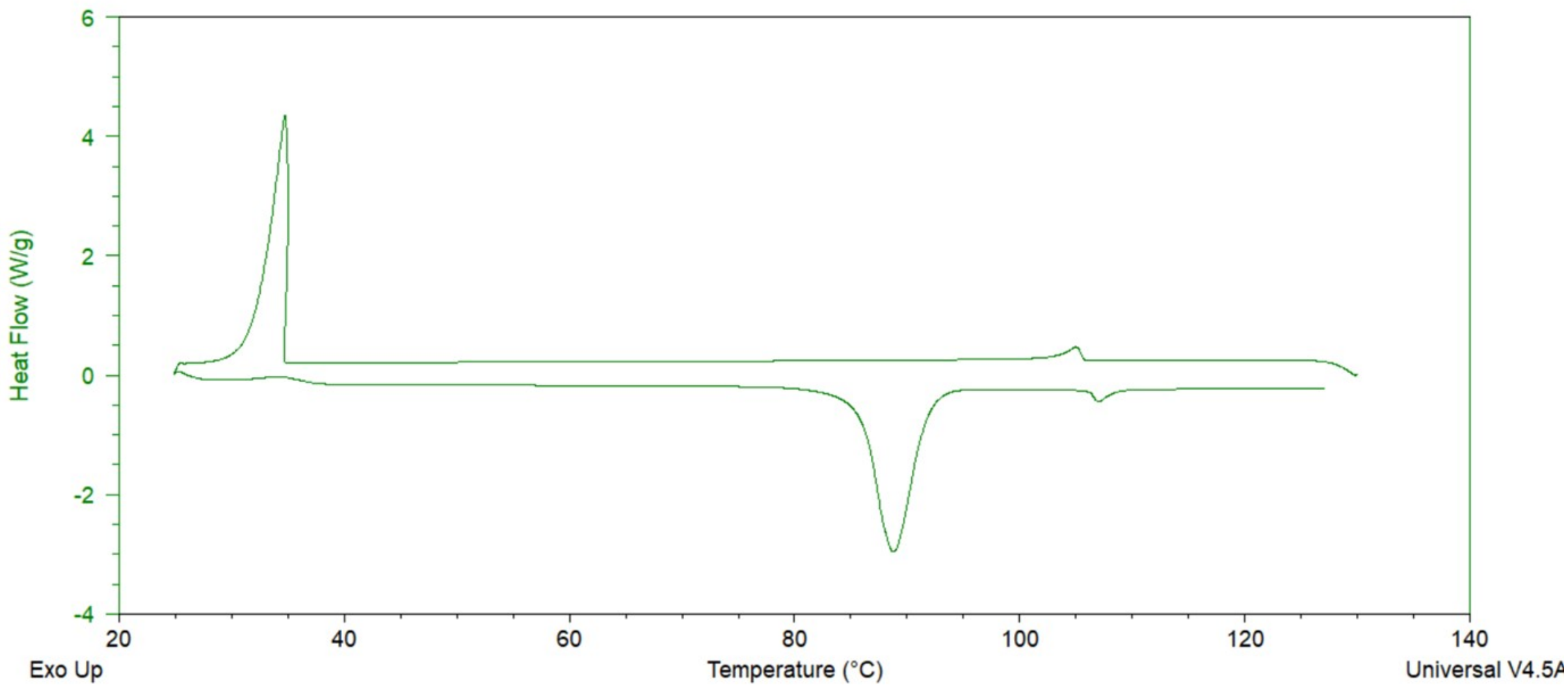

Figure S51 DSC thermogram of **9**.

10 | *4-cyanophenyl (1s,1'r,4S,4'S)-4'-butyl-[1,1'-bi(cyclohexane)]-4-carboxylate*

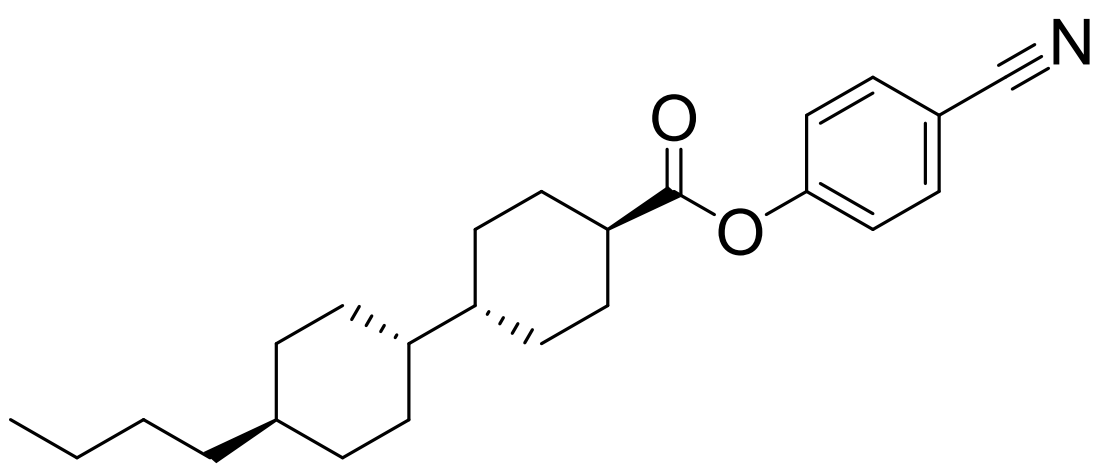

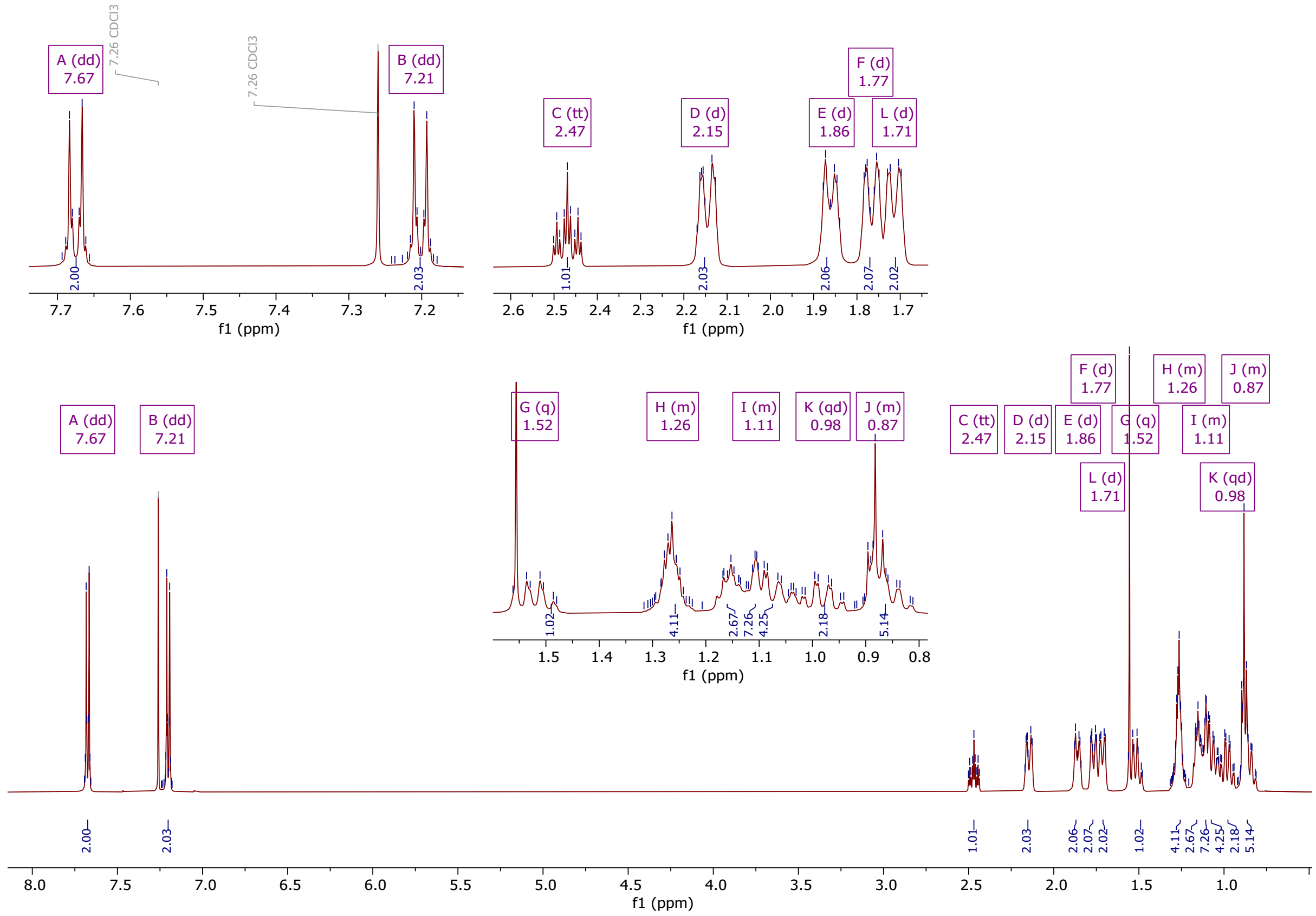

Figure S52 proton NMR spectrum of **10**

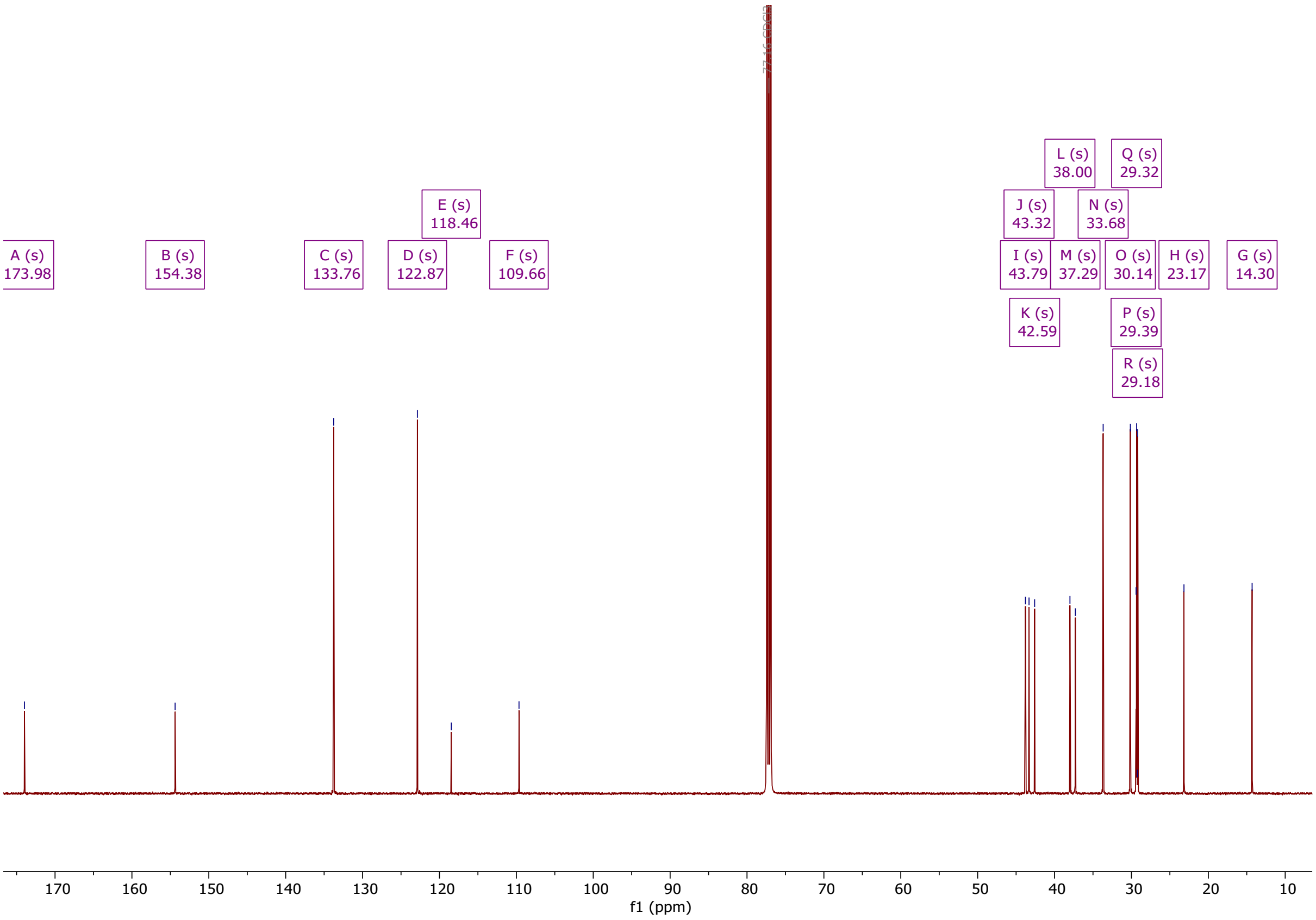

Figure S53 carbon NMR spectrum of **10**

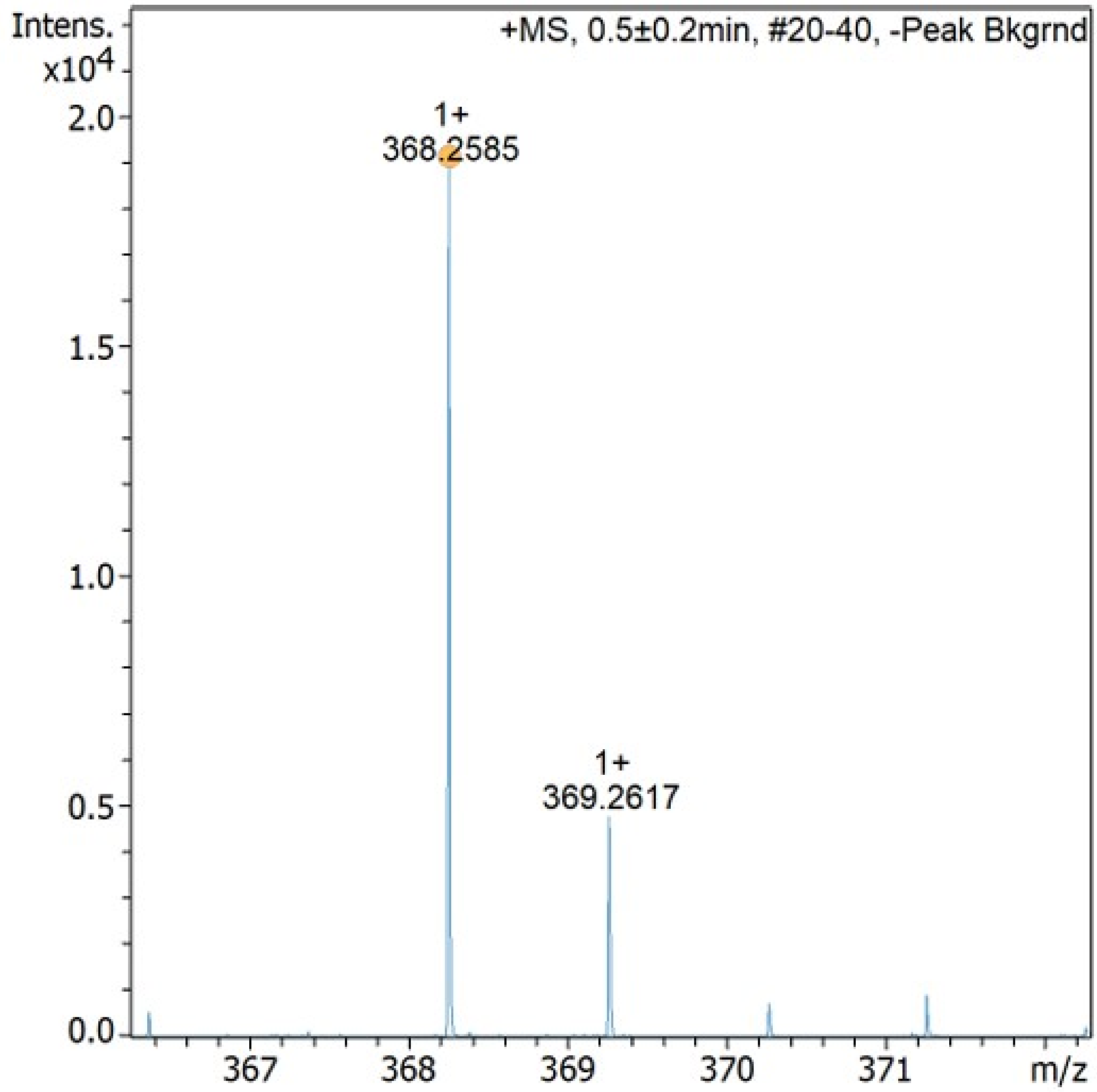

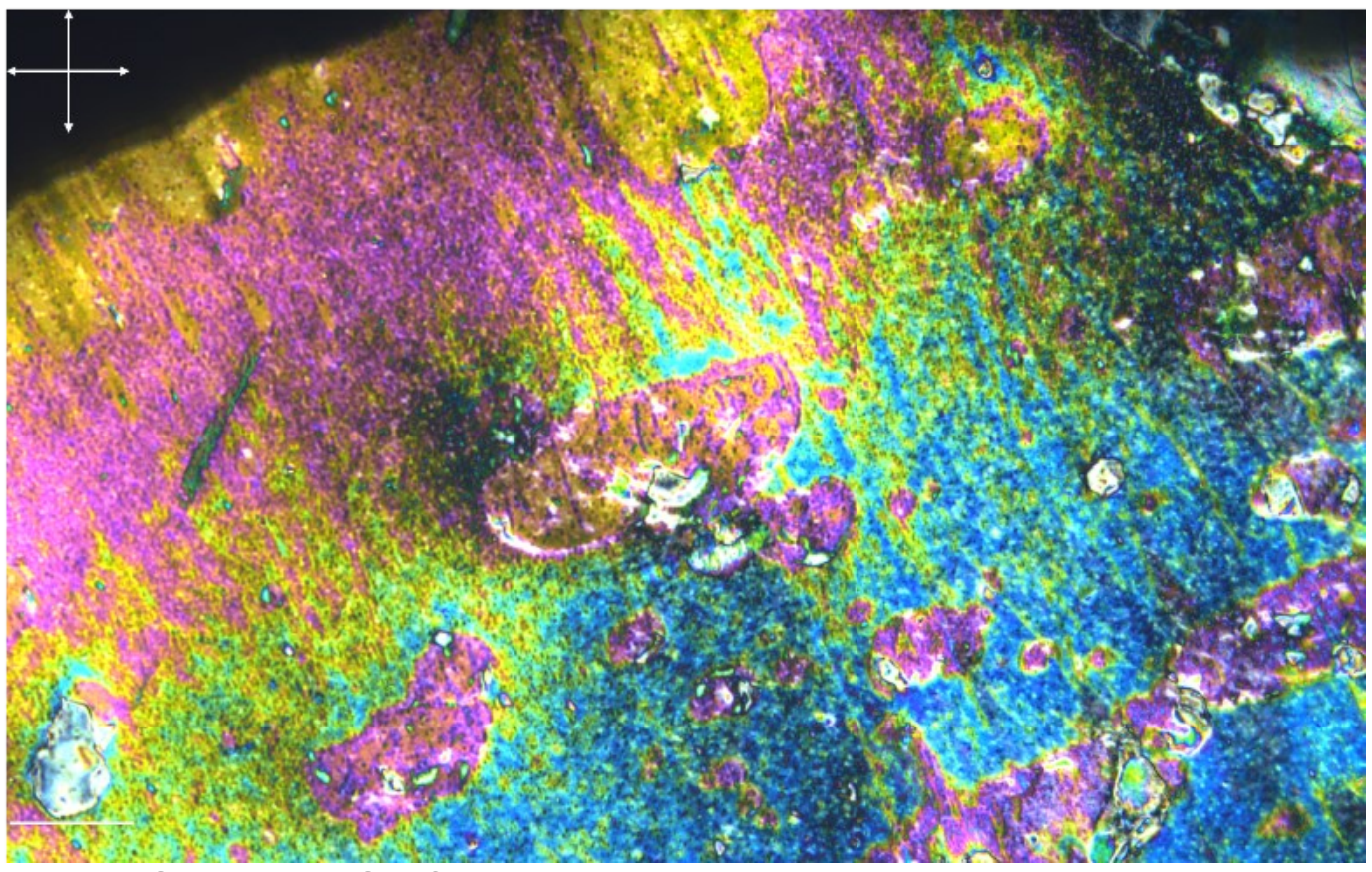
Figure S54 HRMS of **10**

Figure S55 POM of **10**, nematic phase at 160 °C. Scale bar (bottom-left) shows 1 µm, arrows show polariser direction.

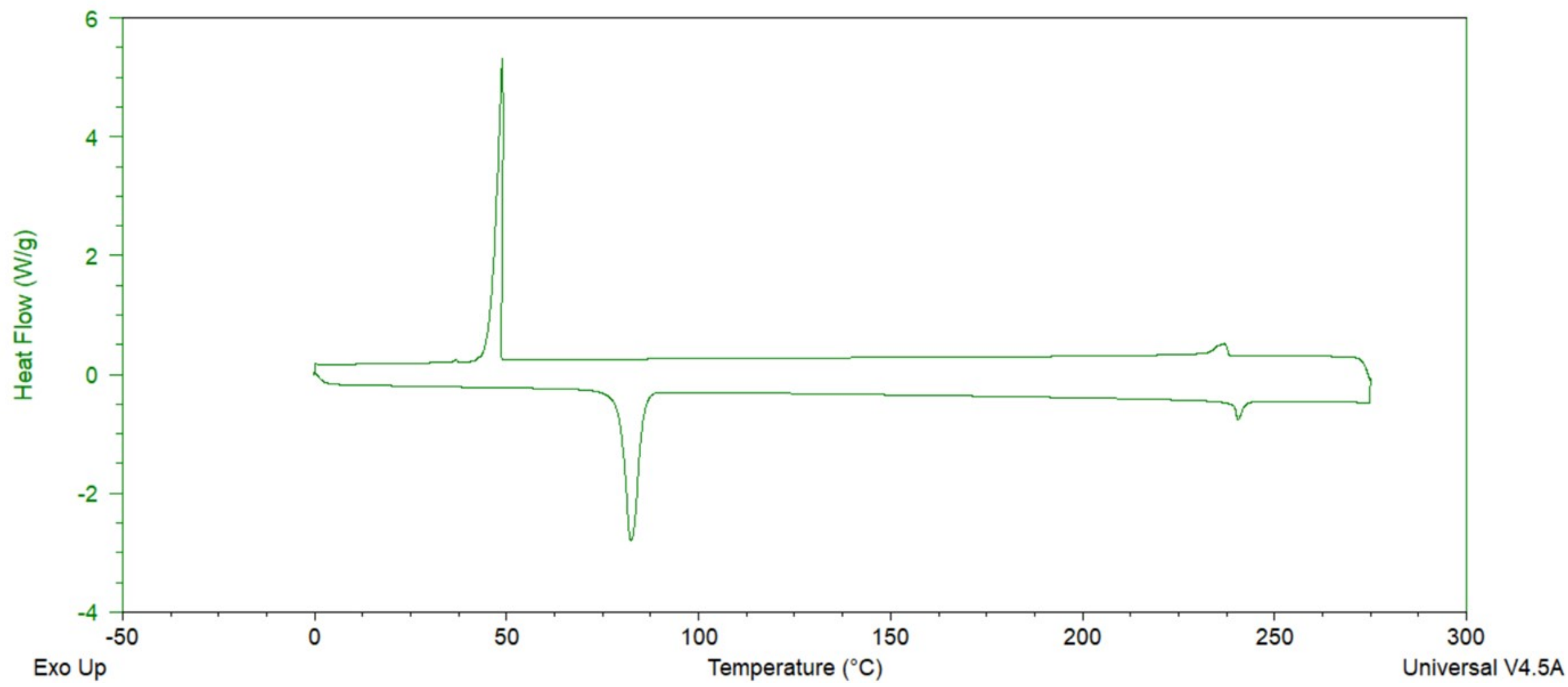

Figure S56 DSC thermogram of **10**.

11 | *4-cyanobenzyl (1r,4r)-4-ethylcyclohexane-1-carboxylate*

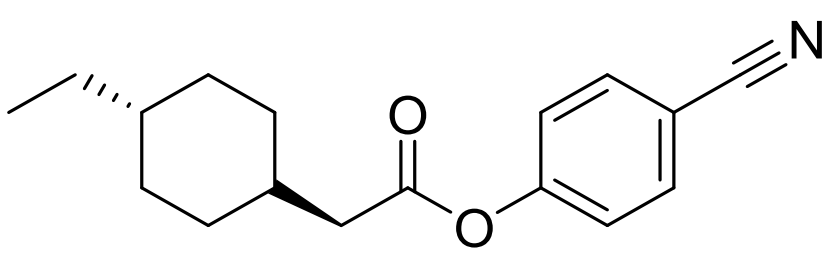

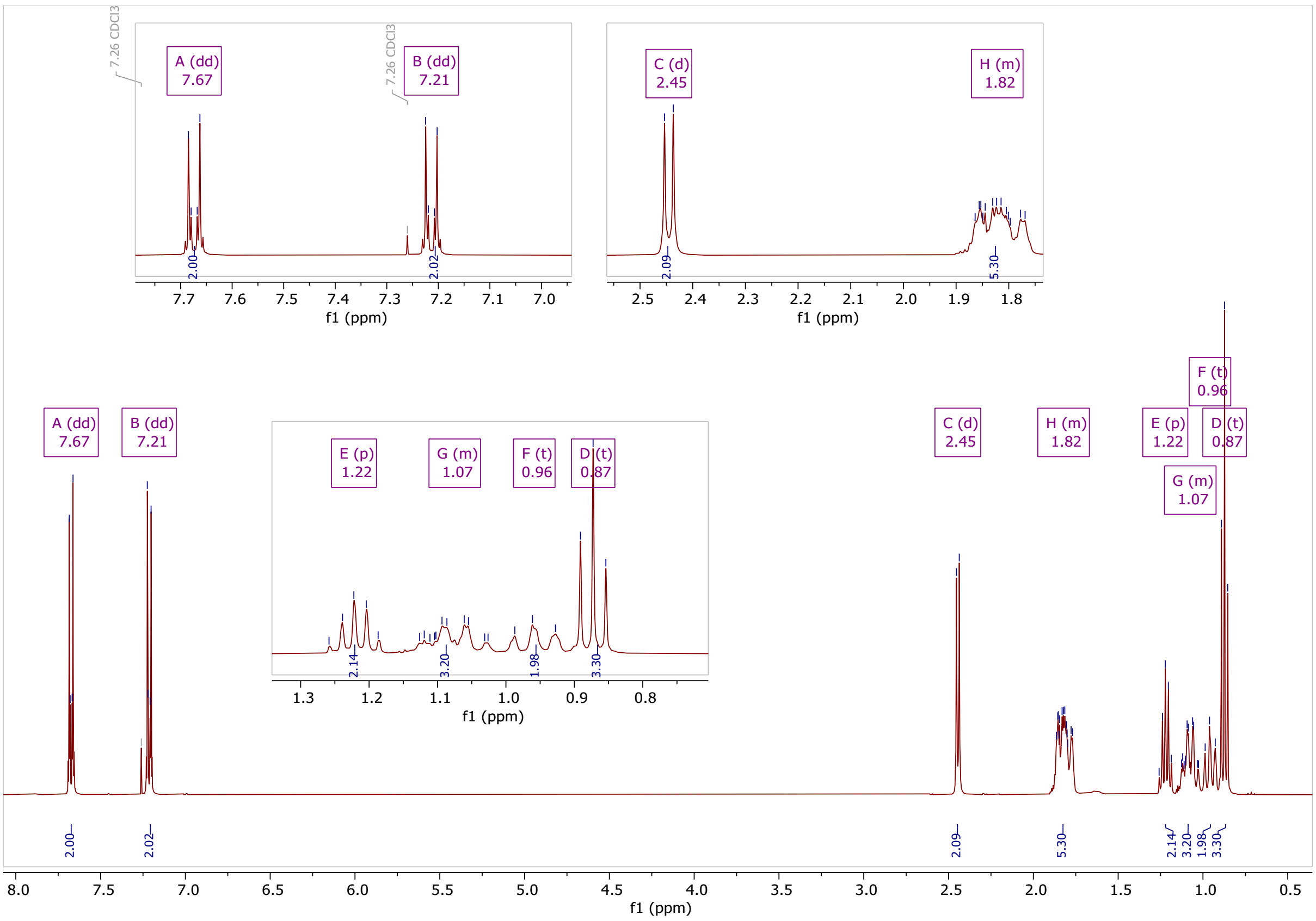

Figure S57 proton NMR spectrum of **11**

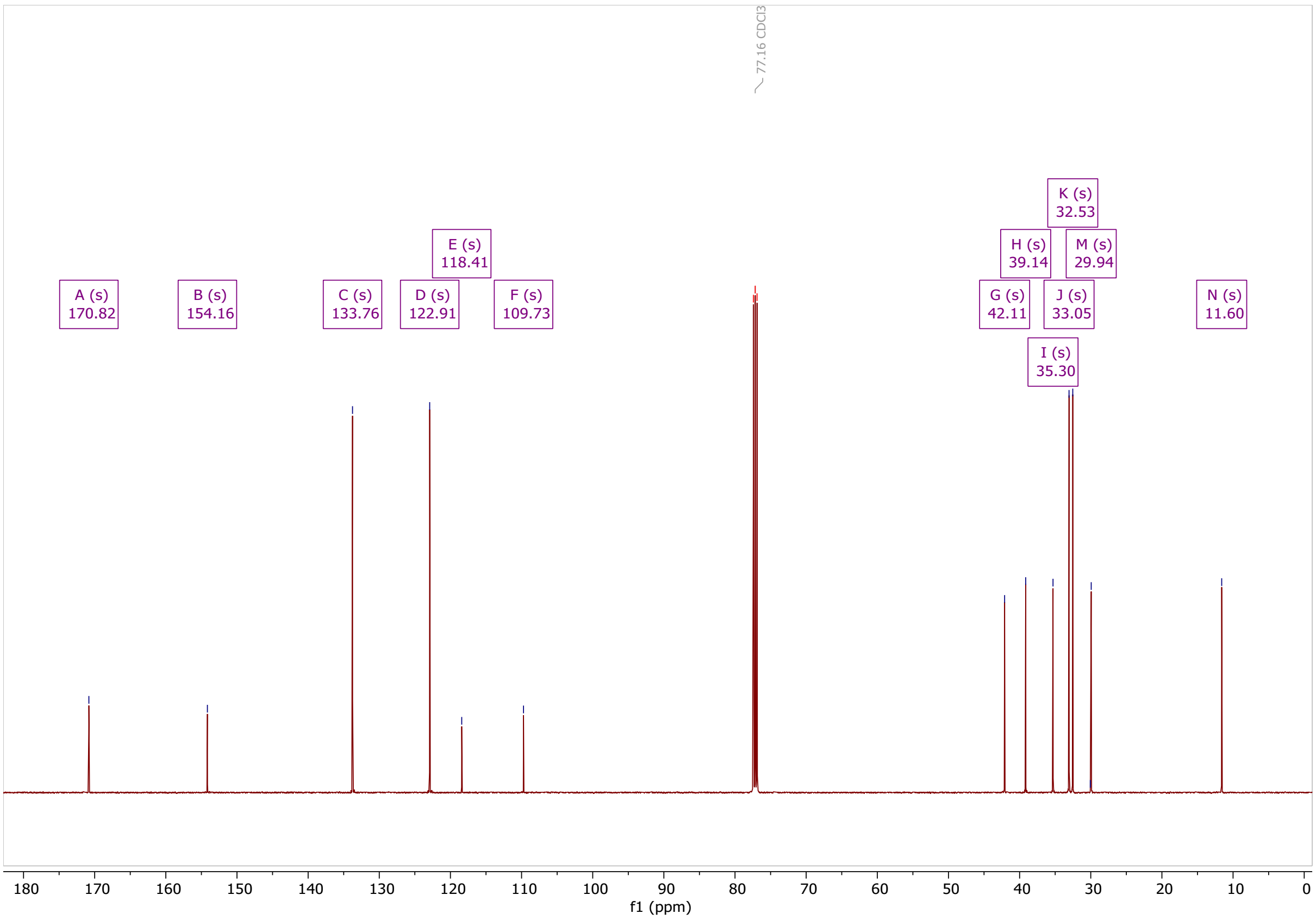

Figure S58 carbon NMR spectrum of **11**.

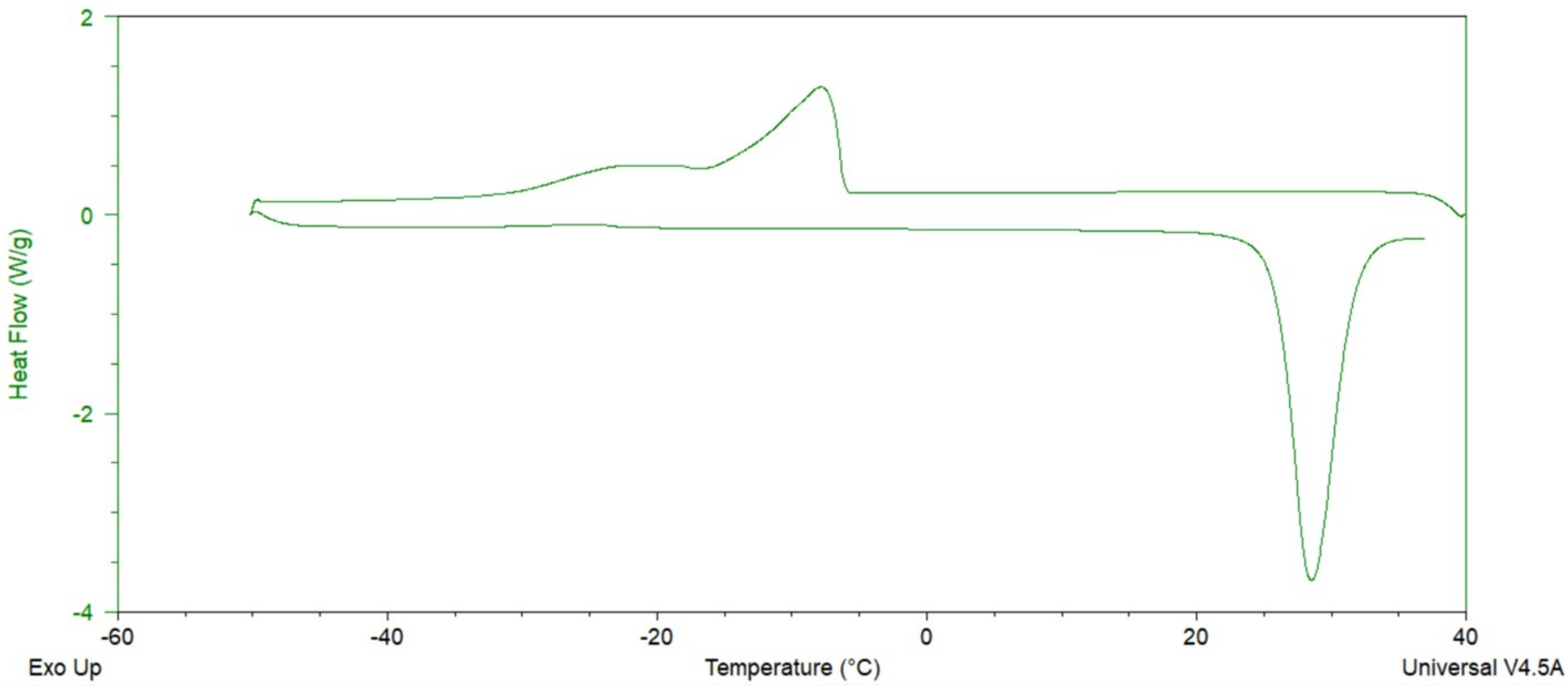

Figure S59 DSC thermogram of **11**.

12 | *4'-cyanophenyl cholesteroate*

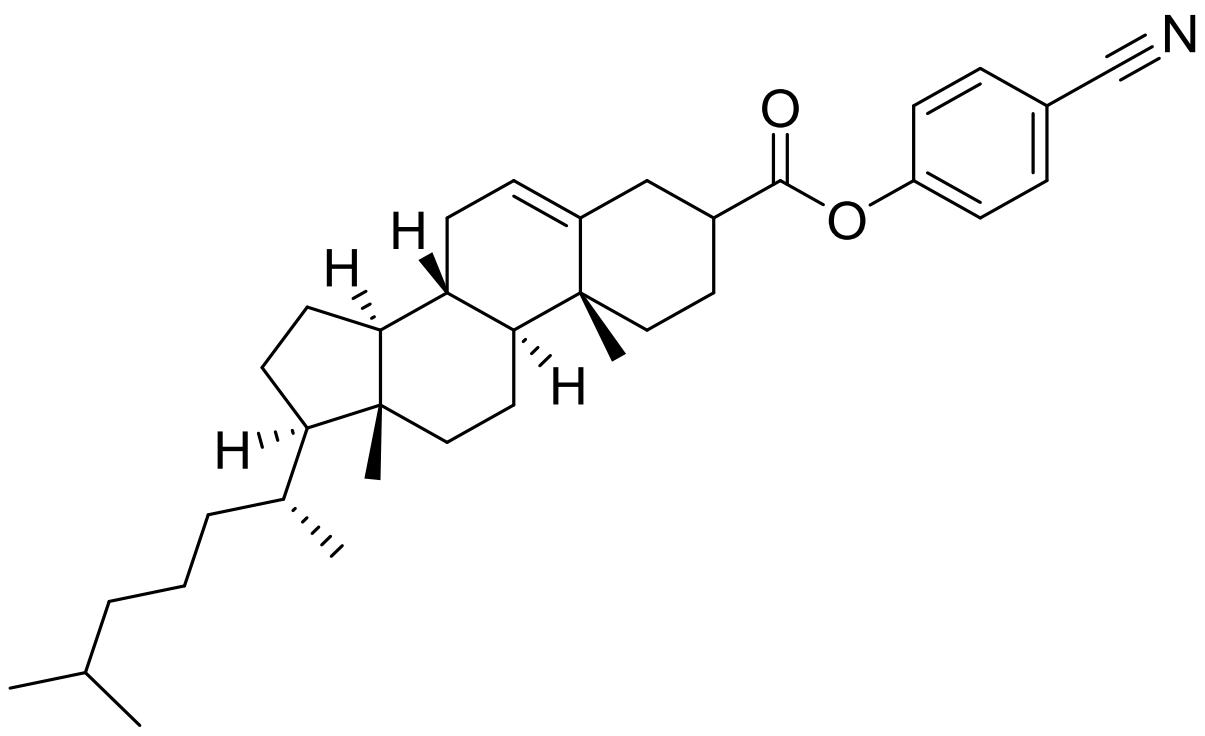

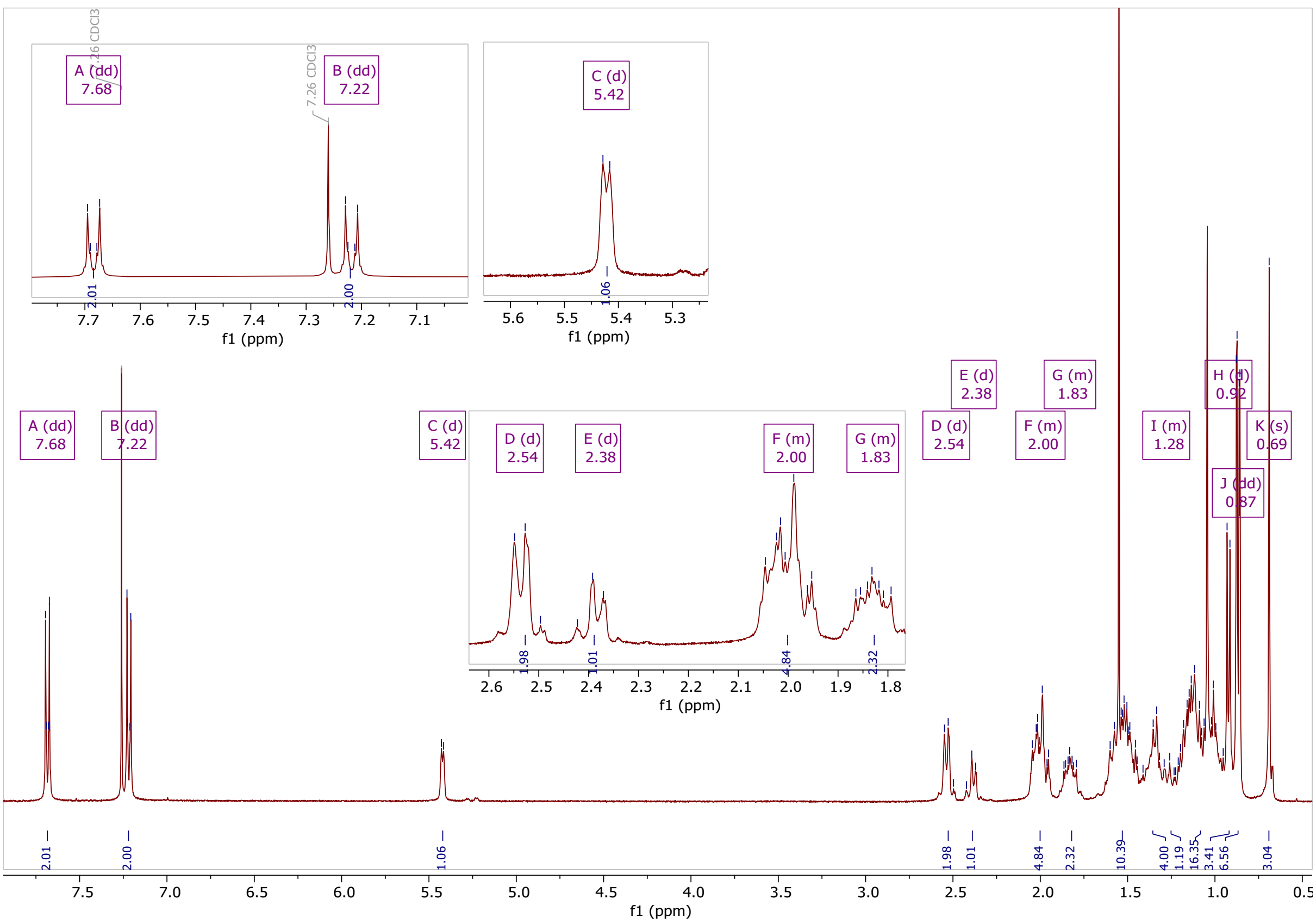

Figure S60 proton NMR spectrum of **12**

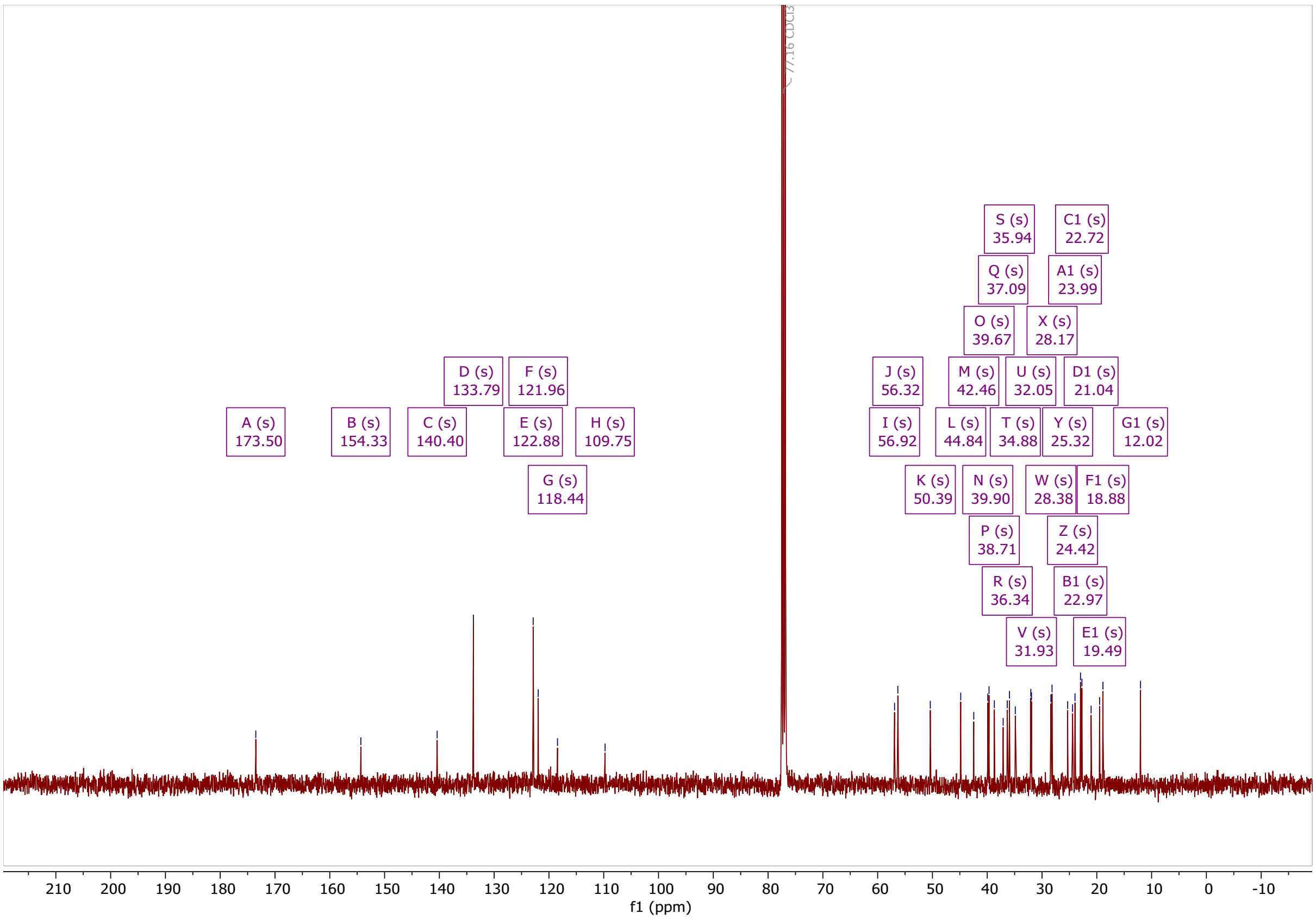

Figure S61 carbon NMR spectrum of **12**.

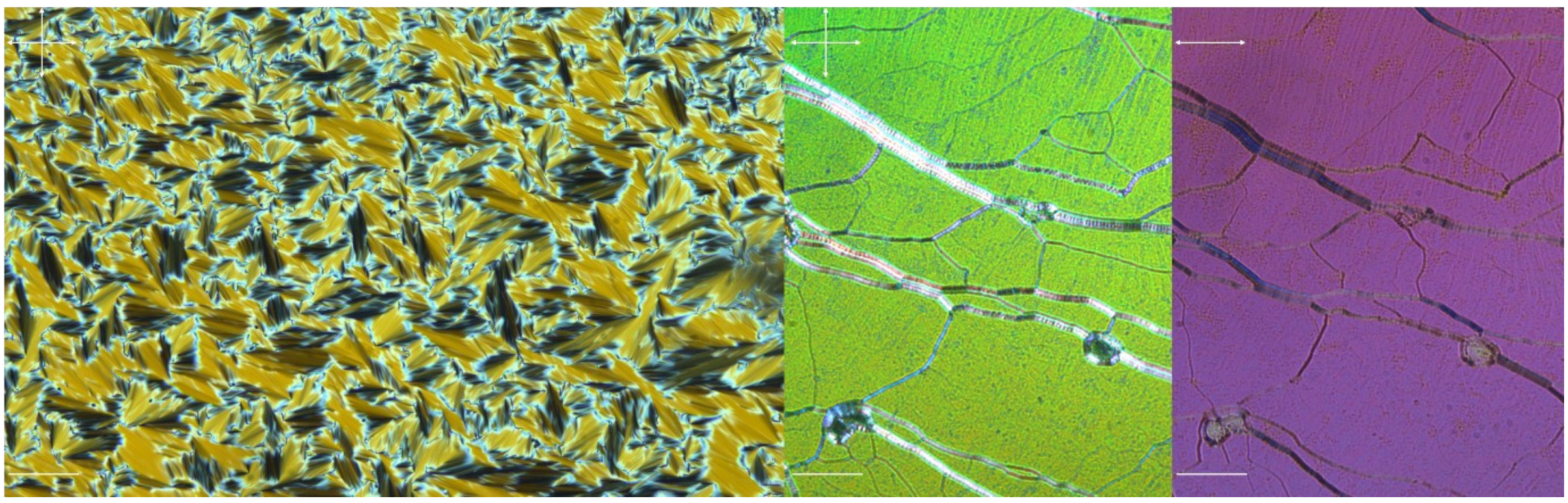

Figure S62 POM images of **12**, SmC* at 150 °C (left), N* at 277 °C with crossed polarisers (central) and 266 °C with aligned polarisers (right). Scale bar (bottom-left) shows 1 μm, arrows show polariser direction.

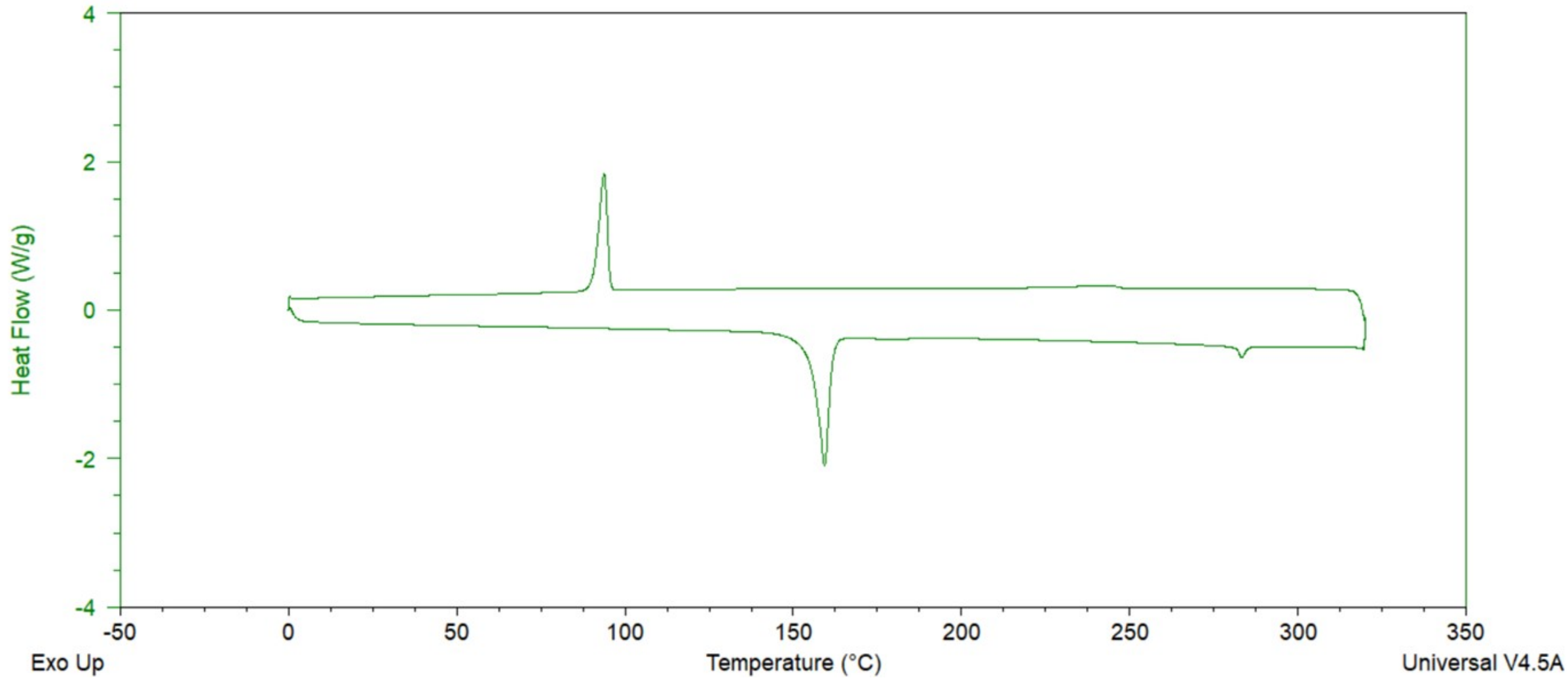

Figure S63 DSC thermogram of **12**.

13 | *4'-cyano-2-fluoro-[1,1'-biphenyl]-4-yl 4-butoxybenzoate*

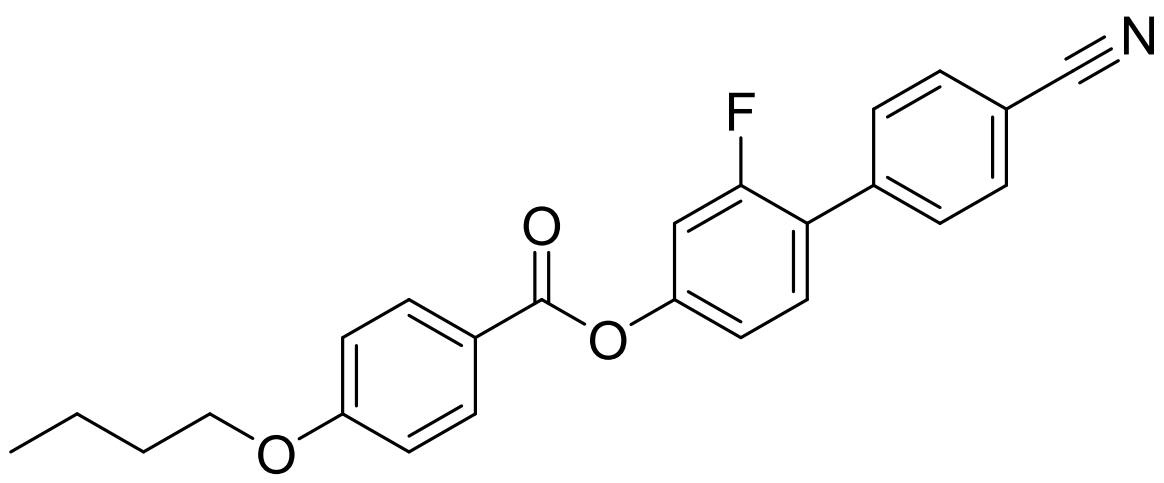

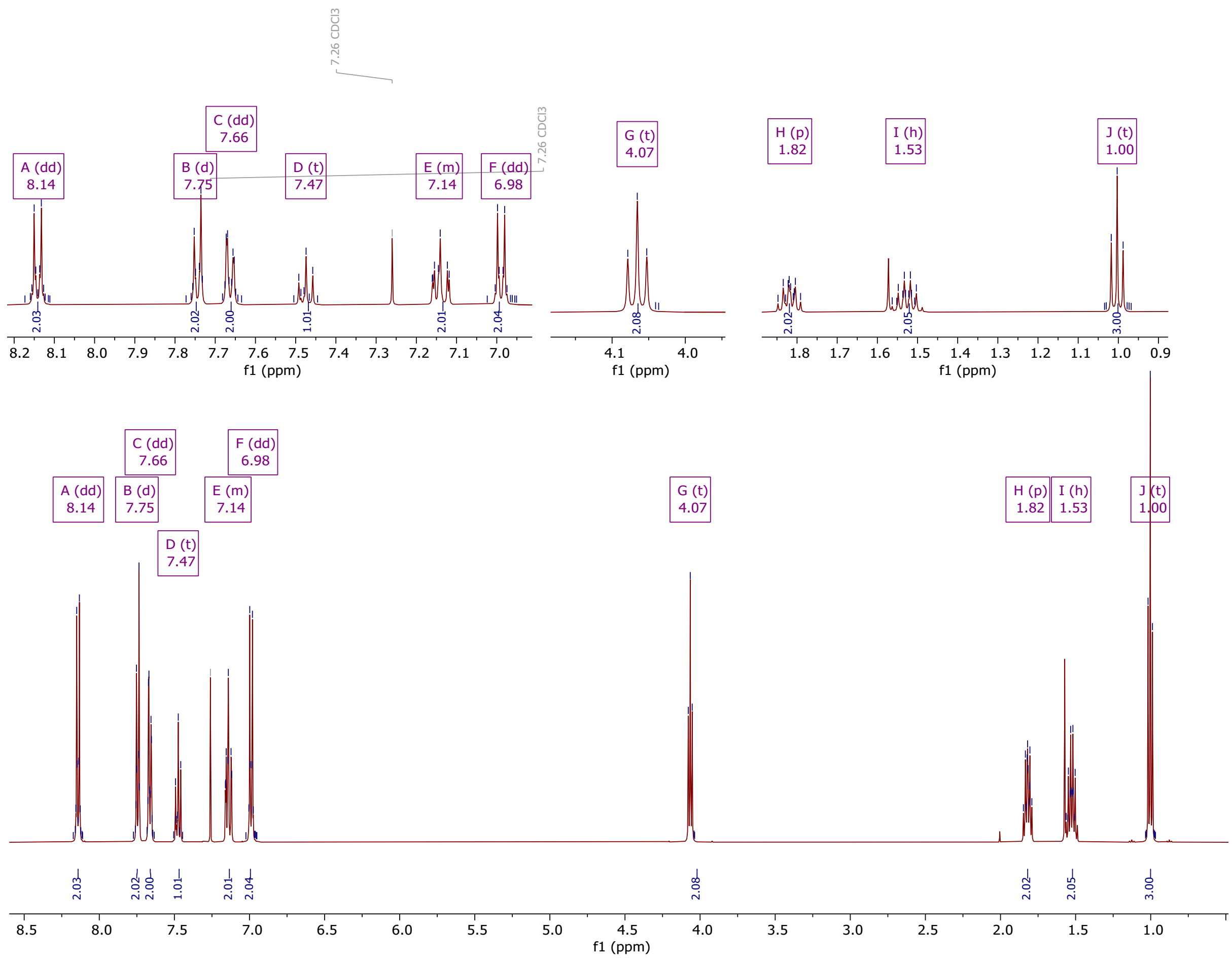

Figure S64 proton NMR spectrum of **13**

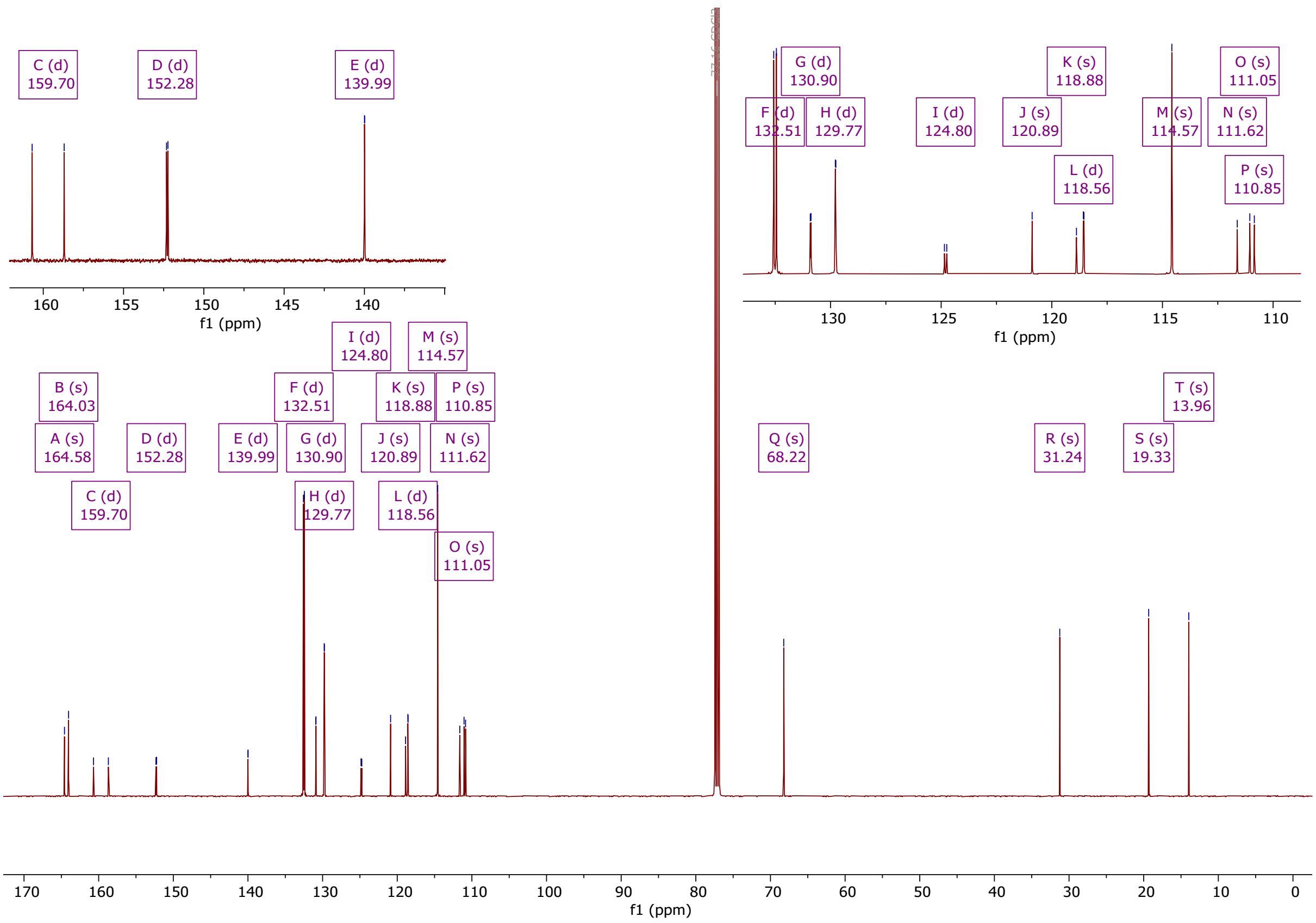

Figure S65 carbon NMR spectrum of **13**

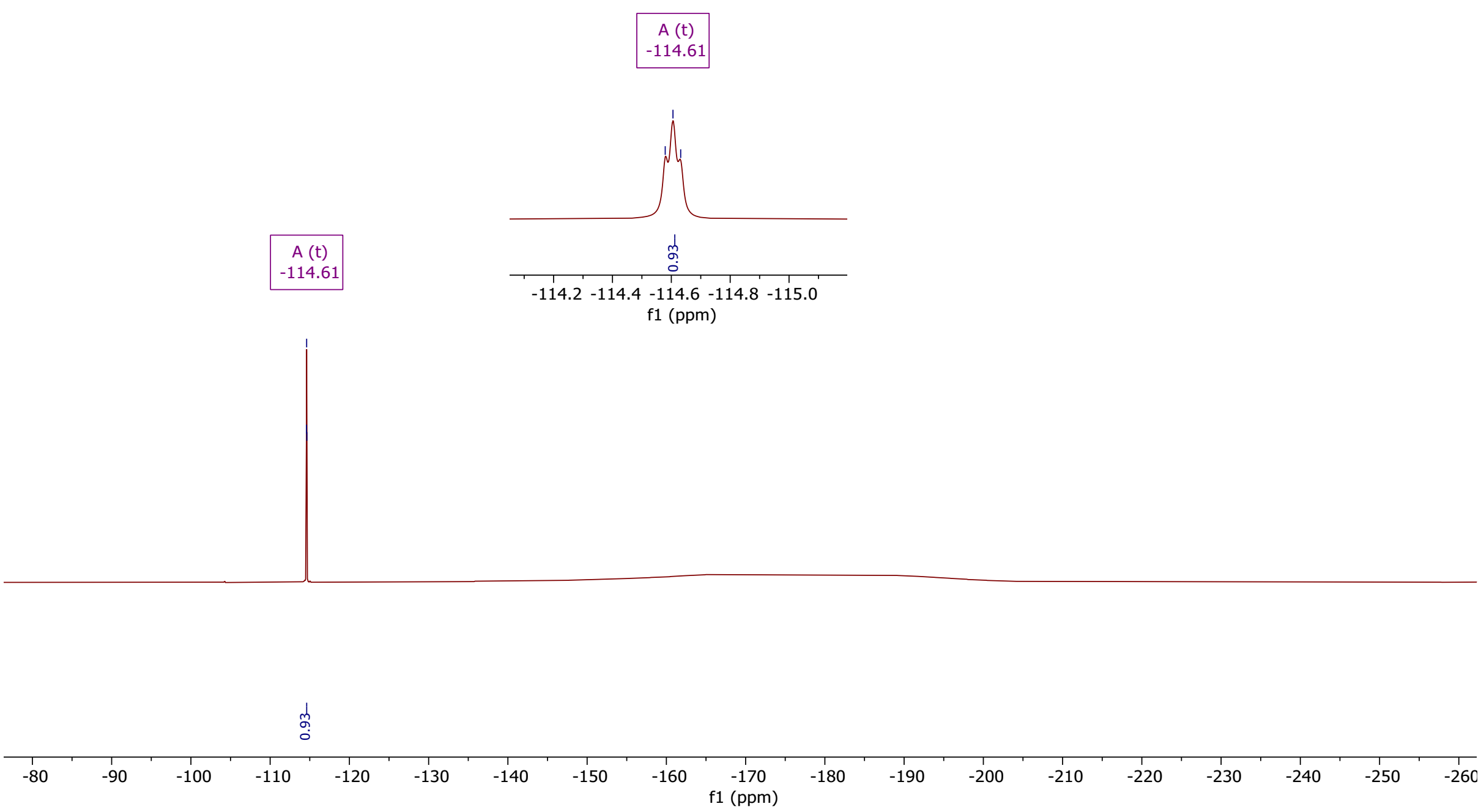

Figure S66 fluorine NMR spectrum of **13**.

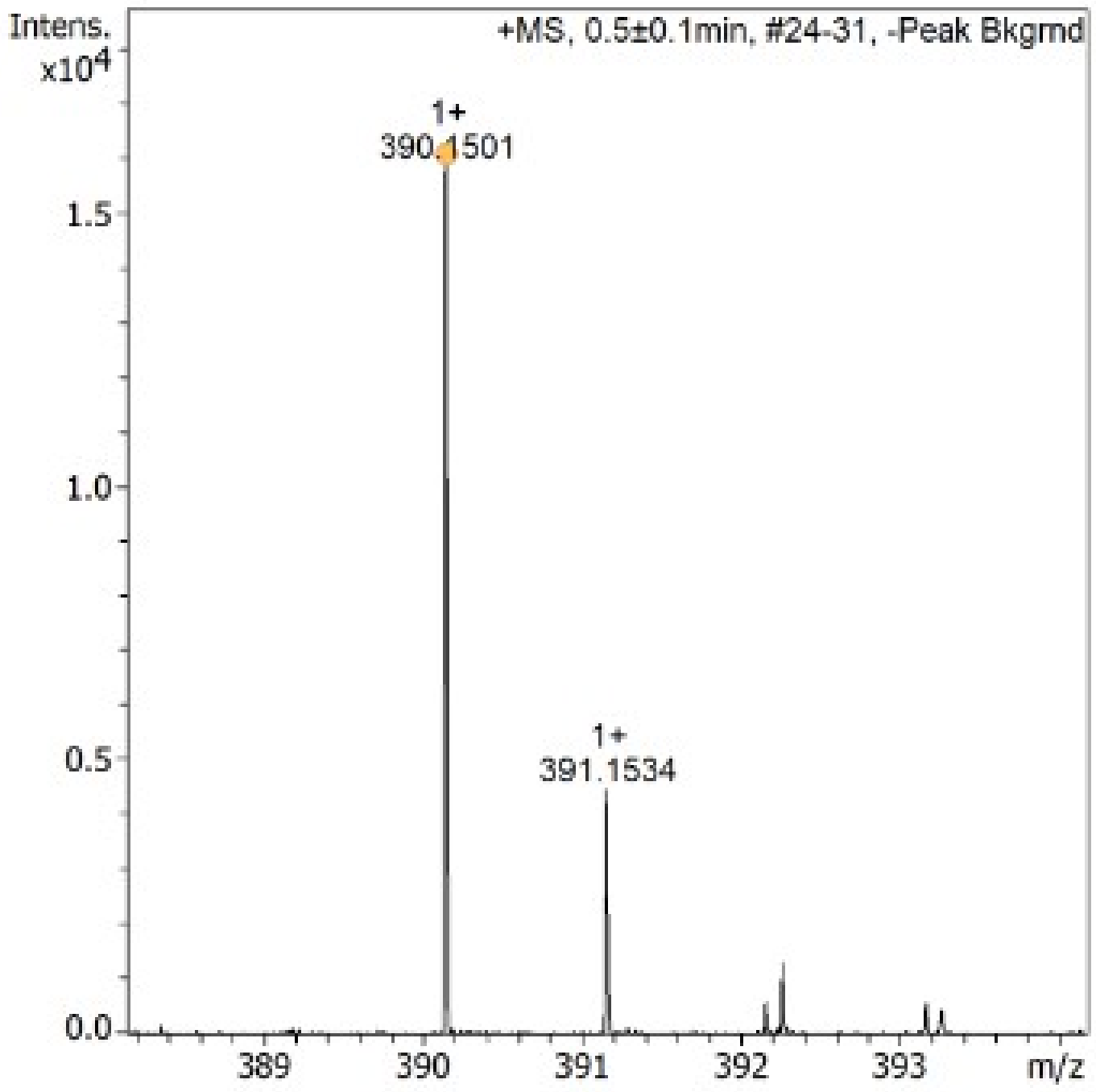

Figure S67 HRMS of **13**

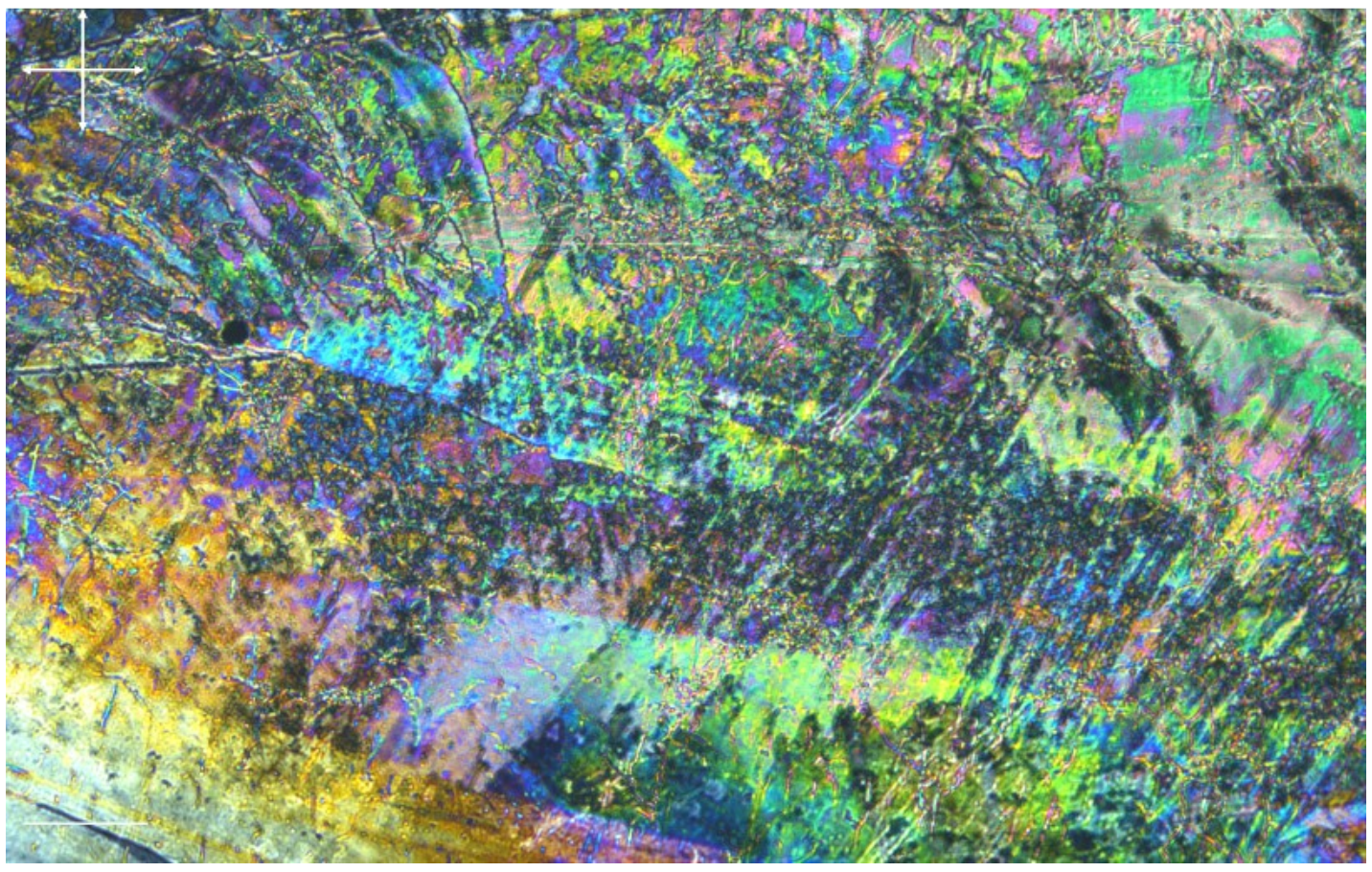

Figure S68 POM image of **13**, N at 130 °C. Scale bar (bottom-left) shows 1 µm, arrows show polariser direction.

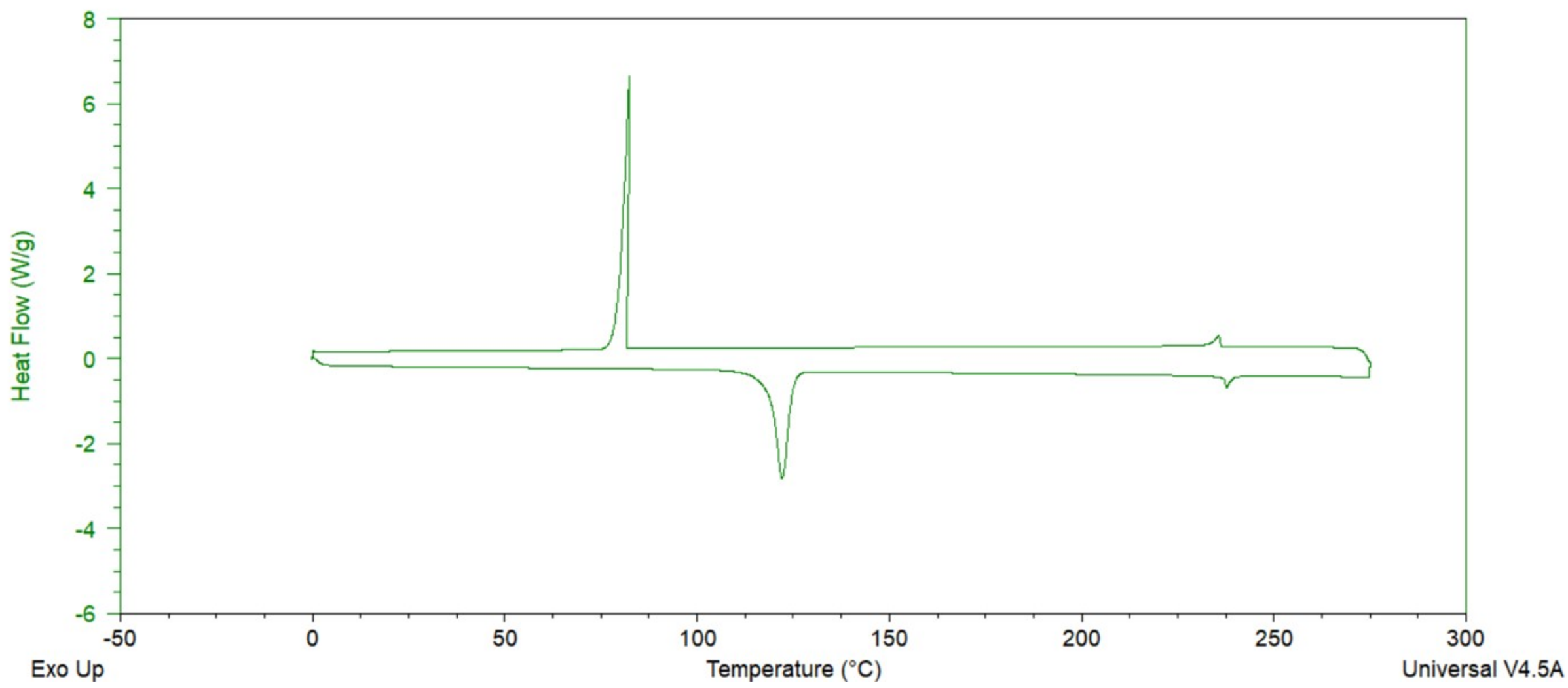

Figure S69 DSC thermogram of **13**.

14 | *4'-nitro-[1,1'-biphenyl]-4-yl 6-hexylspiro[3.3]heptane-2-carboxylate*

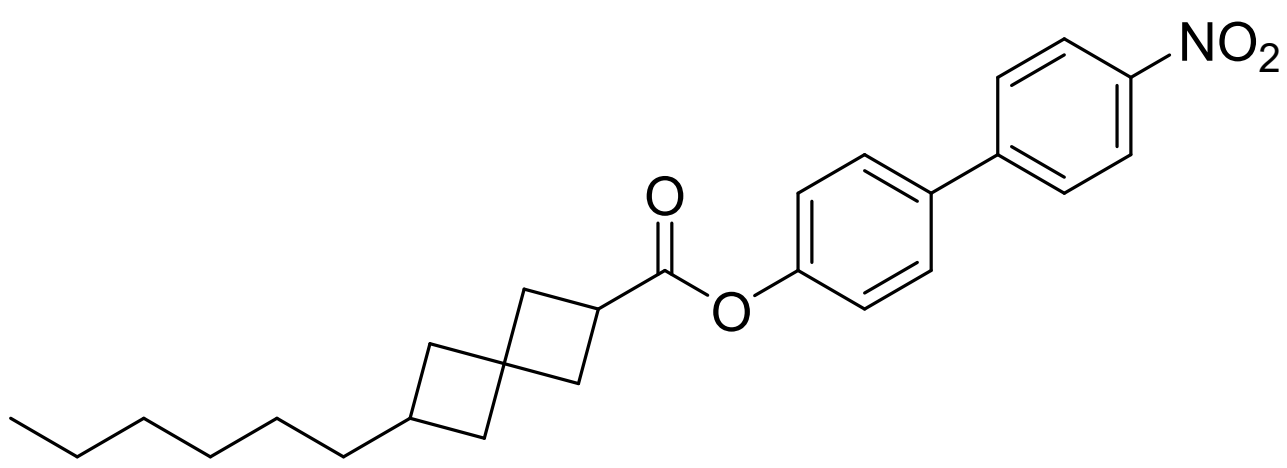

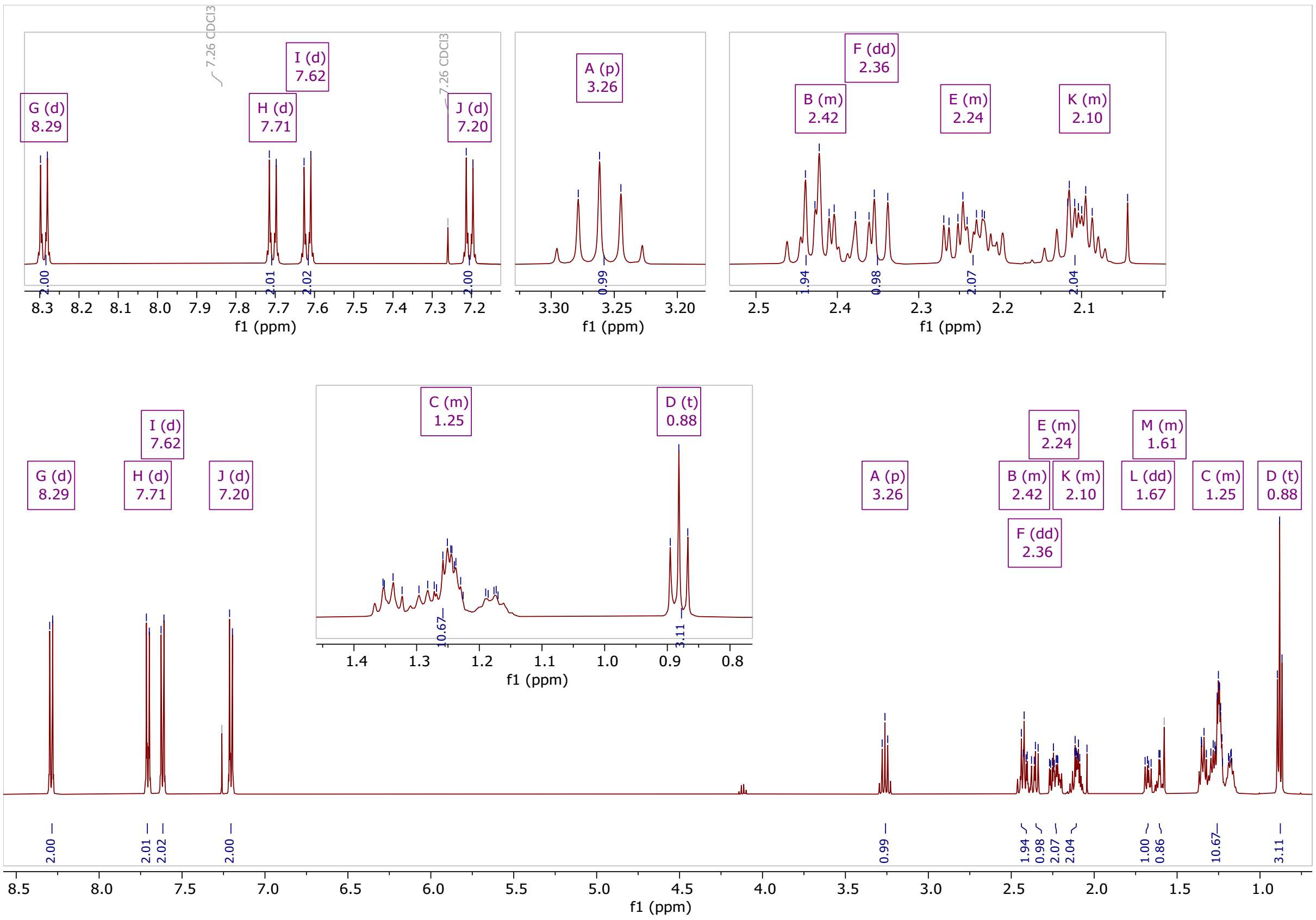

Figure S70 proton NMR spectrum of **14**

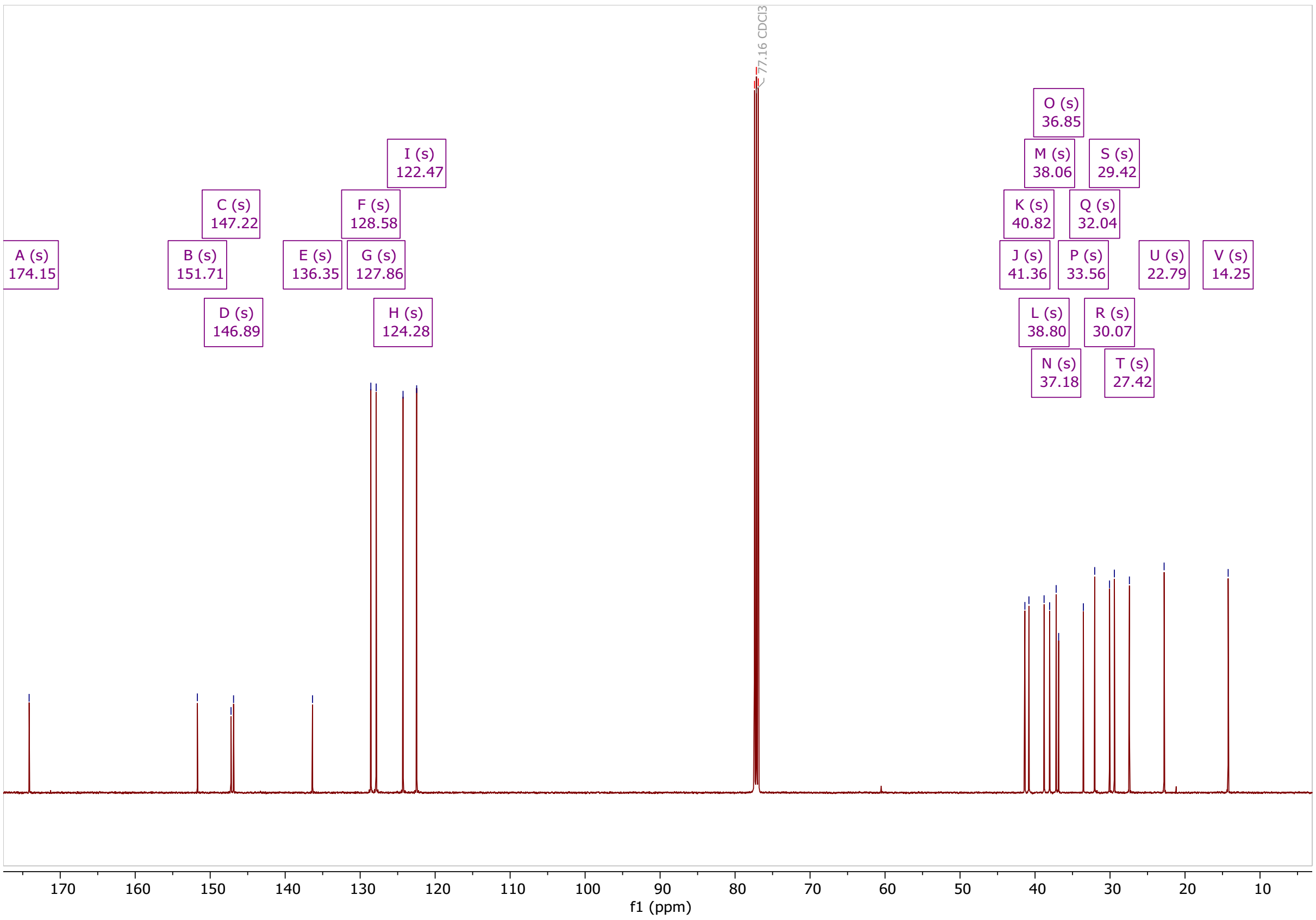

Figure S71 carbon NMR spectrum of **14.**

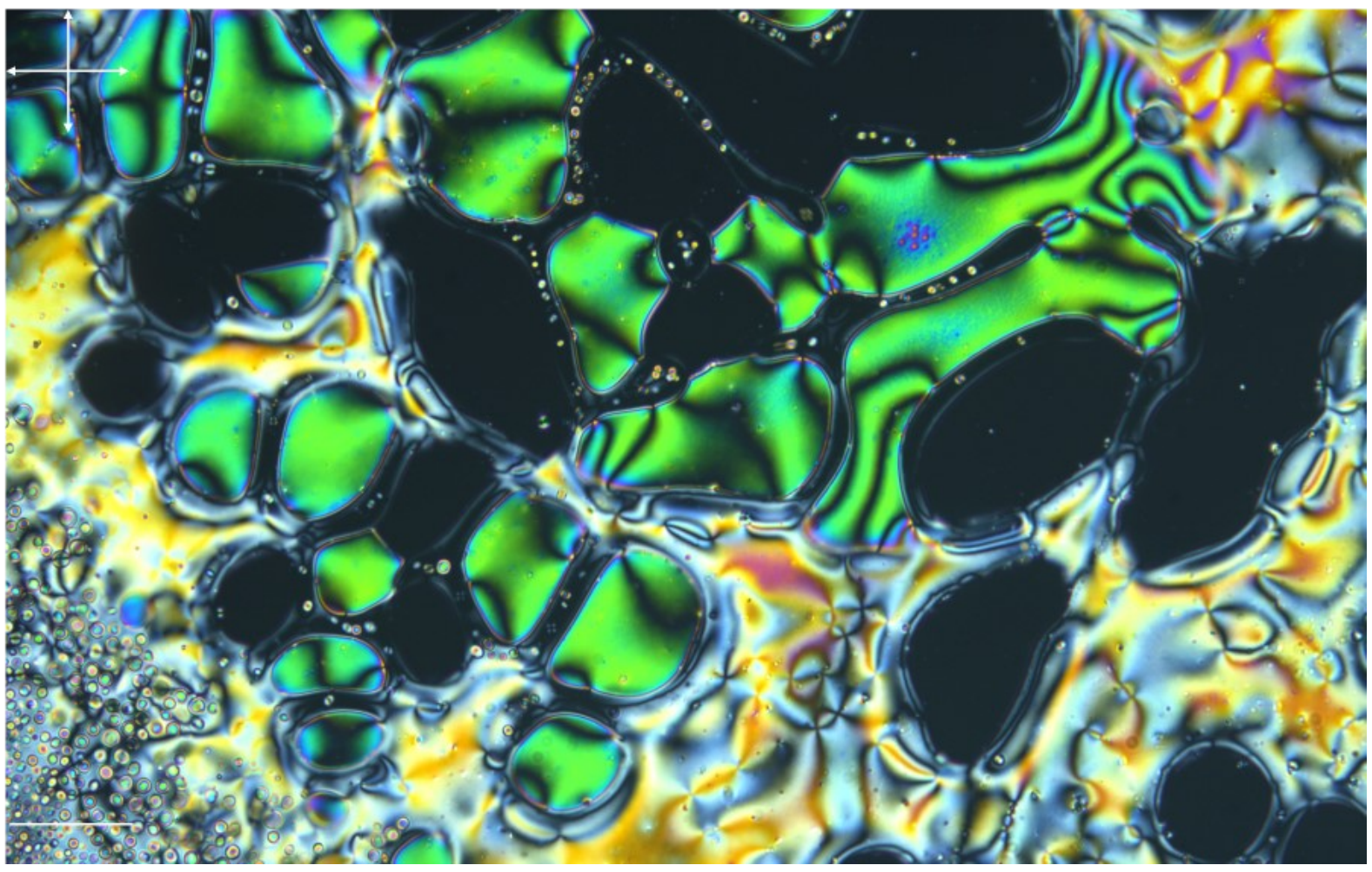

Figure S72 POM image of **14**, N at 115 °C. Scale bar (bottom-left) shows 1 µm, arrows show polariser direction.

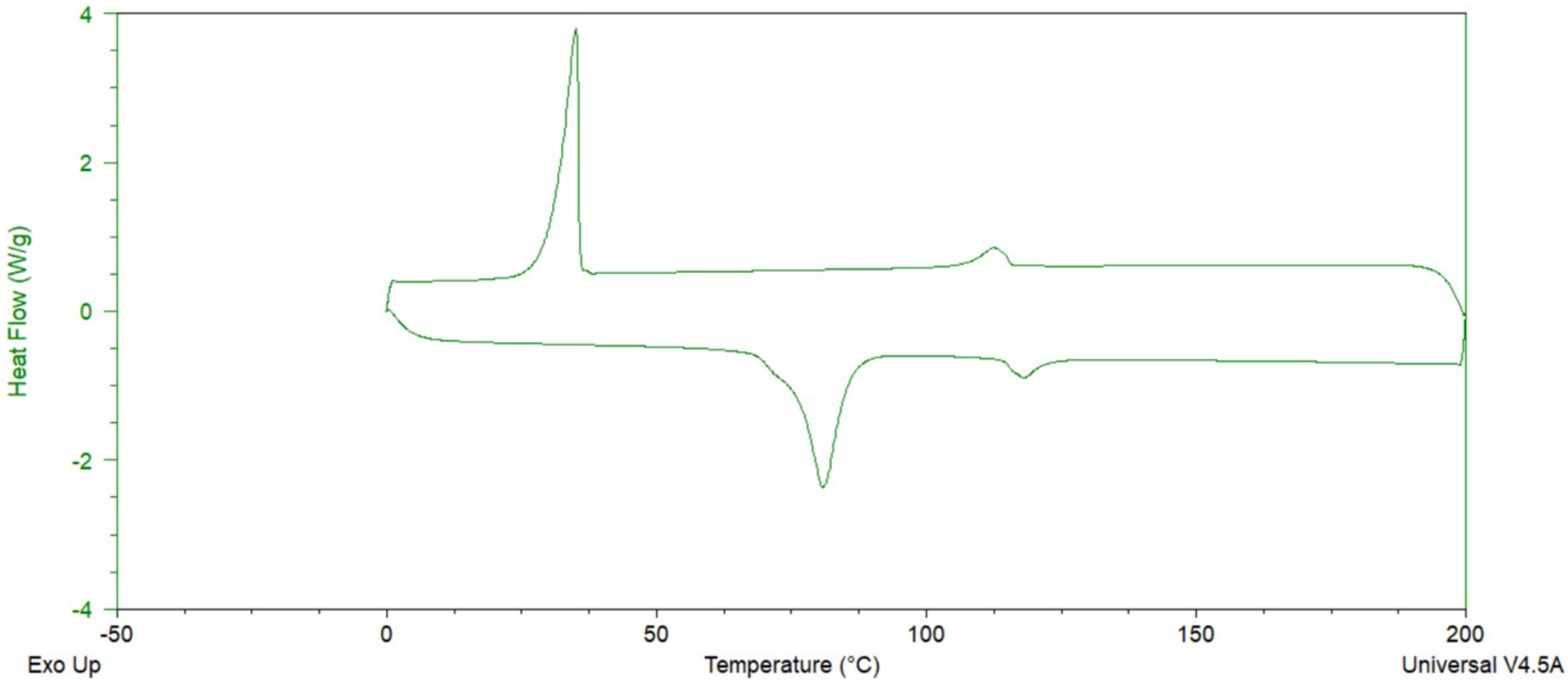

Figure S73 DSC thermogram of **14**.

15 | *4-bromo-3,5-difluorophenyl 3,5-difluoro-4'-pentyl-[1,1'-biphenyl]-4-carboxylate*

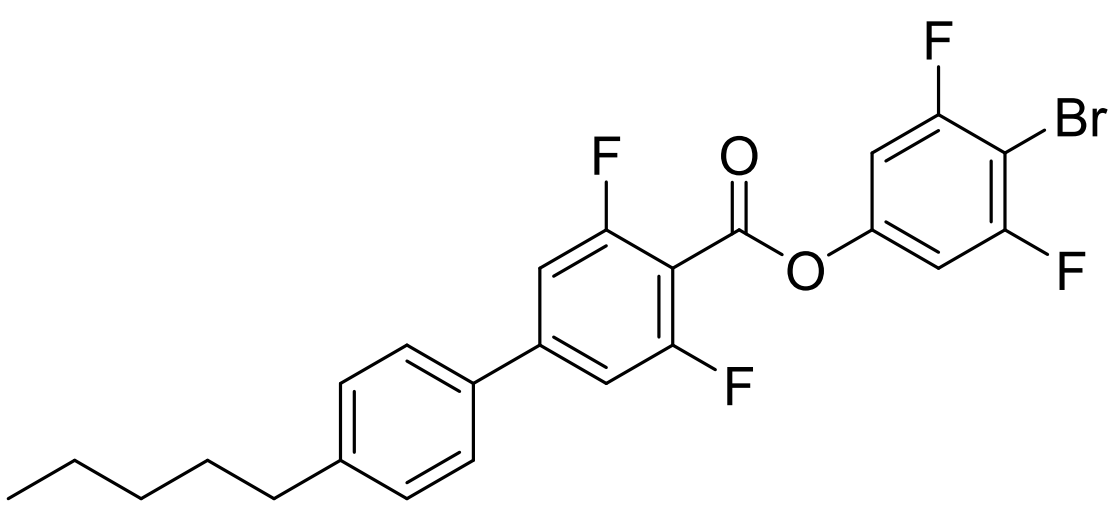

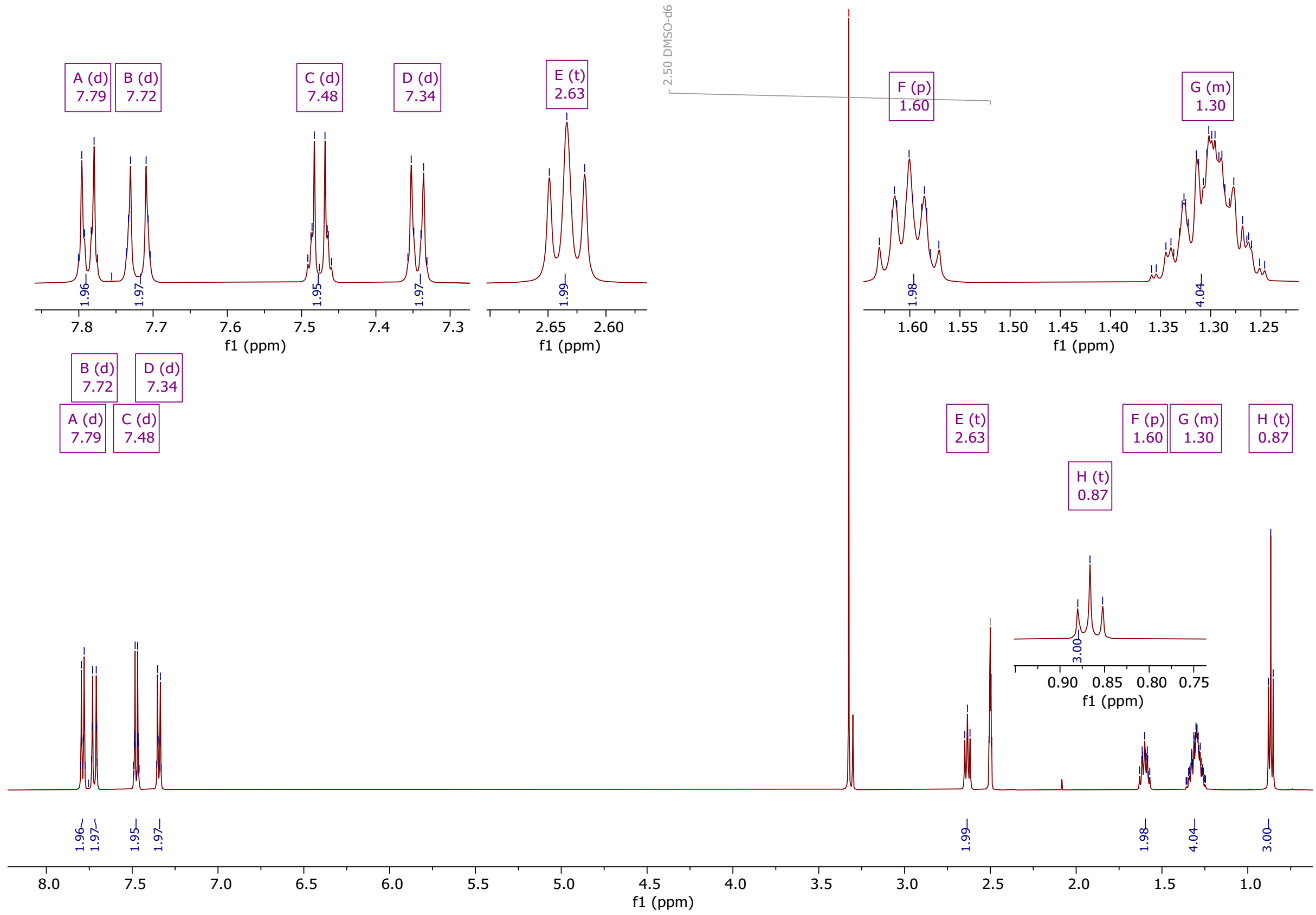

Figure S74 proton NMR spectrum of **15**

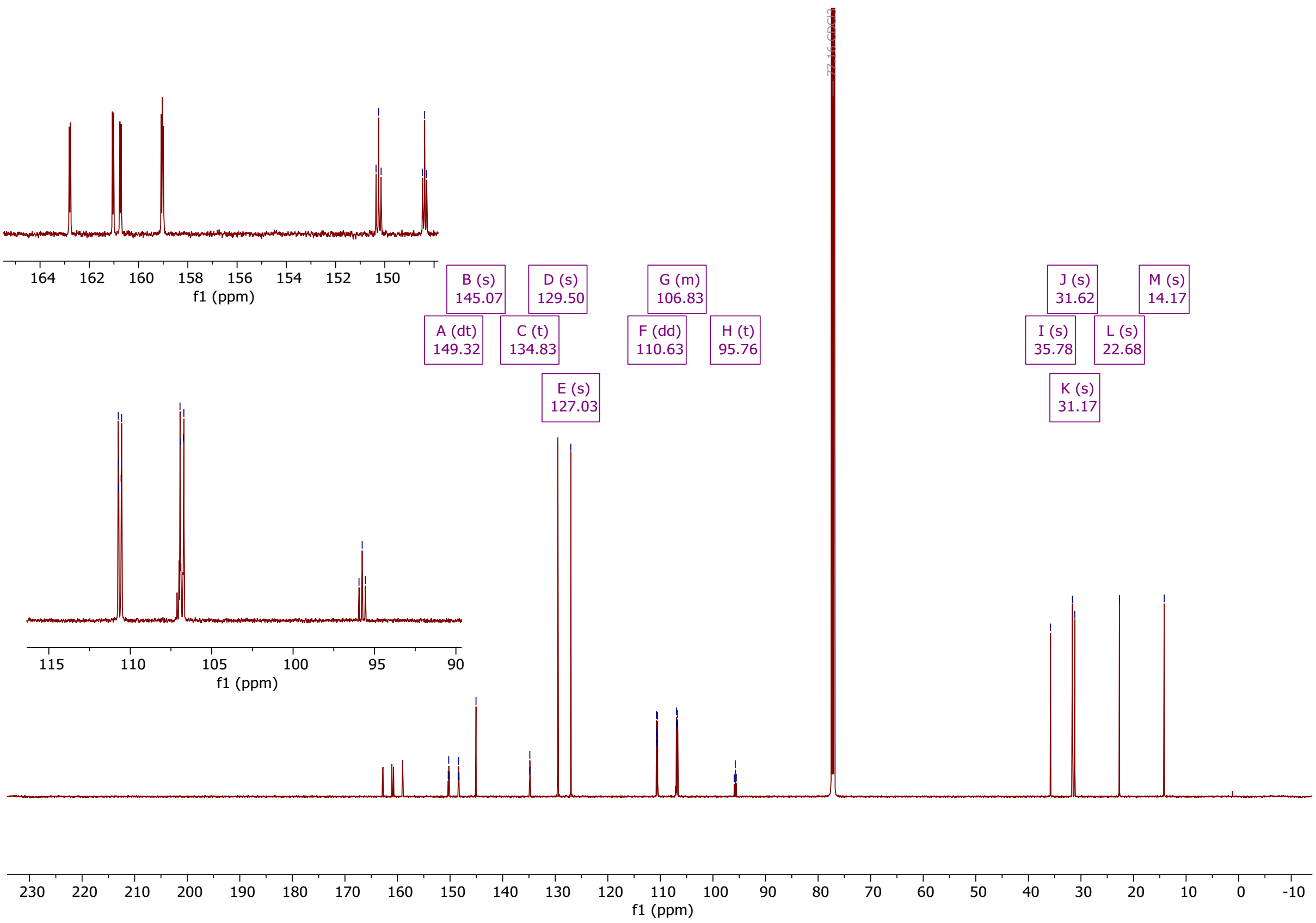

Figure S75 carbon NMR spectrum of **15.** Overlapping C=O, and Ar-F bonds do not have multiple labels but are recorded in data set in section 3.

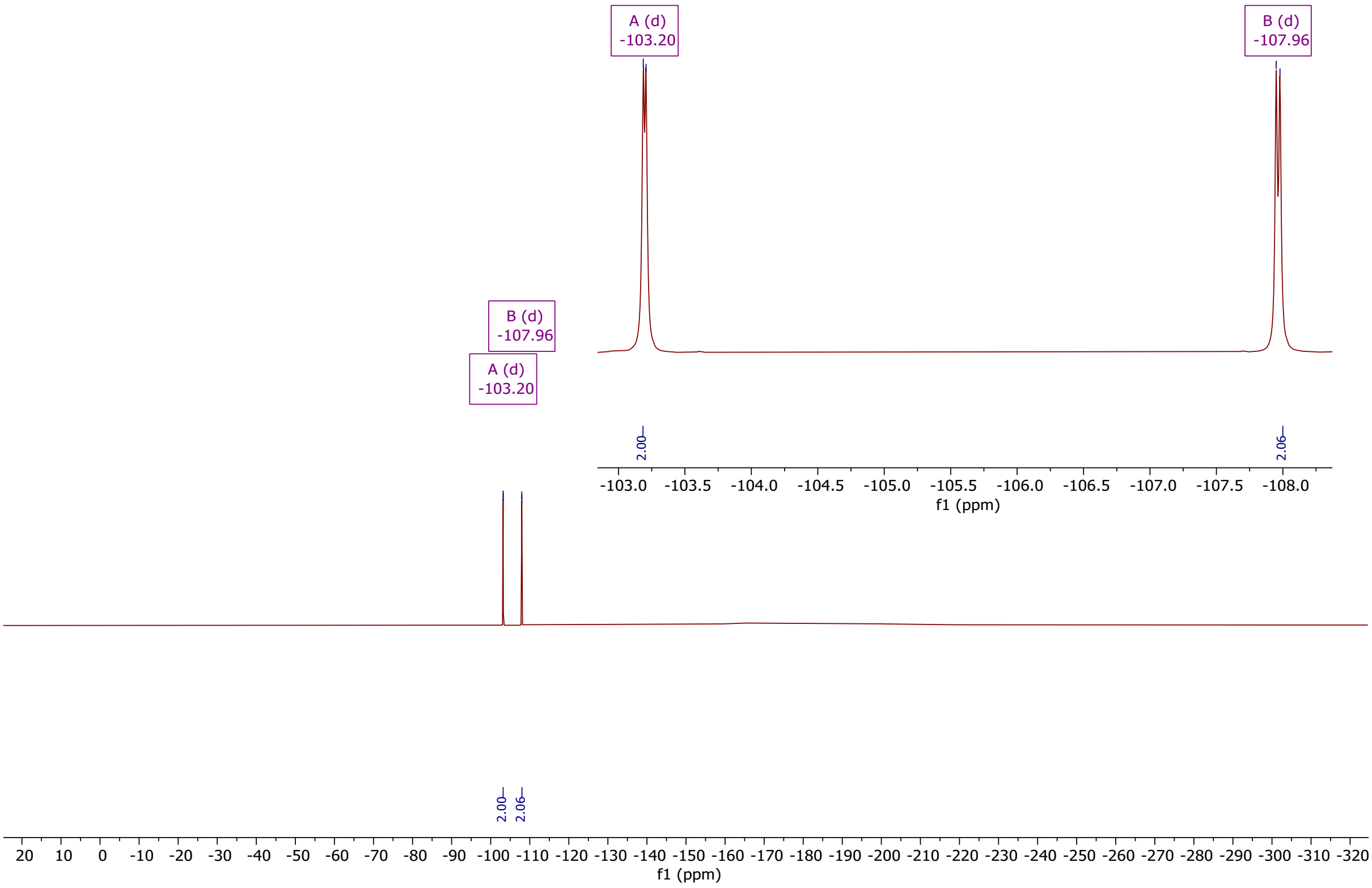

Figure S76 fluorine NMR spectrum of **15.**

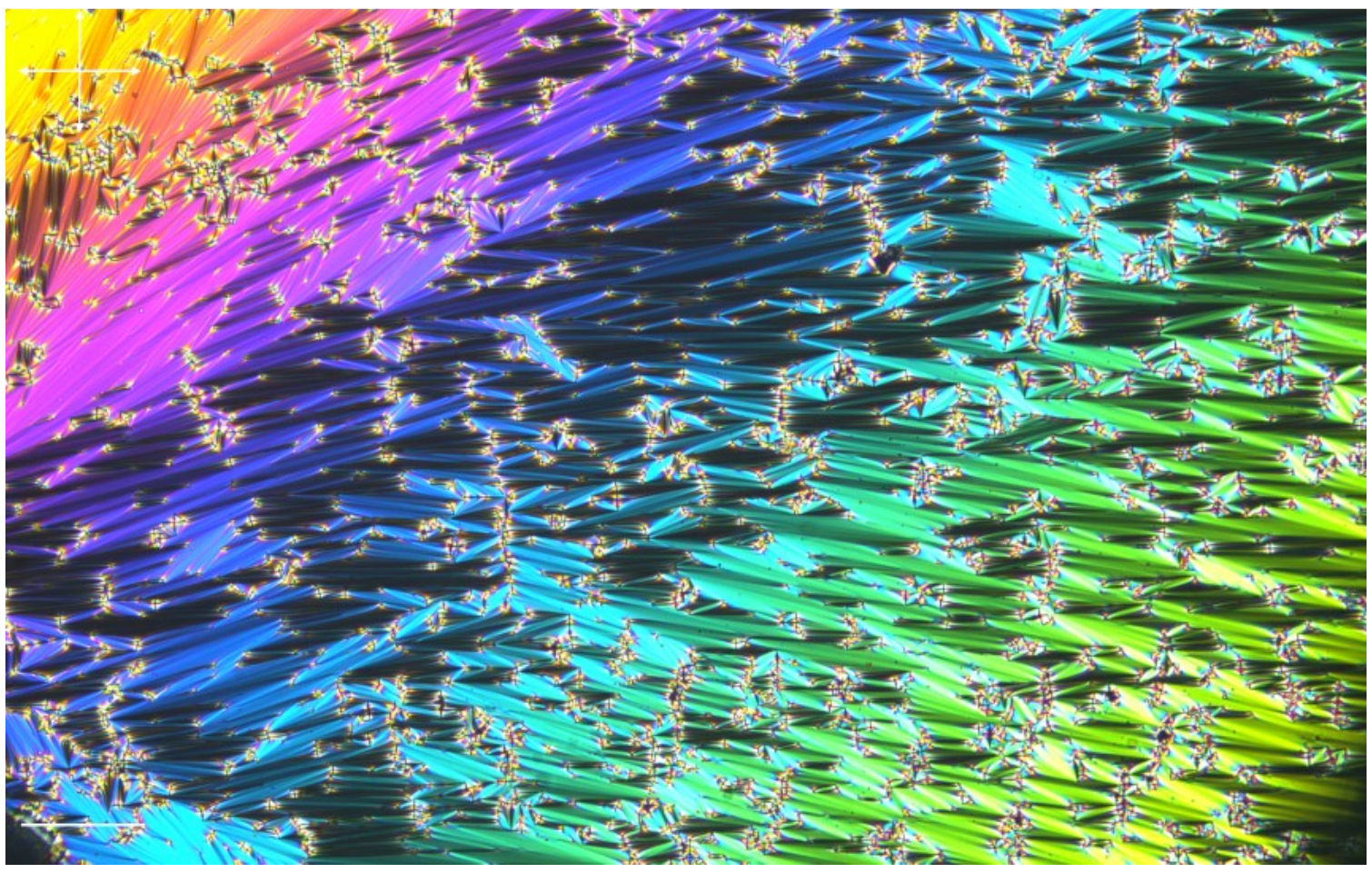

Figure S77 POM image of **15**, SmA at 87 °C. Scale bar (bottom-left) shows 1 μm, arrows show polariser direction.

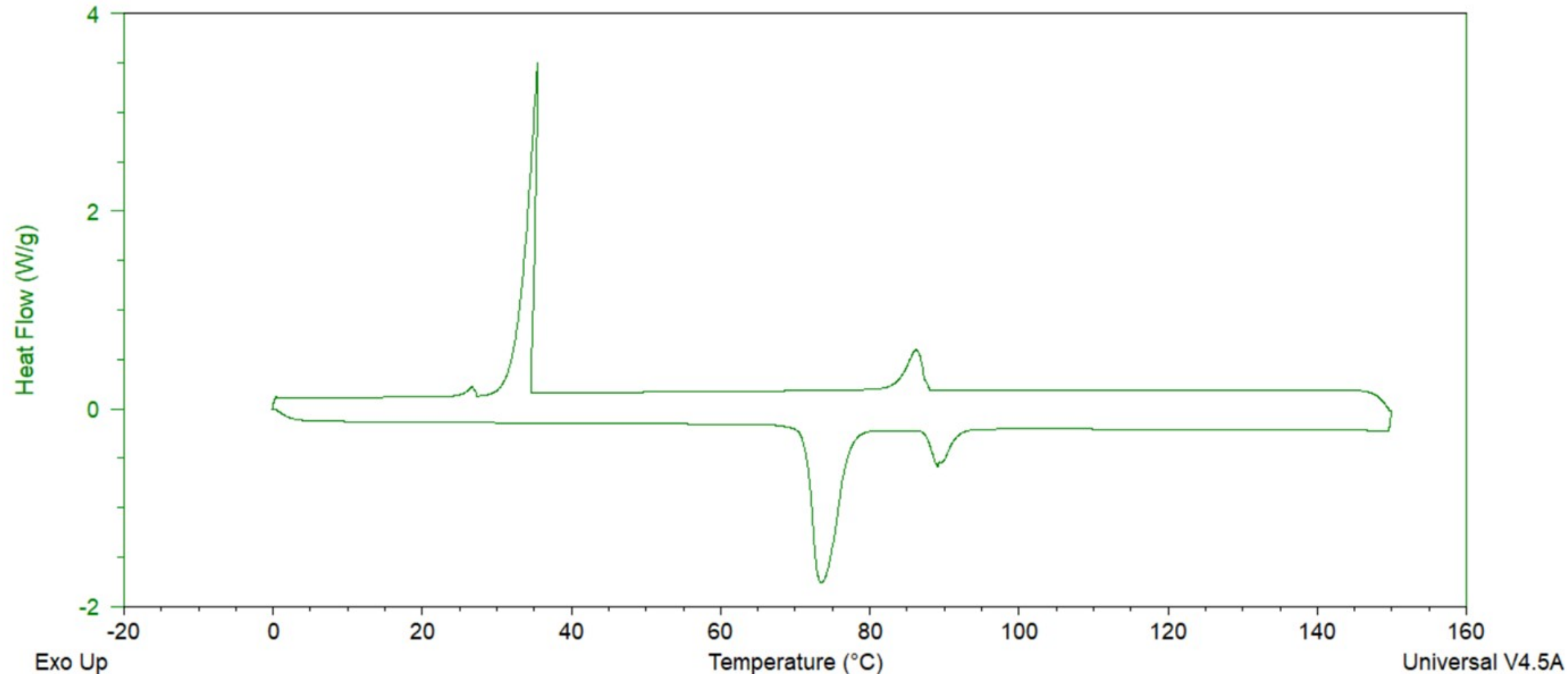

Figure S78 DSC thermogram of **15**.

16 | *(E)-4-(3-ethoxy-3-oxoprop-1-en-1-yl)phenyl 6-methoxy-2-naphthoate*

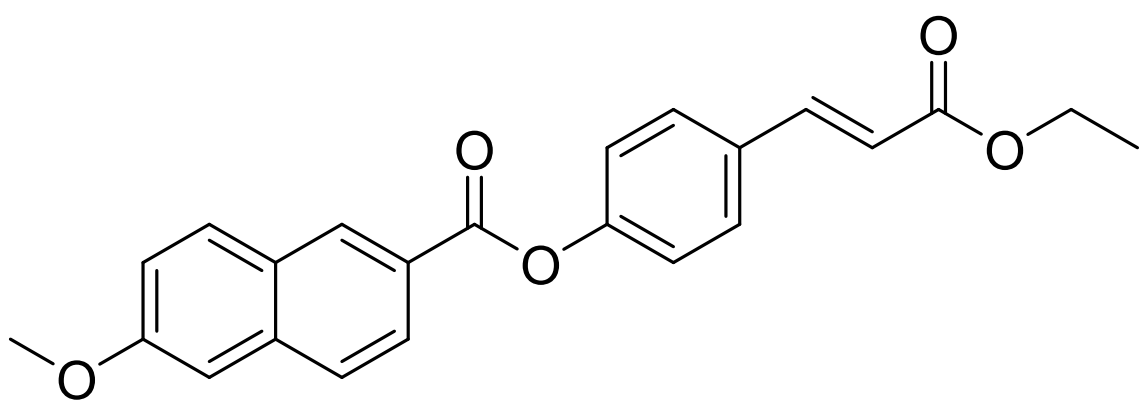

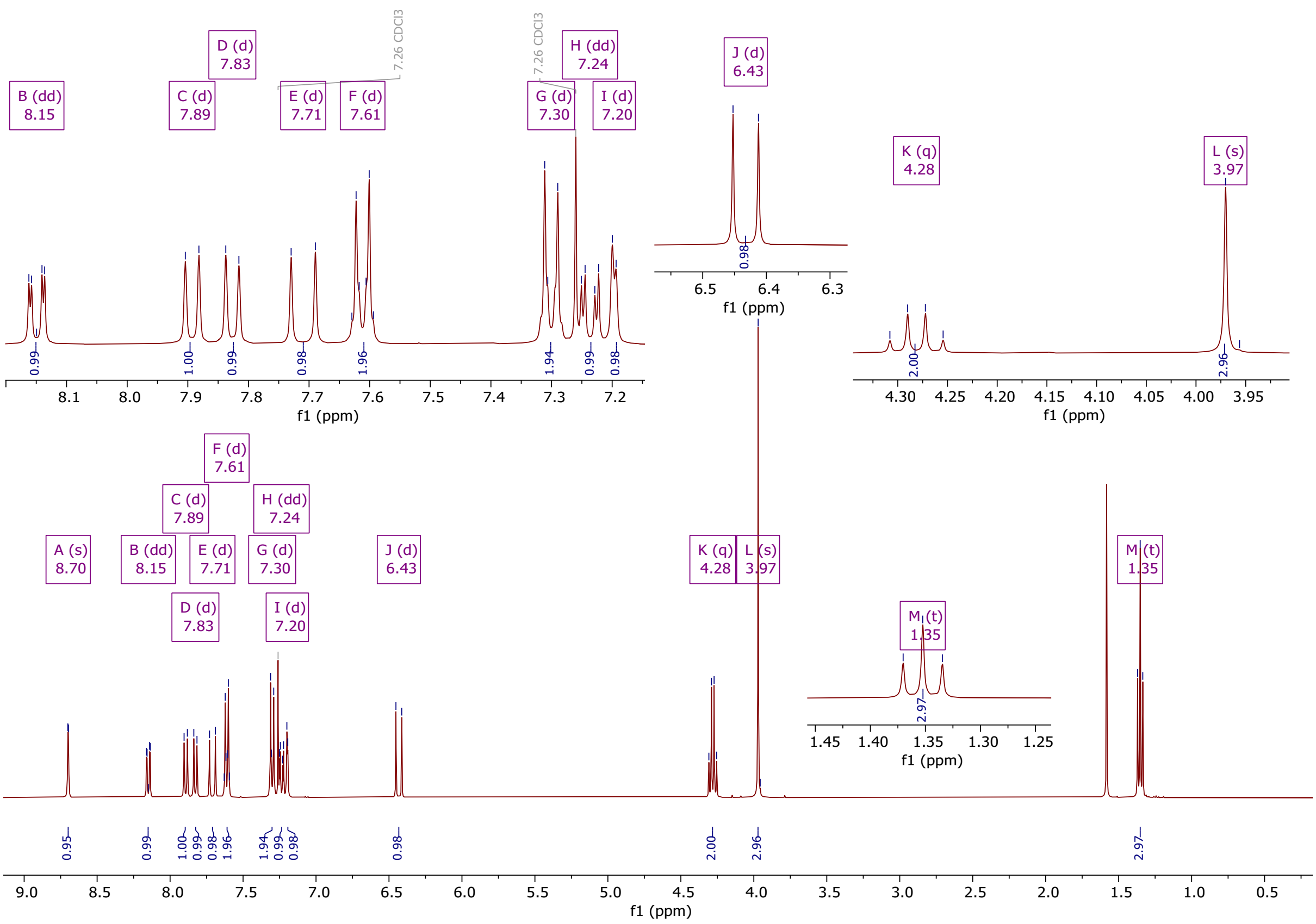

Figure S79 proton NMR spectrum of **16**

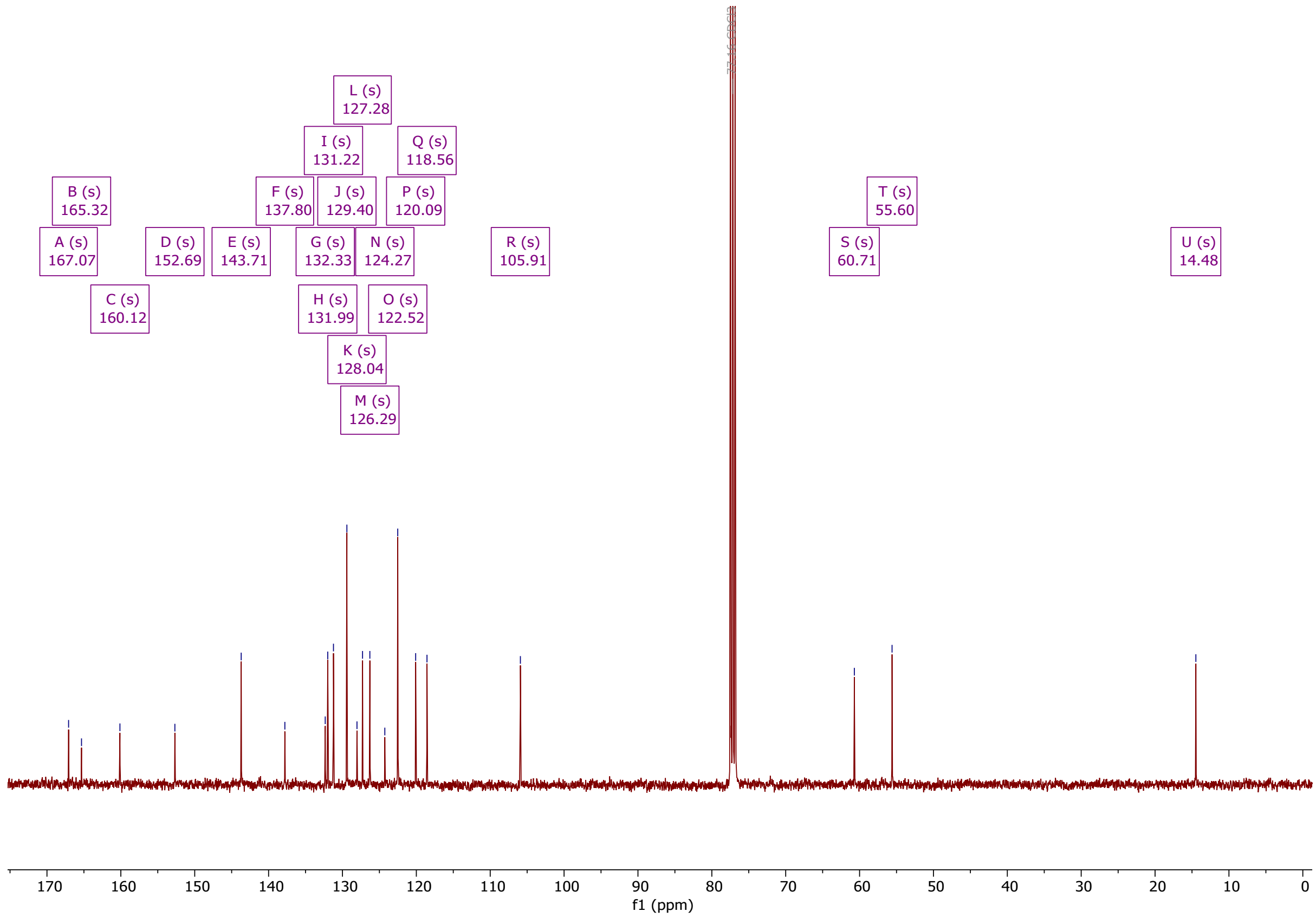

Figure S80 carbon NMR spectrum of **16**

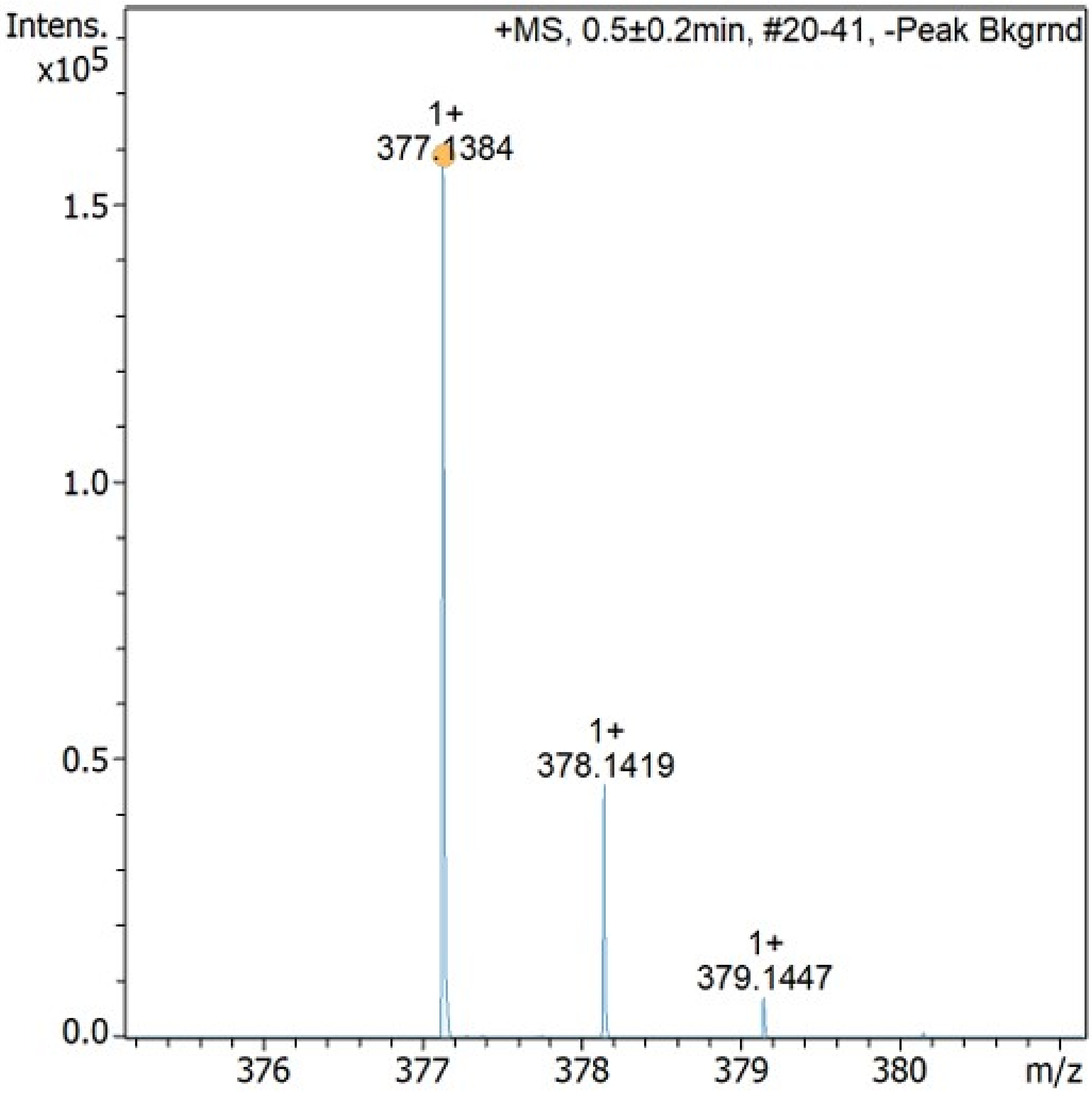

Figure S81 HRMS of **16**.

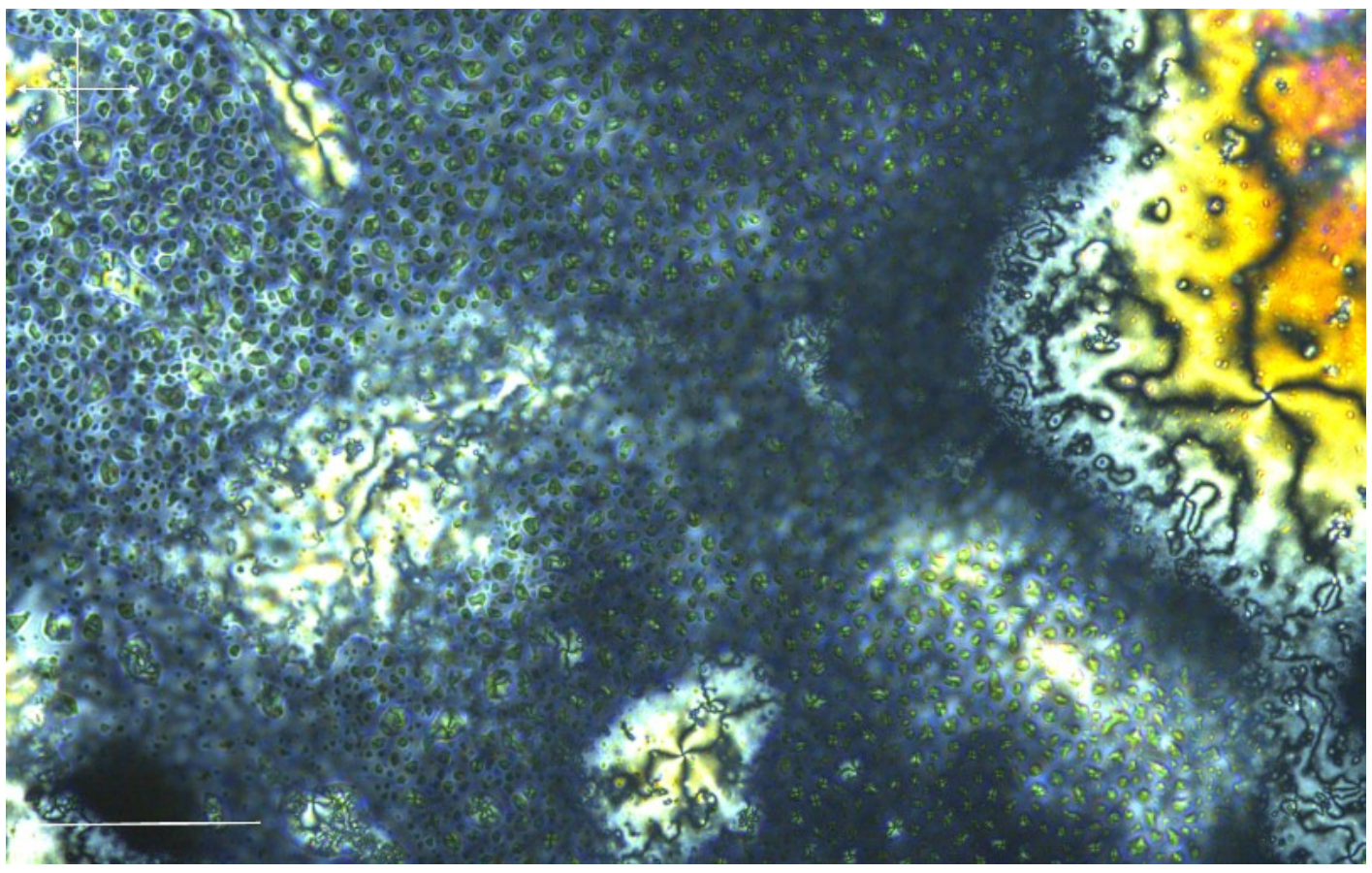

Figure S82 POM image of **16**, showing a N phase at 150 °C. Scale bar (bottom-left) shows 1 μm, arrows show polariser direction.

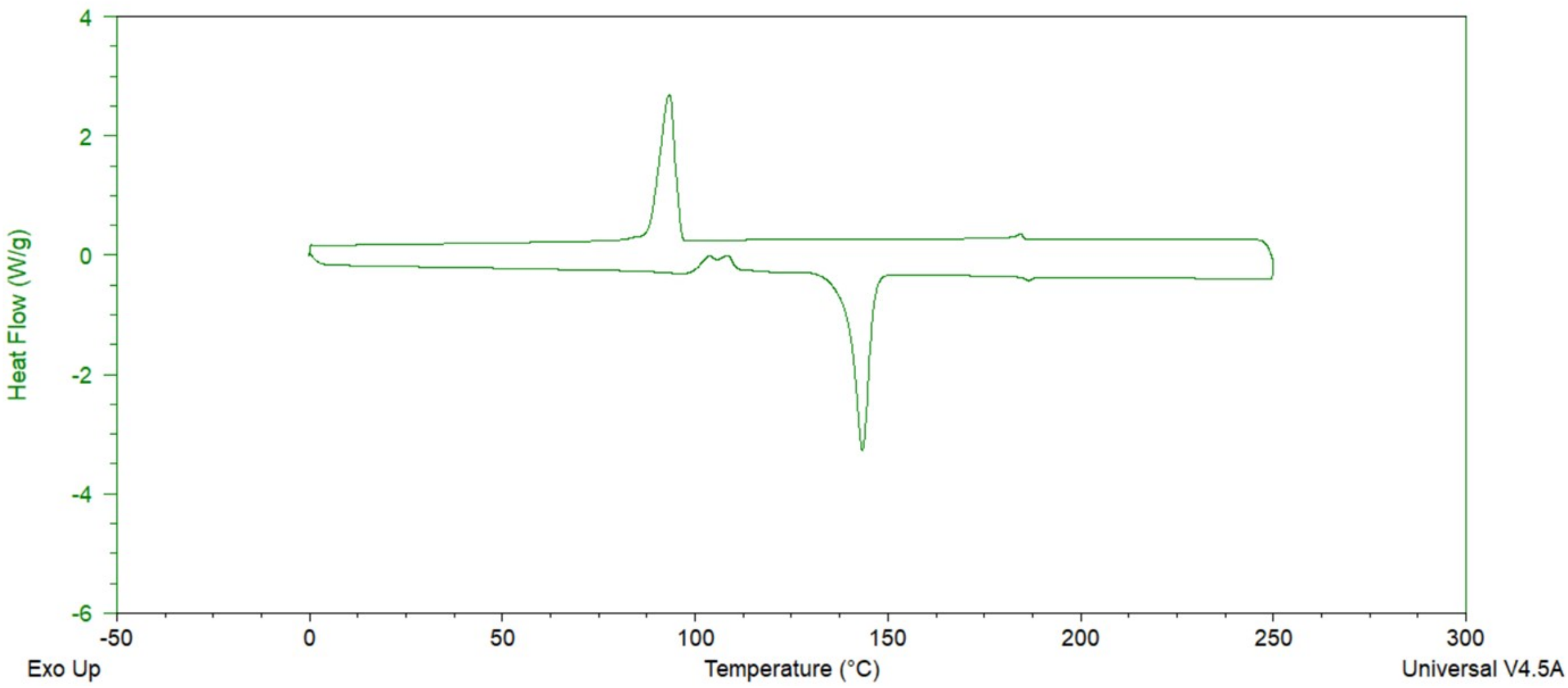

Figure S83 DSC thermogram of **16**.

17 | *4-(5-heptylpyrimidin-2-yl)phenyl 4'-ethoxy-2',3,5-trifluoro-[1,1'-biphenyl]-4-carboxylate*

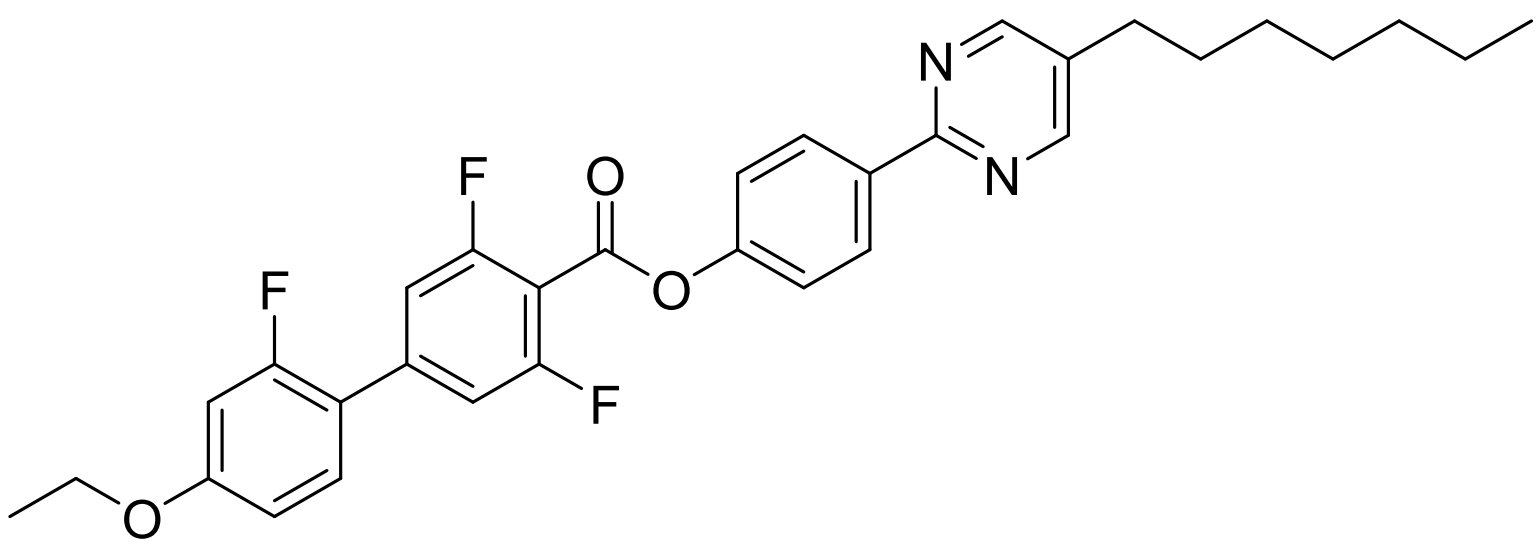

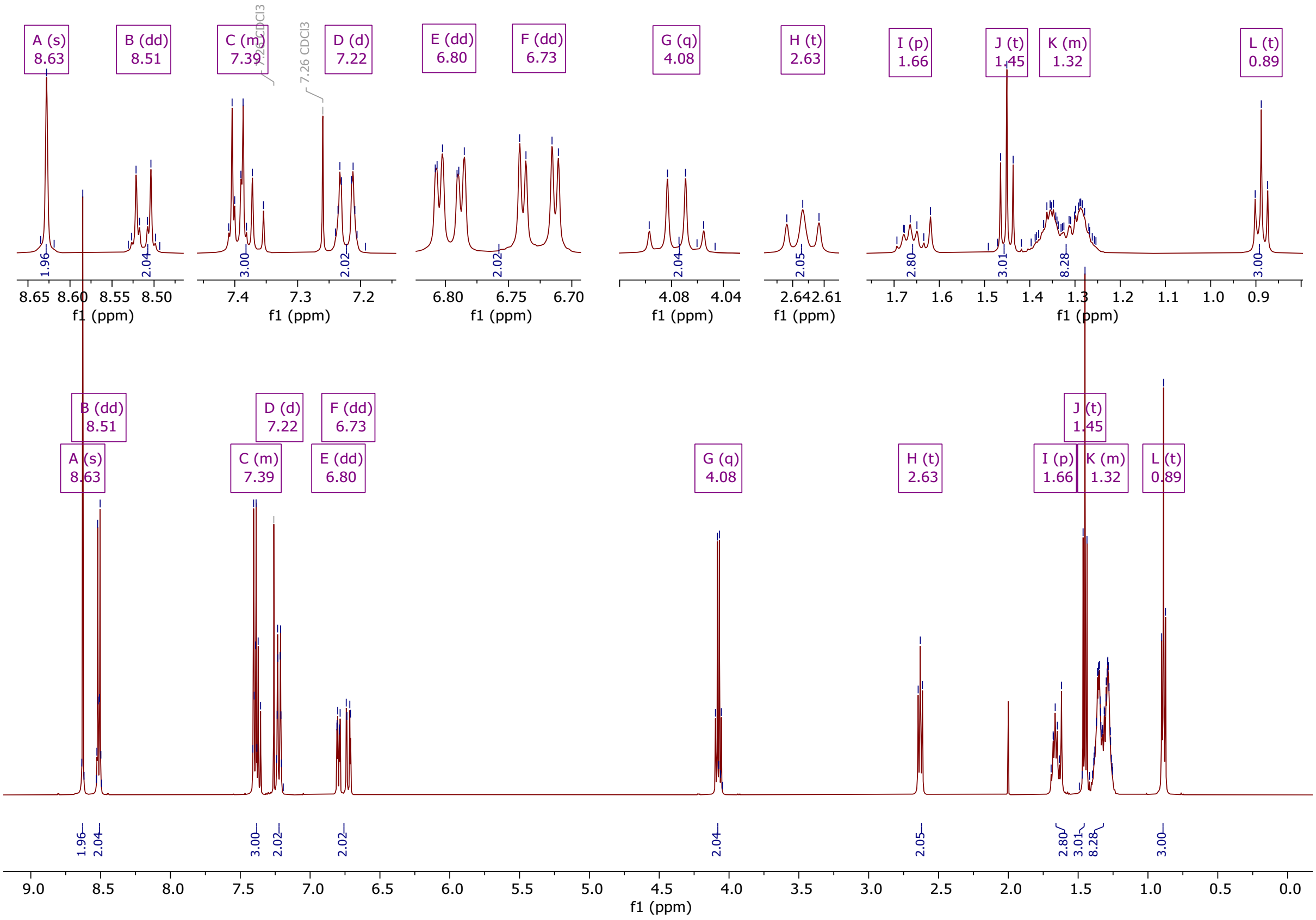

Figure S84 proton NMR spectrum of **17**

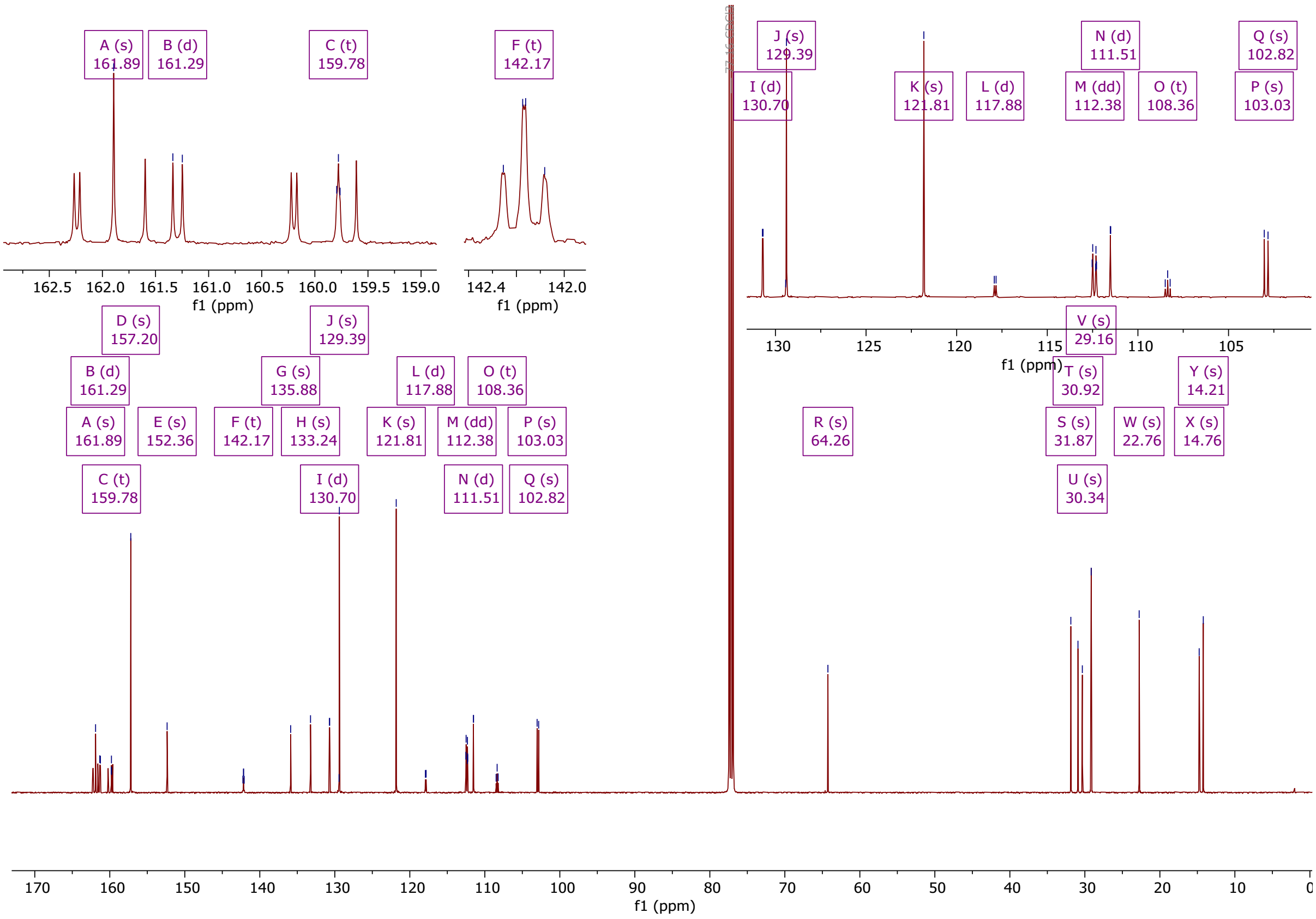

Figure S85 carbon NMR spectrum of **17.** Overlapping C=O, and Ar-F bonds do not have multiple labels but are recorded in data set in section 3.

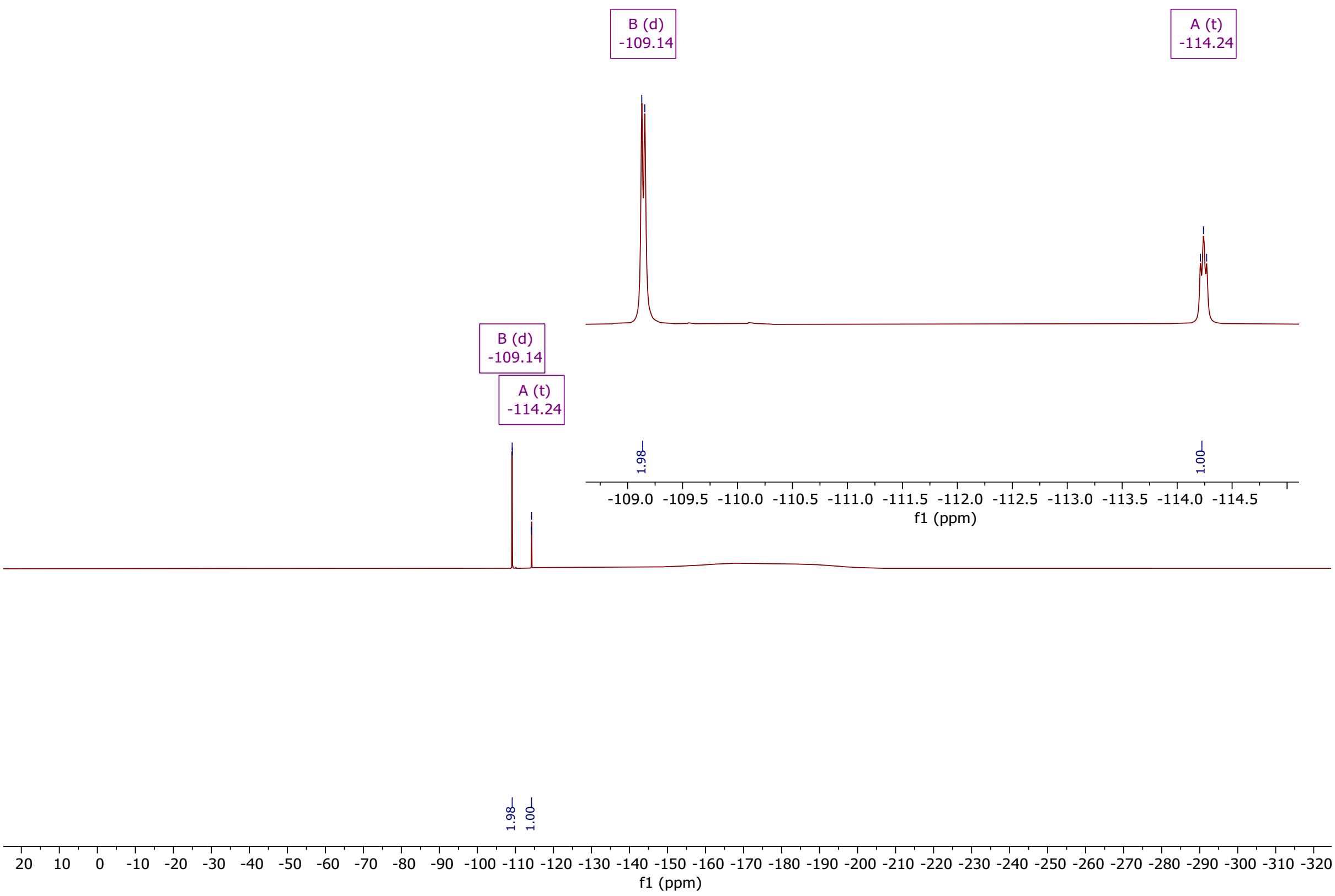

Figure S86 fluorine NMR spectrum of **17**

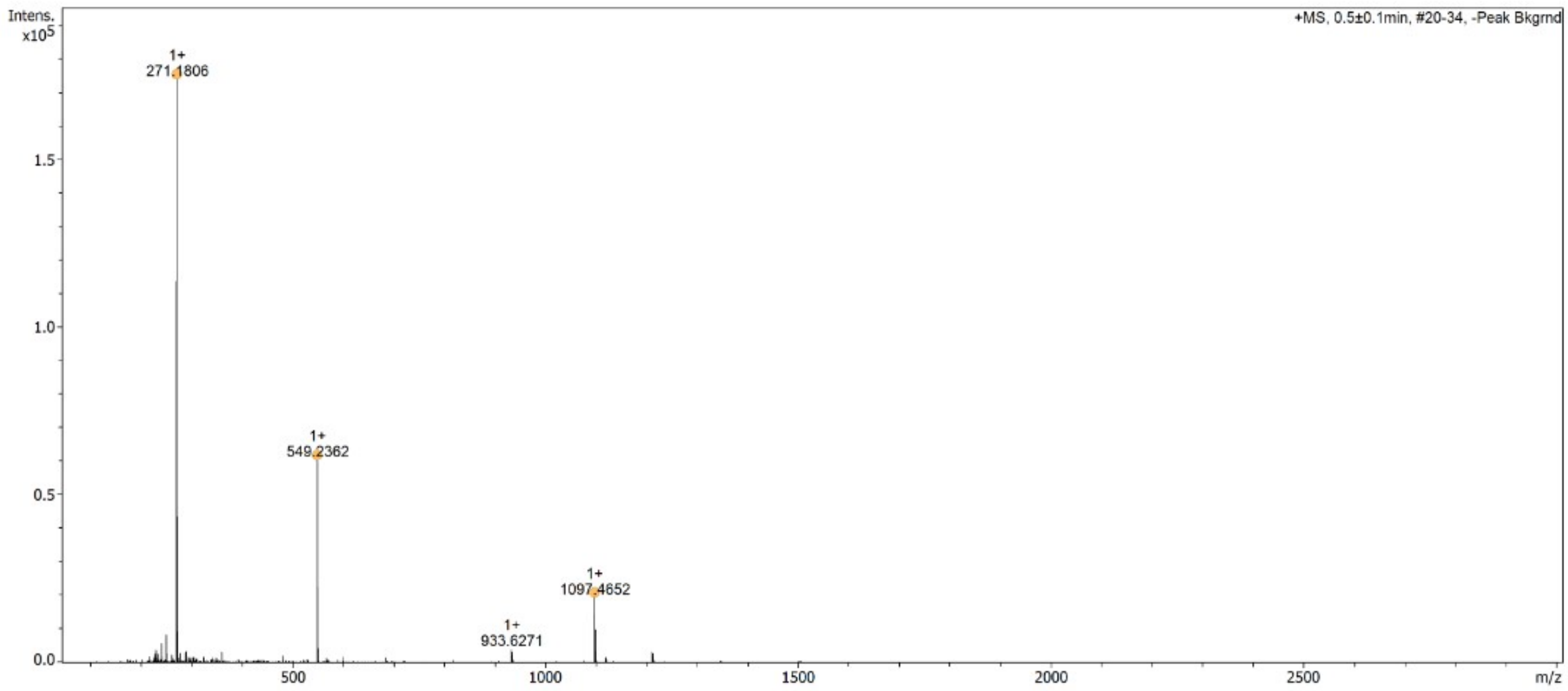

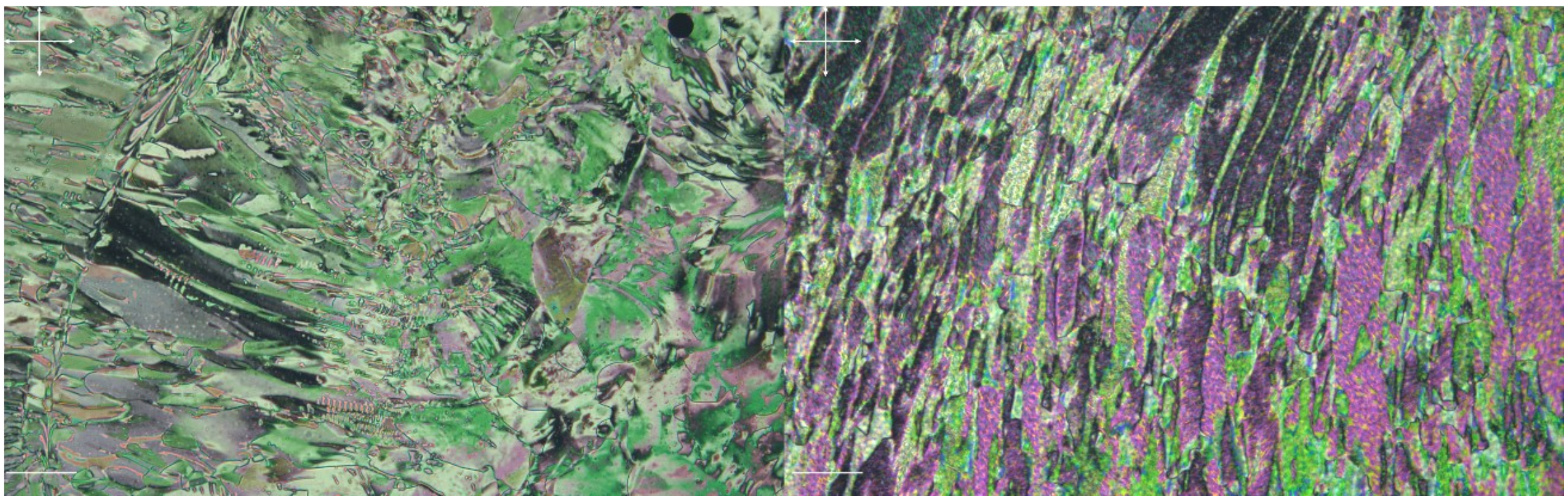

Figure S87 HRMS of **17**

Figure S88 POM image of **17**, both showing N phases at 130 °C (left), and 201 °C (right). Scale bar (bottom-left) shows 1 μm, arrows show polariser direction.

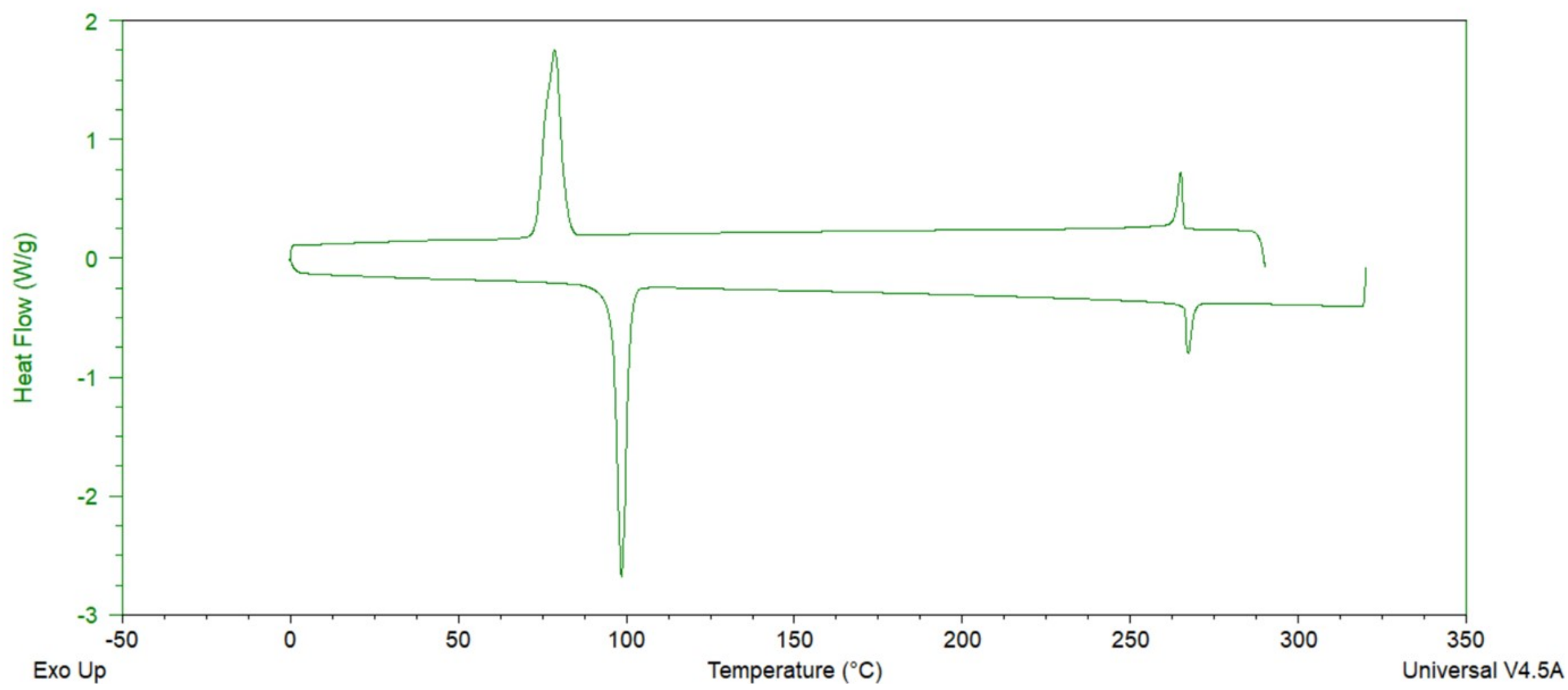

Figure S89 DSC thermogram of **17**.

18 | *3,5-difluoro-4-formylphenyl 4'-ethoxy-2',3,5-trifluoro-[1,1'-biphenyl]-4-carboxylate*

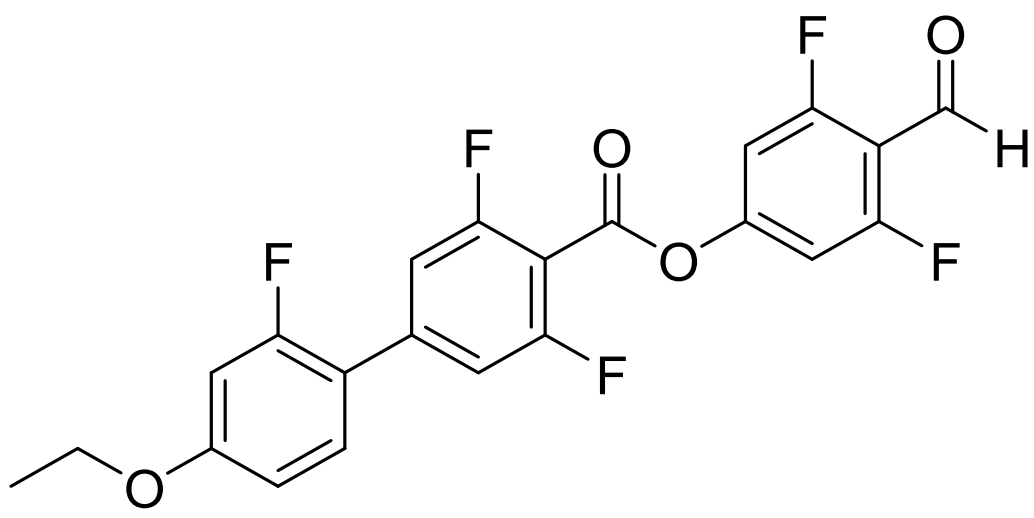

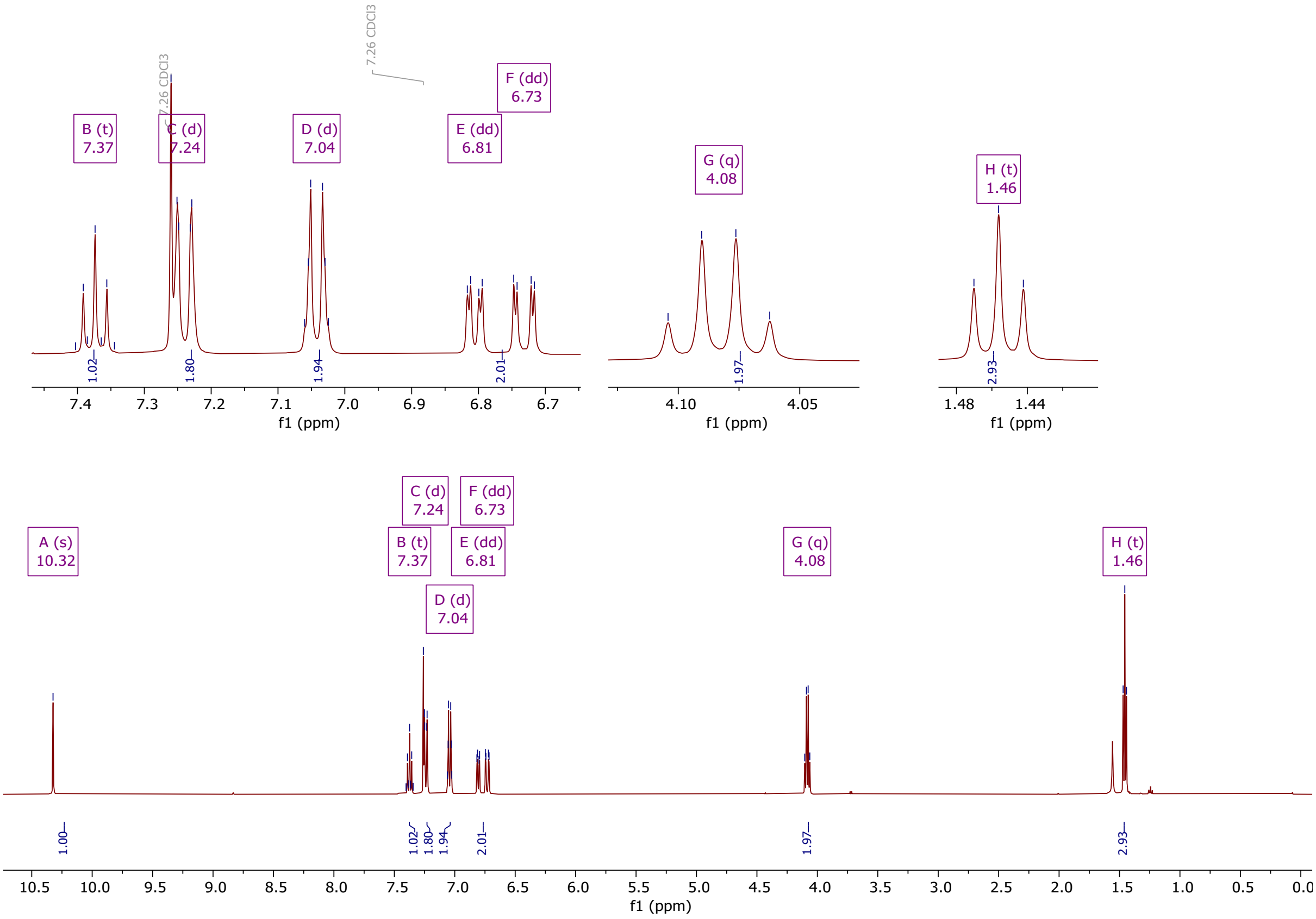

Figure S90 proton NMR spectrum of **18**

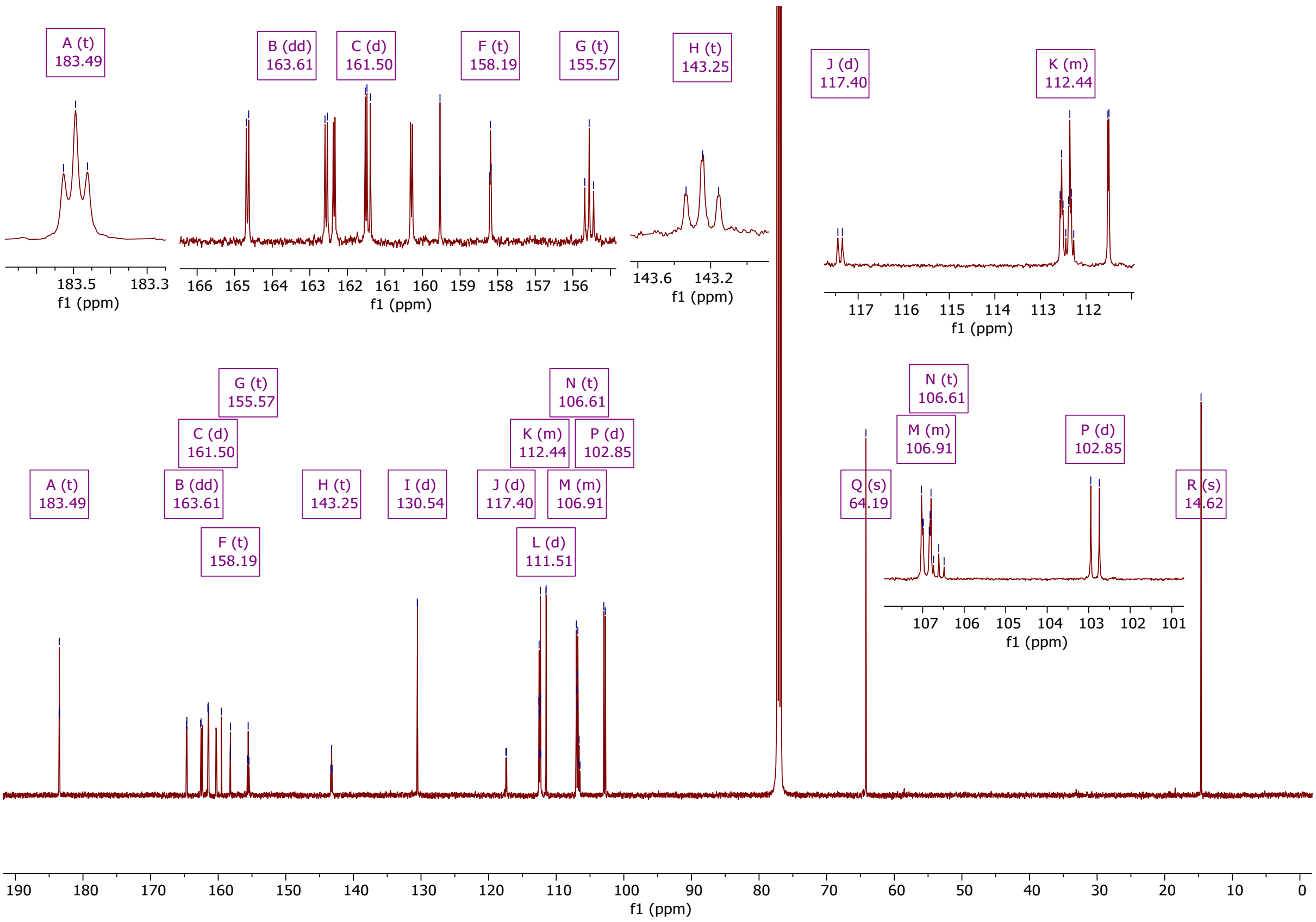

Figure S91 carbon NMR spectrum of **18.** Overlapping C=O, and Ar-F bonds do not have multiple labels but are recorded in data set in section 3.

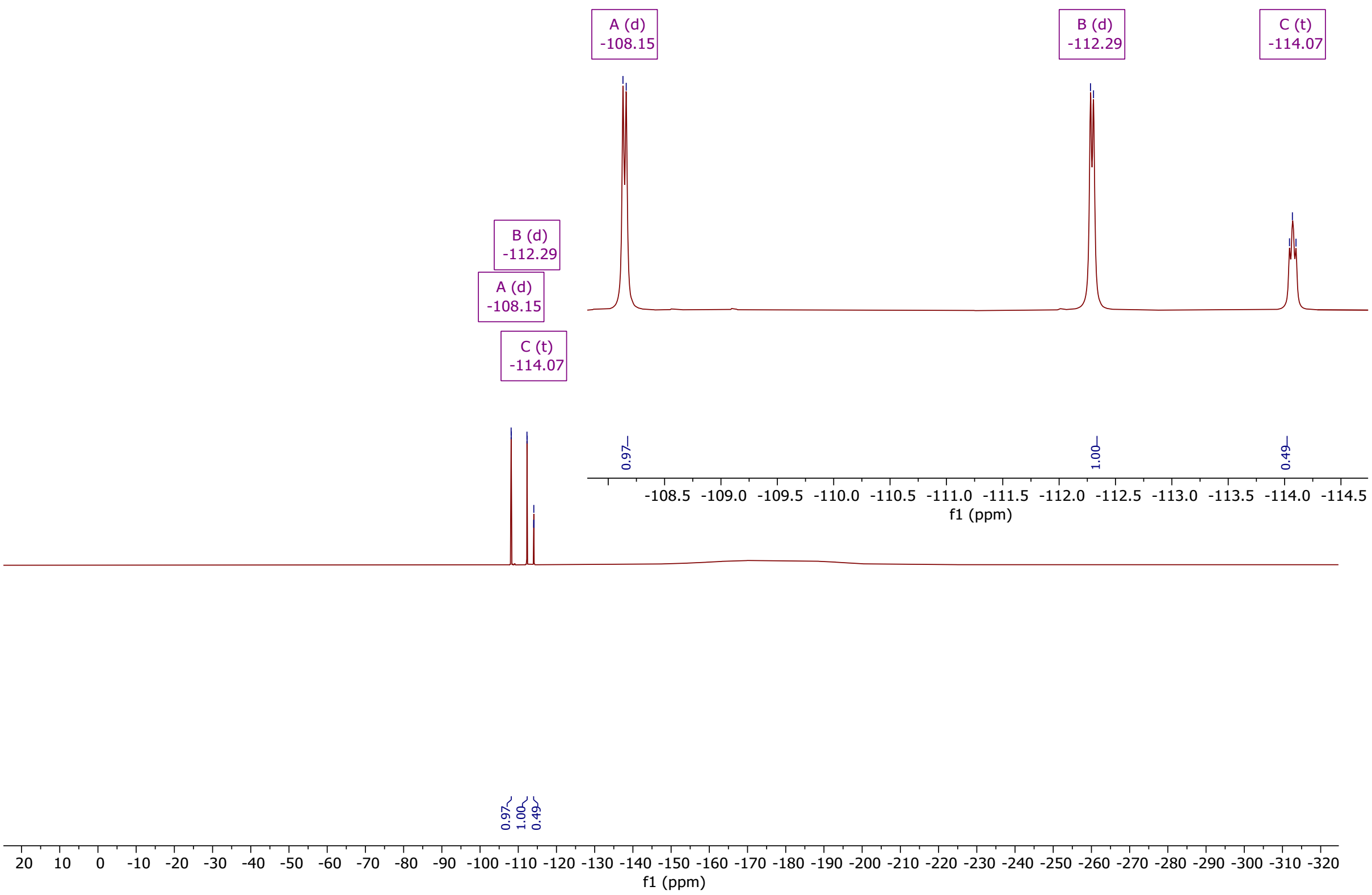

Figure S92 fluorine NMR spectrum of **18**.

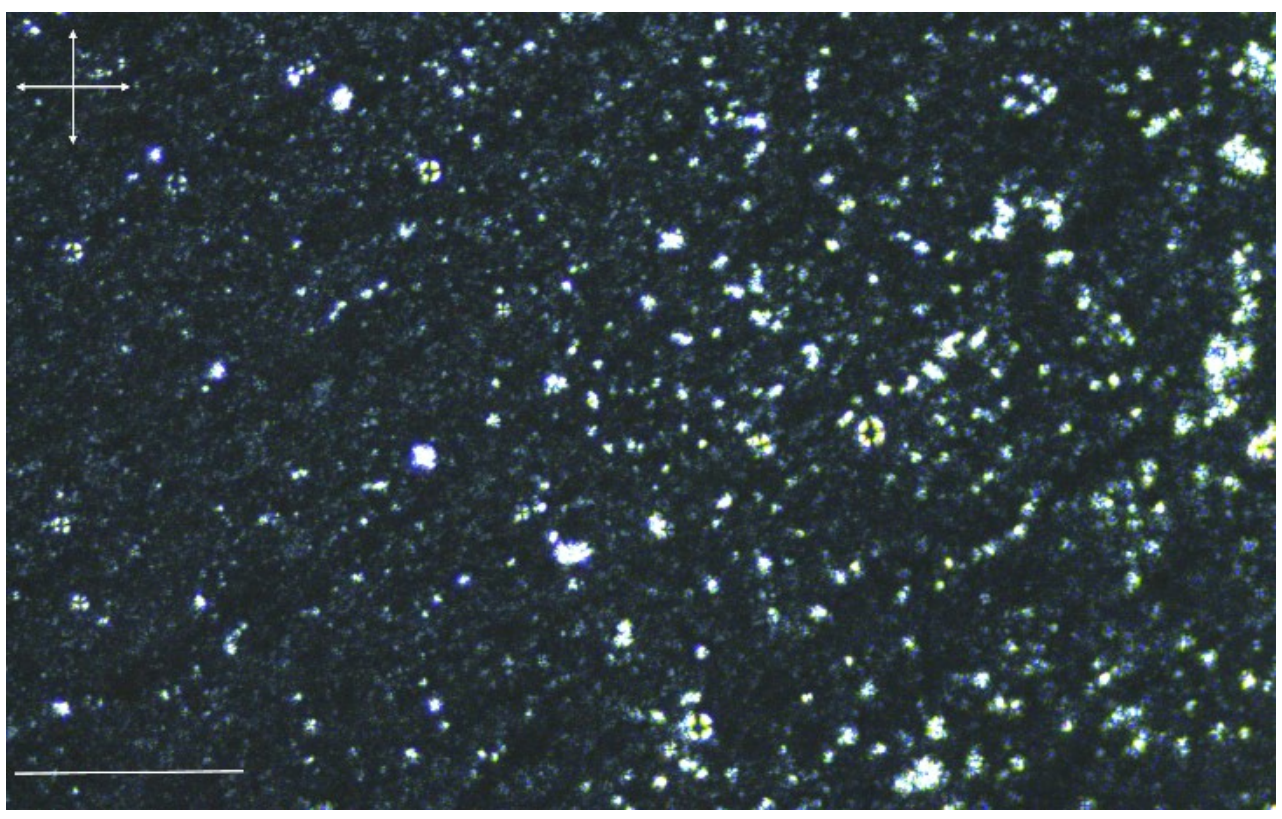

Figure S93 POM image of **18**, N at 126 °C. Scale bar (bottom-left) shows 1 μm, arrows show polariser direction.

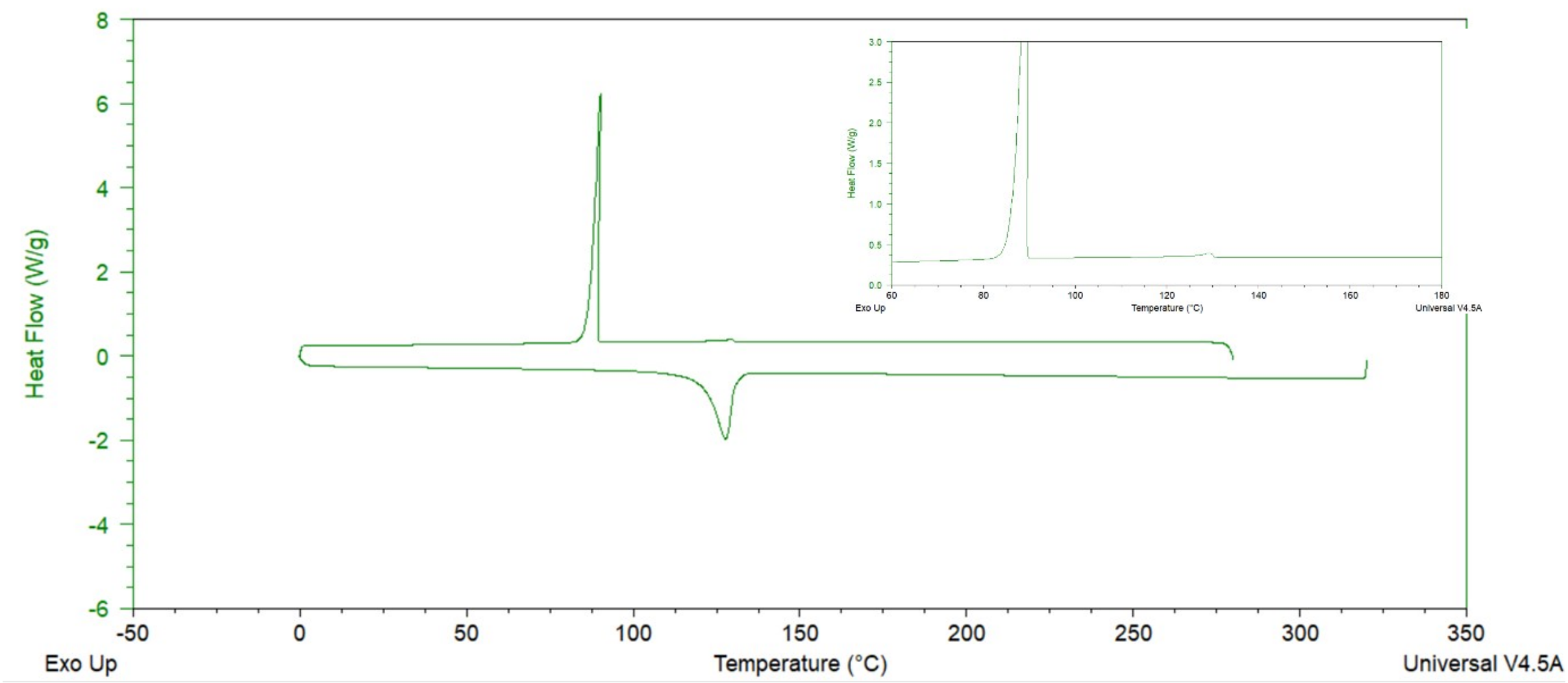

Figure S94 DSC thermogram of **18**.

19 | *4'-(difluoro(3,4,5-trifluorophenoxy)methyl)-2,3',5'-trifluoro-[1,1'-biphenyl]-4-yl 4'-ethoxy-2',3,5-trifluoro-[1,1'-biphenyl]-4-carboxylate*

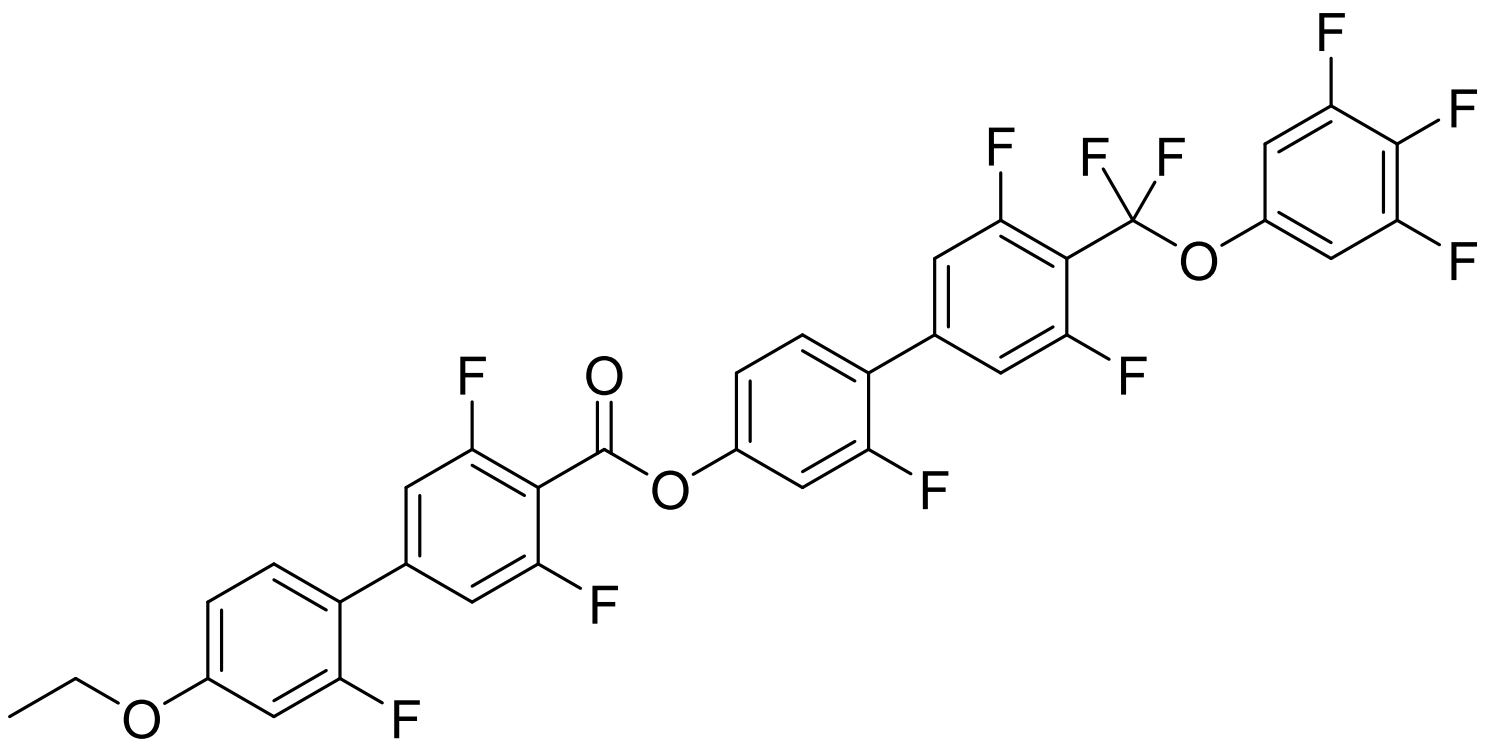

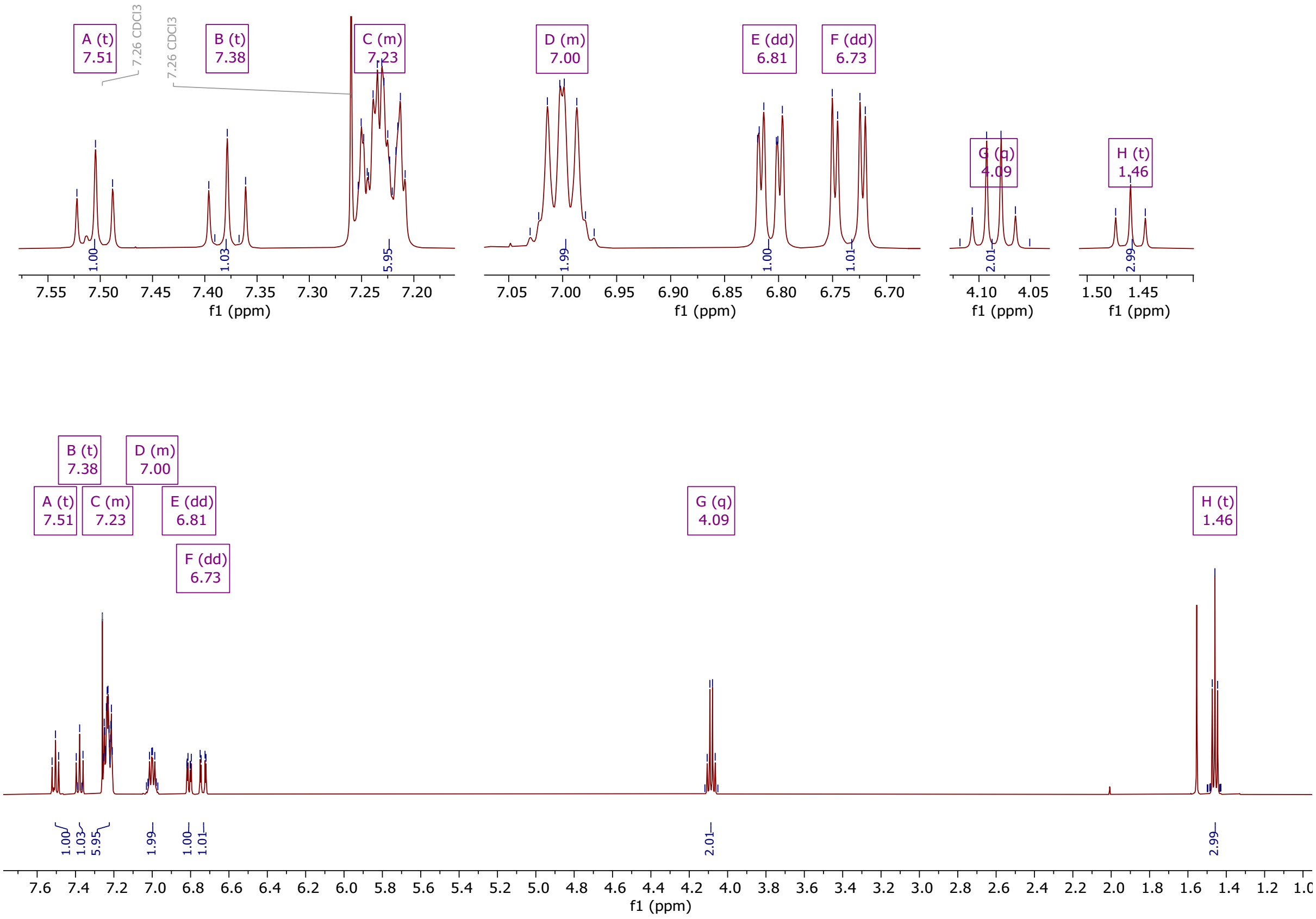

Figure S95 proton NMR spectrum of **19**

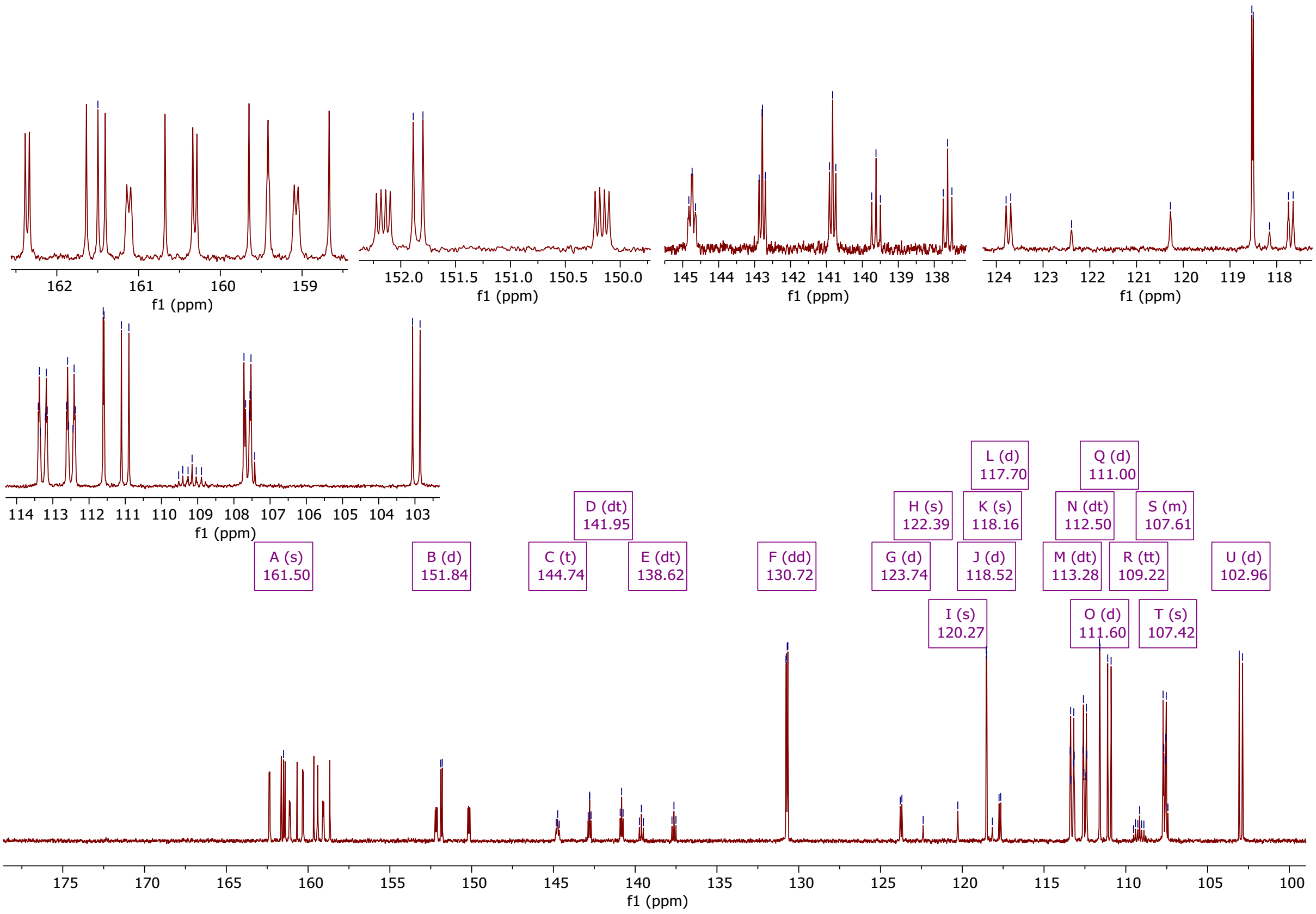

Figure S96 carbon NMR spectrum of **19.** Overlapping C=O, and Ar-F bonds do not have multiple labels but are recorded in data set in section 3.

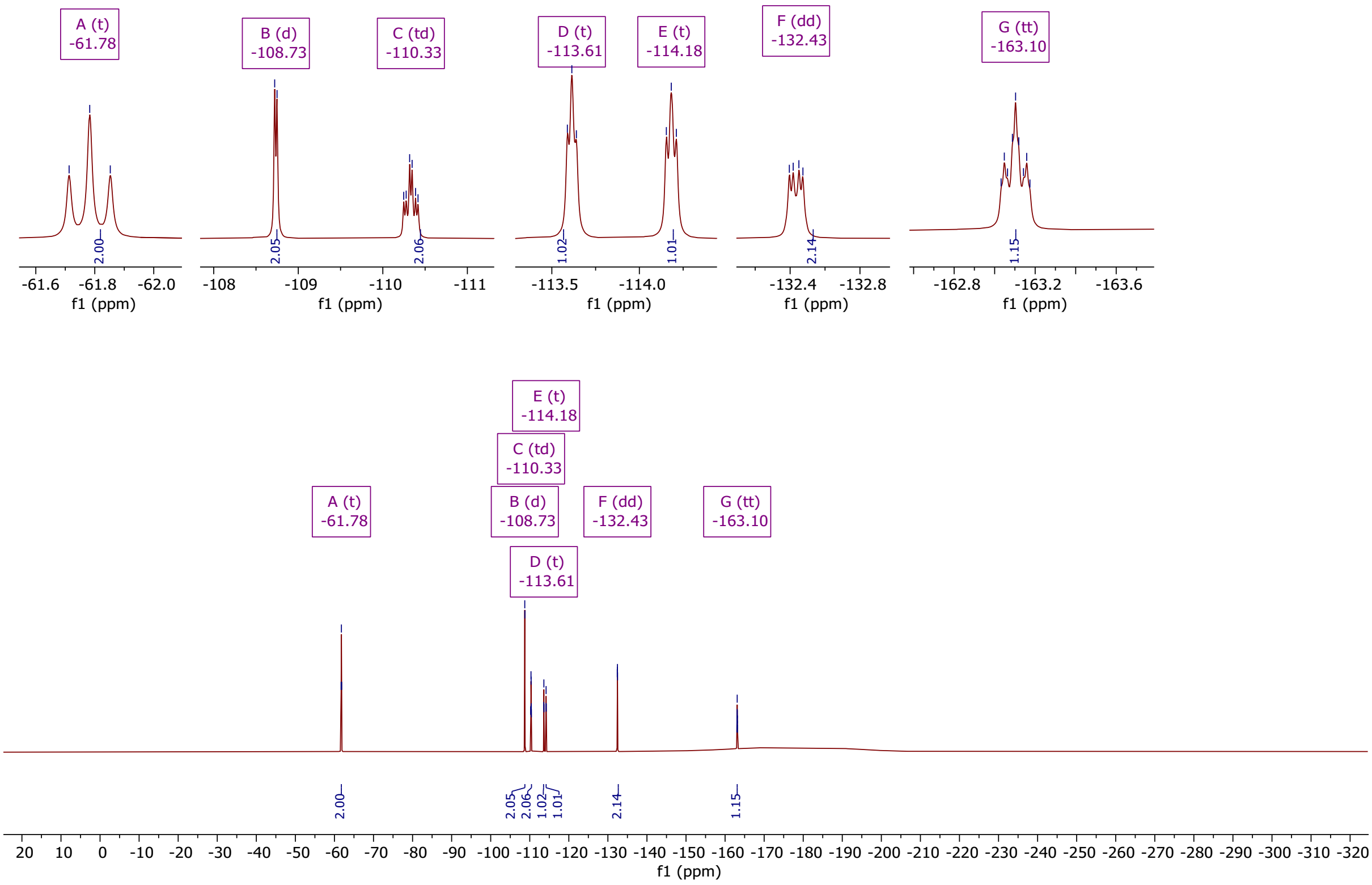

Figure S97 fluorine NMR spectrum of **19**

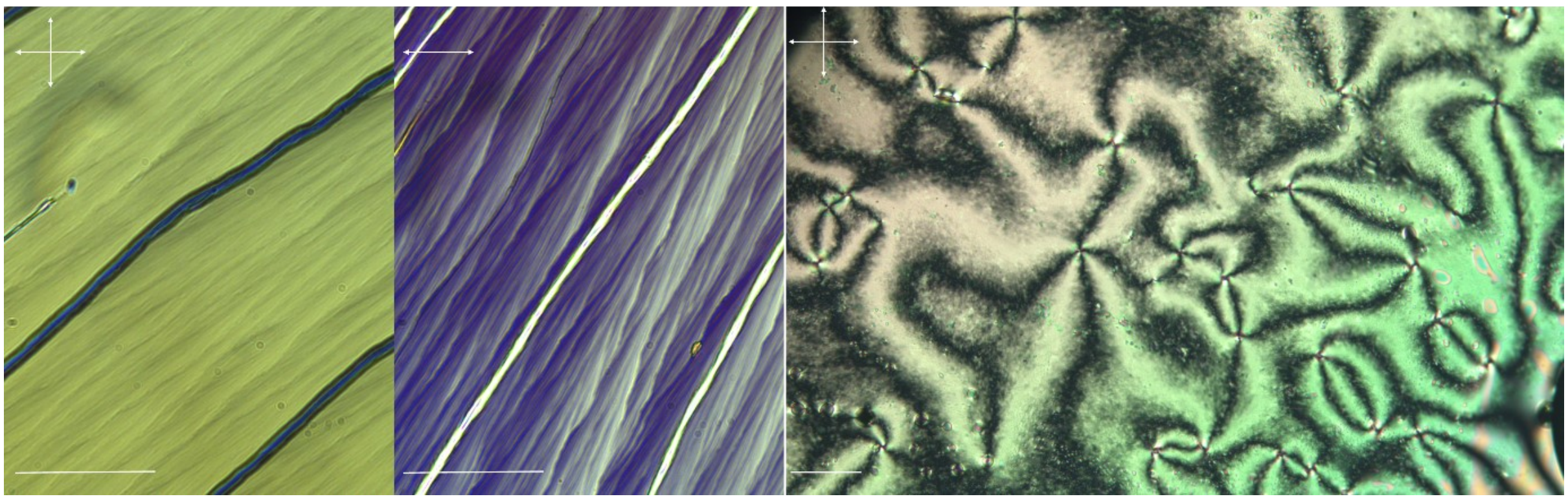

Figure S98 POM images of **19**, SmX at 90 °C with crossed polarisers (left), SmX at 56 °C with aligned polarisers (central), N at 252 °C (right). Scale bar (bottom-left) shows 1 μm, arrows show polariser direction.

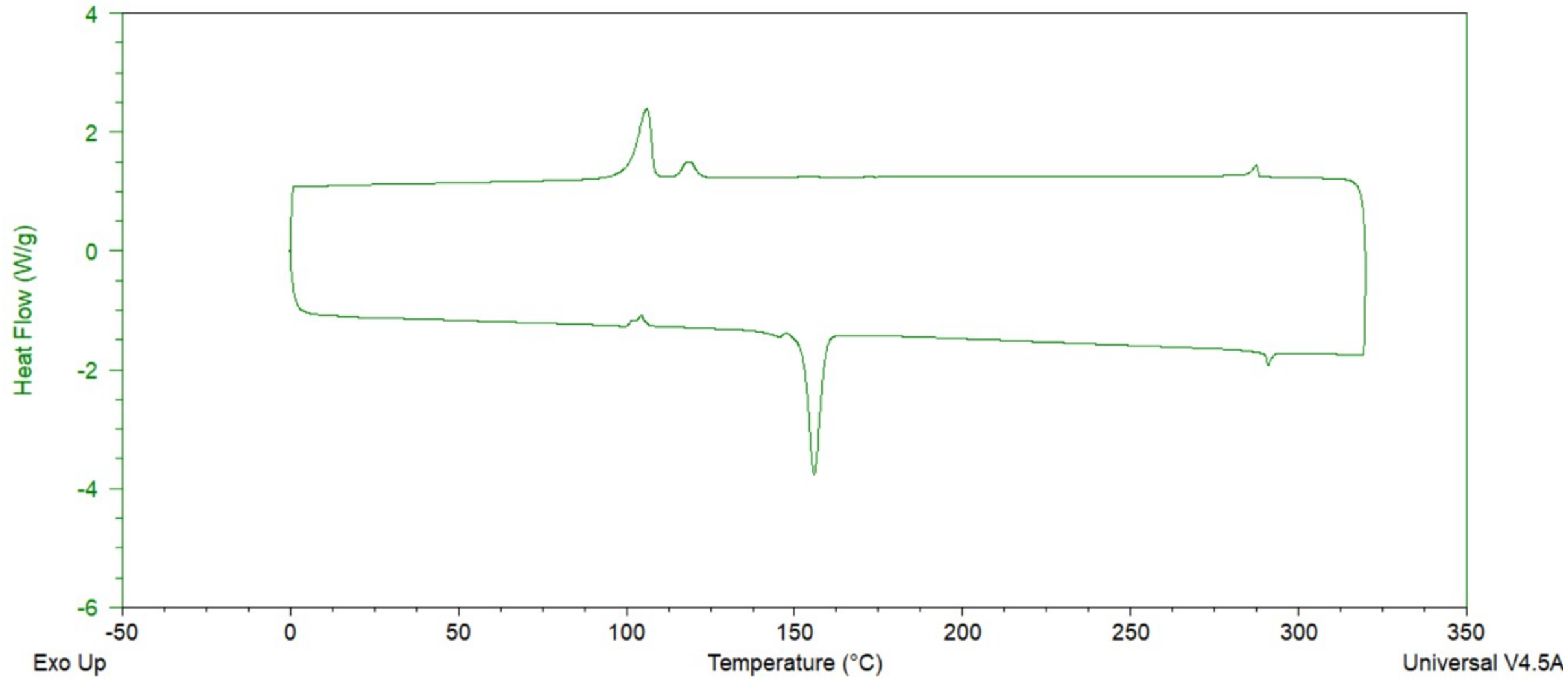

Figure S99 DSC thermogram of **19**.

20 | *4'-(difluoro(3,4,5-trifluorophenoxy)methyl)-2,3',5'-trifluoro-[1,1'-biphenyl]-4-yl 3,5-difluoro-2',4'-dimethoxy-[1,1'-biphenyl]-4-carboxylate*

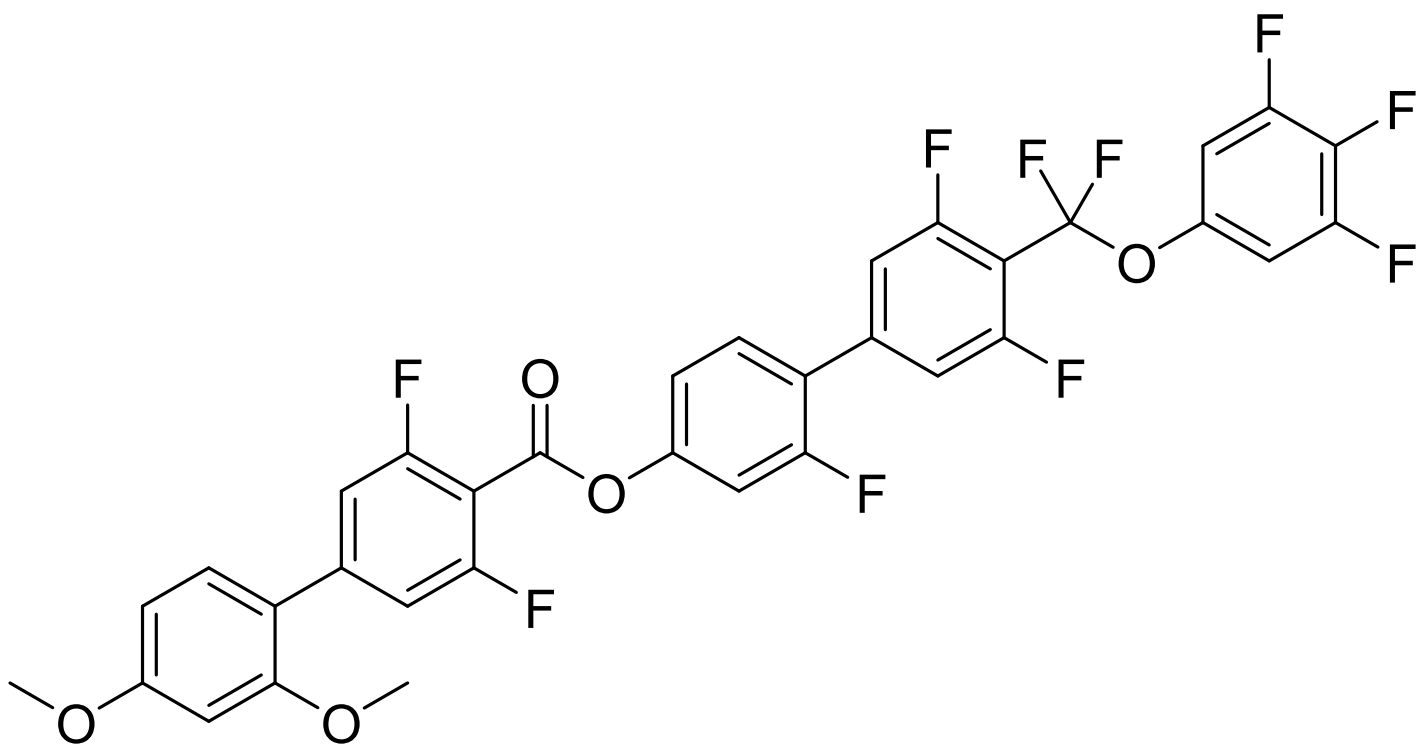

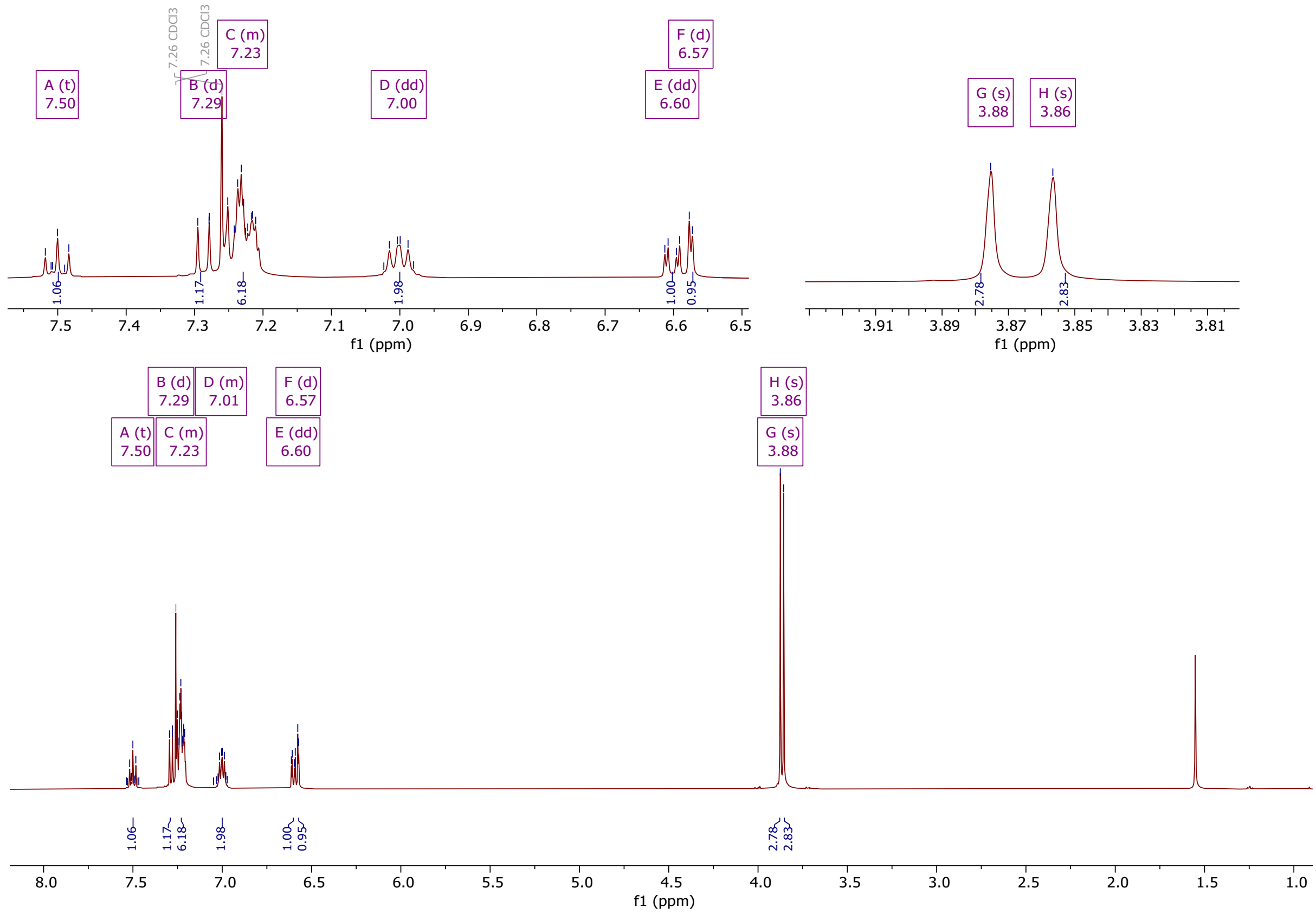

Figure S100 proton NMR spectrum of **20**

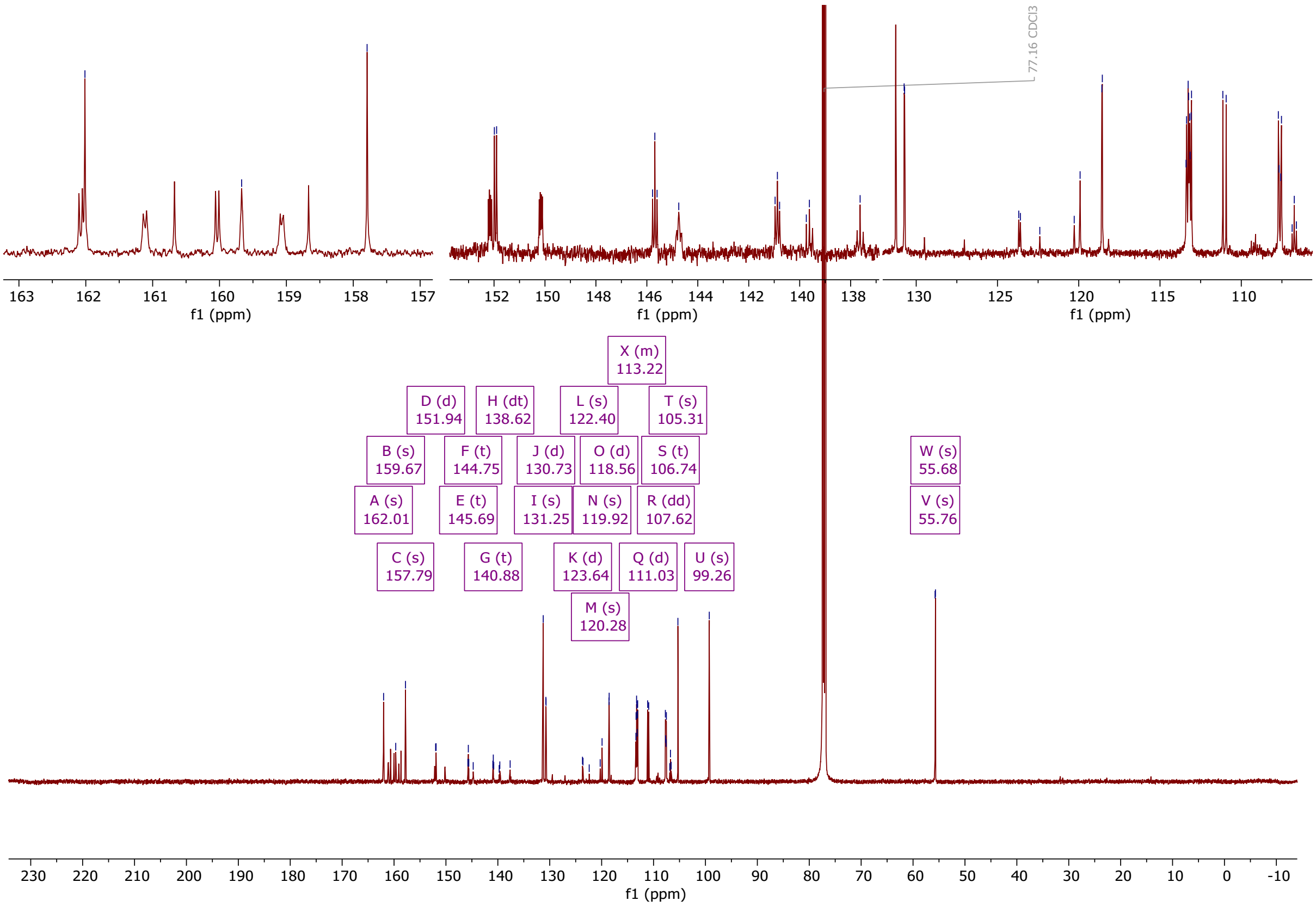

Figure S101 carbon NMR spectrum of **20.** Overlapping C=O, and Ar-F bonds do not have multiple labels but are recorded in data set in section 3.

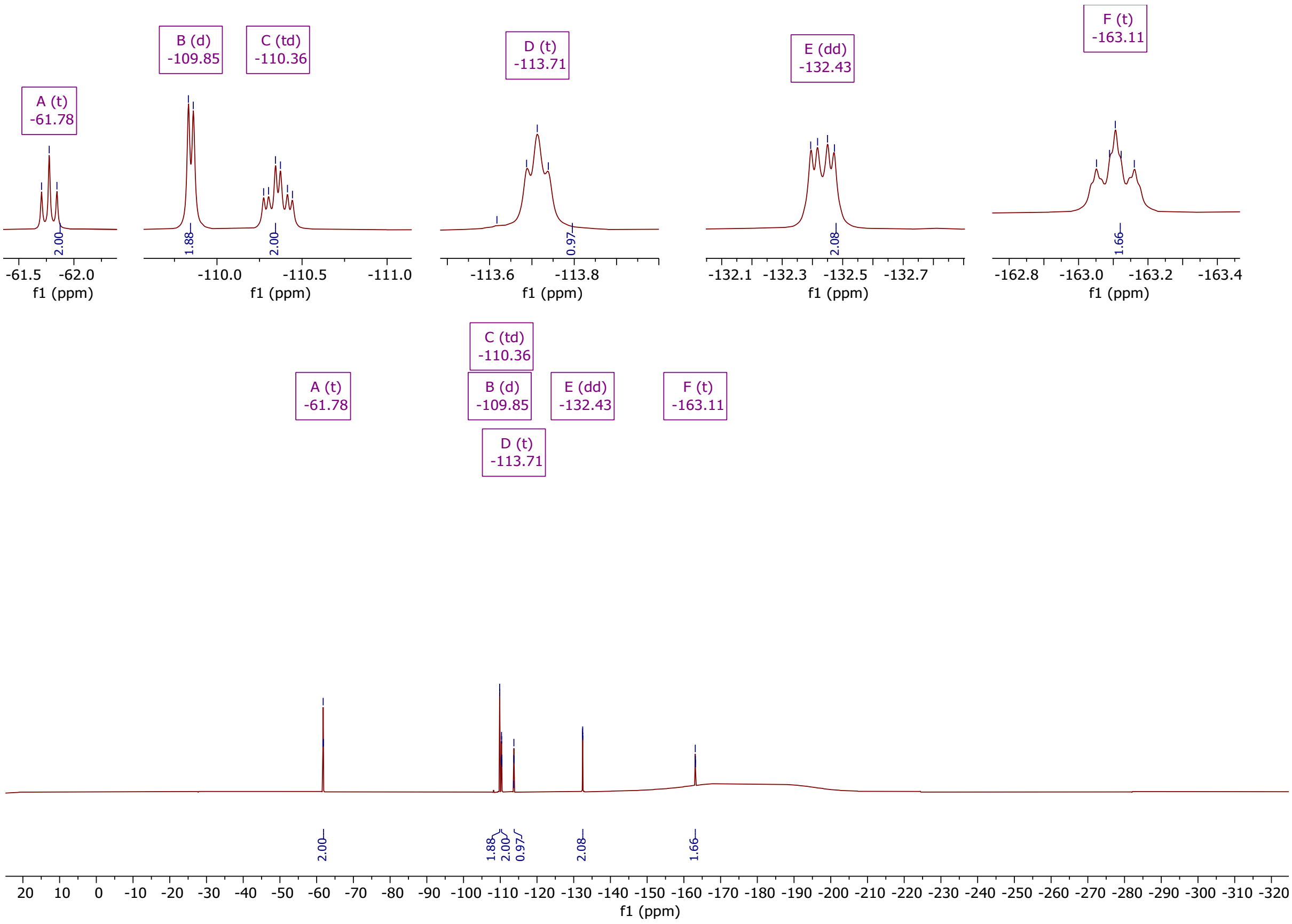

Figure S102 fluorine NMR spectrum of **20**.

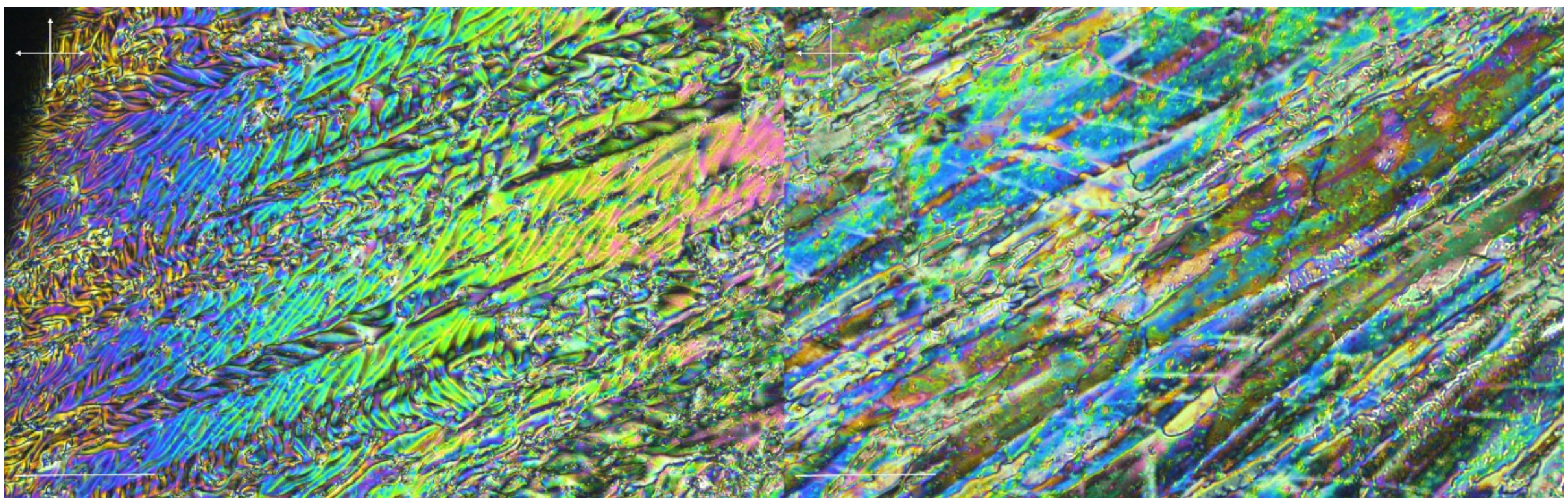

Figure S103 POM image of **20**, N_f phase at 92 °C (left), N phase at 150 °C (right). Scale bar (bottom-left) shows 1 μm, arrows show polariser direction.

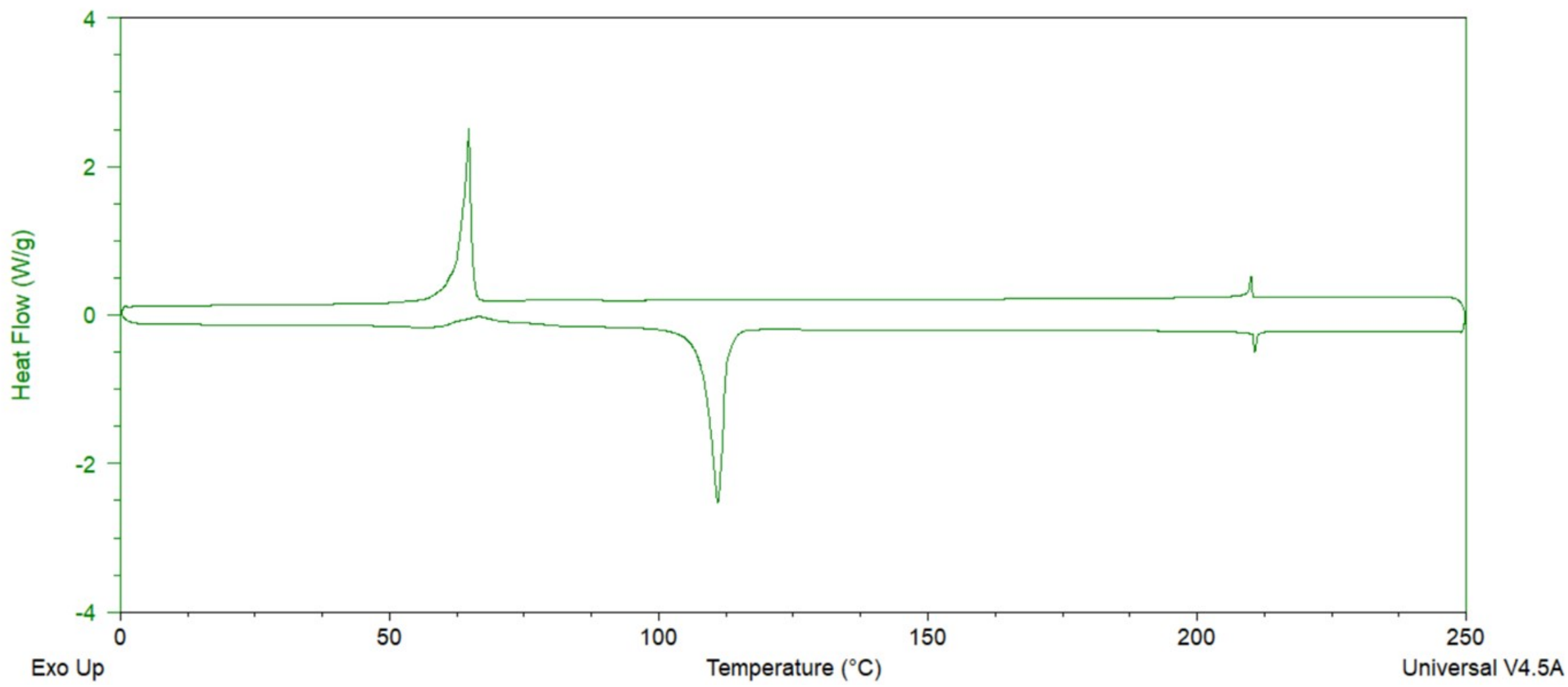

Figure S104 DSC thermogram of **20**.

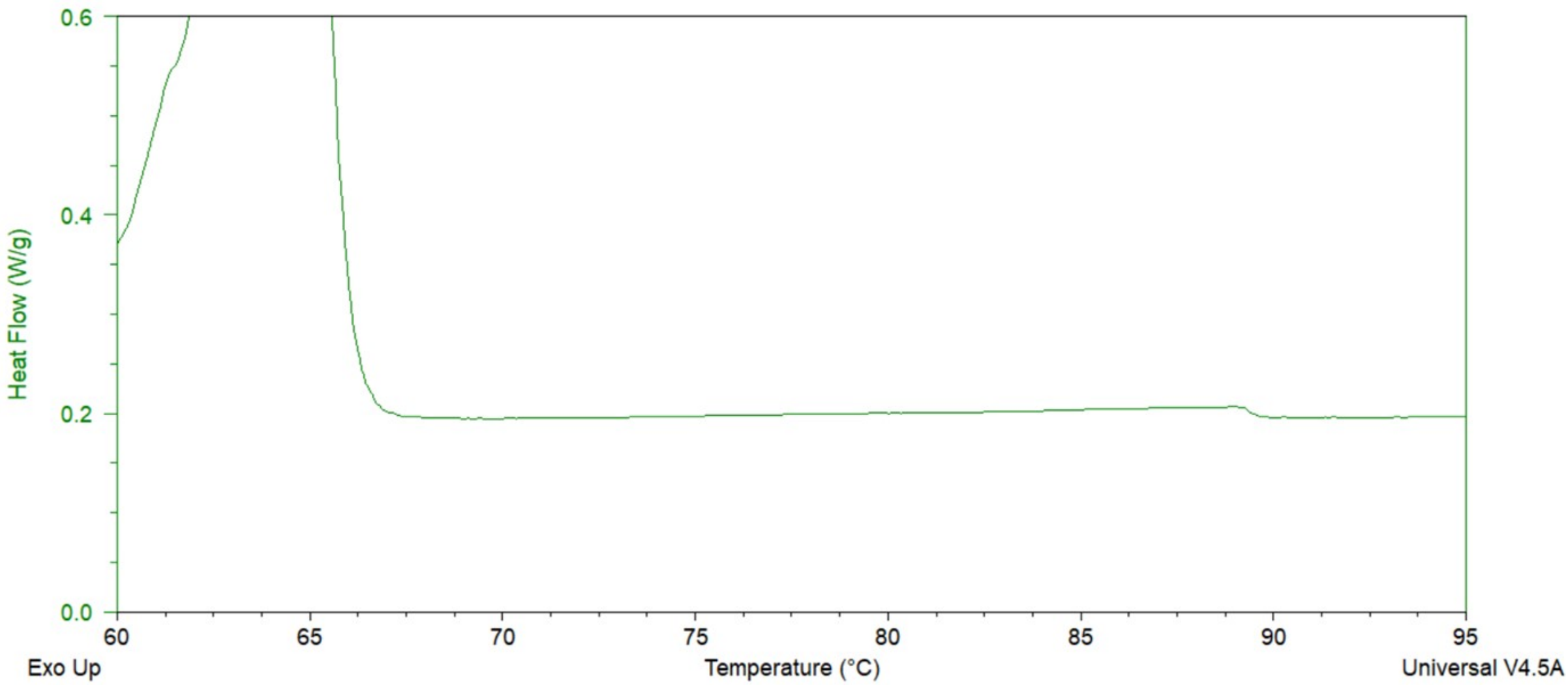

Figure S105 Zoomed in DSC thermogram of **20** showing the N_f transition.

21 | *4-(ethoxycarbonyl)phenyl 2,3-dihydrobenzo[b][1,4]dioxine-6-carboxylate*

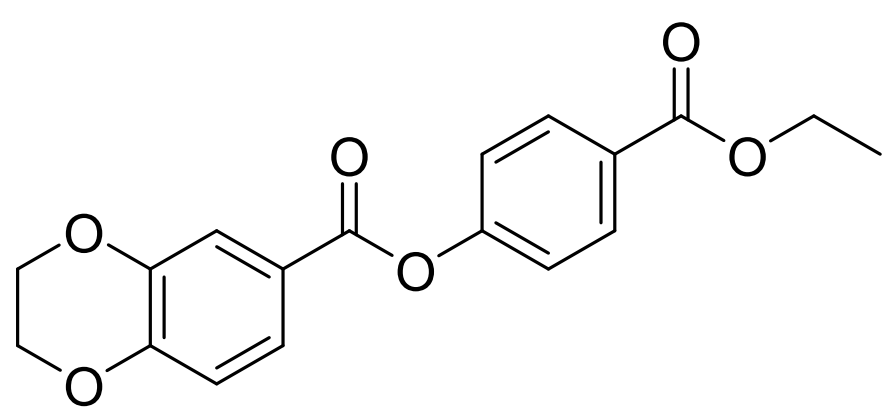

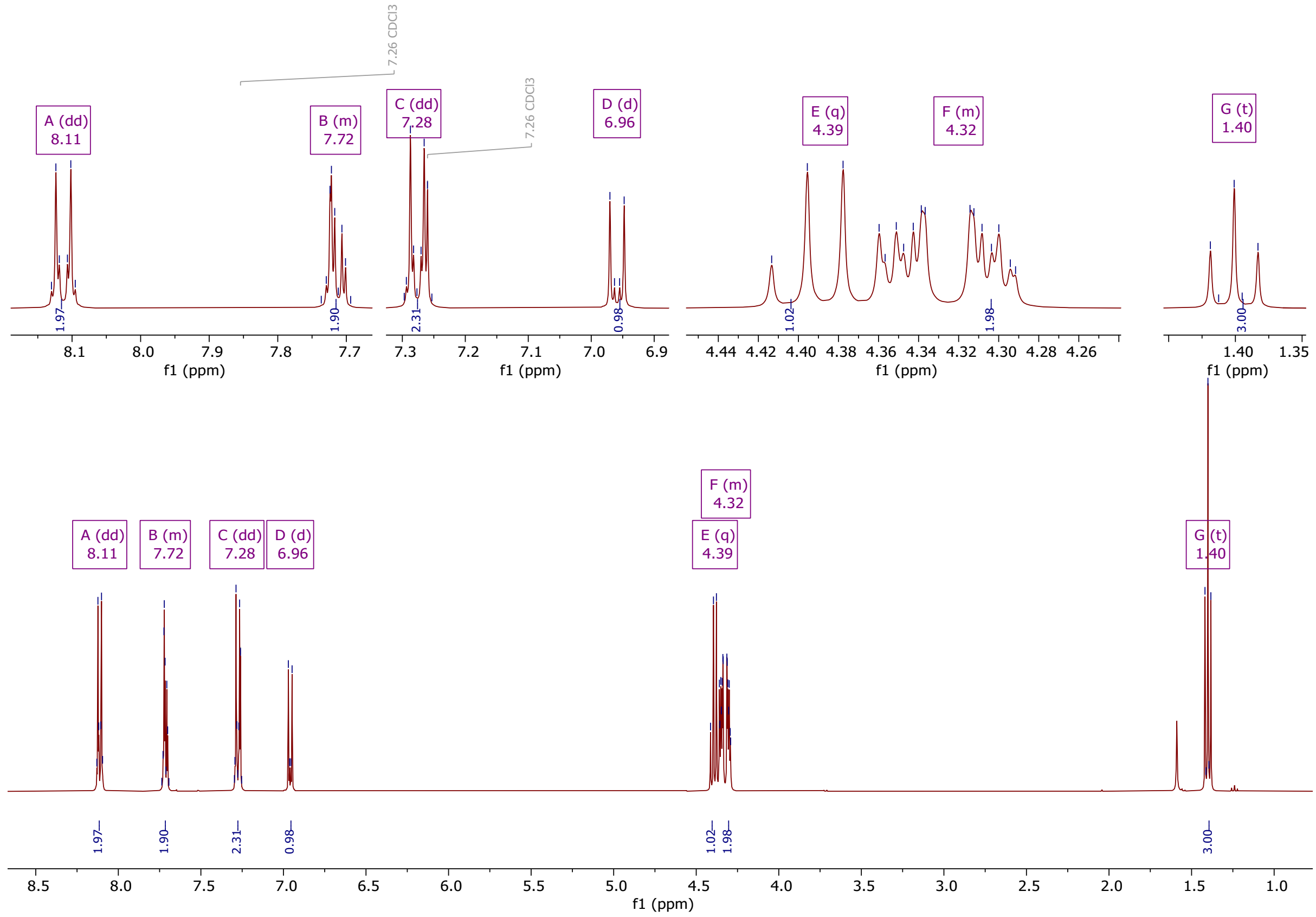

Figure S106 proton NMR spectrum of **21**

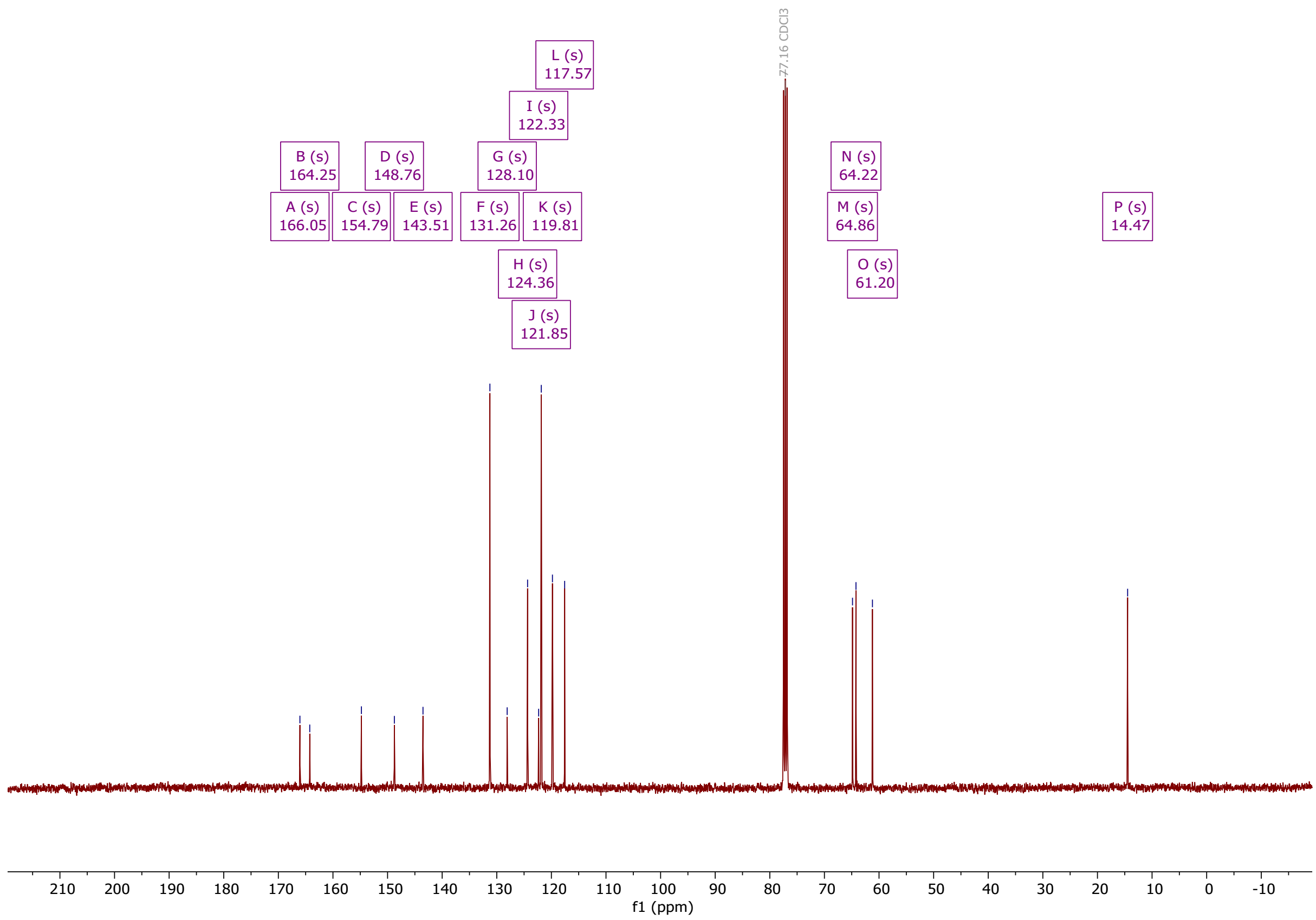

Figure S107 carbon NMR spectrum of **21**.

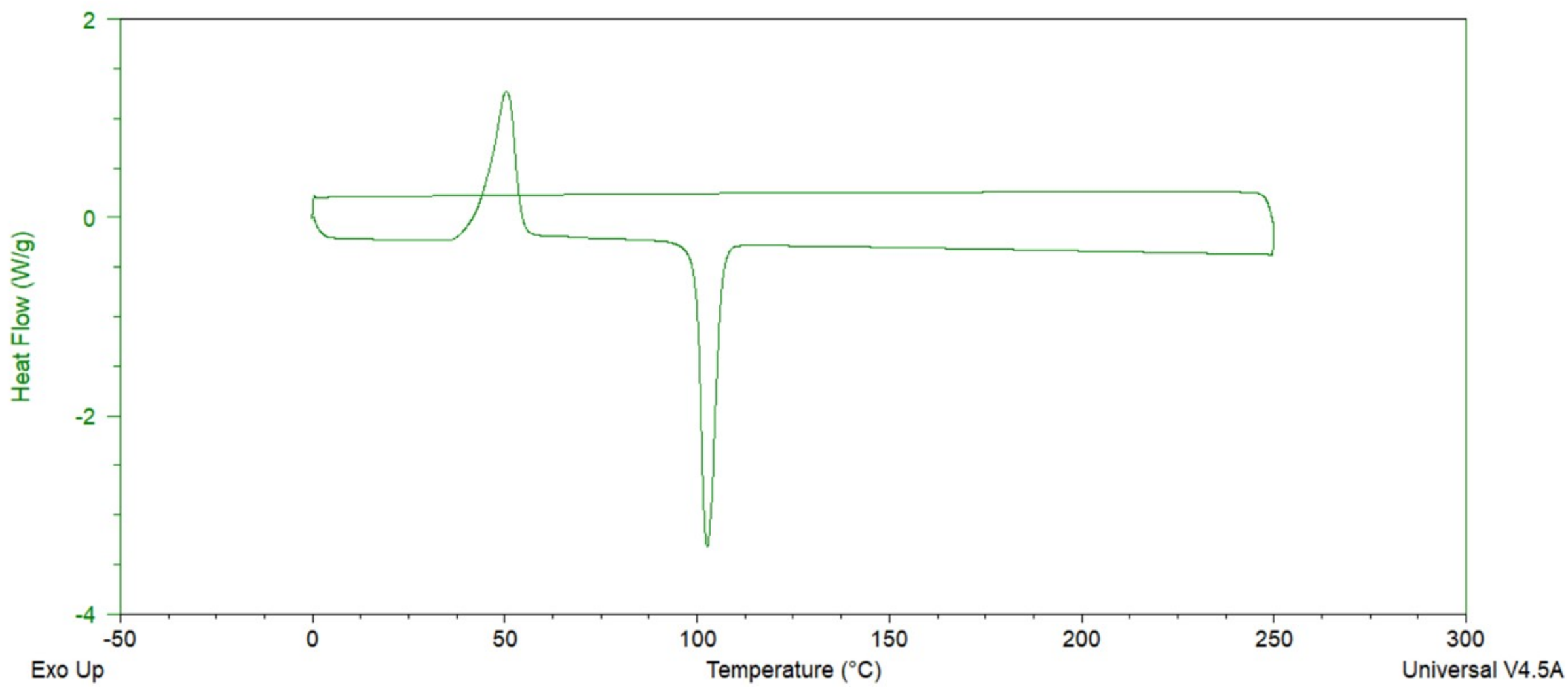

Figure S108 DSC thermogram of **21**.

22 | *4-cyanophenyl (E)-3-(2,4-dimethoxyphenyl)acrylate*

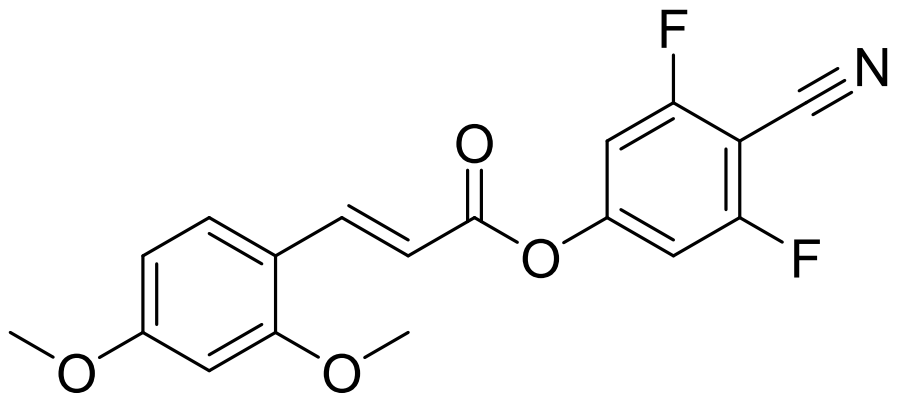

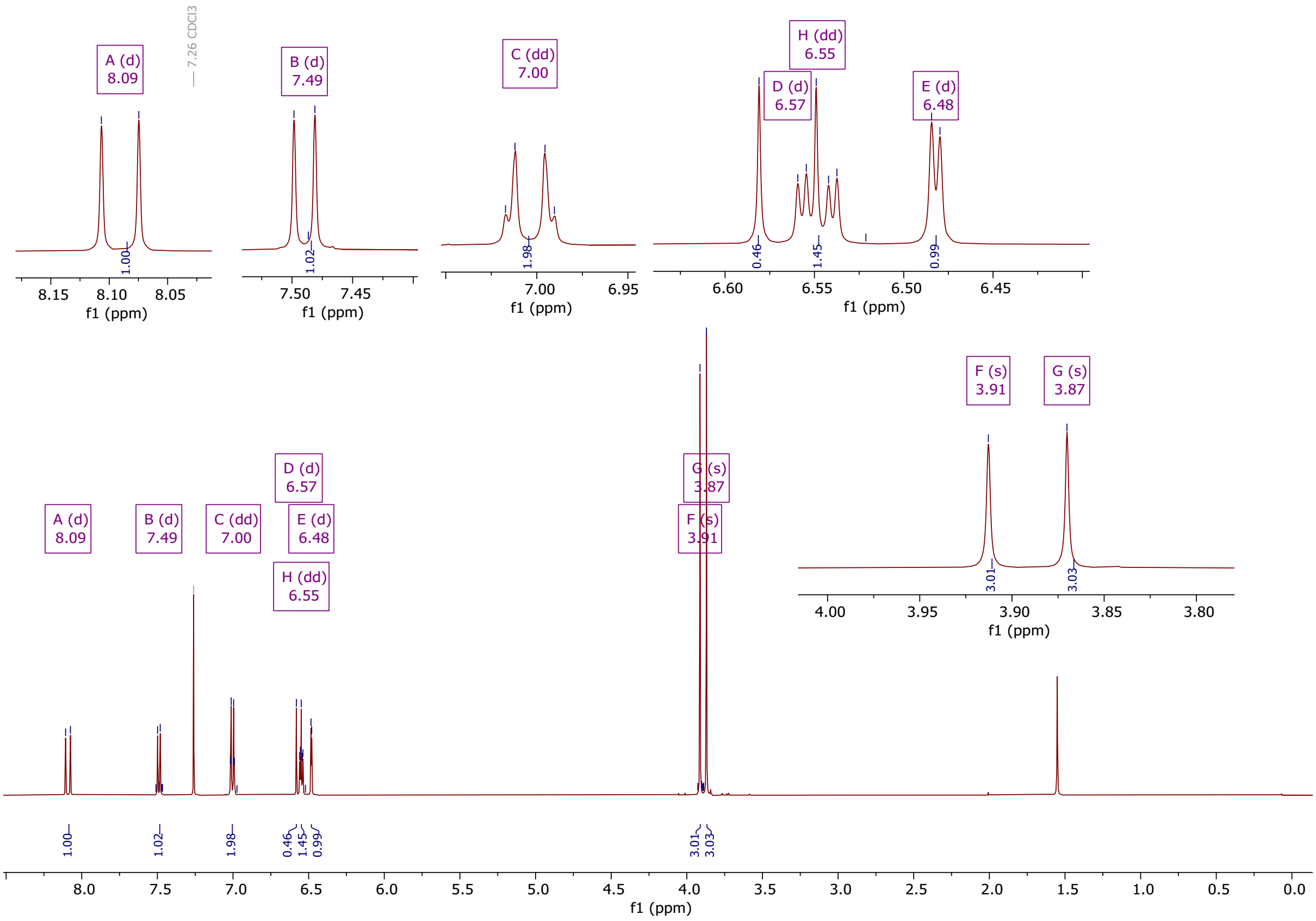

Figure S109 proton NMR spectrum of **22**

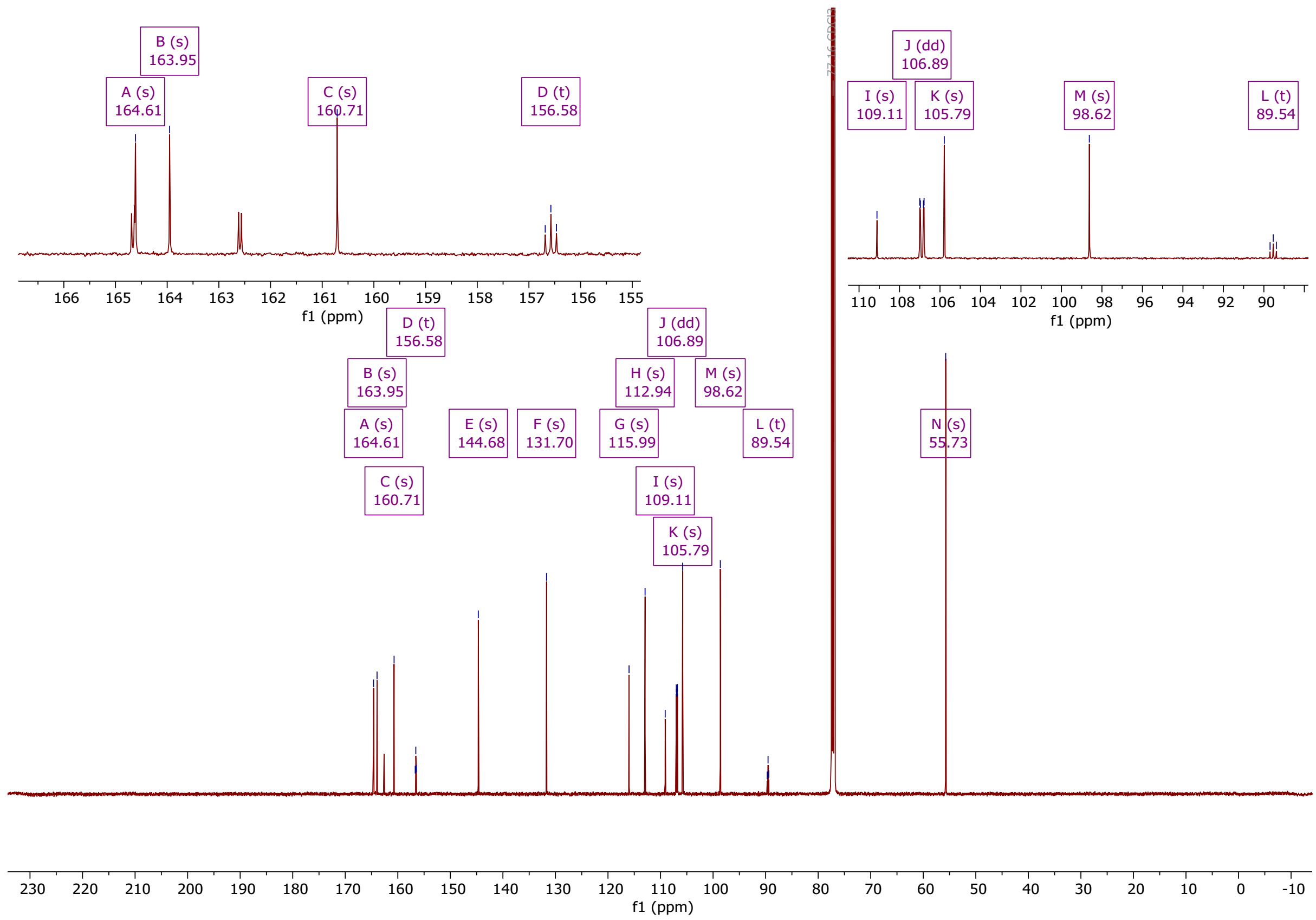

Figure S110 carbon NMR spectrum of **22**

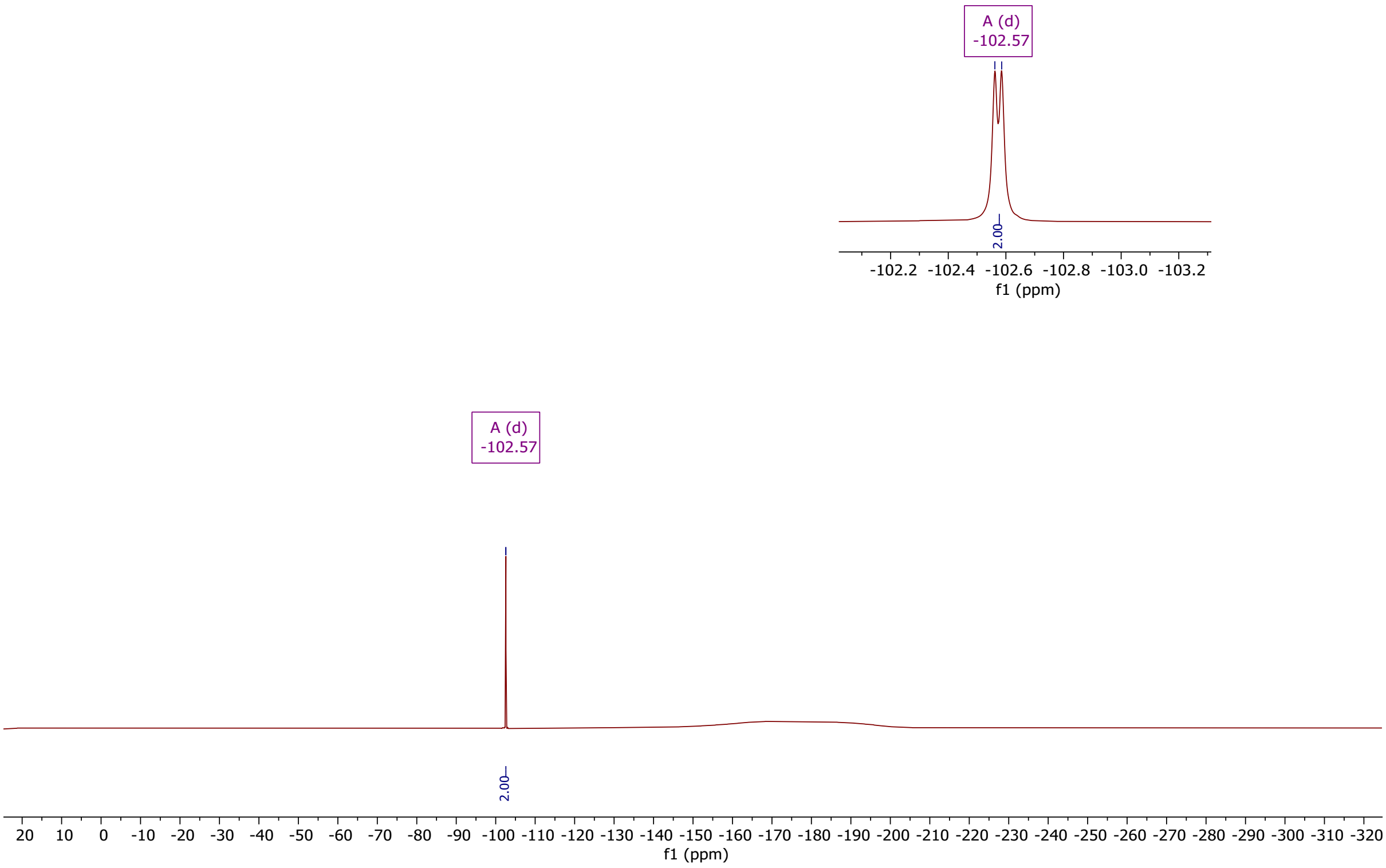

Figure S111 fluorine NMR spectrum of **22**

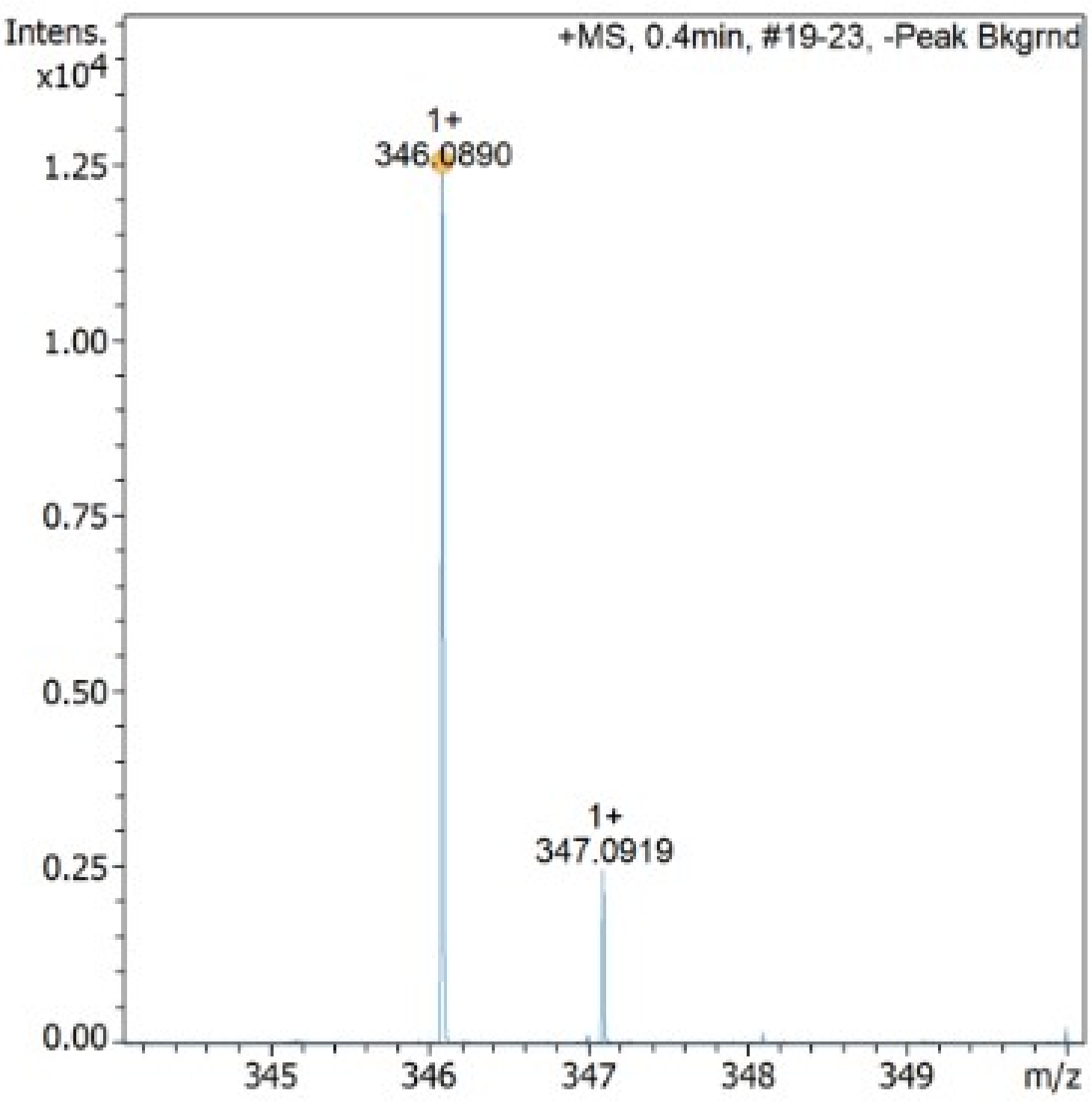

Figure S112 HRMS of **22.**

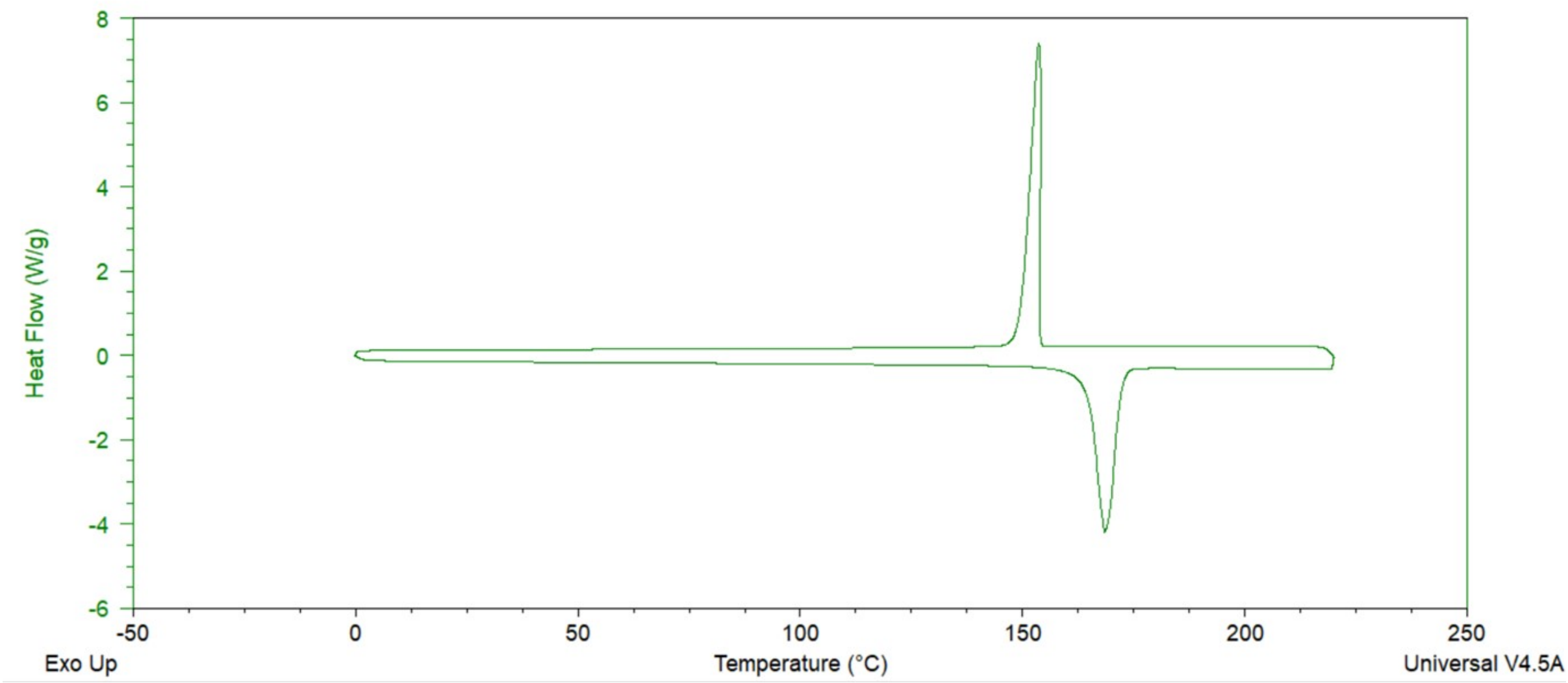

Figure S113 DSC thermogram of **22**.

23 | *4'-cyano-2,3',6-trifluoro-[1,1'-biphenyl]-4-yl 2-methoxy-4-(trifluoromethoxy)benzoate*

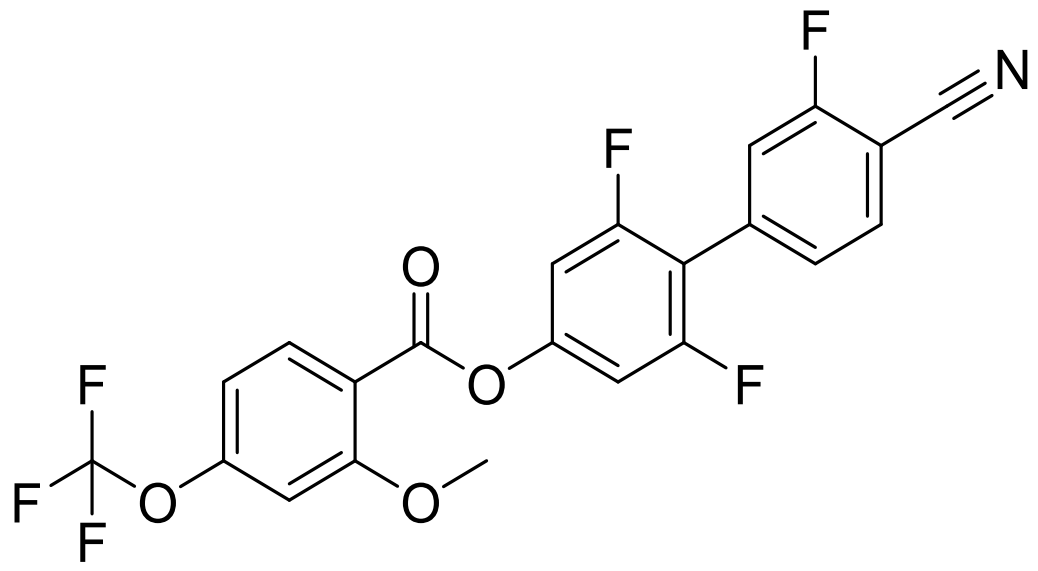

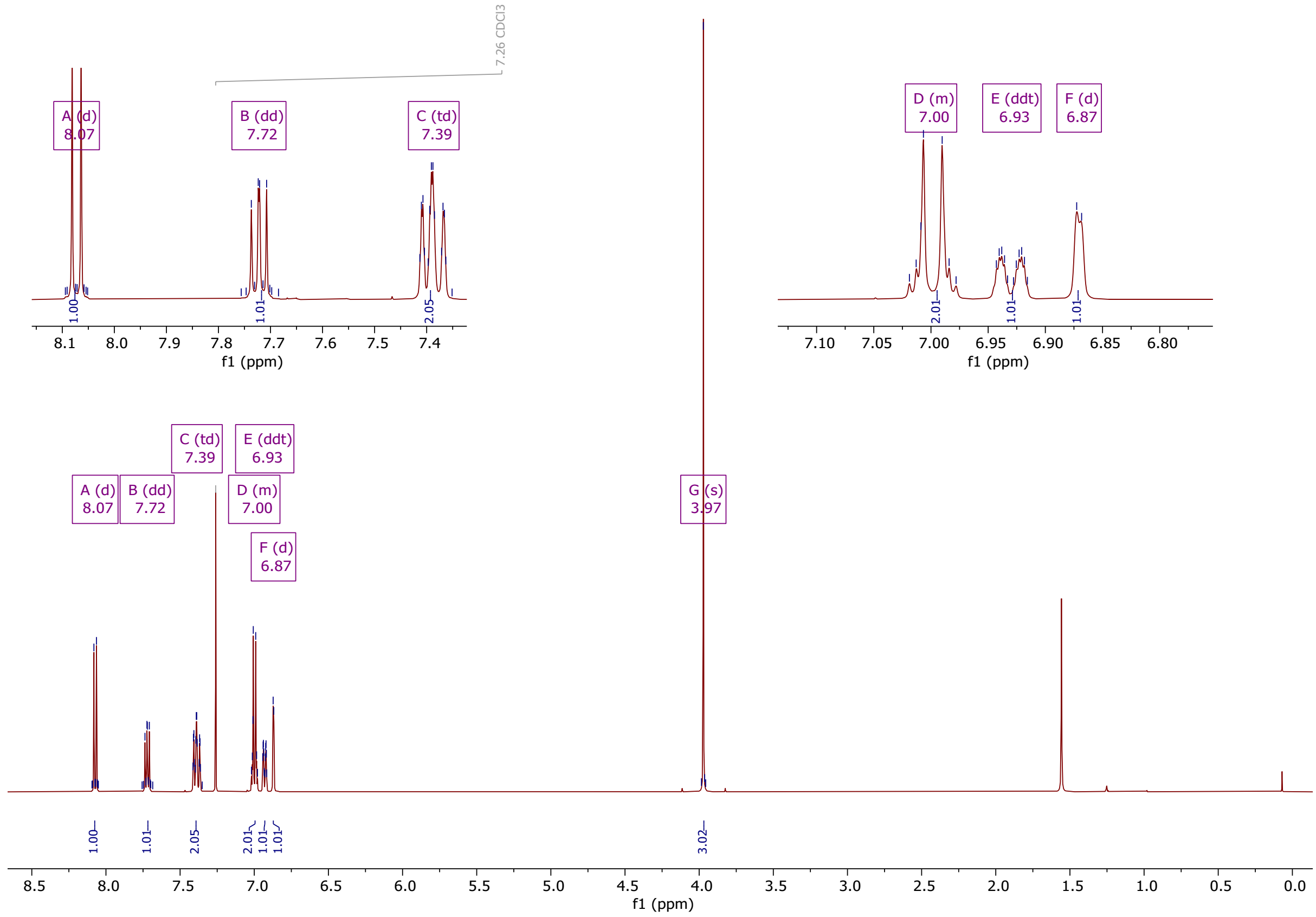

Figure S114 proton NMR spectrum of **23**

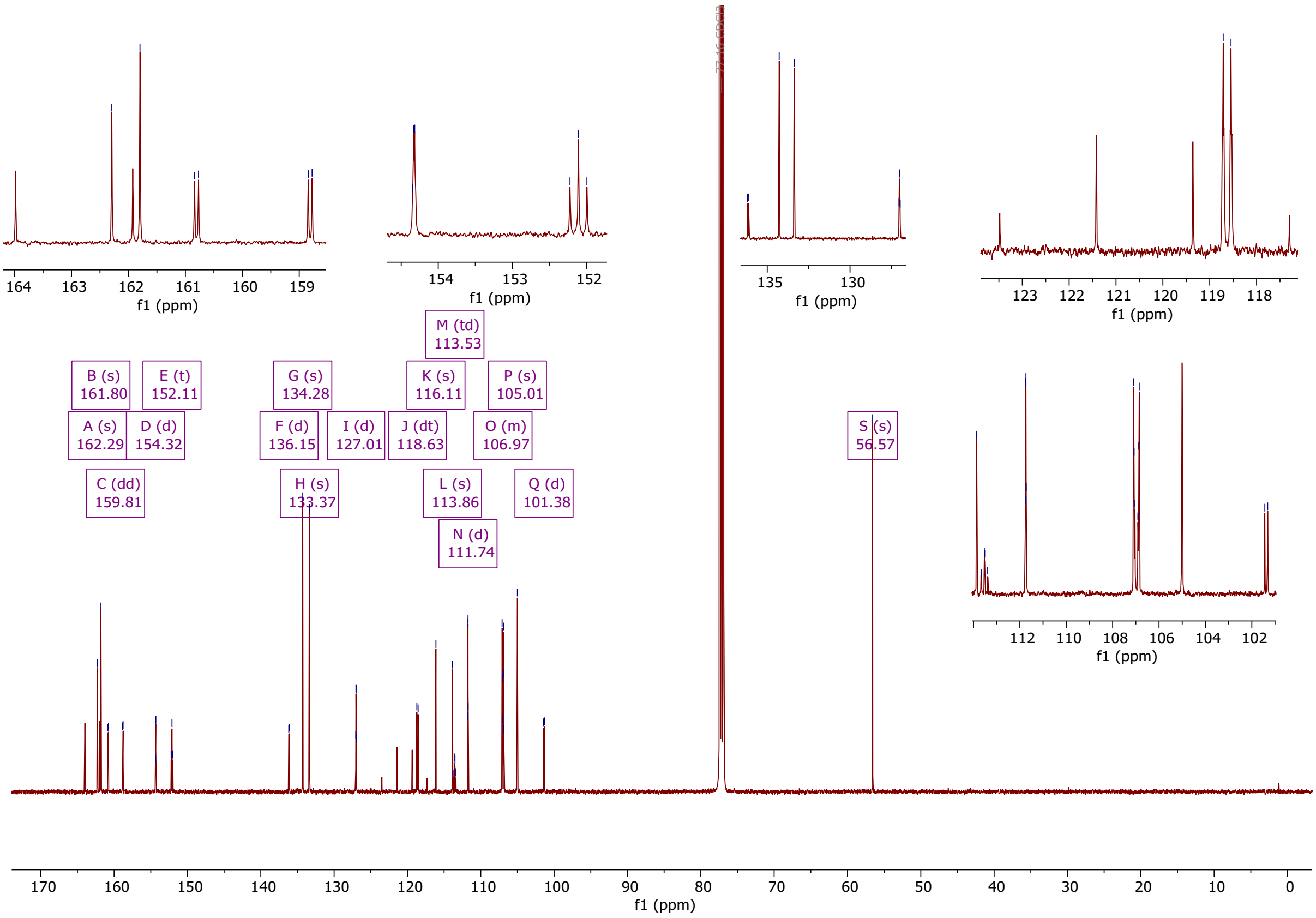

Figure S115 carbon NMR spectrum of **23**

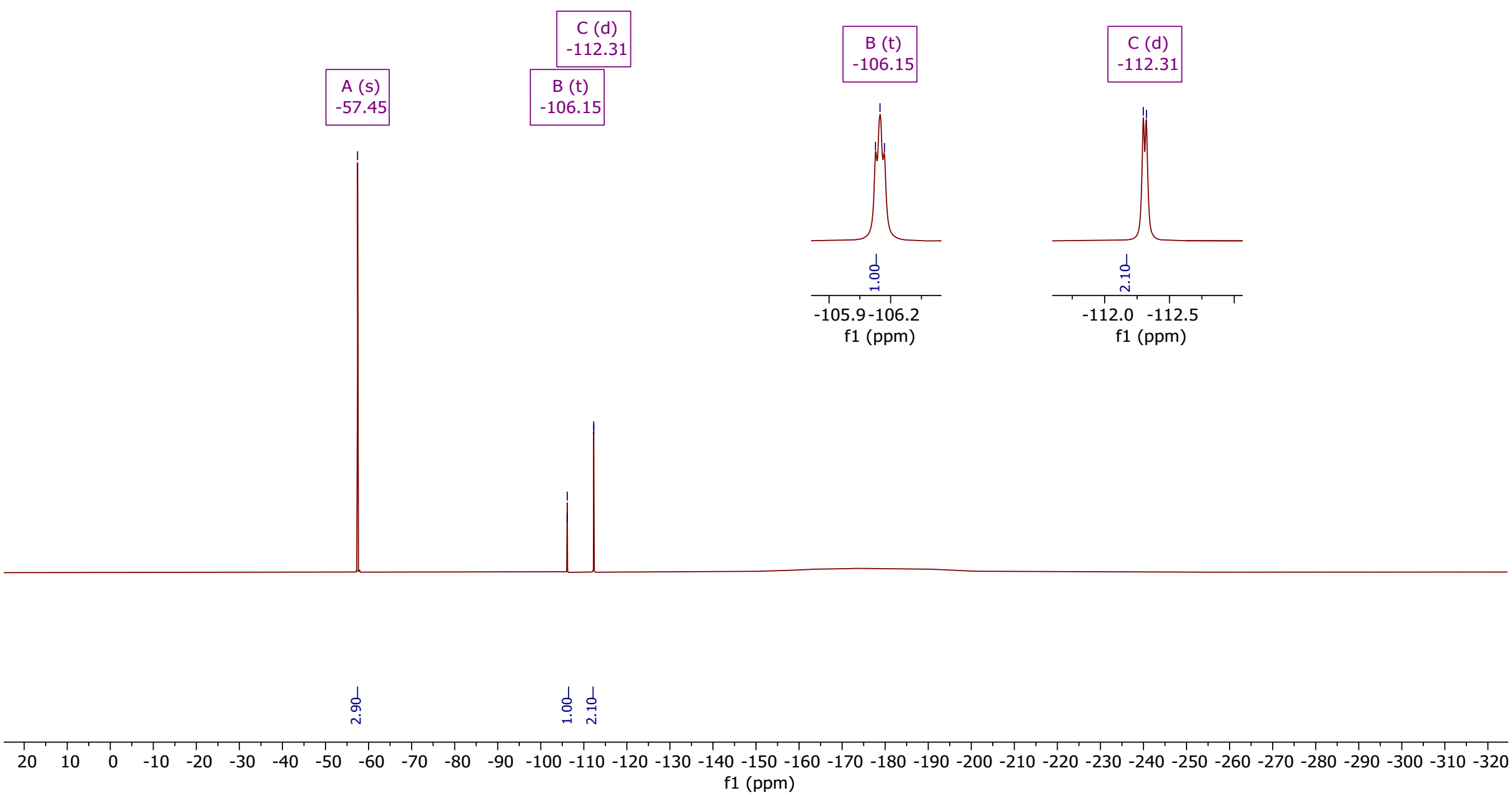

Figure S116 fluorine NMR spectrum of **23**

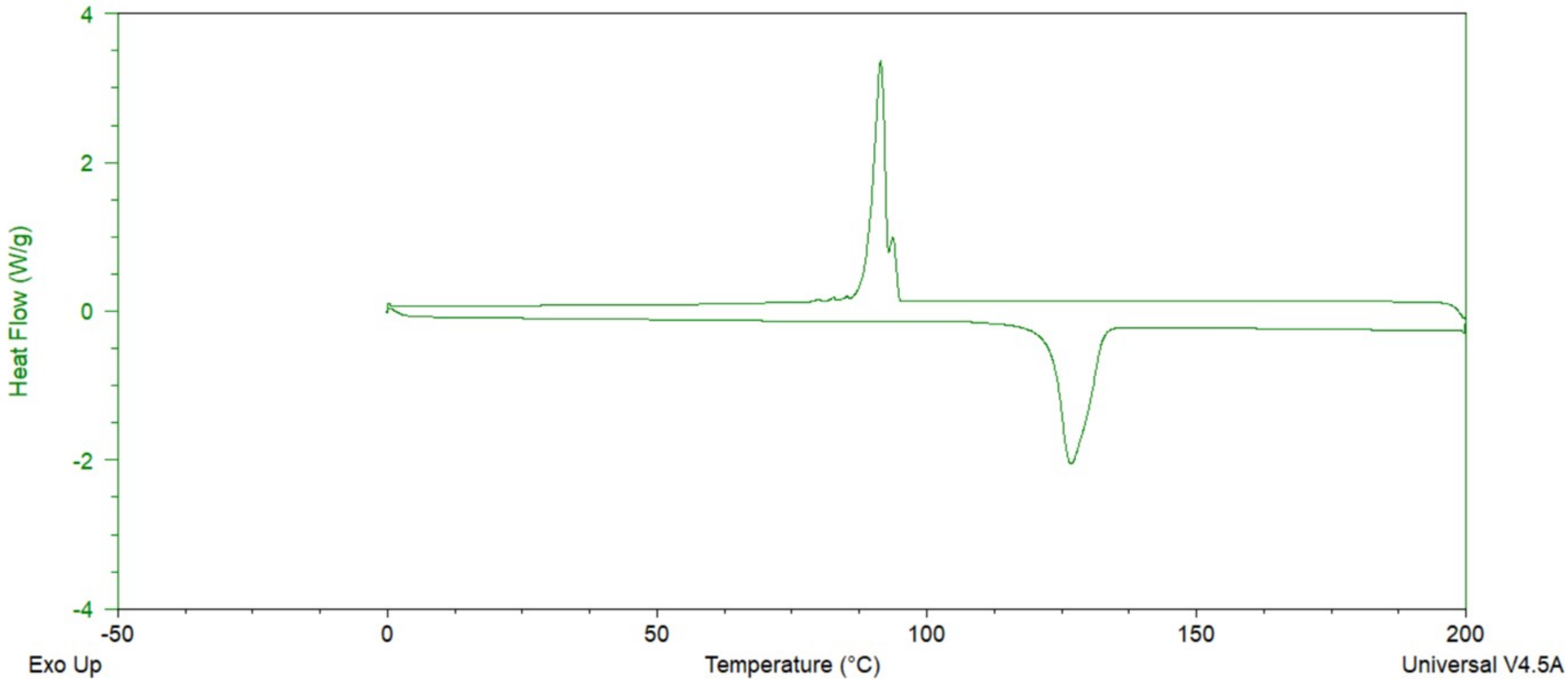

Figure S117 DSC thermogram of **23**.

24 | *4-bromo-3-methoxyphenyl 2',3,5-trifluoro-4'-methoxy-[1,1'-biphenyl]-4-carboxylate*

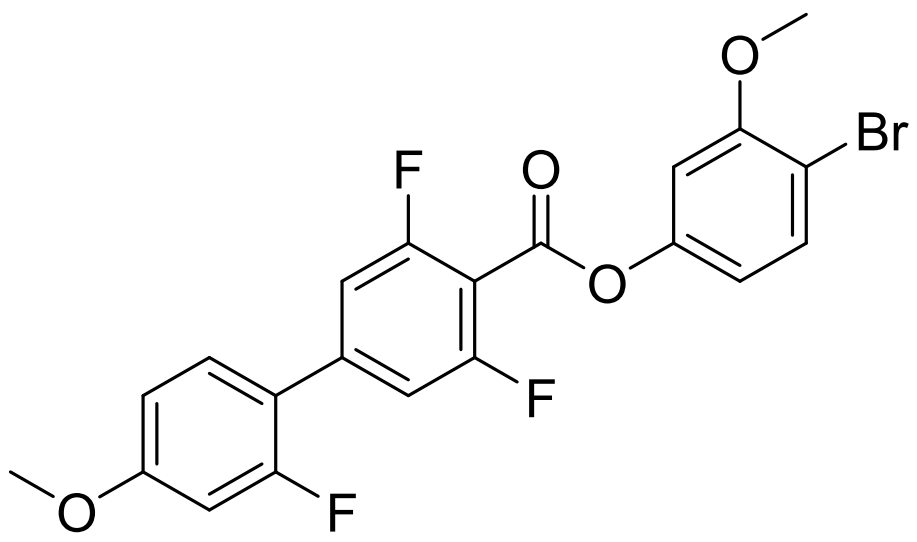

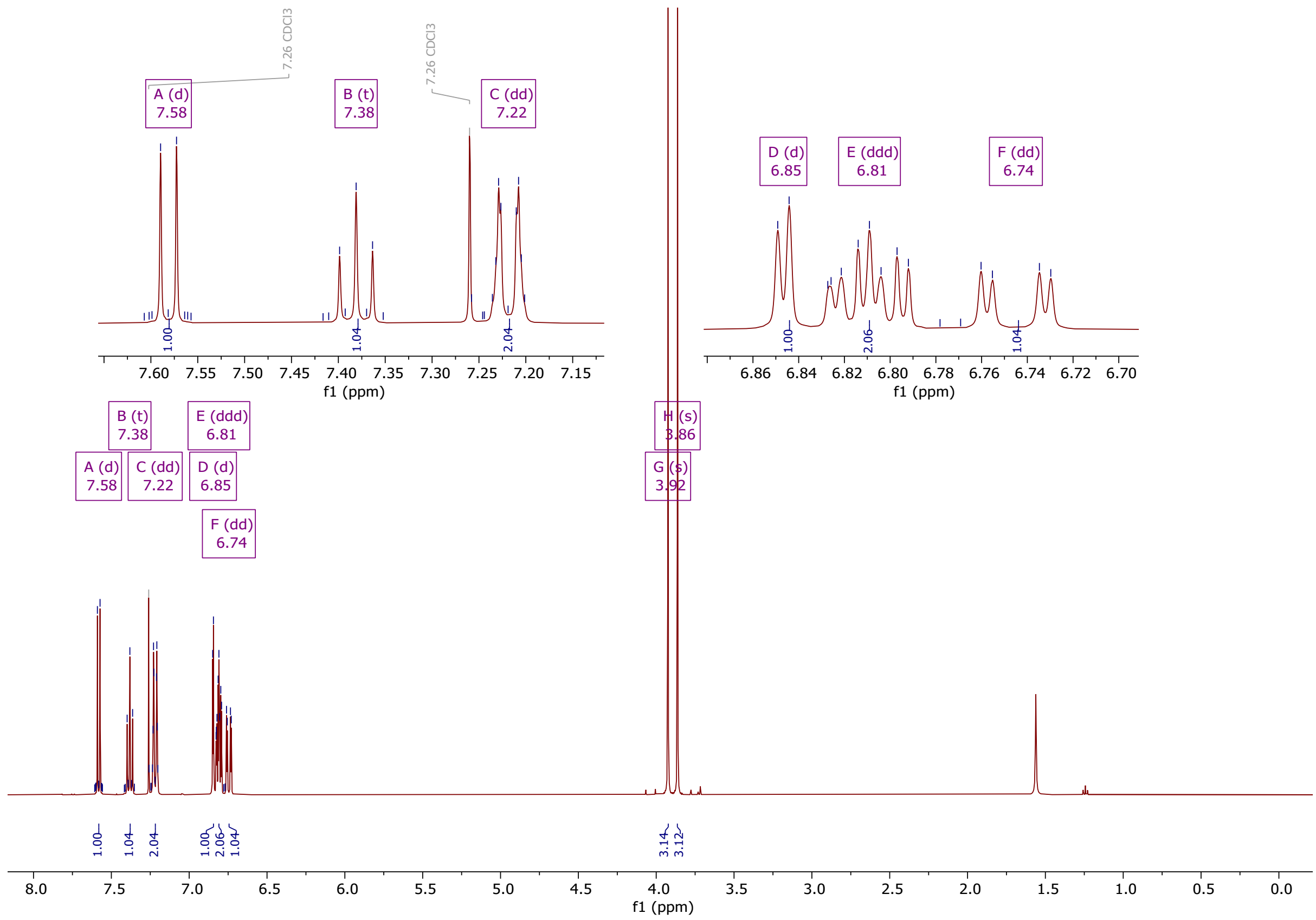

Figure S118 proton NMR spectrum of **24**

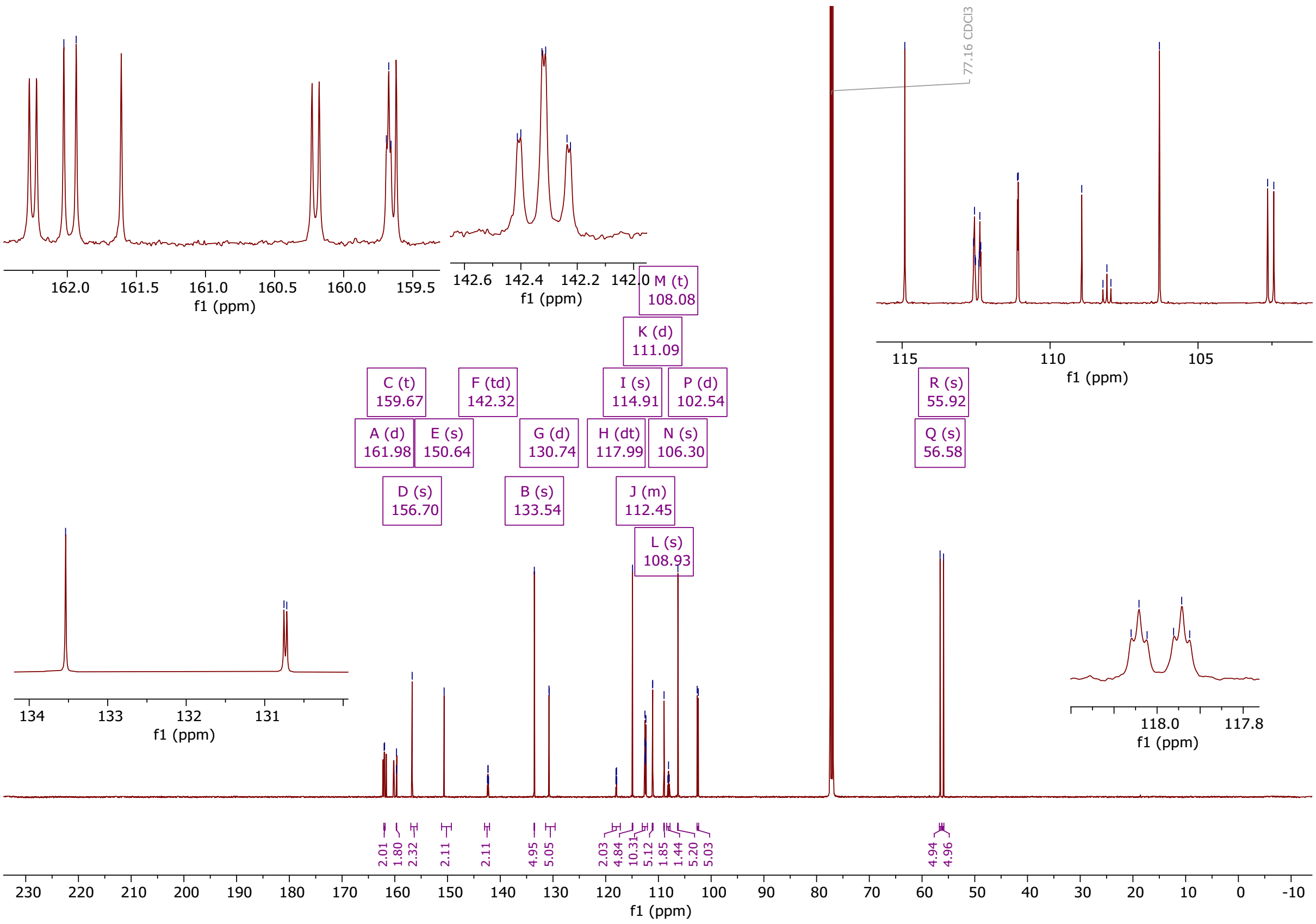

Figure S119 carbon NMR spectrum of **24**

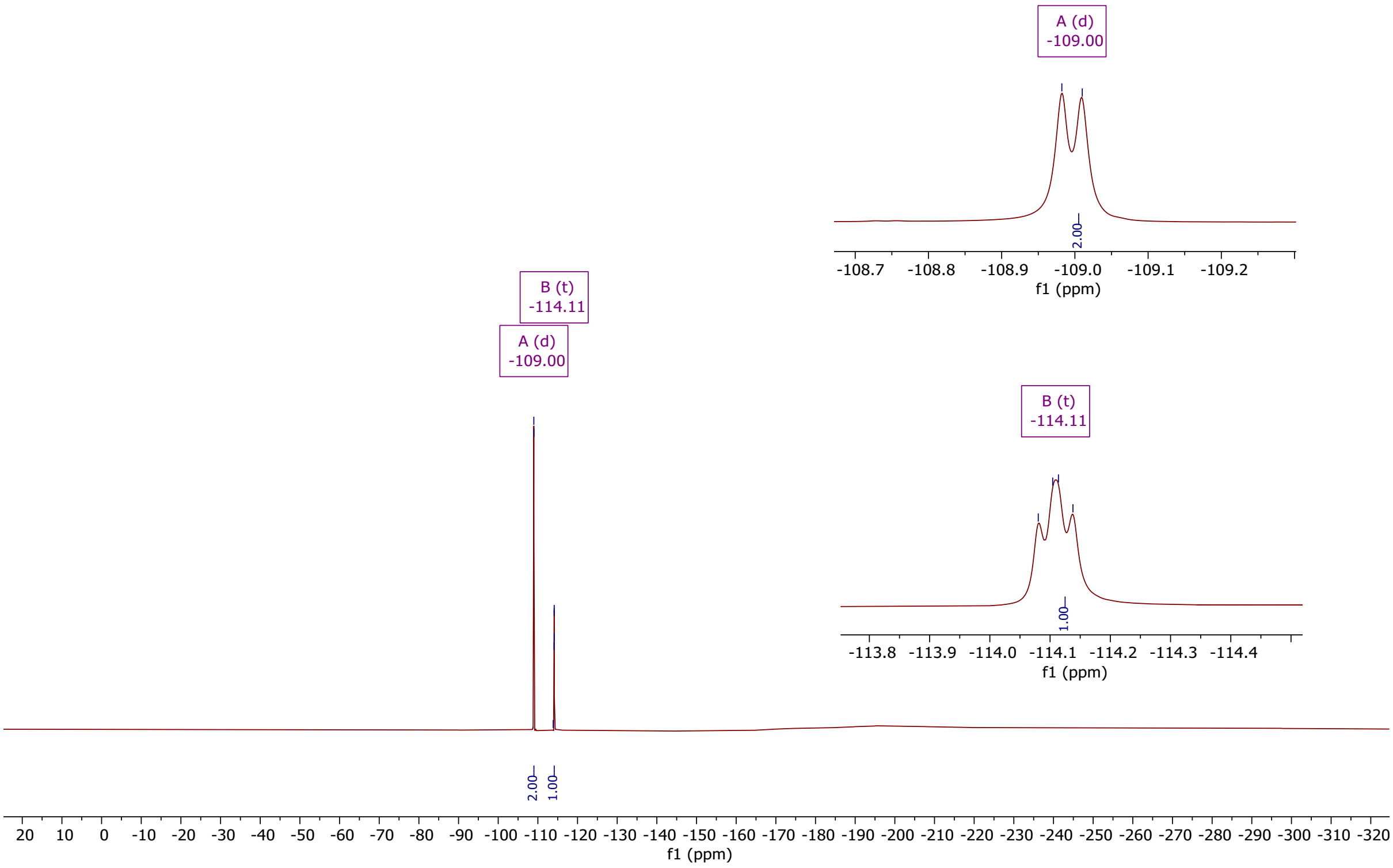

Figure S120 fluorine NMR spectrum of **24**.

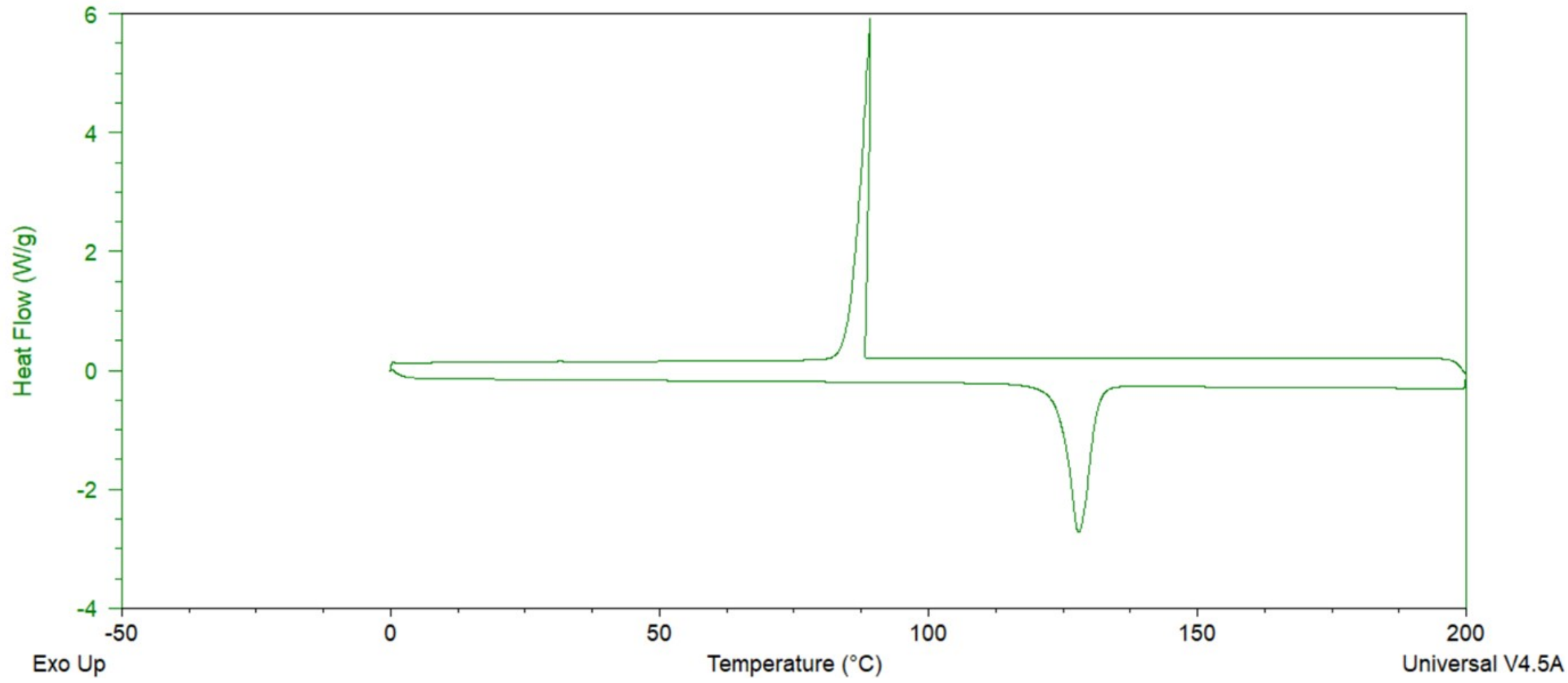

Figure S121 DSC thermogram of **24**.

25 | *4-isothiocyanatophenyl 4-bromo-2,6-difluorobenzoate*

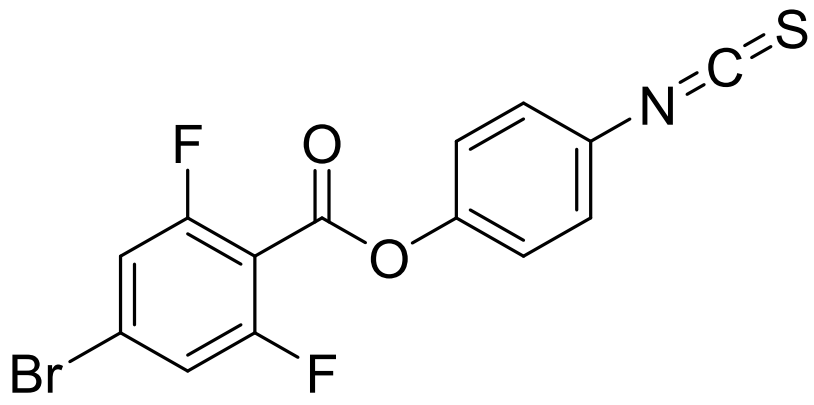

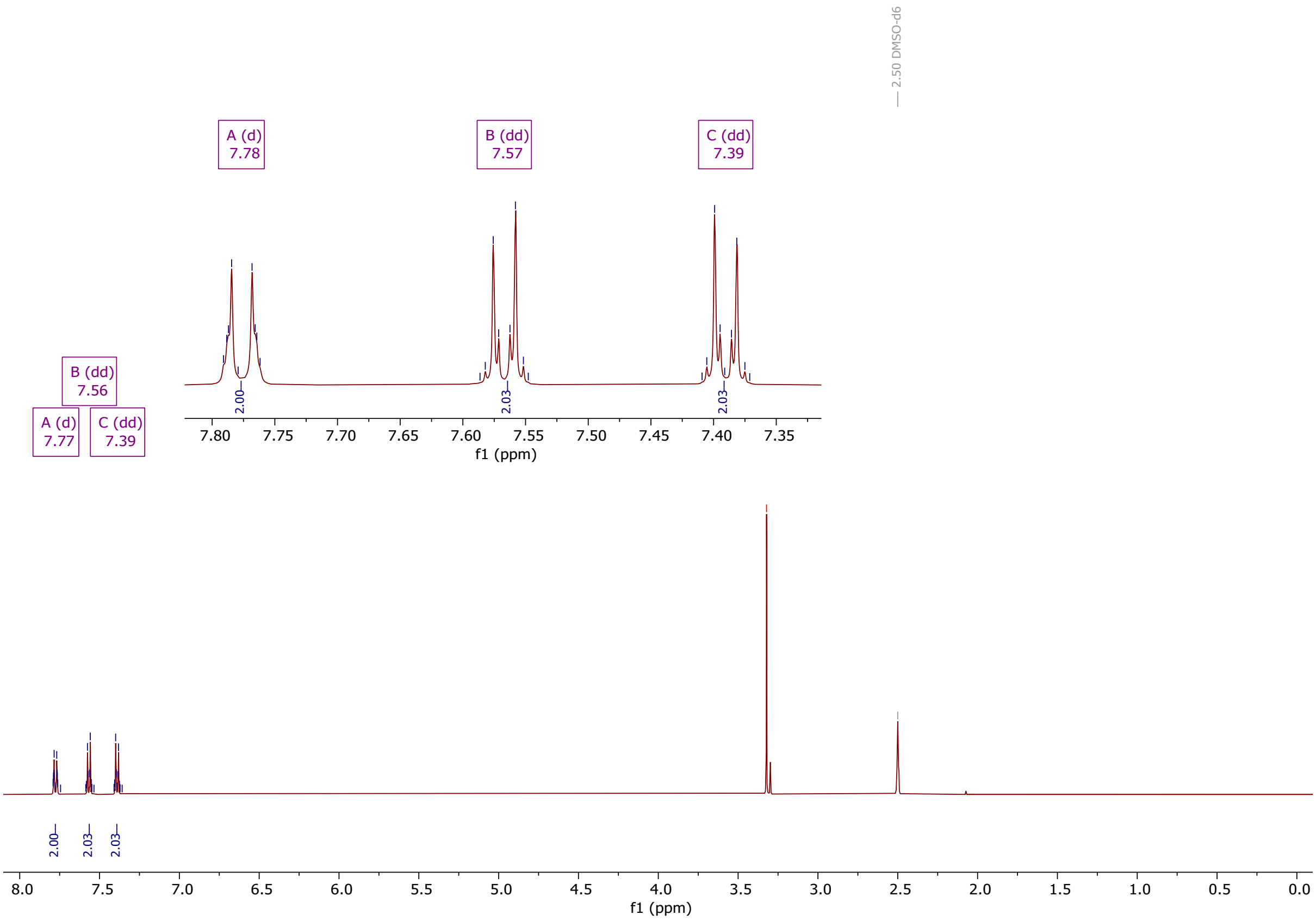

Figure S122 proton NMR spectrum of **25**

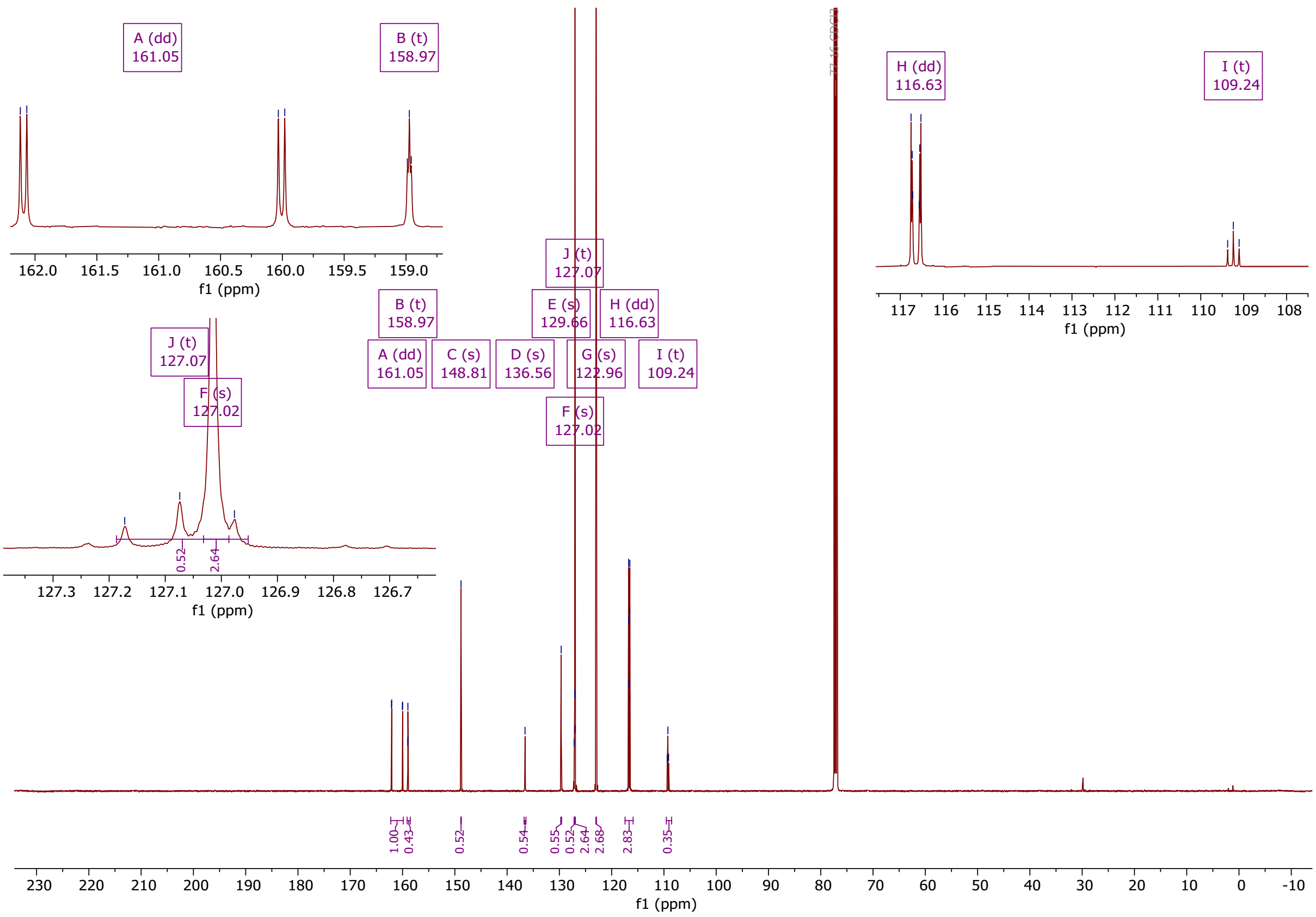

Figure S123 carbon NMR spectrum of **25**

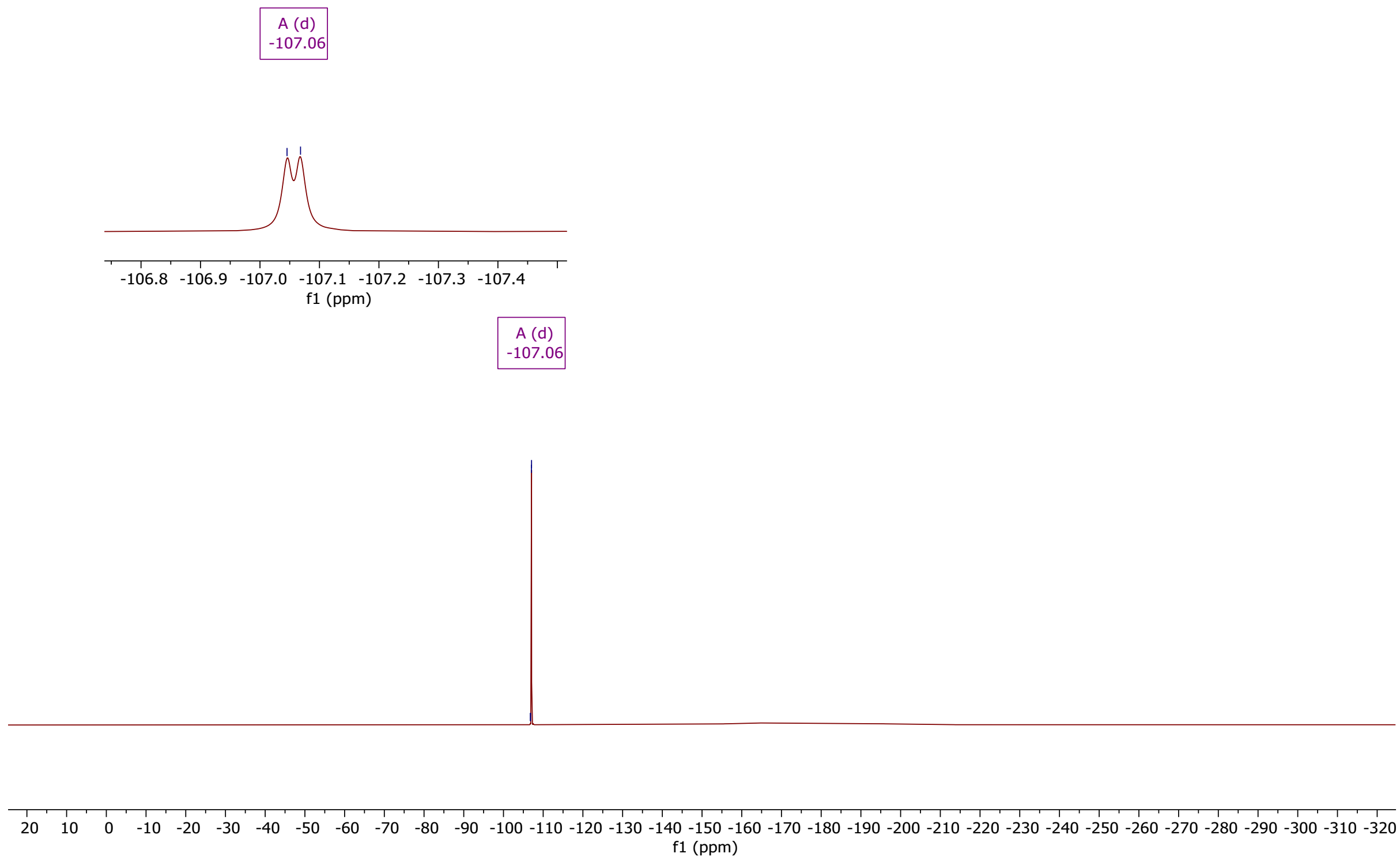

Figure S124 fluorine NMR spectrum of **25**

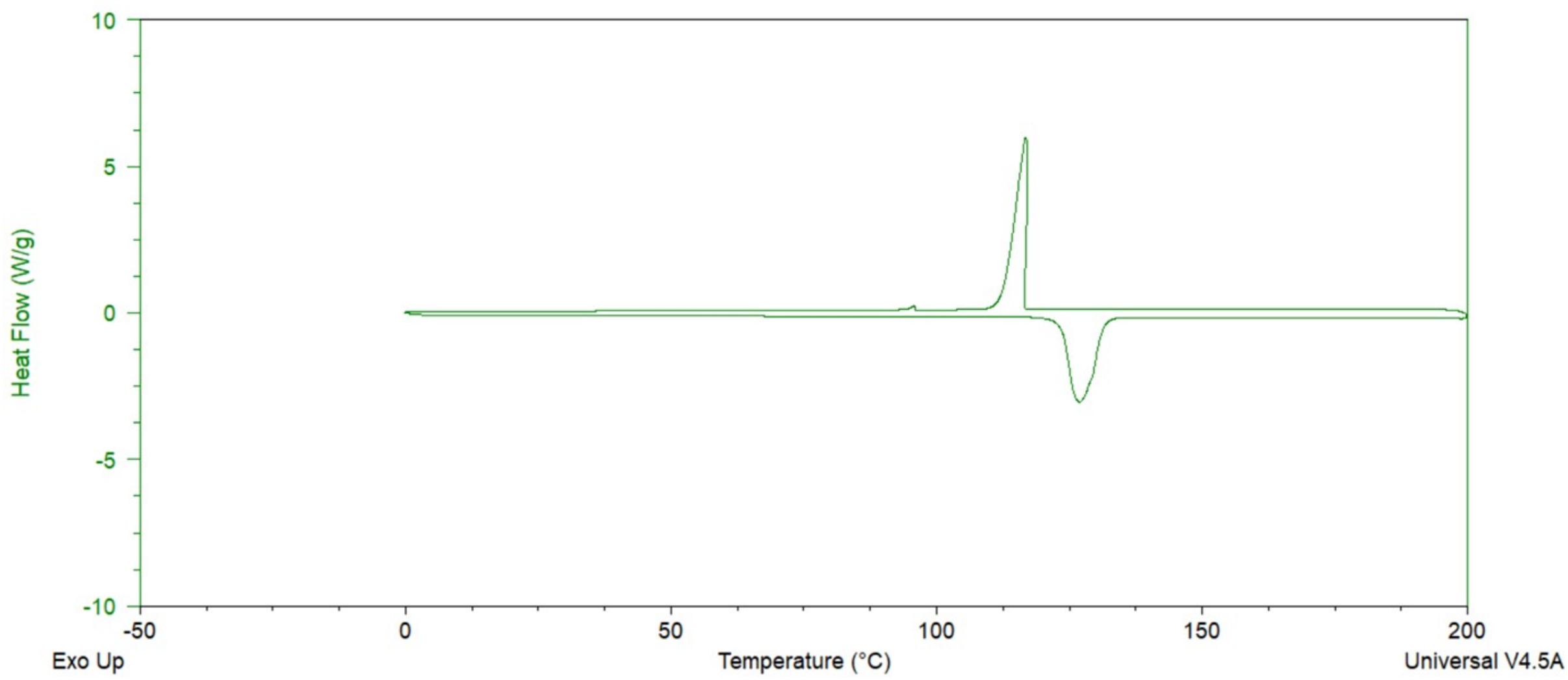

Figure S125 DSC thermogram of **25**.

26 | *4-((1r,4s)-4-propylcyclohexyl)phenyl 4-(benzyloxy)benzoate*

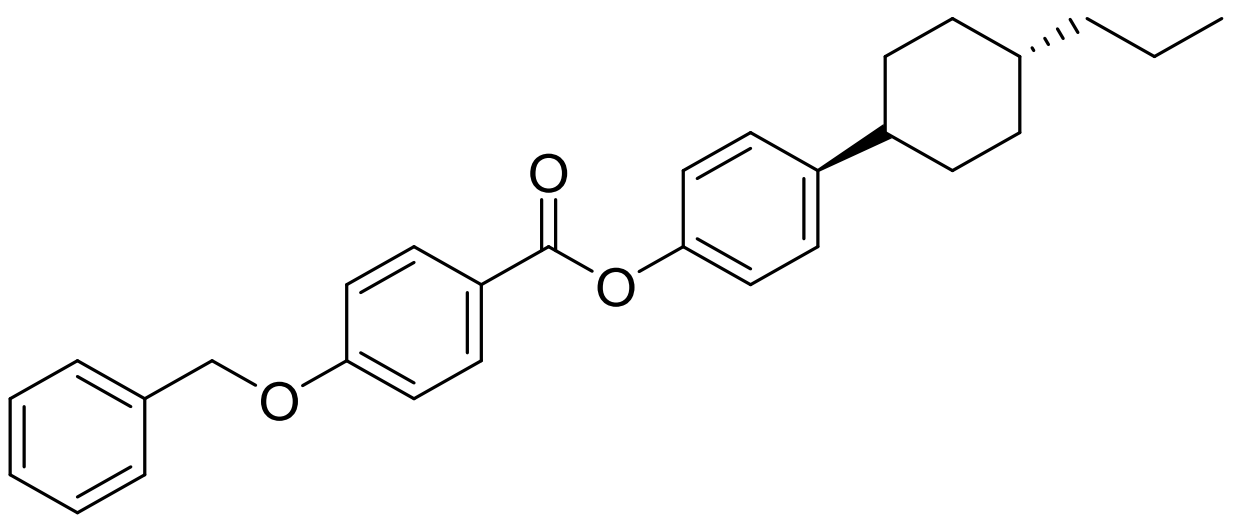

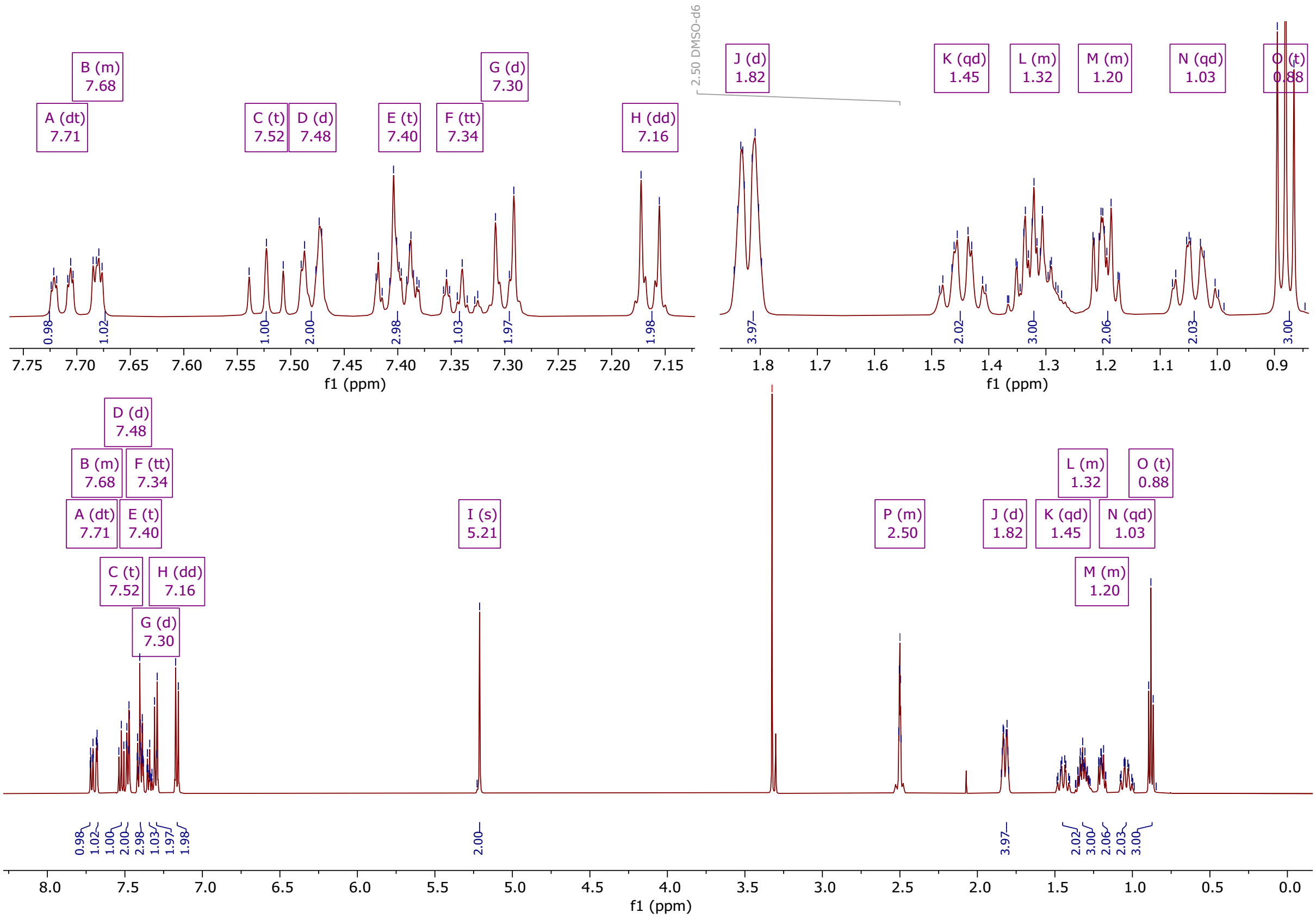

Figure S126 proton NMR spectrum of **26**

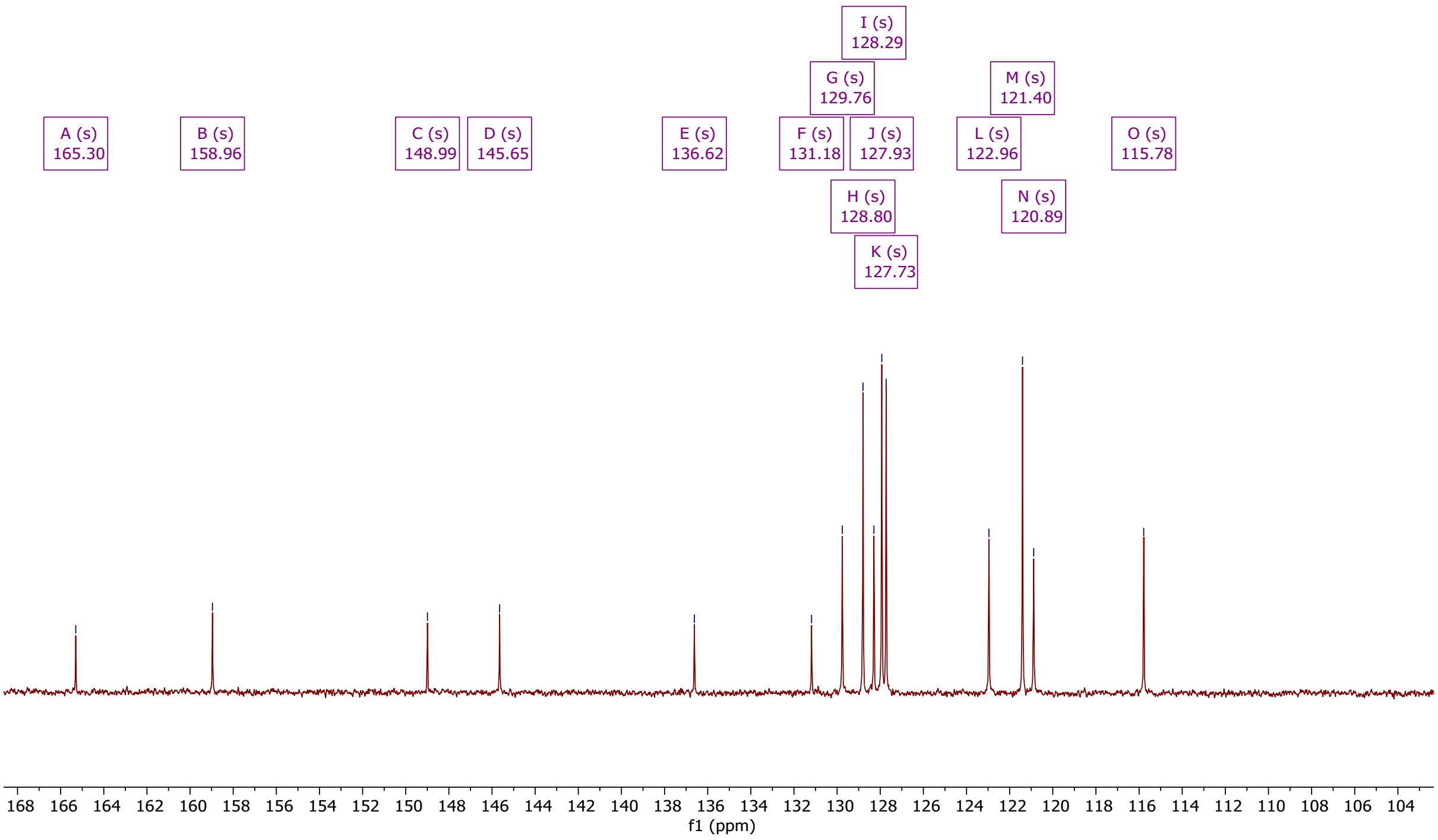

Figure S127 carbon NMR spectrum of **26**

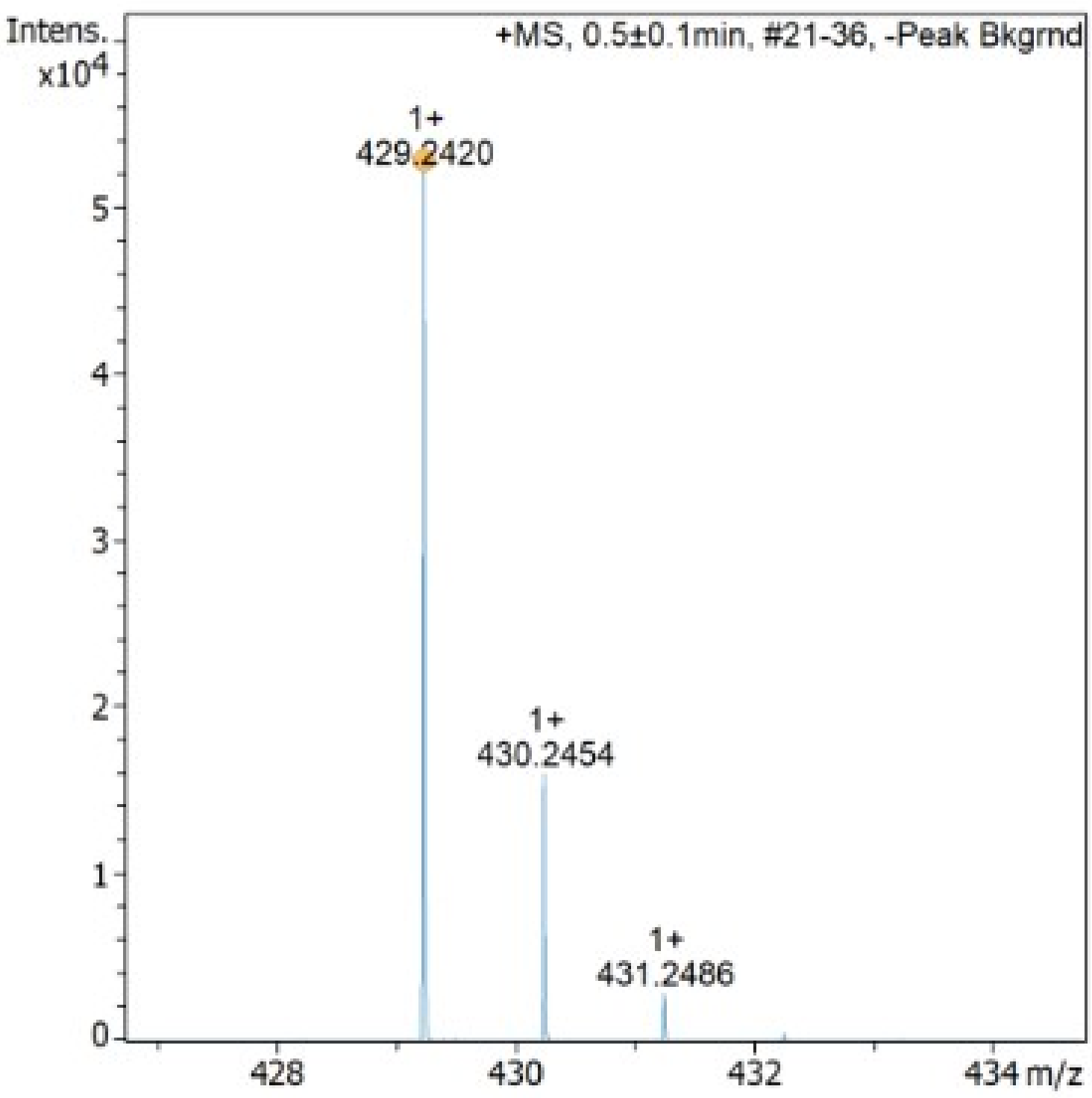

Figure S128 HRMS of **26**.

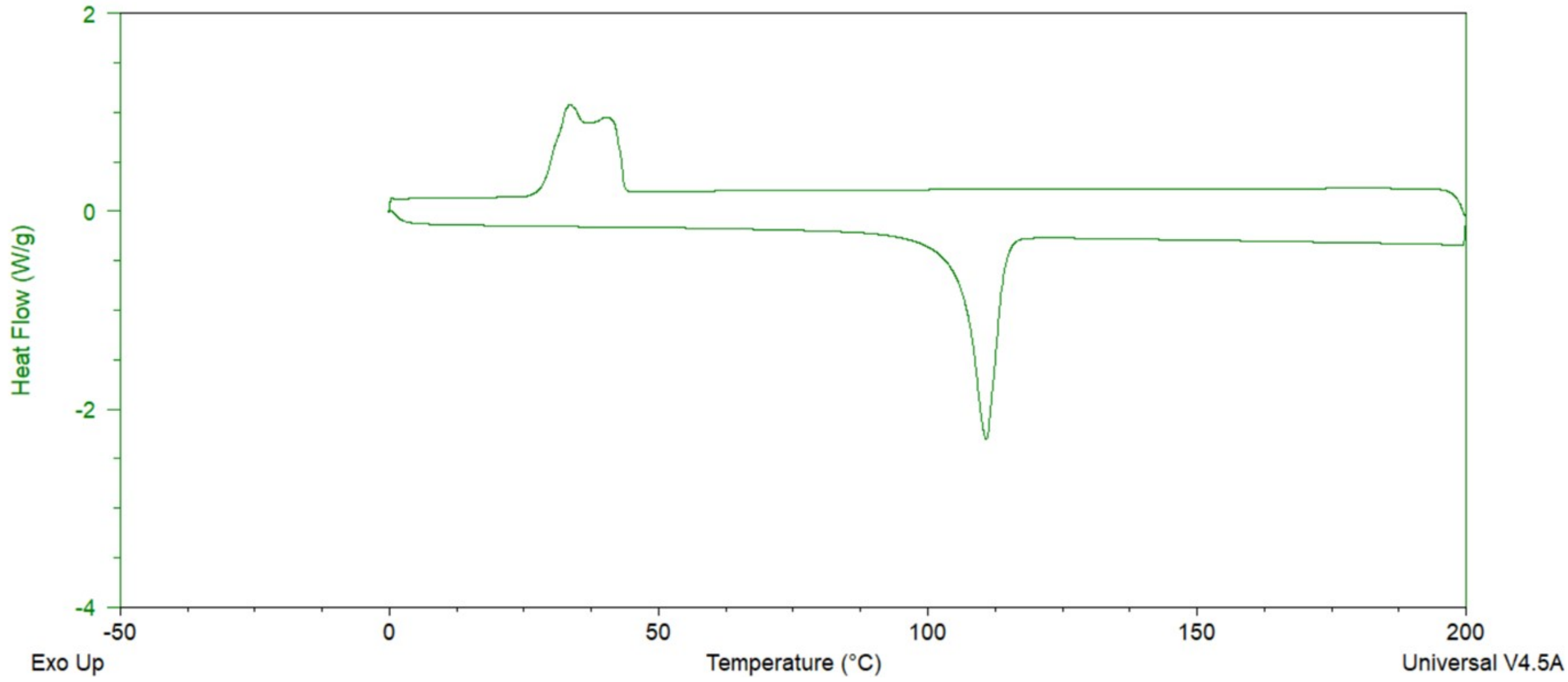

Figure S129 DSC thermogram of **26**.

27 | *bis(2,3',4',5'-tetrafluoro-[1,1'-biphenyl]-4-yl) nonanedioate*

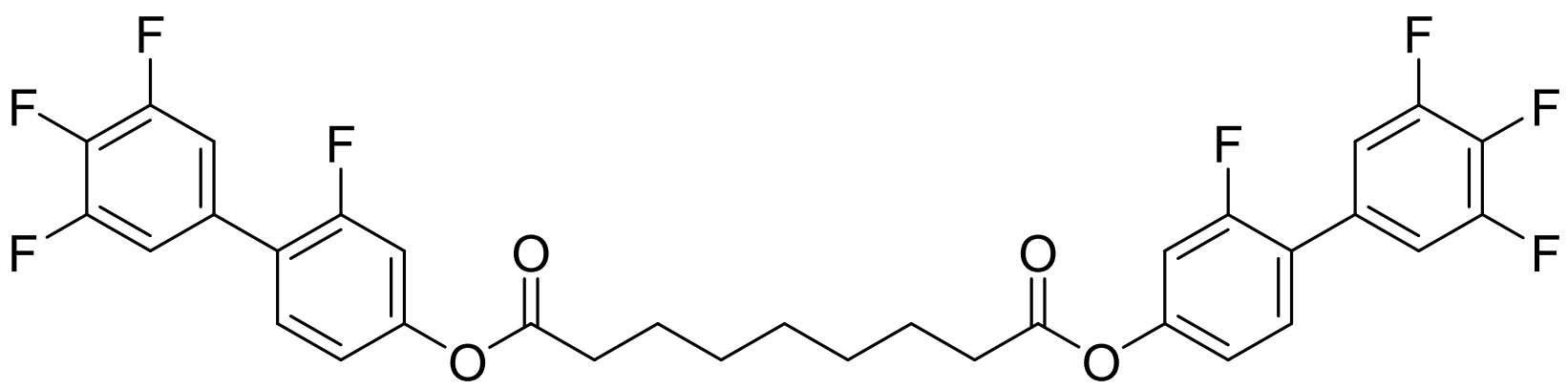

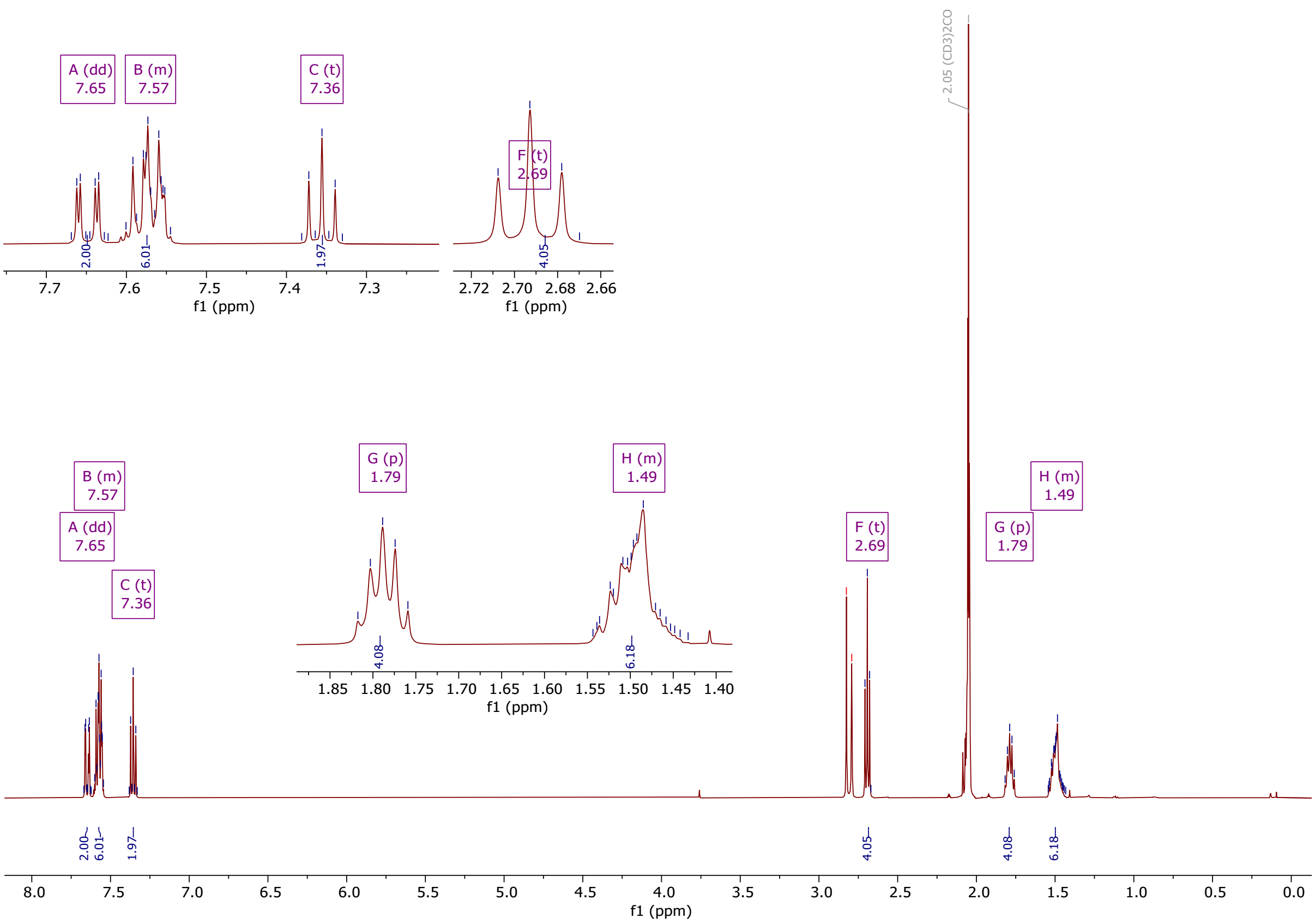

Figure S130 proton NMR spectrum of **27**

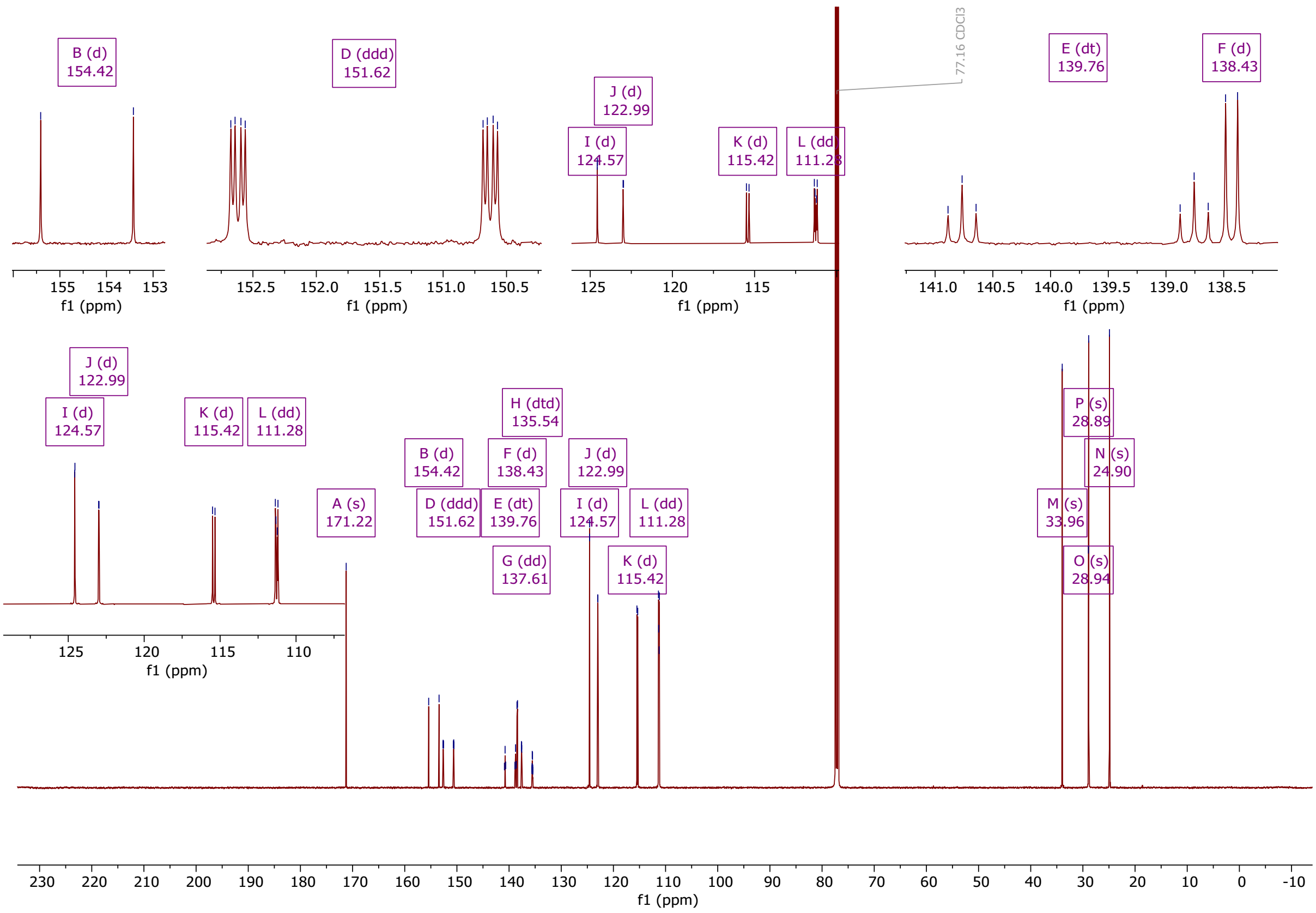

Figure S131 carbon NMR spectrum of **27**

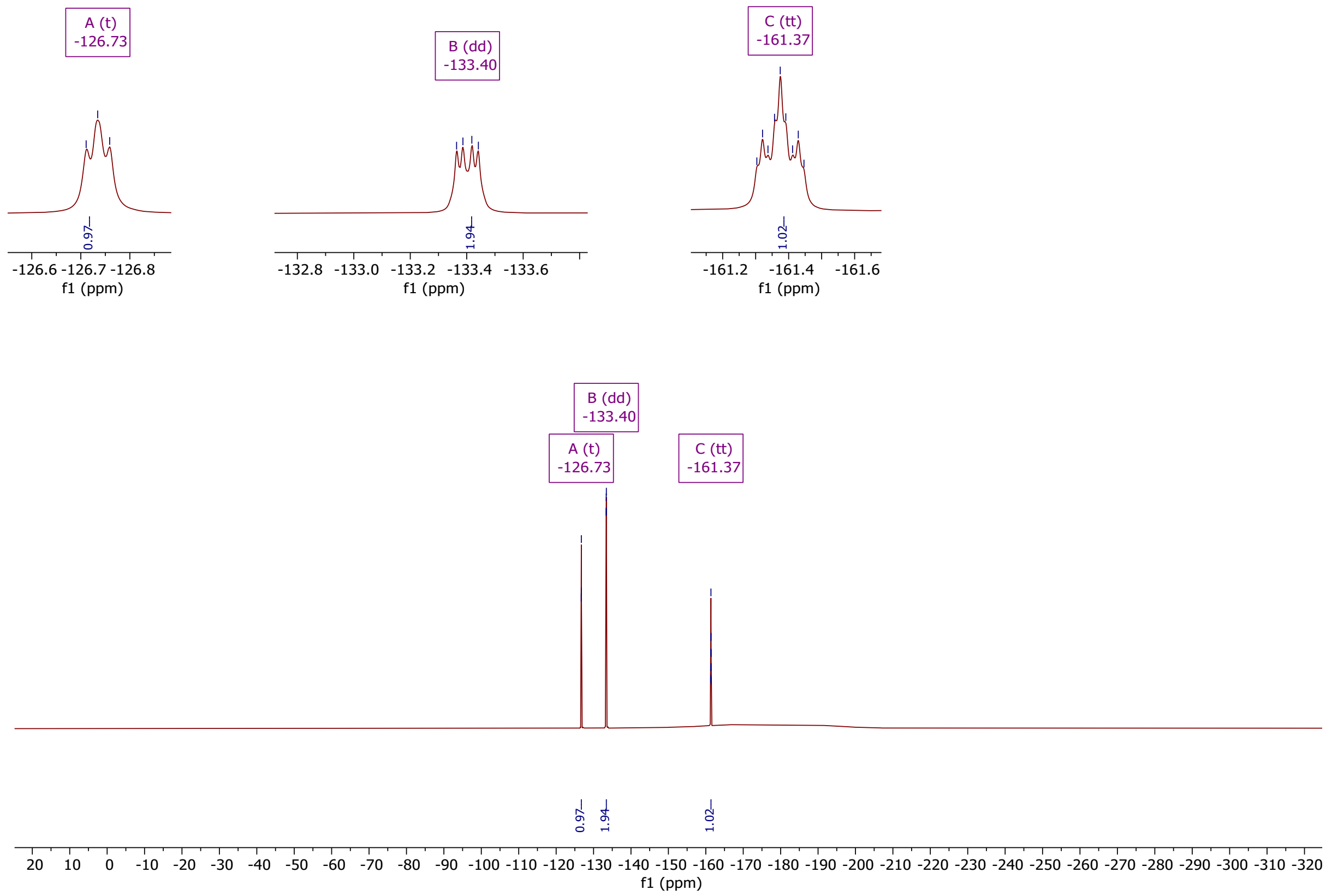

Figure S132 fluorine NMR spectrum of **27**

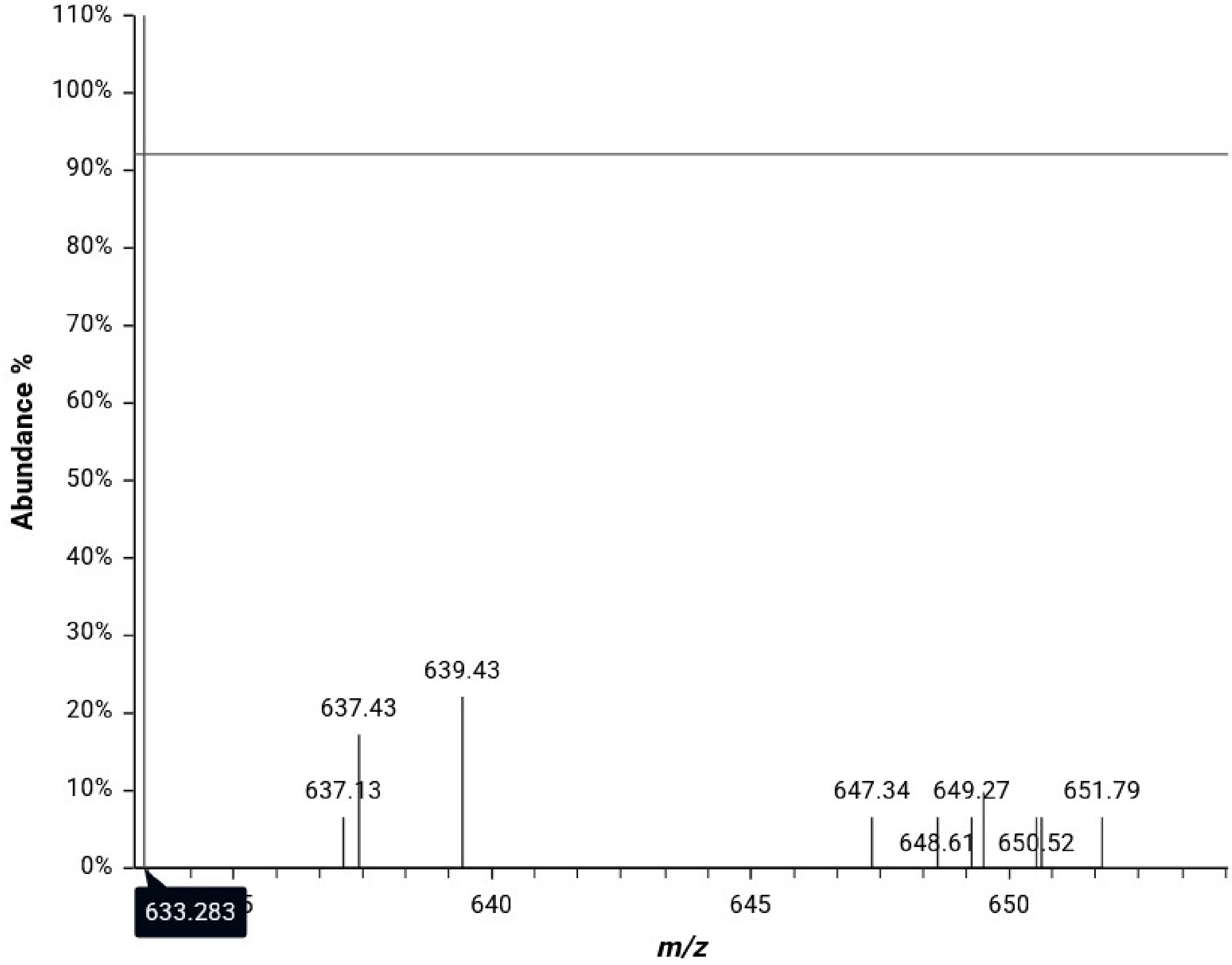

Figure S133 HRMS of **27**.

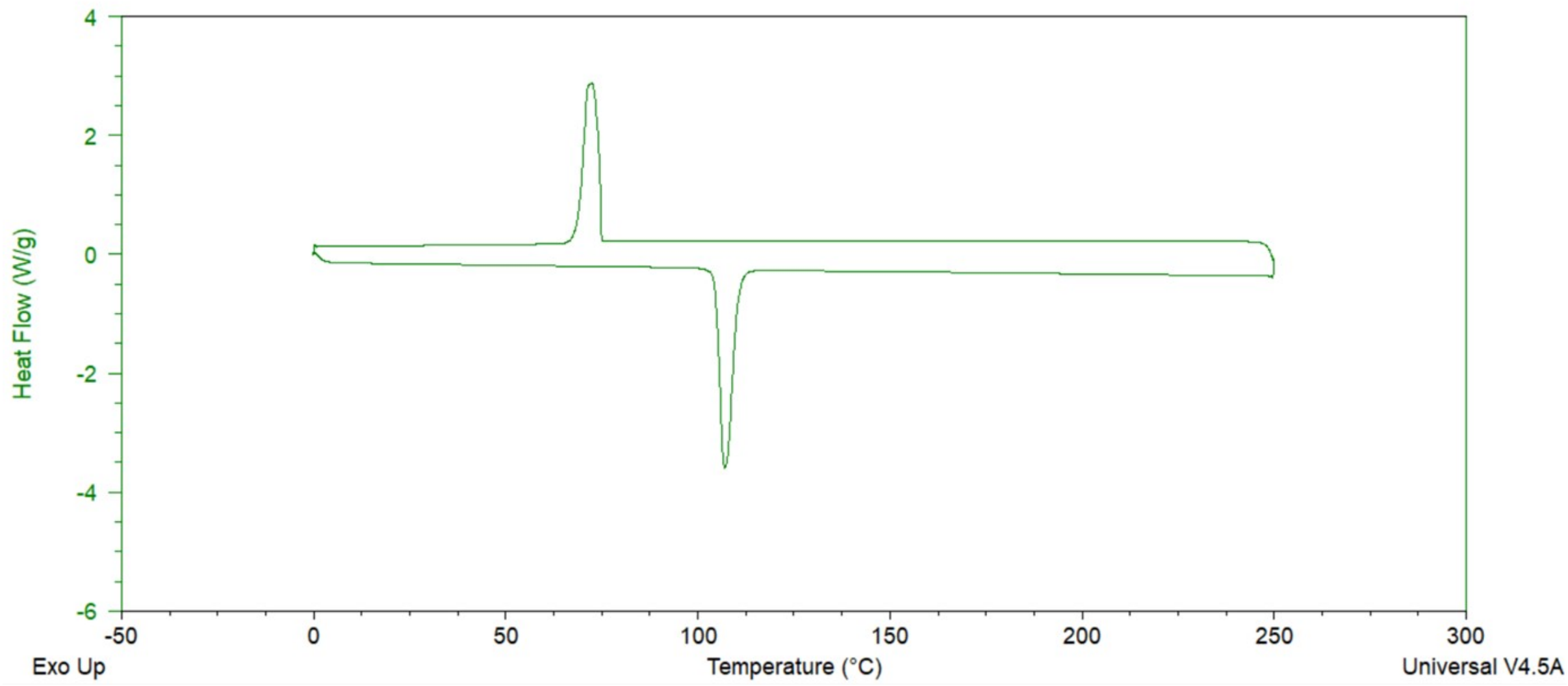

Figure S134 DSC thermogram of **27**.

28 | *bis(4-((1s,4r)-4-propylcyclohexyl)phenyl) nonanedioate*

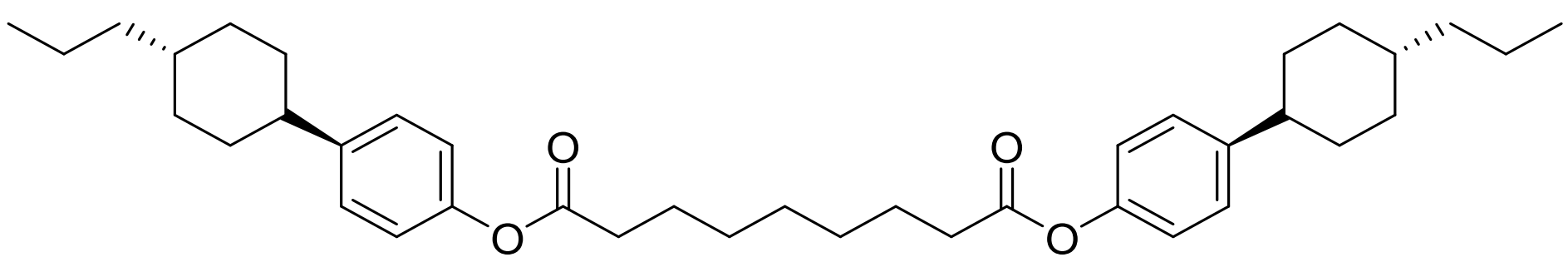

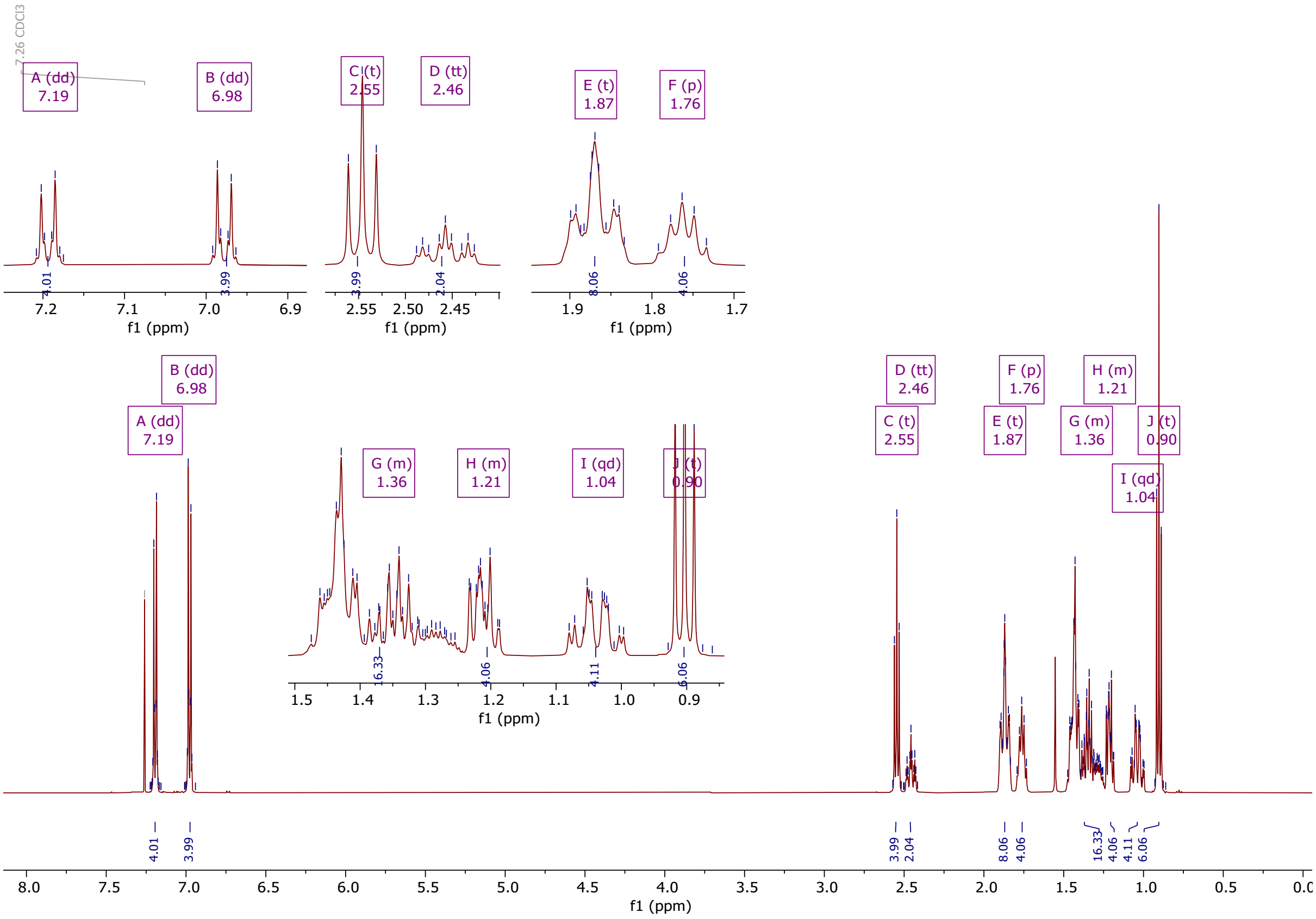

Figure S135 proton NMR spectrum of **28**

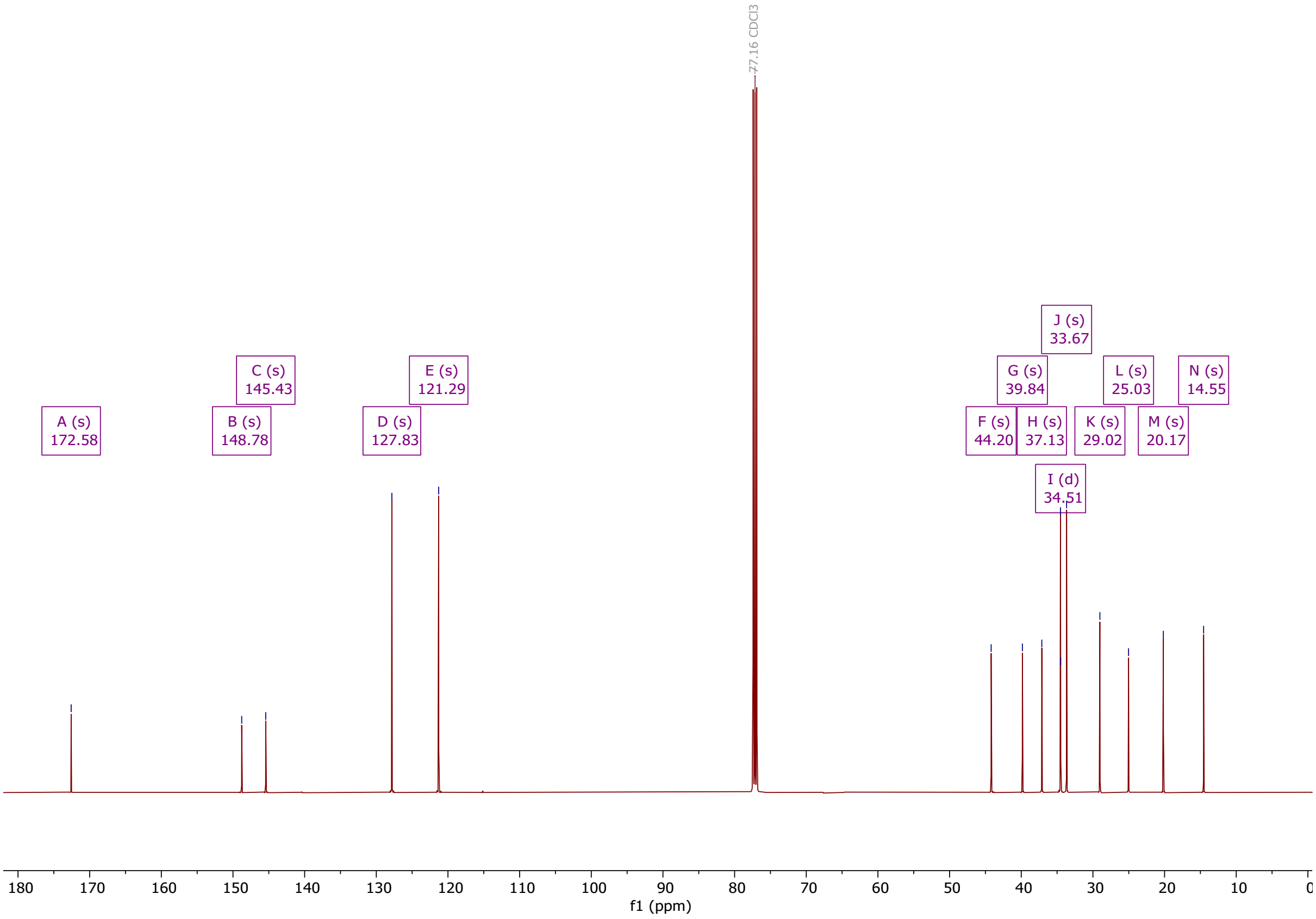

Figure S136 carbon NMR spectrum of **28**

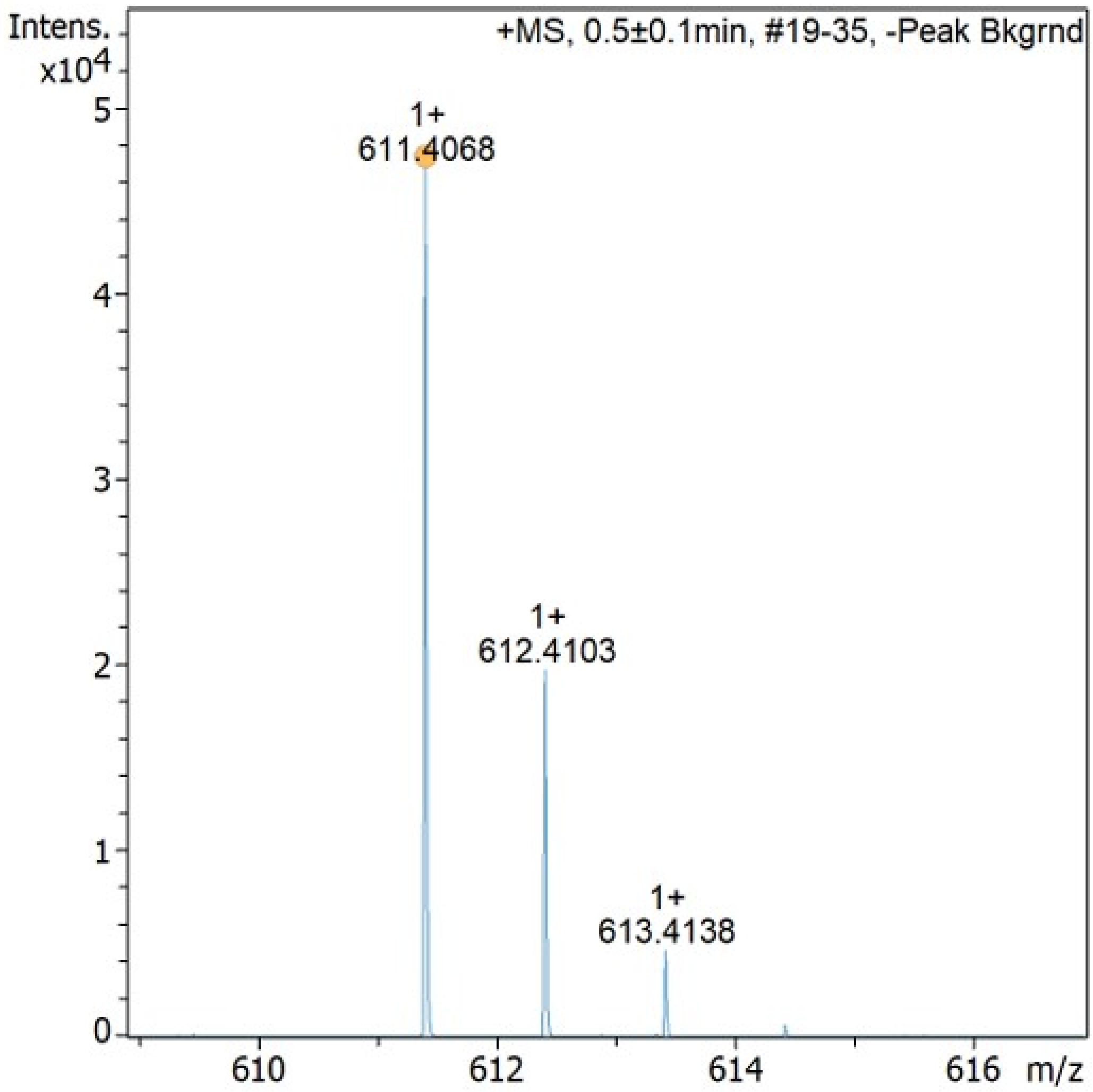

Figure S137 HRMS of **28**.

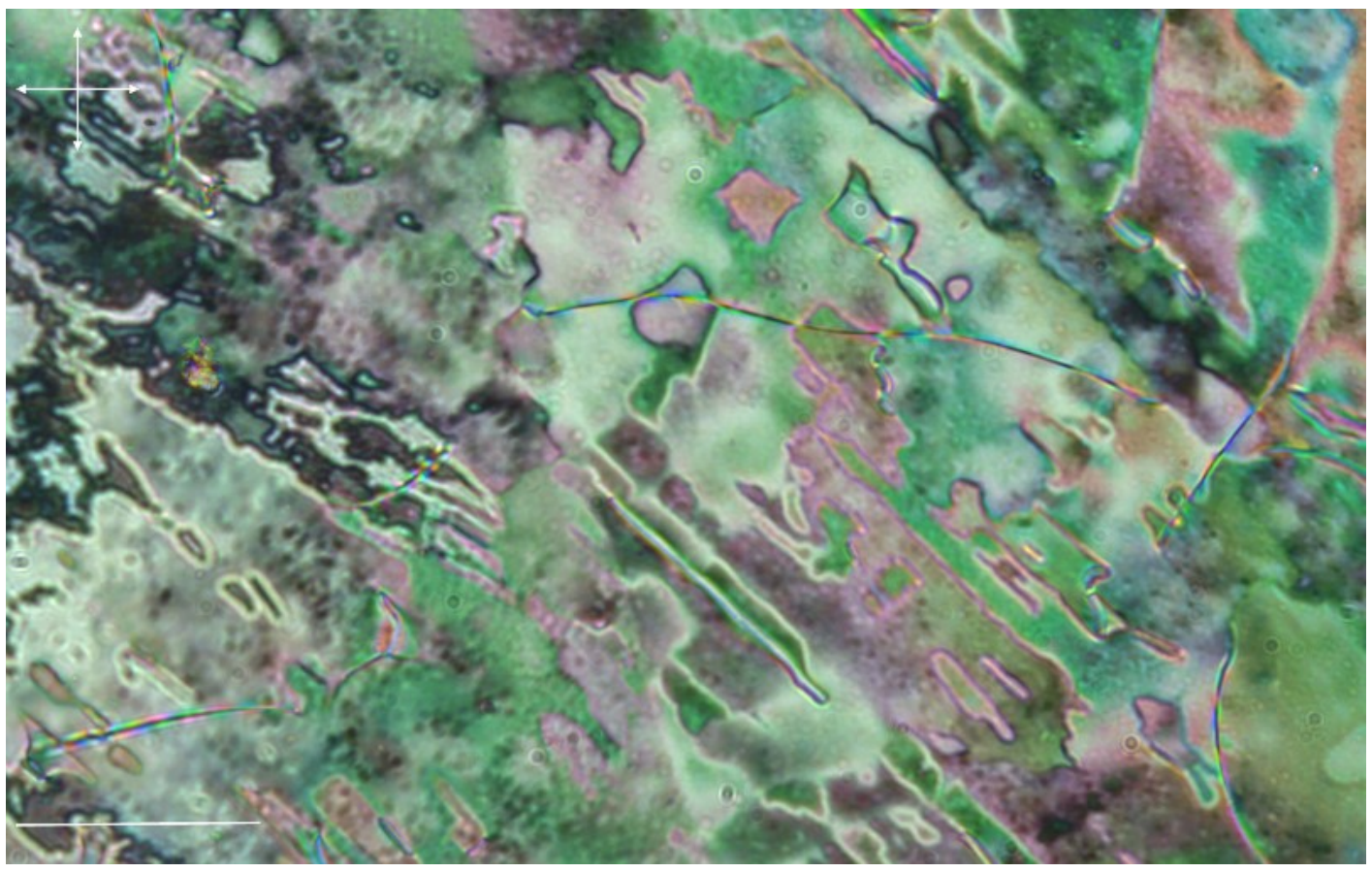

Figure S138 POM image of **28**, N at 108 °C. Scale bar (bottom-left) shows 1 μm, arrows show polariser direction.

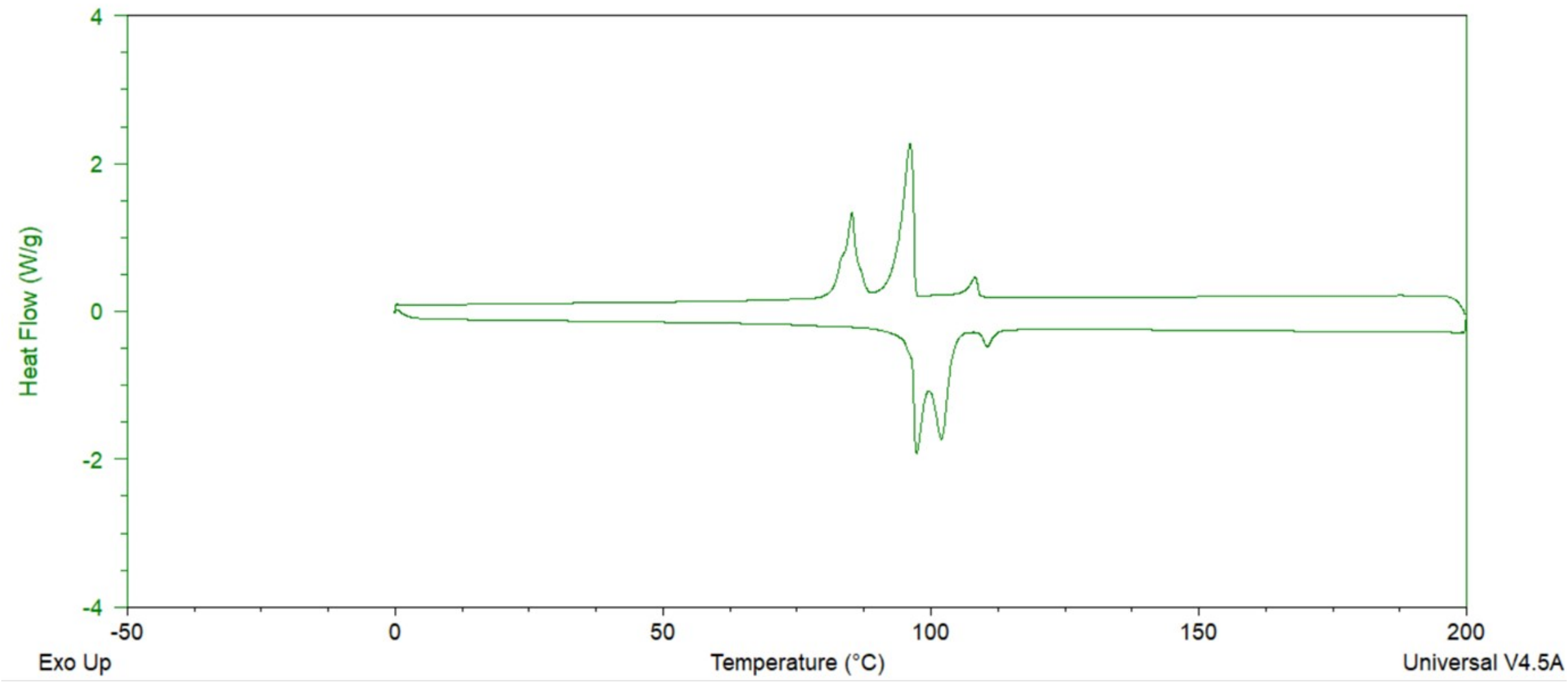

Figure S139 DSC thermogram of **28**.

CA1 | *2,6-difluoro-4-(4-pentylphenyl)benzoic acid*

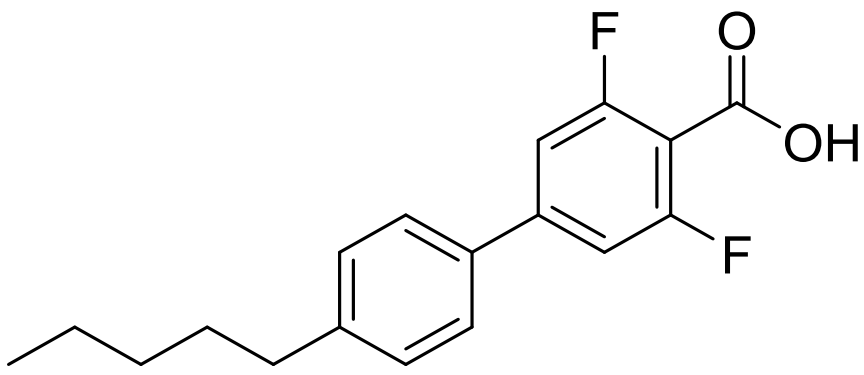

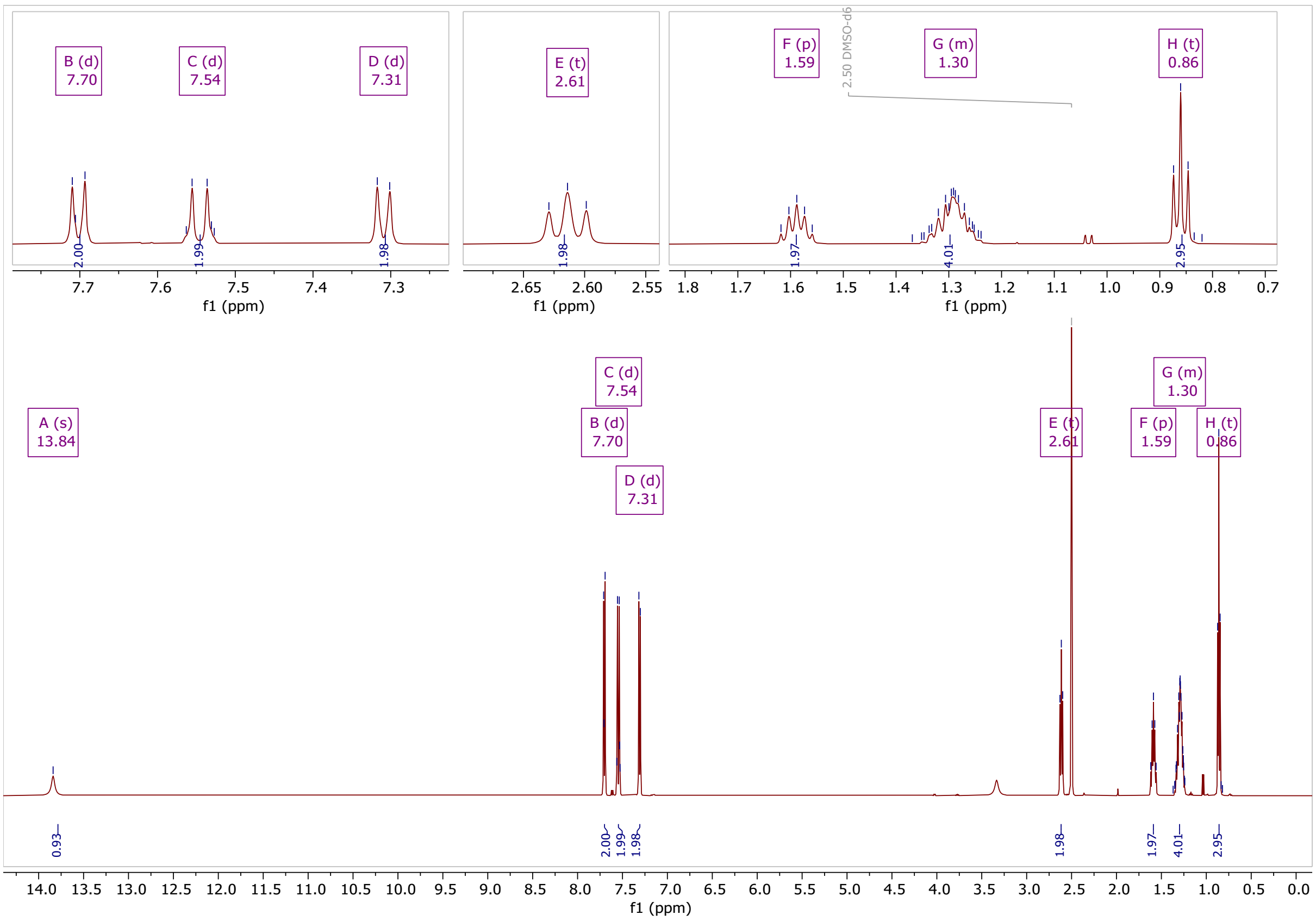

Figure S140 proton NMR spectrum of **CA1**

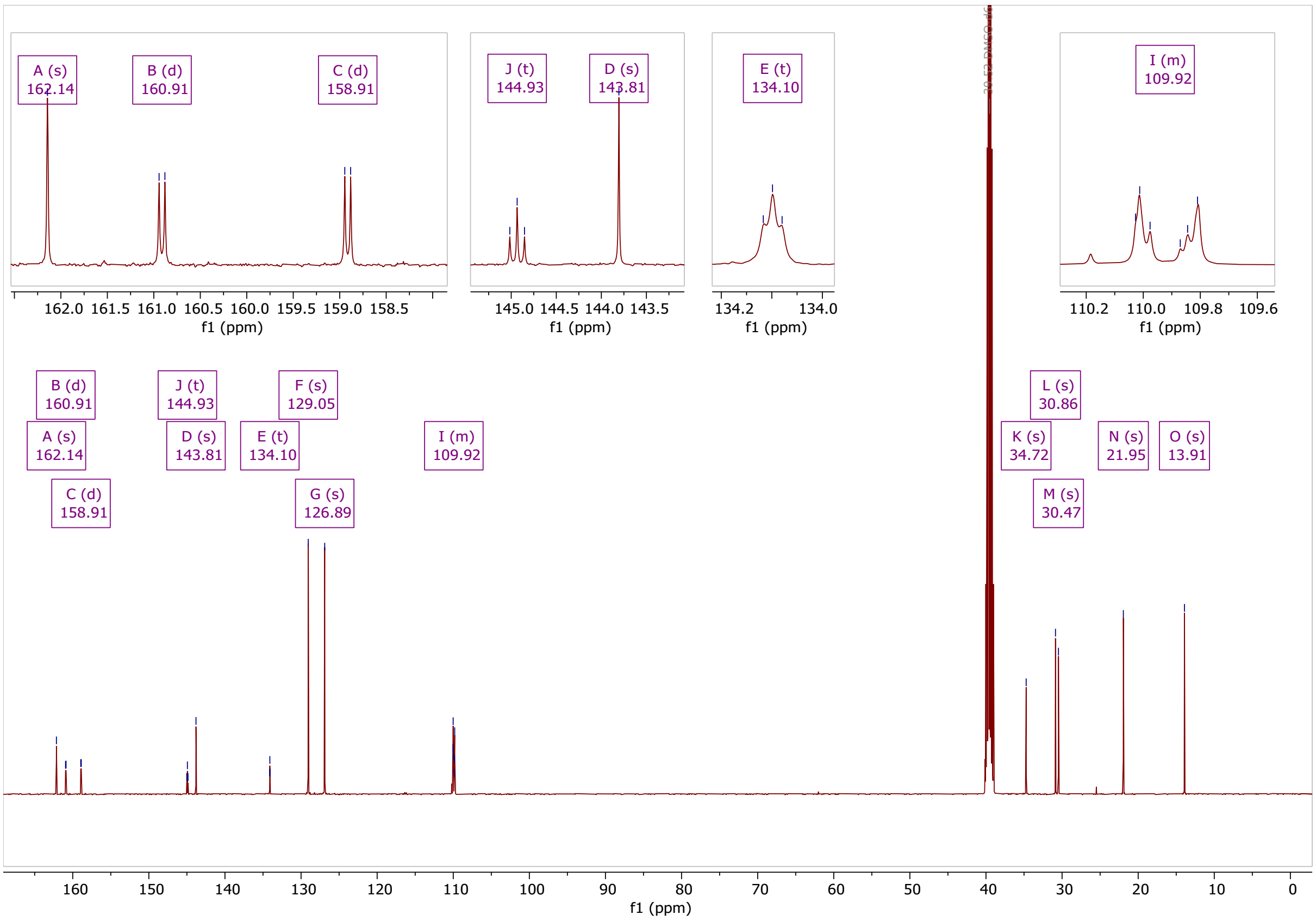

Figure S141 carbon NMR spectrum of **CA1**

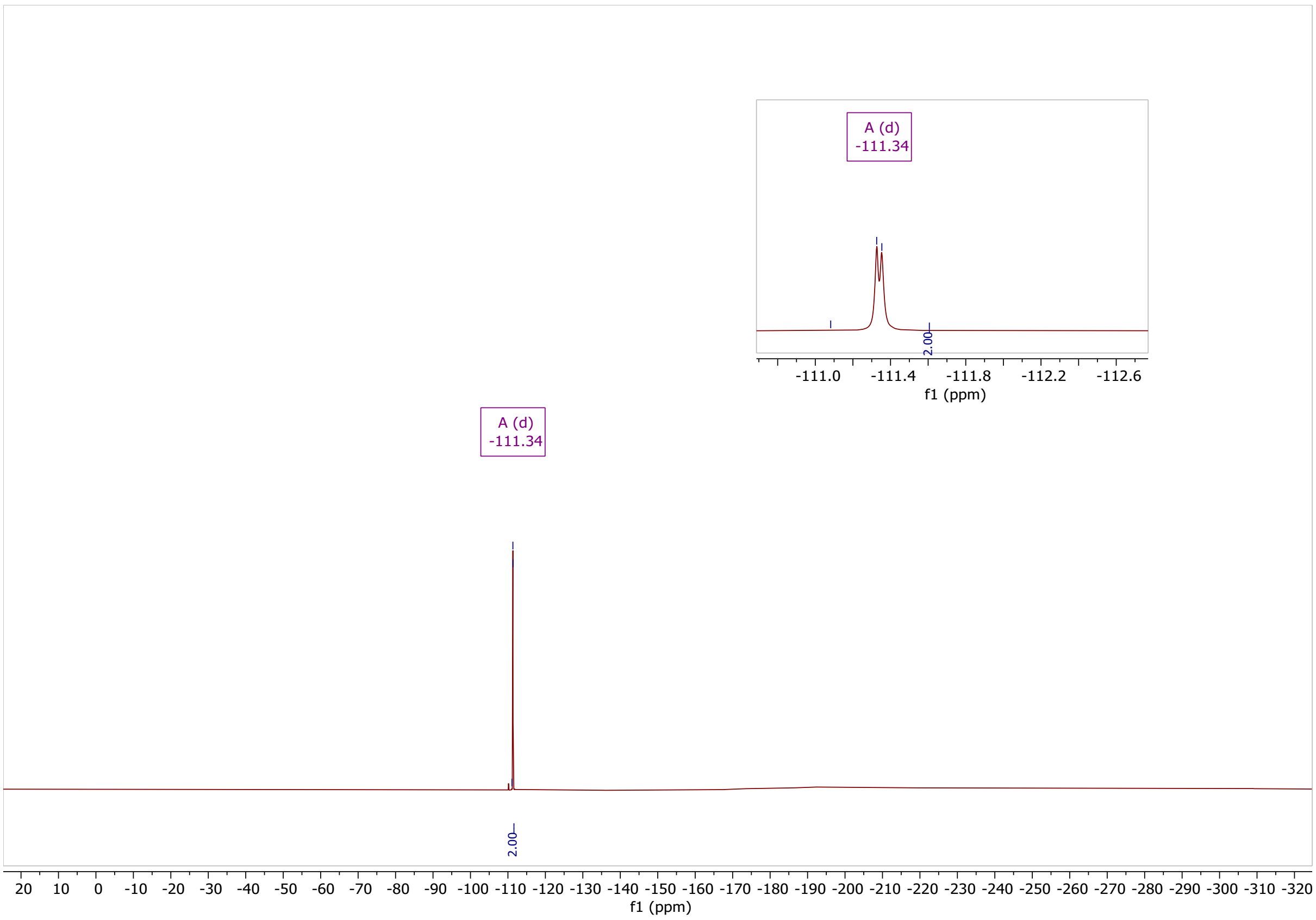

Figure S142 fluorine NMR spectrum of **CA1**

CA2 | *4-(4-Ethoxy-2-fluorophenyl)-2,6-difluorobenzoic acid*

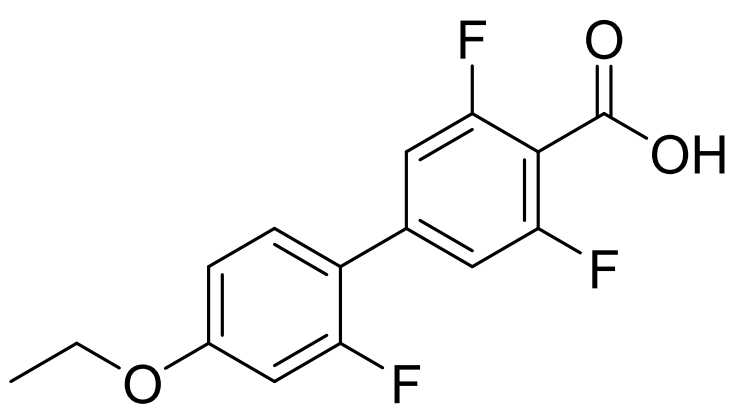

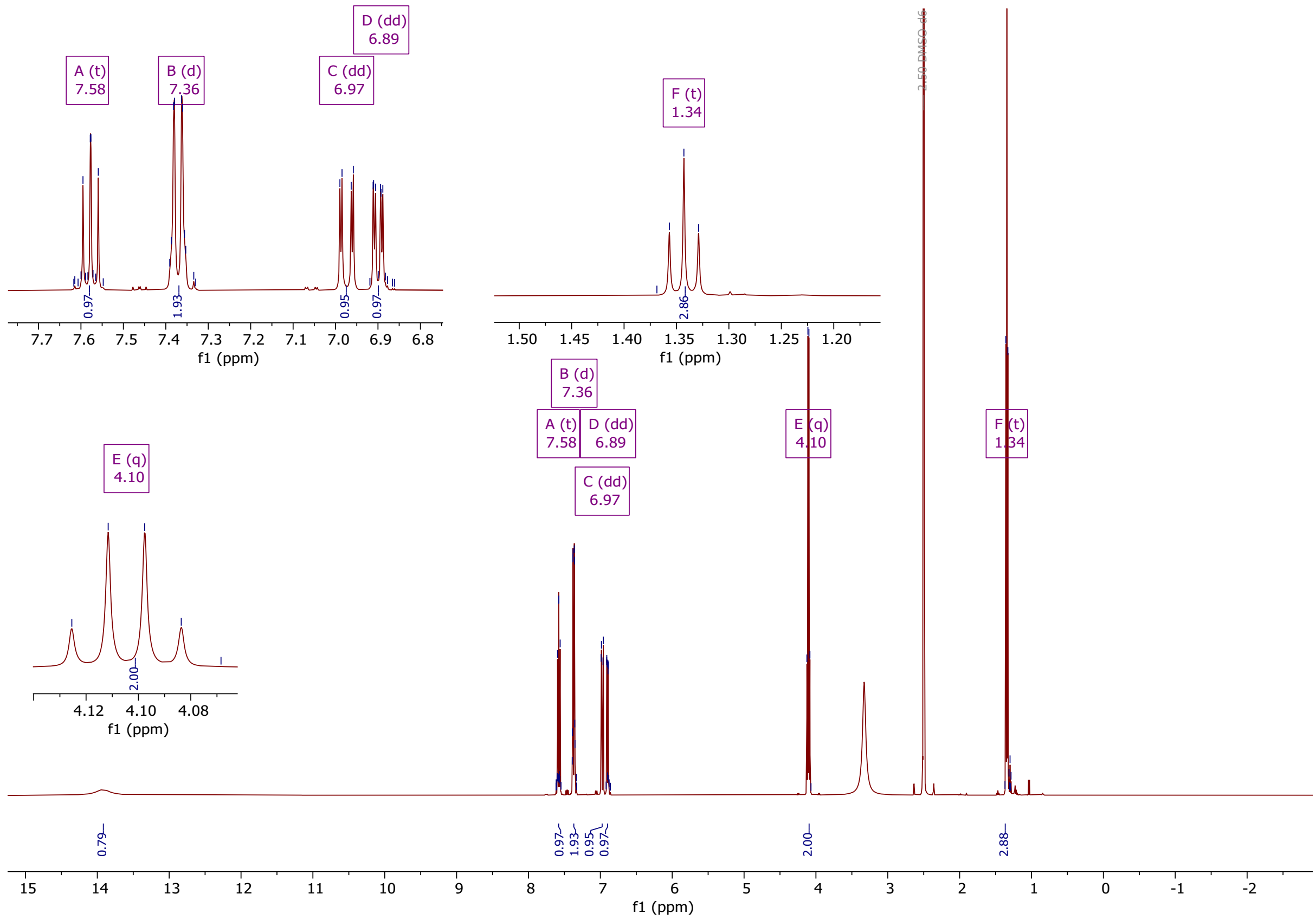

Figure S143 proton NMR spectrum of **CA2**

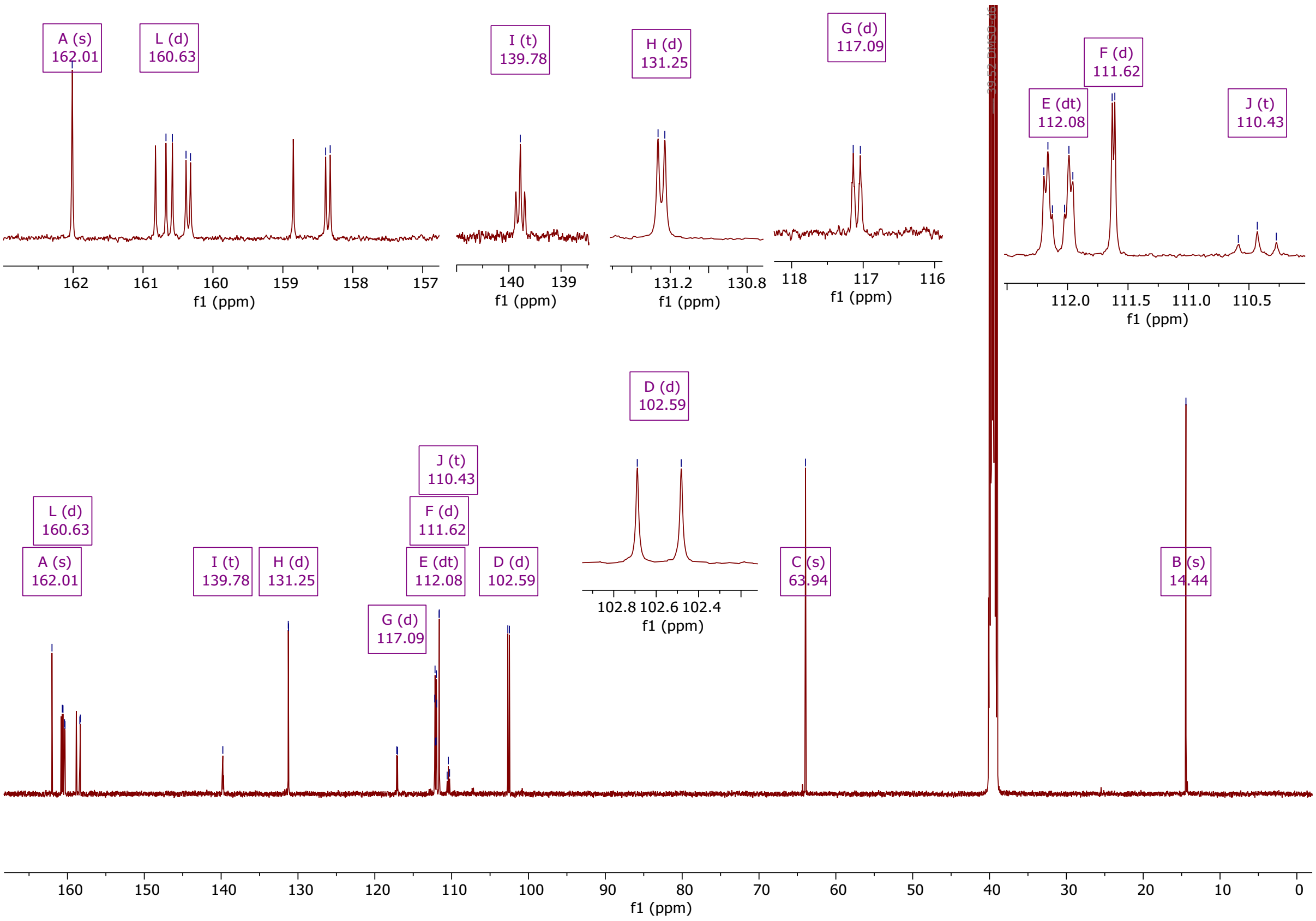

Figure S144 carbon NMR spectrum of **CA2**

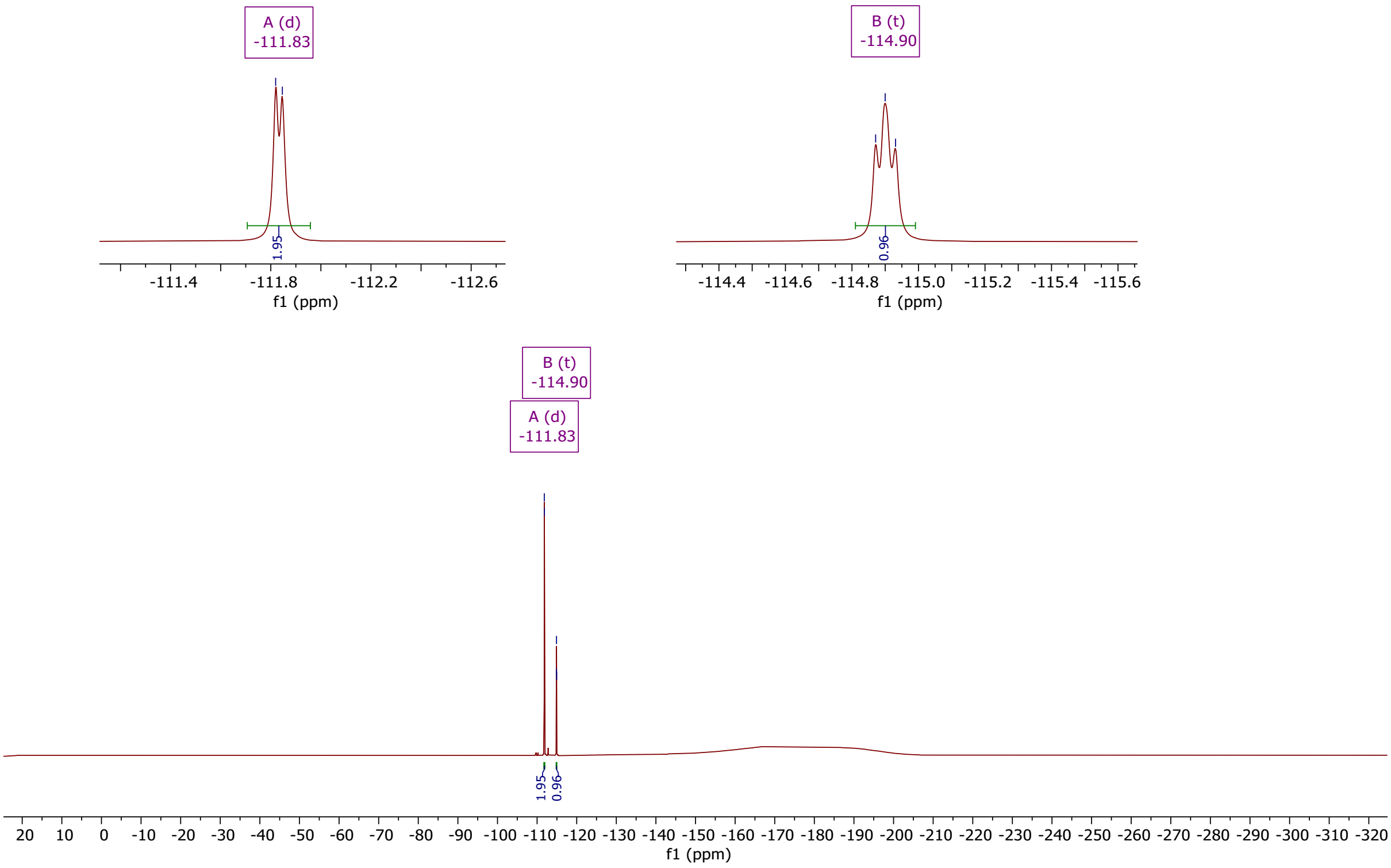

Figure S145 fluorine NMR spectrum of **CA2**

CA3 | *4-(2,4-Dimethoxyphenyl)-2,6-difluorobenzoic acid*

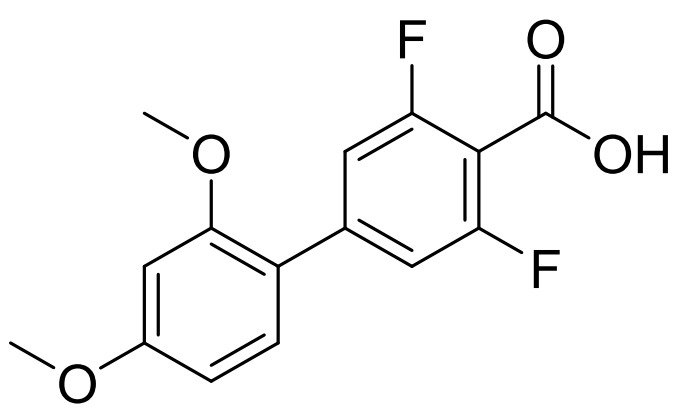

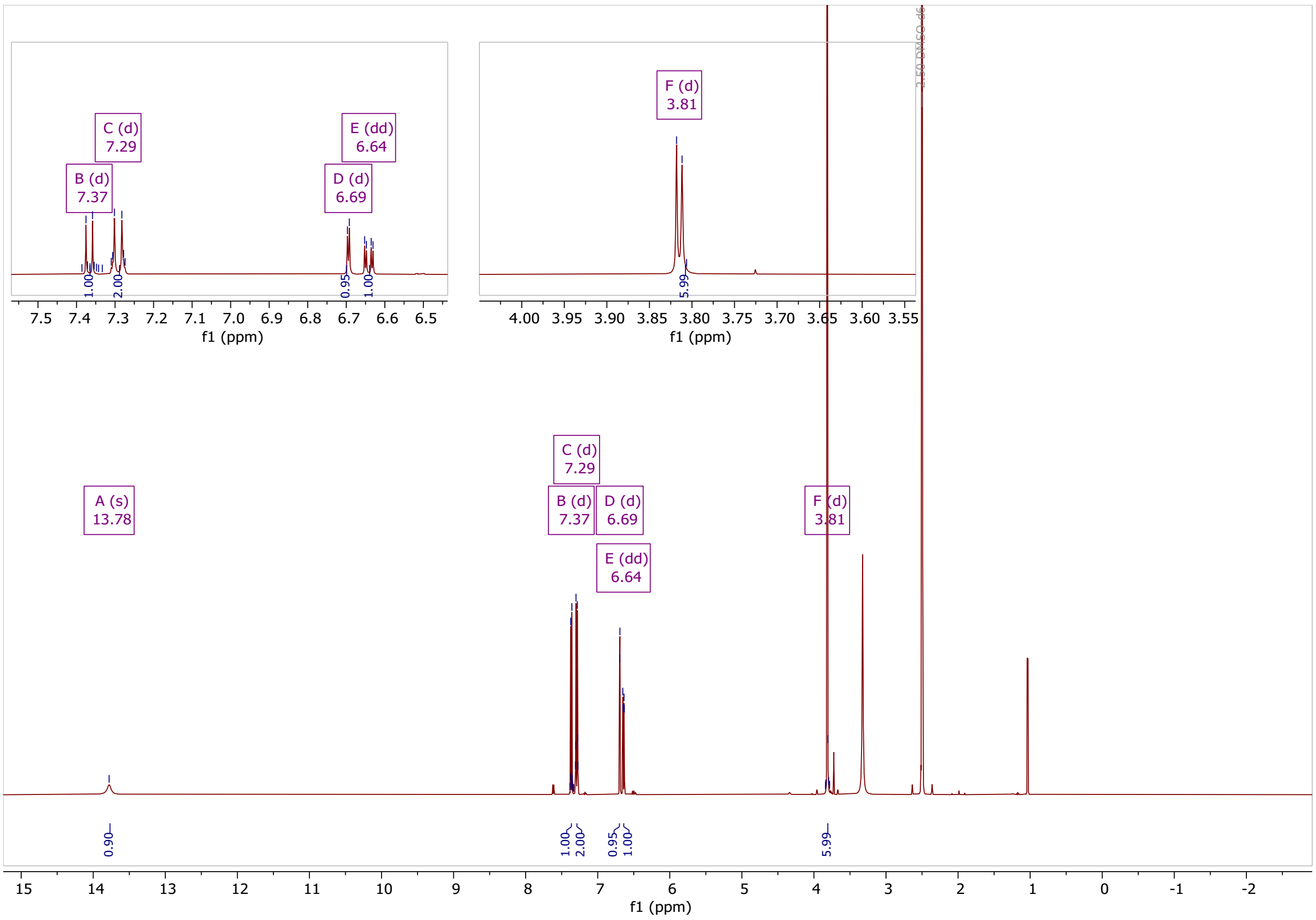

Figure S146 proton NMR spectrum of **CA3**

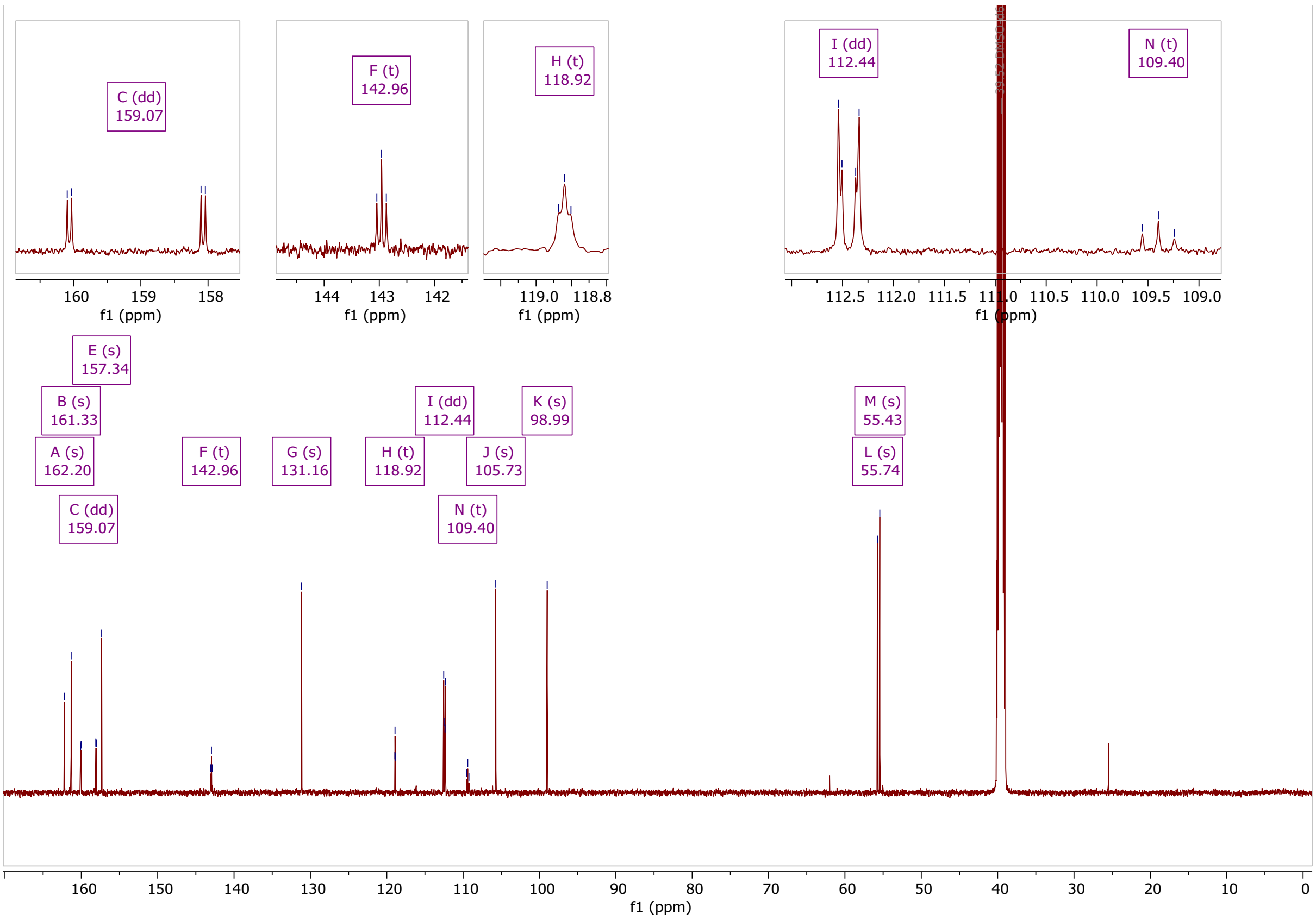

Figure S147 carbon NMR spectrum of **CA3.** Overlapping C=O, and Ar-F bonds do not have multiple labels but are recorded in data set in section 3.

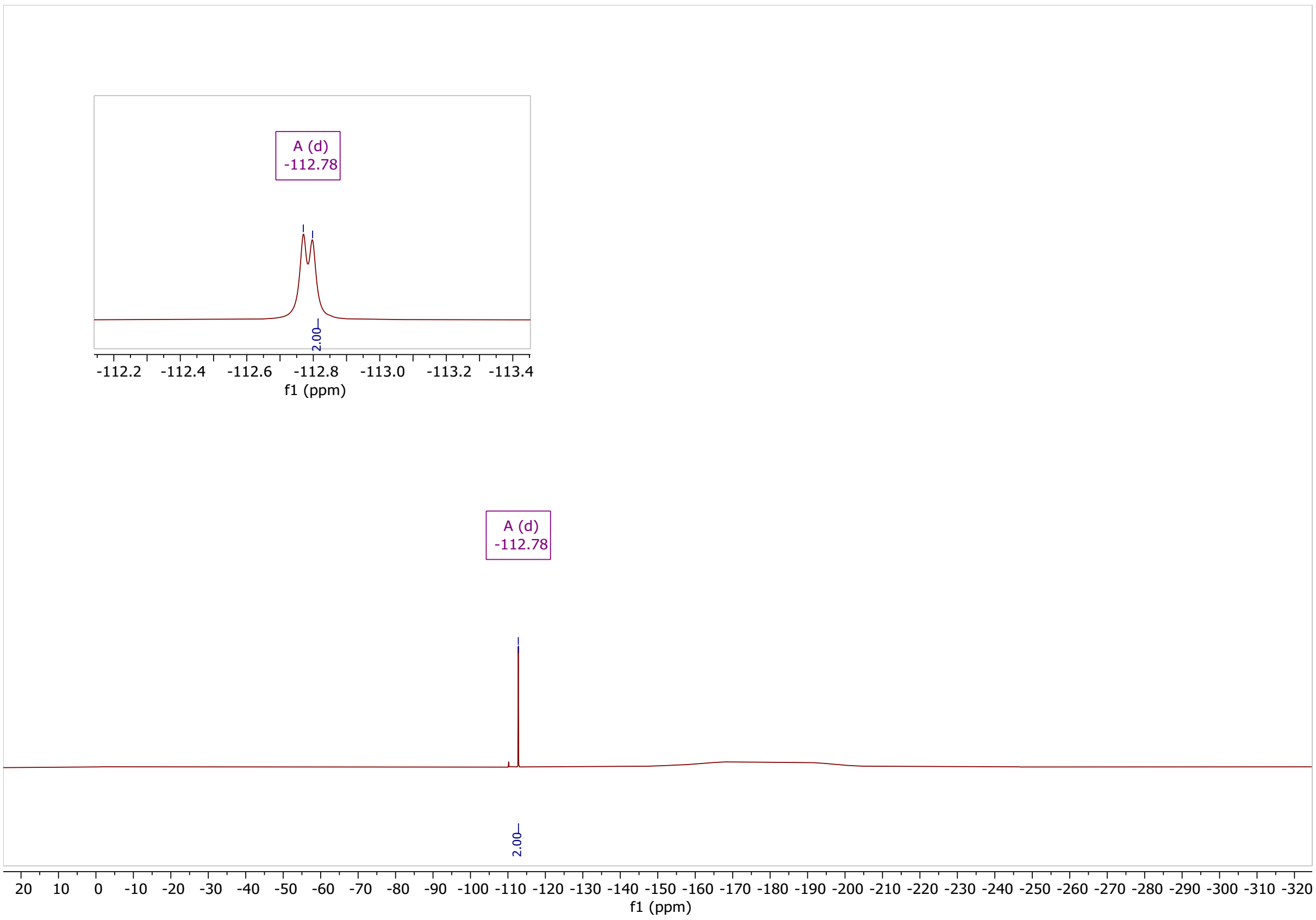

Figure S148 fluorine NMR spectrum of **CA3**

CA4 | *4-(4-Methoxy-2-fluorophenyl)-2,6-difluorobenzoic acid*

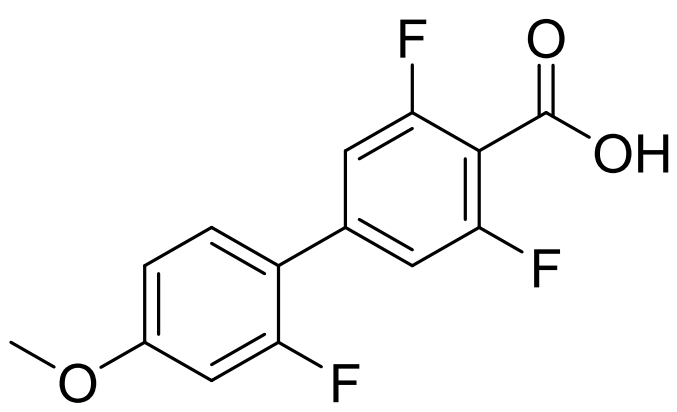

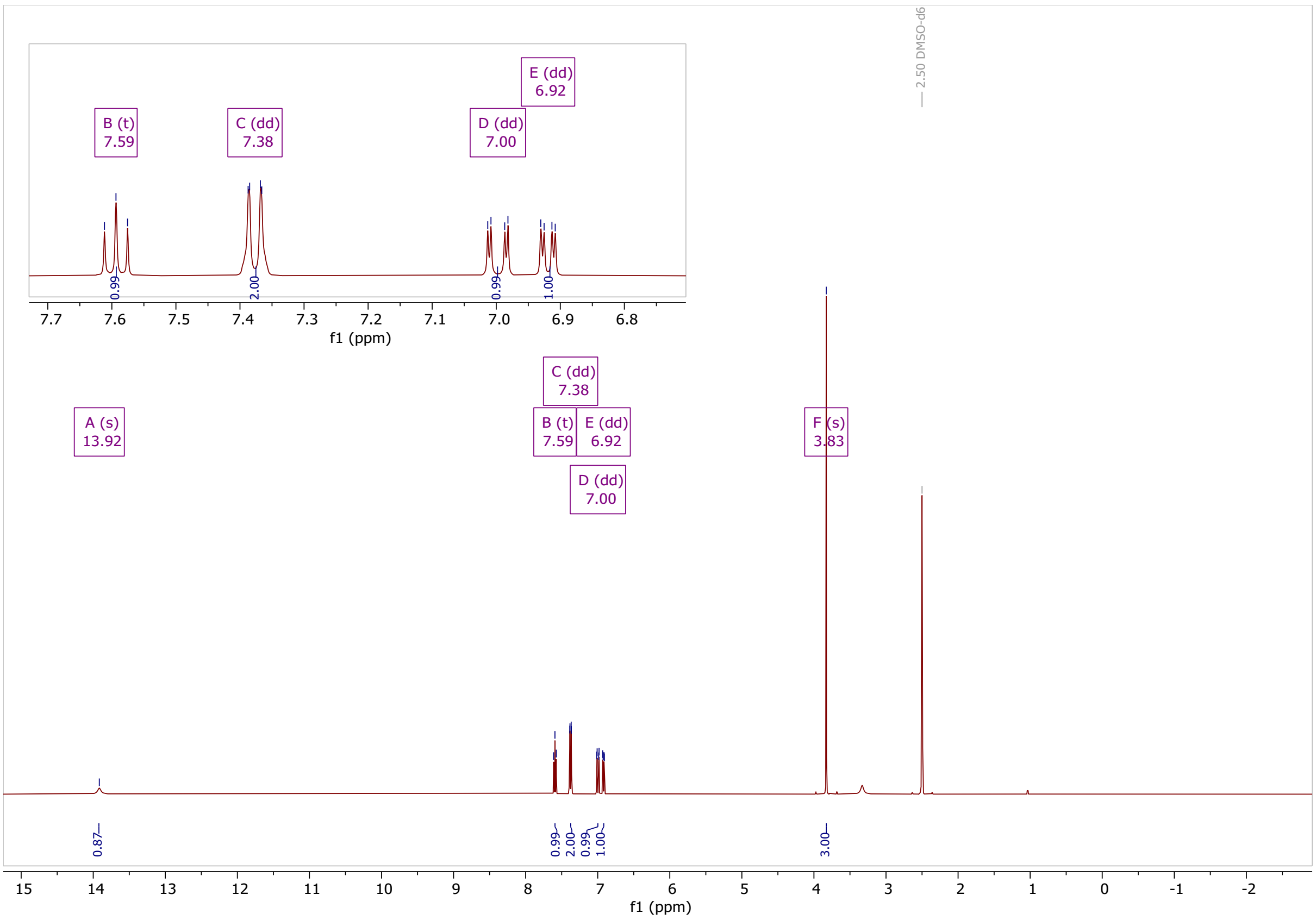

Figure S149 proton NMR spectrum of **CA4**

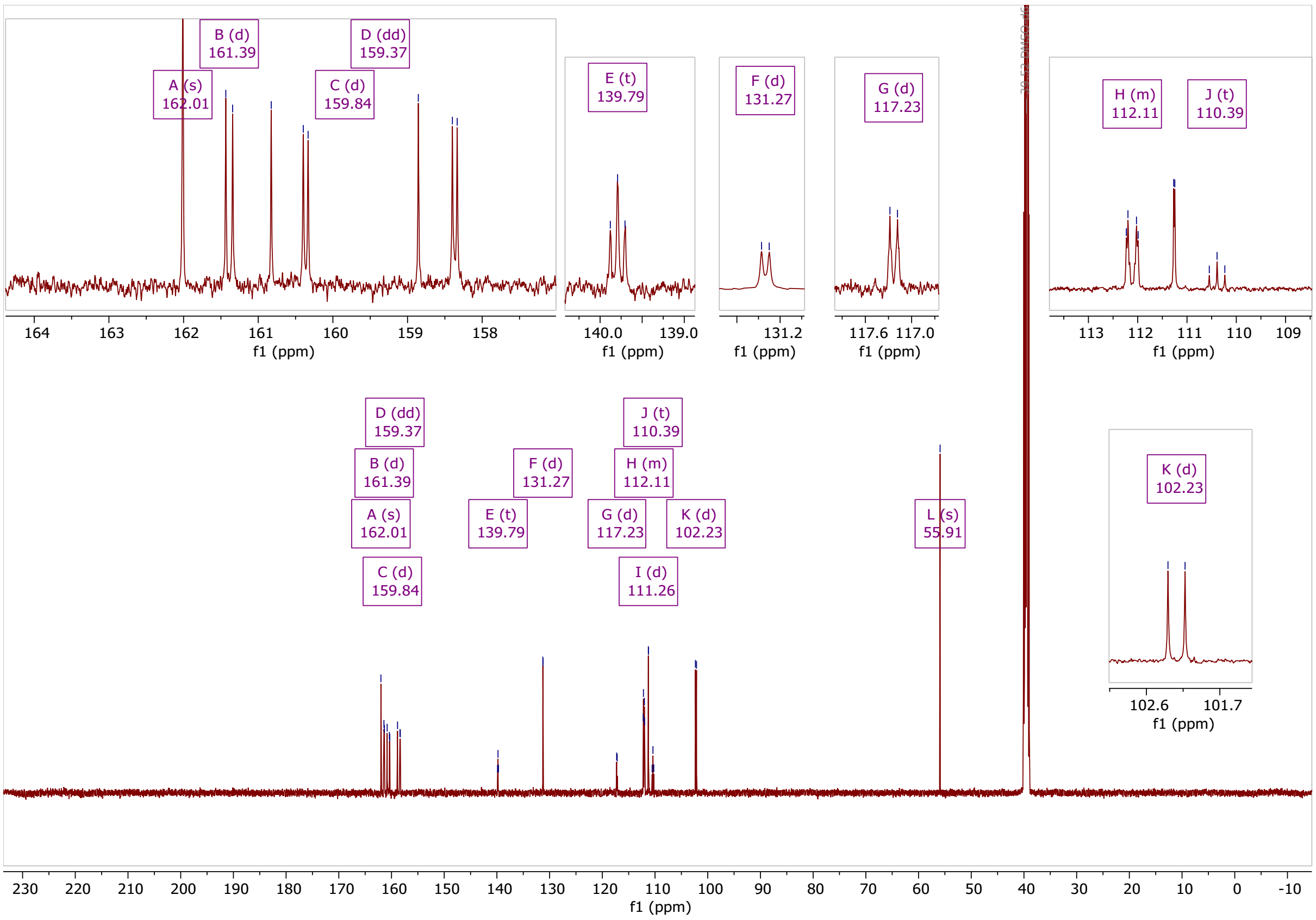

Figure S150 carbon NMR spectrum of **CA4**

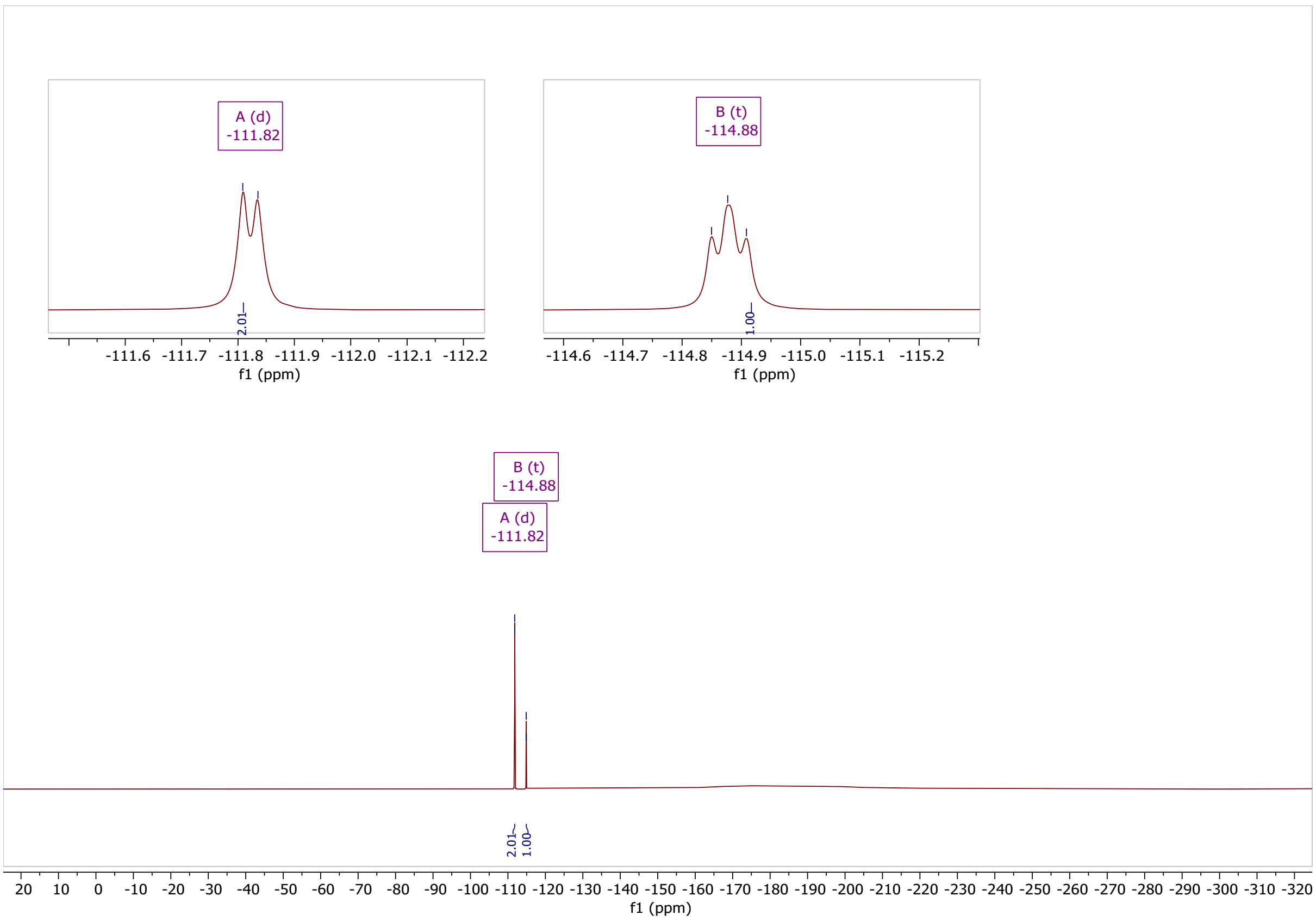

Figure S151 fluorine NMR spectrum of **CA4**
